# Abemaciclib in meningiomas with somatic *NF2* or CDK pathway alterations: the phase 2 Alliance A071401 trial

**DOI:** 10.1038/s41591-025-04141-4

**Published:** 2026-01-16

**Authors:** Priscilla K. Brastianos, Katharine Dooley, Susan Geyer, Elizabeth R. Gerstner, Timothy J. Kaufmann, A. John Iafrate, Maria Martinez-Lage, Mohammed Milhem, Mary Roberta Welch, Thomas J. Kaley, Jan Drappatz, Amy Chan, Priya Kumthekar, Carlos Kamiya Matsuoka, Roy E. Strowd, Adam L. Cohen, Kurt Jaeckle, Lindsay Robell, Rajiv S. Magge, Joo Yeon Nam, Nicholas Blondin, Nawal Shaikh, Ian Rabinowitz, Alissa A. Thomas, David E. Piccioni, Paul Brown, Stefan Kaluziak, Elizabeth Codd, Daniel P. Cahill, Sandro Santagata, Frederick G. Barker, Evanthia Galanis

**Affiliations:** 1 Massachusetts General Hospital Cancer Center, Boston, MA USA; 2 Alliance Statistics and Data Management Center, Mayo Clinic, Rochester, MN USA; 3 Mayo Clinic, Rochester, MN USA; 4 University of Iowa/Holden Comprehensive Cancer Center, Iowa City, IA USA; 5 NYP/Columbia University Medical Center, New York, NY USA; 6 Memorial Sloan Kettering Cancer Center, New York, NY USA; 7 UPMC Hillman Cancer Center, Pittsburgh, PA USA; 8 Dartmouth Hitchcock Medical Center, Boston, MA USA; 9 Alliance Protocol Operations Office, University of Chicago, Chicago, IL USA; 10 MD Anderson Cancer Center, University of Texas, Houston, TX USA; 11 Wake Forest University Health Sciences, Winston-Salem, NC USA; 12 Inova Schar Cancer Institute, Fairfax, VA USA; 13 Mayo Clinic in Florida, Jacksonville, FL USA; 14 University of Michigan Health West, Wyoming, MI USA; 15 Weill Cornell Medical Center, New York, NY USA; 16 Rush University Medical Center, Chicago, IL USA; 17 Smilow Cancer Hospital Care Center, New Haven, CT USA; 18 University of Mississippi Medical Center, Jackson, MS USA; 19 University of New Mexico Cancer Center, Albuquerque, NM USA; 20 University of Vermont Medical Center, Burlington, VT USA; 21 UC San Diego Health, San Diego, CA USA; 22 BWH/Dana-Farber/Partners Cancer Care, Boston, MA USA

**Keywords:** Translational research, CNS cancer, CNS cancer, Targeted therapies

## Abstract

Systemic treatments are limited for patients with meningiomas that have progressed after surgery or radiation. Loss of *NF2* and *CDKN2A/CDKN2B* is common in higher-grade meningiomas and promotes progression in preclinical models. We evaluated the efficacy of abemaciclib, a cyclin-dependent kinase 4/6 inhibitor, as one arm of the Alliance umbrella trial A071401, a genomically driven phase 2 study in recurrent and progressive meningiomas. Eligible patients with grade 2 or 3 tumors and *NF2* mutations or CDK pathway alterations were treated with abemaciclib. Two co-primary endpoints were used: progression-free survival at 6 months (PFS6) and response rate as defined by local review; the trial would be declared positive if either endpoint was met. The success threshold for PFS6 was 8 or more of 24 patients; for the response rate, it was 3 or more of 24 patients. Ninety-six patients were screened and 36 patients received treatment. The mean number of treatment cycles was nine and the median follow-up was 21 months. The first 24 patients who met the eligibility criteria and began treatment could be evaluated for the primary endpoint. The observed PFS6 rate was 58% (14 of 24 patients, 95% confidence interval = 37–78%), thus meeting the PFS6 criteria for promising activity. The best response was stable disease in 16 of 24 patients. Of the 36 patients who started treatment, nine had a grade 3 and two had grade 4 adverse events at least possibly related to treatment. Grade 4 toxicities included alanine aminotransferase elevation (1), aspartate aminotransferase elevation (1) and vomiting (1). The trial met its primary endpoint. Abemaciclib was well tolerated and resulted in improved PFS6. Abemaciclib warrants further investigation for patients with progressive grade 2 or 3 meningiomas harboring *NF2* or CDK pathway alterations. ClinicalTrials.gov registration no. NCT02523014.

## Abstract (web summary)

In an arm of an ongoing multicenter phase 2 trial testing different therapies in patients with genetically profiled grade 2 or 3 meningiomas, treatment with an oral CDK4/6 inhibitor met the primary endpoint for progression-free survival at 6 months in patients with CDK or *NF2* alterations.

## Main

Meningiomas, the most common primary brain tumors, have limited treatment options once surgery and radiation fail^1^. Several systemic therapies have been evaluated in patients with recurrent and progressive meningiomas, with limited success^1^. The field of meningioma investigation has historically been hampered by the lack of knowledge of the molecular drivers of meningioma. This is rapidly changing, as over the last decade, seminal papers have characterized the genetic landscape of meningioma^2–5^ and opened up new avenues for the diagnosis, classification and treatment of meningiomas^6–8^.

Several recent studies have demonstrated that meningiomas harbor potentially clinically actionable alterations^2–4,9,10^. Genetic changes such as *TERT*-promoter mutations^10–12^ and *CDKN2A* and *CDKN2B* loss are prognostic^13^ and now incorporated into the World Health Organization (WHO) guidelines for the classification of grade 3 meningioma^14^. In addition to loss of function mutations in the well-established *NF2* gene, a subset of meningiomas harbor alterations in *SMO, AKT* and *PIK3CA*^2,3,15^ that are potentially targetable with existing systemic therapy. These insights into the genetic changes of meningiomas led to the design of Alliance for Clinical Trials in Oncology (Alliance) A071401, a National Cancer Institute-supported Cooperative Group genomically driven trial for patients with progressive or recurrent meningioma. This is the first national study to investigate the role of precision medicine in genetically defined subclasses of meningioma. One arm of the study evaluating FAK inhibition in patients with progressive and recurrent meningiomas met its primary endpoint and was published elsewhere^16^.

Cyclin-dependent kinase (CDK) signaling pathway alterations are frequent in high-grade meningiomas^5,13,17–19^. In mouse models, *CDKN2A* and *CDKN2B*, and *NF2*, loss promotes meningioma progression^20^; pharmacological inhibition with CDK4 and CDK6 inhibitors has promising activity in in vitro and in vivo models^21–23^. Thus, we sought to investigate the clinical activity of abemaciclib, an oral continuous dosing CDK4 and CDK6 inhibitor^24^, in high-grade meningioma. We present the results of the CDK inhibitor arm of Alliance A071401, where we evaluated the efficacy of abemaciclib in patients with recurrent or progressive grade 2 or 3 meningioma harboring CDK pathway or *NF2* alterations.

## Results

### Patients

Alliance A071401 is a multi-arm genomically driven phase 2 study evaluating the efficacy of targeted therapies in cohorts of patients harboring specific genetic mutations. Patients with recurrent or progressive grade 2 or 3 meningiomas harboring the CDK pathway or *NF2* mutations who met the eligibility criteria were enrolled in the abemaciclib arm. Key eligibility criteria included grade 2 or 3 meningioma, measurable disease, presence of an alteration in the CDK pathway or in *NF2* in the tumor tissue, progressive or residual disease as defined by (1) residual measurable disease immediately after surgery for grade 2 or 3 disease; (2) progressive measurable disease (increase in the size of the measurable lesion on imaging by 25% or more in 25 months); or (3) progressive disease after radiation. The co-primary endpoints were progression-free survival at 6 months (PFS6) and response rate (RR) after starting treatment. Secondary endpoints included overall survival, progression-free survival, toxicity and activity according to the central radiology review. Exploratory endpoints included genetic and histological biomarkers of response.

**Fig. 1:**
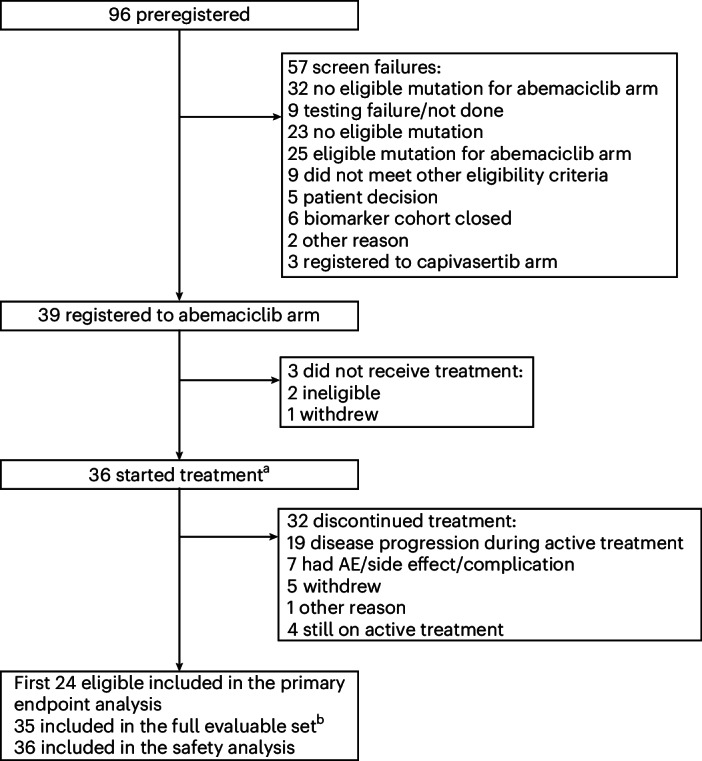
CONSORT diagram of patient population. Ninety-six patients were identified as having *NF2* or CDK pathway alterations while the arm was open to accrual. ^a^One patient with a grade 1 tumor was registered in error and was considered unevaluable for the primary endpoint analyses. ^b^Two patients withdrew from all follow-up before 6 months and did not have an event before withdrawal; one patient withdrew consent to the clinical follow-up and tumor assessment data were not submitted before 6 months.

During the period that the abemaciclib arm was open from 15 September 2021 to 3 October 2022, 96 patients were screened to the study. The median time from baseline scan to start of protocol treatment was 9.5 days (interquartile range (IQR) = 7.0–14.5 days). Sixty-four of these patients had eligible mutations and 39 were registered to the abemaciclib arm (Fig. 1). The trial overaccrued because we allowed patients to start treatment if they were undergoing screening when the accrual was complete, given the lack of other standard treatment options for these patients. Thirty-six patients started treatment. One patient with a grade 1 tumor was registered in error and was considered unevaluable for the primary endpoint analyses. The first 24 eligible patients who began treatment were considered assessable for evaluation of the primary endpoint decision rule, as per the prespecified study design (Table 1 and Supplementary Table 1). As the trial overaccrued, demographics, PFS6, RR and secondary endpoints are also reported in all 35 evaluable patients (Supplementary Table 1).

**Table 1:** Demographics of patients enrolled to the abemaciclib arm

Demographics	First 24 patients enrolled	Safety population (*n* = 36)
Age (years)		
Mean (s.d.)	61.9 (12.8)	62.9 (11.8)
Median	63.5	63.5
Q1, Q3	52.5, 71.5	54.5, 73.5
Range	39.0–84.0	39.0–84.0
Sex		
Female	14 (58.3)	22 (61.1)
Male	10 (41.7)	14 (38.9)
Ethnicity		
Asian	1 (4.2)	1 (2.8)
Black or African American	5 (20.8)	5 (13.9)
Native Hawaiian or Pacific Islander	0 (0)	1 (2.8)
Not reported: patient refused or not available	1 (4.2)	1 (2.8)
Unknown: patient unsure	0 (0)	2 (5.6)
White	17 (70.8)	26 (72.2)
Ethnic group		
Hispanic or Latino	2 (8.3)	6 (16.7)
Not Hispanic or Latino	22 (91.7)	28 (77.8)
Unknown: patient unsure	0 (0)	2 (5.6)
ECOG performance status		
0	7 (29.2)	10 (27.8)
1	14 (58.3)	22 (61.1)
2	3 (12.5)	4 (11.1)
Central Review WHO grade		
1	0 (0)	1 (2.8)
2	15 (62.5)	20 (55.6)
3	9 (37.5)	15 (41.7)
Status of tumor at registration		
Both	7 (29.2)	9 (25.0)
Progressive measurable disease	14 (58.3)	19 (52.8)
Residual measurable disease	3 (12.5)	8 (22.2)
Multifocal disease		
Yes	12 (50.0)	17 (47.2)
No	12 (50.0)	19 (52.8)
Corticosteroid therapy at study entry		
No	19 (79.2)	28 (77.8)
Yes	5 (20.8)	8 (22.2)
Previous surgery related to this tumor		
Yes	24 (100.0)	36 (100.0)
Prior radiation therapy for this tumor		
Yes	23 (95.8)	35 (97.2)
No	1 (4.2)	1 (2.8)^a^
Prior systemic (cancer) therapy for this tumor		
Yes	9 (37.5)	13 (36.1)
No	15 (62.5)	23 (63.9)
Any prior cancer diagnosis		
Yes	3 (12.5)	5 (13.9)
No	21 (87.5)	31 (86.1)
Number of prior treatment modalities		
1	1 (4.2)	2 (5.6)
2	14 (58.3)	21 (58.3)
3	9 (37.5)	13 (36.1)

Data are shown as *n* (%) unless stated otherwise. The first 24 patients enrolled were included in the protocol-specified primary endpoint analysis. The safety population includes all patients consented who started treatment and could be evaluated for AEs.

^a^The reasons for no prior radiation therapy in this patient were mutation in a tumor suppressor gene leading to higher risk of additional tumor growth with radiation, other therapeutic options available and transportation challenges.

Across the first 24 patients enrolled to the study, the median age at enrollment was 64 years (range = 39–84), and 14 were female (58%). Most patients had an Eastern Cooperative Oncology Group performance (ECOG) status of 0 or 1 at study entry (21 of 24 = 88%). Fifteen patients had grade 2 (63%) and nine (38%) had grade 3 meningiomas. All patients had undergone surgery for their meningioma (100%), 23 had received radiation (96%) and nine had received prior medical therapy (38%). Twenty-three patients (96%) had previously received at least two treatment modalities (Table 1).

### Efficacy

**Fig. 2:**
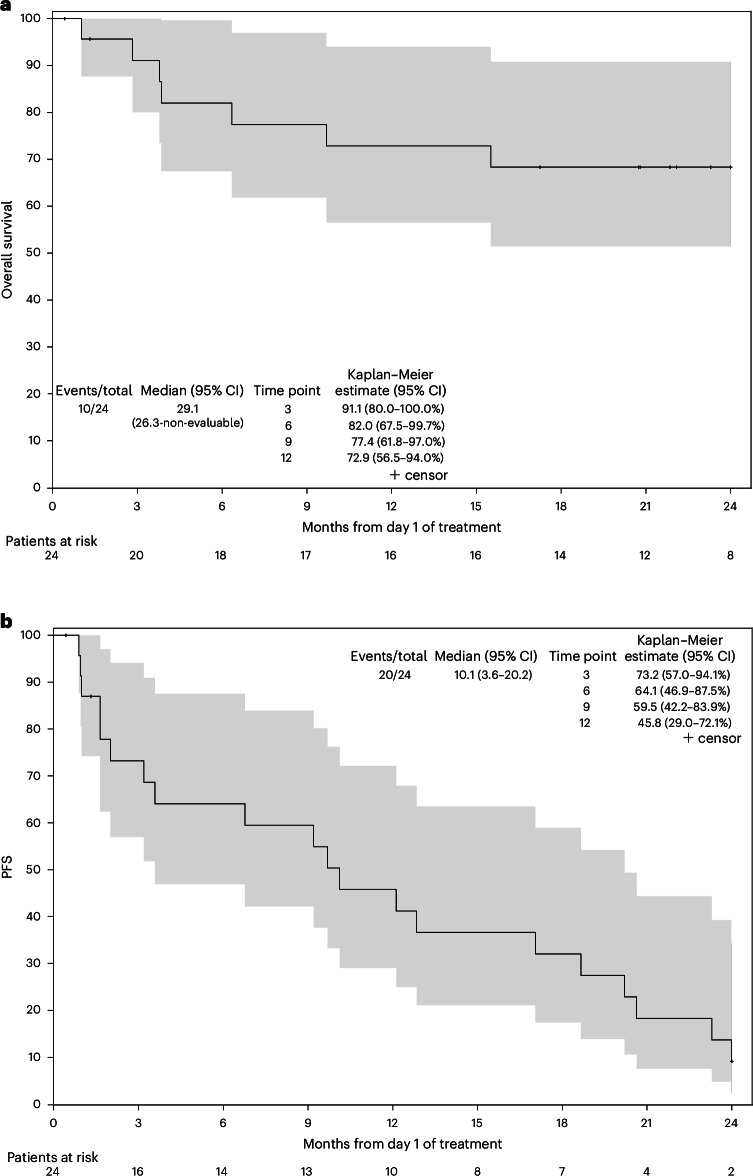
Kaplan–Meier curves for the first 24 evaluable patients treated with abemaciclib on the Alliance A071401. **a**,**b**, Overall survival (**a**) and PFS (**b**). Median overall survival was 29.1 months and median PFS was 10.1 months.

**Extended Data Fig. 1:**
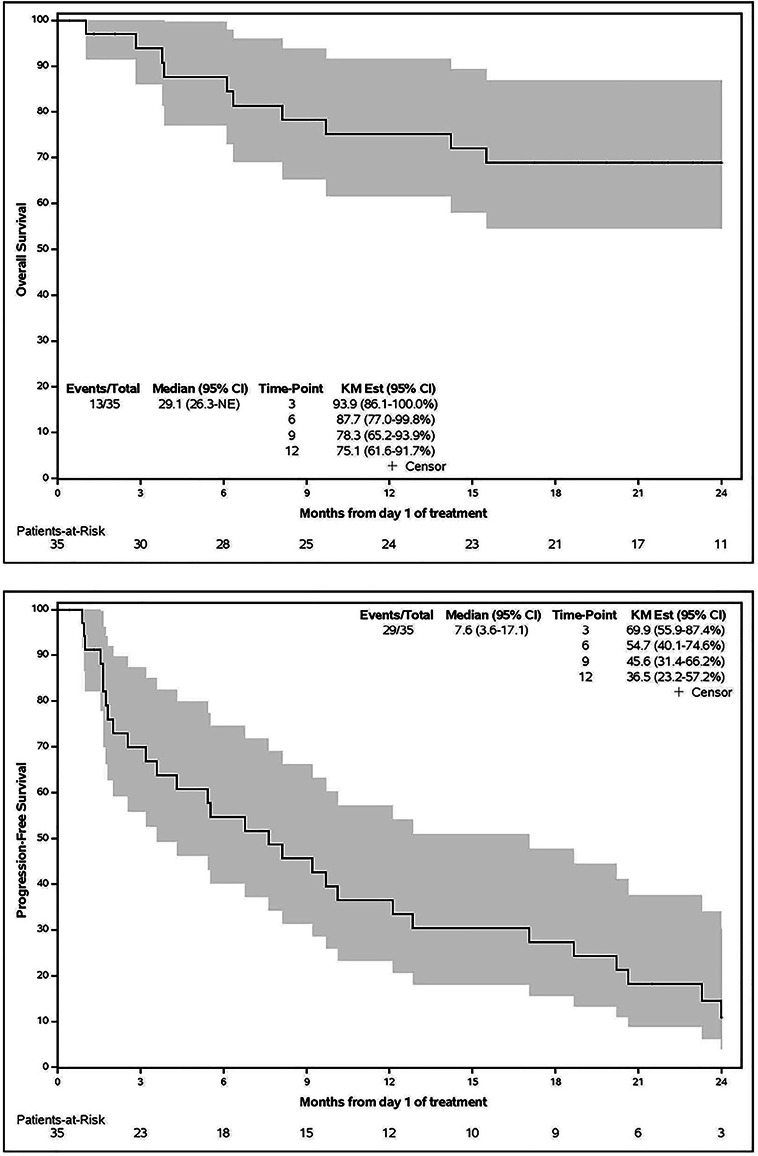
Kaplan-Meier curves of overall survival (top) and progression-free survival (bottom) for all 35 evaluable patients treated with abemaciclib on Alliance A071401. Median overall survival was 29.1 months and median progression-free survival was 7.6 months. KM, Kaplan-Meier; NE, not evaluable.

**Extended Data Fig. 2:**
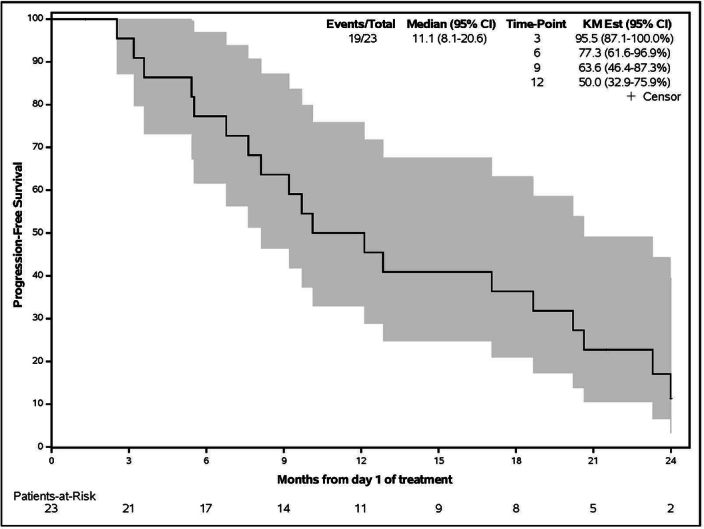
Kaplan-Meier curve of progression-free survival for evaluable patients with best response of stable disease treated with abemaciclib on Alliance A071401. Median PFS was 11.1 months. KM, Kaplan-Meier.

The median follow-up since the start of treatment was 21 months. At 6 months, 14 of the first 24 patients (58%, 95% confidence interval (CI) = 36.6–77.9%, 90% CI = 39.7–75.4%) were progression-free and alive (Table 2 and Extended Data Table 1). The best response was stable disease, which was observed in 16 of 24 patients (67%, 95% CI = 44.7–84.4%). No objective complete or partial responses were observed (Table 2 and Extended Data Table 2). Per the prespecified trial criteria, the requirement for a successful PFS6 endpoint was eight patients being progression-free and alive at 6 months. Thus, the PFS6 endpoint was met and the trial met the overall criteria for abemaciclib to be considered worthy of further study in this patient population. Central radiology review was conducted for 21 patients on the study with scans available for review through the Imaging Central Lab. Of those 21 patients, 12 (57%) patients were progression-free at 6 months as defined by Macdonald criteria measurements, and 12 (57%) were PFS6 as defined by volumetric measurements, thus meeting the primary endpoint according to the central review (Extended Data Table 3). The median overall survival was 29.1 months for the first 24 evaluable patients (95% CI = 26.3–not evaluable) and was the same when all 35 evaluable patients were included (Fig. 2 and Extended Data Fig. 1). The median PFS (Fig. 2) was 10.1 months (95% CI = 3.6–20.2) for the first 24 evaluable patients enrolled and 7.6 months (Extended Data Fig. 1) for all 35 evaluable patients (95% CI = 3.6–17.1). The median PFS among the 23 patients with a best response of stable disease in the overall cohort was 11.1 months (95% CI = 8.1–20.6 months) (Extended Data Fig. 2).

**Table 2:** PFS6 and best response by 12 months in the first 24 evaluable patients^a^

PFS6		
PFS6	*n* (%)	95% CI
Progression-free and alive	14 (58.3)	36.6–77.9
Progression or death	10 (41.7)	22.1–63.4

RR according to the Macdonald response criteria (best response by 12 months)	
Response type	*n* (%)
Response (partial response or complete response)	0
Stable disease	16 (66.7)
Progressive disease	6 (25.0)
Non-evaluable	2 (8.3)

^a^Strictly according to registration order, including patients who were not followed for at least 6 months and withdrew consent. Patients who withdrew consent before 6 months were included in the denominator for the PFS6 proportion calculation.

### Toxicity

All 36 patients who started the protocol treatment (including the one patient with a grade 1 tumor) were evaluated for toxicity (Table 3 and Supplementary Tables 2–6). The mean number of cycles of abemaciclib received was nine (s.d. = 9.9) (Supplementary Table 2). Twelve patients (33%) had a treatment delay in at least one cycle. Seven patients discontinued treatment because of side effects, adverse event (AEs) or complications. Nine patients had grade 3 events and two patients had grade 4 events at least possibly related to treatment (Table 3 and Supplementary Tables 3 and 4). Grade 4 treatment-related events were aspartate aminotransferase (AST)/alanine aminotransferase (ALT) elevation and vomiting.

**Table 3:** Listing of grade 3+ AEs, and maximum grade per patient per event, possibly related to treatment (*n* = 36)

	Grade of adverse event		
	3 (severe)	4 (life-threatening)	5 (lethal)
	*n* (%)	*n* (%)	*n* (%)
**Hematological AEs**			
Blood/bone marrow			
Anemia	2 (6)	0 (0)	0 (0)
Lymphocyte count decreased	1 (3)	0 (0)	0 (0)
Neutrophil count decreased	2 (6)	0 (0)	0 (0)
White blood cell decreased	1 (3)	0 (0)	0 (0)
**Non-hematological AEs**			
Eye disorders			
Blurred vision	1 (3)	0 (0)	0 (0)
Gastrointestinal disorders			
Diarrhea	2 (6)	0 (0)	0 (0)
Vomiting	0 (0)	1 (3)	0 (0)
Generalized disorders and administration site conditions			
Fatigue	2 (6)	0 (0)	0 (0)
Investigations			
ALT increased	1 (3)	1 (3)	0 (0)
AST increased	0 (0)	1 (3)	0 (0)
Weight loss	1 (3)	0 (0)	0 (0)
Metabolic and nutrition disorders			
Dehydration	1 (3)	0 (0)	0 (0)
Hyperkalemia	1 (3)	0 (0)	0 (0)
Hyponatremia	1 (3)	0 (0)	0 (0)
Nervous system disorders			
Dizziness	1 (3)	0 (0)	0 (0)
Seizure	1 (3)	0 (0)	0 (0)
Renal and urinary disorders			
Acute kidney injury	1 (3)	0 (0)	0 (0)
Vascular disorders			
Thromboembolic event	1 (3)	0 (0)	0 (0)

### Exploratory

**Fig. 3:**
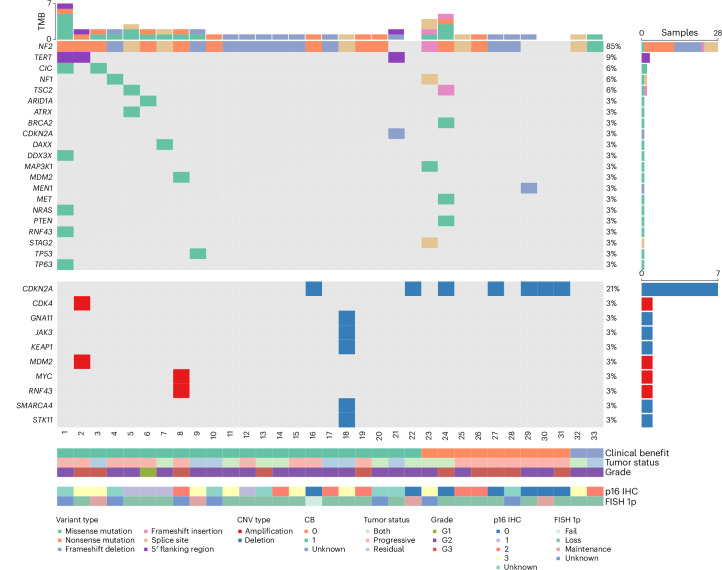
Comutation plot showing the mutational landscape in trial patients with meningioma. The top and side histograms represent the tumor mutational burden (TMB) and the fraction of reported variants per sample or gene, respectively. CNV, copy number variant.

**Extended Data Fig. 3:**
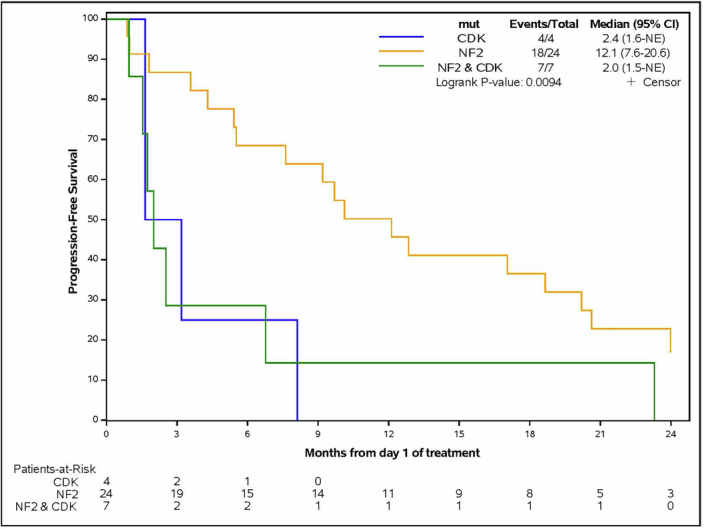
Progression-free survival across mutational subgroups for patients treated with abemaciclib on Alliance A071401, in patients harboring alterations in NF2, the CDK pathway or both.

We evaluated the mutational landscape of tumors collected as part of this arm of the study (Fig. 3). Patients with *NF2* alterations had favorable PFS6 outcomes (15 of 24, 62.5%, 95% CI = 41–81%) compared to those with the CDK pathway (1 of 4, 25%, 95% CI = 1–81%) and CDK pathway and *NF2* alterations (2 of 7, 29%, 95% CI = 4–71%), although this difference did not approach statistical significance (Fisher’s exact test *P* = 0.205). However, patients with *NF2* alterations had significantly longer PFS (12.1 months, 95% CI = 7.6–20.6) compared to those with the CDK pathway (2.4 months, 95% CI = 1.6–not evaluable) and CDK pathway and *NF2* alterations (2.0 months, 95% CI = 1.5–not evaluable) (log-rank *P* = 0.0094) (Extended Data Fig. 3). Notably, no other mutations evaluated were associated with PFS6.

**Extended Data Fig. 4:**
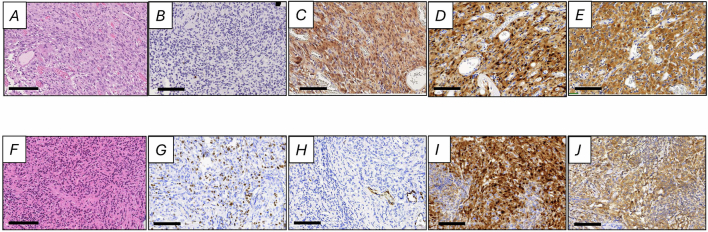
Representative histologic analysis of meningioma with clinical benefit (panels A-E) and case with no benefit (panels F-J). H&E stains (**A**,**B**), immunostains for ki67 (**B**,**G**), p16 (**C**,**H**), cyclin **D** (**D**,**I**) and CDK4 (**E**,**J**). Size bar = 100 microns. Summary statistics across the cohort of samples are presented in Extended Data Table 4.

We evaluated Ki-67, p16, CDK4 and cyclin D1 with immunohistochemistry (IHC) and 1p loss using fluorescence in situ hybridization (FISH) (Extended Data Fig. 4 and Extended Data Table 4). There were no significant differences observed in the distribution of Ki-67, cyclin D1, CDK4 or the 1p-to-1q ratio between patients who had clinical benefit (CB) from abemaciclib and those who did not, although some trends could be observed. Patients who had clinical benefit from abemaciclib had a greater mean p16 than those who did not clinically benefit (mean ± s.d.: CB, 1.8 ± 1.1 versus no CB, 0.78 ± 1.2, *P* = 0.04).

## Discussion

Patients with meningiomas that have progressed or recurred after surgery or radiation have a paucity of therapeutic options^25^. Historically, most clinical trials evaluating systemic therapy for meningiomas have yielded disappointing results^1^. Recent studies have provided insights into the genetic landscape of meningiomas, revealing potentially clinically actionable mutations in these tumors^2–4^. Based on these studies, we designed Alliance A071401, the first national genomically driven trial for patients with recurrent or progressive meningiomas harboring specific genetic alterations. We previously presented data on the FAK inhibitor arm of this study and showed that in 36 eligible patients with *NF2* alterations who received the drug GSK2256098, the PFS6 rate was 83% in grade 1 patients and 33% in grade 2 or 3 patients and met the primary endpoint in both cohorts^16^. However, GSK2256098 is no longer being developed, highlighting the need for additional drugs to evaluate in this patient population. With this unmet need in mind, and given the frequent *CDKN2A* and *CDKN2B* alterations in high-grade meningiomas, we evaluated abemaciclib in patients with grade 2 or 3 meningiomas harboring the CDK pathway or *NF2* alterations. The trial encouragingly met the primary endpoint with a PFS6 of 58% and a median PFS of 10.1 months in the primary evaluation cohort. Abemaciclib showed a similar AE profile to what has previously been reported in patients receiving these inhibitors in other cancers^24,26^.

We chose to include both CDK pathway and *NF2* alterations as eligibility criteria for the abemaciclib arm for several reasons. First, *CDKN2A*, *CDKN2B* and *NF2* loss frequently co-occur in high-grade meningiomas^20^. Mouse models have demonstrated that meningioma progression is triggered by *NF2* and *CDKN2A* and *CDKN2B* loss^20,27^. *NF2* (merlin) loss upregulates cell-cycle targets, which leads to increased cell proliferation and cell-cycle progression in meningioma^28^; this further strengthens the rationale to investigate CDK4 and CDK6 inhibitors in patients with meningioma^22^. Preclinical data suggest that CDK4 and CDK6 inhibition shows encouraging activity in cell lines^22,23^. Second, the optimal predictive biomarkers for response to CDK inhibition are not yet known. Some data suggest that higher CDK4 levels may be associated with response to CDK4 and CDK6 inhibition in breast cancer^29^. Clinical benefit, including durable responses, has been reported in patients across other cancers with genetic alterations in the CDK pathway receiving CDK inhibitors^30–33^, although the results in genomically driven studies have been mixed^34–36^. Specifically, in meningiomas, data from preclinical models suggest that CDK4 and CDK6 inhibition has activity in meningioma models of different genetic contexts, including those harboring alterations in either *NF2* or *CDKN2A* and *CDKN2B*^22^. Interestingly, in our study, CDK pathway alterations, and more specifically *CDKN2A* and *CDKN2B* loss, were associated with a poor prognosis, whereas PFS was better in patients with mutation of *NF2*, although numbers were small. More investigations are needed to identify the optimal biomarkers of response for patients with meningioma receiving abemaciclib, and to investigate additional combinatorial strategies for the treatment of patients with CDK-altered tumors.

One limitation of this study is the lack of a control arm. Given the lack of effective systemic therapies in this setting, there was no appropriate control arm for this study because there is no standard-of-care option after surgery and radiation have failed. Because this trial was designed in 2015, the PFS6 primary endpoint was selected based on historical control data from a comprehensive systematic review of prior trials of systemic therapies in patients with meningioma^1^. Recent prospective clinical trials have used a similar PFS6 as an endpoint for this patient population^37,38^. Nevertheless, the results of this study compare favorably to other trials in similar populations of heavily pretreated patients with recurrent or progressive meningioma^1^, with the caveat that prior studies were not genomically driven, making comparisons across studies challenging. A more recent pooled analysis from the RANO group, published in 2025 after the abemaciclib arm of our trial completed accrual, reported a pooled PFS6 of 38% across clinical trials published in grade 2 or 3 meningiomas; these new RANO benchmarks merit consideration in the design and interpretation of future meningioma trials. Of note, our reported PFS6 of 58% (90% CI = 39.7–75.4%) also compares favorably with this newer historical control^39^. The RR endpoint was selected because historically systemic therapy has had poor RRs in patients with recurrent meningioma^1^. In this study, we observed stable disease as the best response in most patients, which is clinically meaningful in patients with limited treatment options whose tumors have otherwise progressed after surgery and radiation. A possible limitation is the lack of central radiology review of pretreatment scans 12–24 months before going on study. Pretreatment scans were evaluated by the local investigators using guidance provided in the protocol. For future trials of systemic therapies in meningiomas, systematically assessing pretreatment and posttreatment growth rates may provide further insight into the therapeutic impact of new therapies. Finally, future trials of systemic therapies for this patient population would benefit from more detailed collection of quality of life, neurocognitive outcome and AE data.

In conclusion, we demonstrate that genomically driven trials for patients with meningioma are feasible. Abemaciclib was well tolerated and showed encouraging activity in patients with recurrent or progressive high-grade meningioma with CDK pathway or *NF2* alterations and warrants further investigation. We are in the process of planning additional trials in this patient population.

## Methods

### Study oversight

This Alliance study was designed by the principal investigators and conducted in accordance with the provision of the Declaration of Helsinki (2013) and Good Clinical Practice guidelines. The National Cancer Institute Central Institutional Review Board approved the protocol. All patients provided signed informed consent. The protocol document is included in the Supplementary Information.

### Patients

Eligible patients had intracranial grade 2 or 3 meningioma as documented by central pathology review, and measurable disease (as defined by bidimensionally measurable enhancing lesions with a minimum diameter of 10 mm). Central genetic testing was conducted on a tumor sample from each patient for study arm determination. Presence of an alteration in the CDK pathway or in *NF2* in the tumor tissue was required for enrollment to the abemaciclib arm. Patients must have had progressive or residual disease as defined by residual measurable disease immediately after surgery, progressive measurable disease (increase in the size of the measurable lesion on imaging by 25% or more in 25 months) or progressive disease after radiation. Inclusion criteria also included steroid dosing stable for at least 4 days, no craniotomy for 28 days before or after registration, not pregnant and not nursing, ECOG performance status of 2 or less, no extracranial meningiomas, hemoglobin ≥8 g dl^−^^1^ recovered to Common Terminology Criteria for Adverse Events (CTCAE) grade 1 or less toxicity, except for residual alopecia, or grade 2 neuropathy and no chemotherapy, cancer-directed hormonal therapy or other investigational agents within 28 day of study treatment. Key exclusion criteria included active bacterial, fungal or detectable viral infection and a personal history of the following conditions: syncope of cardiovascular etiology, ventricular arrhythmia or pathological origin or sudden cardiac arrest.

### Study design, treatment and endpoints

Central pathology review was conducted on the diagnostic hematoxylin and eosin slides to confirm the diagnosis of meningioma. In addition, integral molecular testing to evaluate the presence of an eligible gene mutation was performed on a single formalin-fixed paraffin-embedded (FFPE) tissue block from the surgery that contained representative tumor tissue. For patients with specimens from multiple time points, it was recommended that the most recent specimen be submitted for genetic testing. For central genetic testing, there was a prespecified time limit of 21 days between central laboratory receipt of patient tumor specimen and notification of patient eligibility. Registration needed to occur within 28 days of receiving notification of patient eligibility from the central testing laboratory.

Abemaciclib was administered orally at 200 mg twice daily for 28-day cycles until disease progression, excessive toxicity, symptomatic neurological deterioration or study consent withdrawal. Guidelines for dose modifications were provided in the protocol. Patients underwent contrast-enhanced brain magnetic resonance imaging every 8 weeks using a consensus magnetic resonance imaging protocol^40^. Response was determined by local investigator review using standard Macdonald response criteria^41^. PFS6 was defined as the number of patients not having progressive disease or death within 6 months after the first day of treatment divided by the total number of evaluable patients. The RR was defined as the number of responses divided by the total number of evaluable patients. A patient was deemed to have a response if they had a confirmed partial or complete response.

### Statistical design

Alliance A071401 is a prospective, multi-arm, phase 2 study evaluating the efficacy of targeted therapies in cohorts of patients with specific genetic mutations (Supplementary Information). Each mutation-based cohort/arm was designed to be evaluated separately using a phase 2 study design. A tumor sample from each patient underwent central pathology review and genetic testing for arm determination. Grading was based on the 2016 edition of the WHO classification of brain tumors^42^. Patients with recurrent or progressive grade 2 or 3 meningiomas harboring CDK pathway or *NF2* mutations who met the eligibility criteria were enrolled in the abemaciclib arm; these results are reported in this article. The co-primary endpoints were PFS6 and RR after starting treatment. The trial was designed such that the treatment would be considered worthy of further study if either the PFS6 or RR endpoint was met. To accommodate the two co-primary endpoints, a Bonferroni correction^43^ was used to further constrain the one-sided type I error (bounded at 5% per endpoint). EAST v.6.3 and PASS 15.01 were used for the power calculations.

The first 24 patients who met the eligibility criteria, signed the consent form and began treatment were considered evaluable for the analysis of the primary endpoint decision rule. Twenty-four evaluable patients provided at least 85% power to detect a true PFS6 rate of at least 41.5%, with a significance level of 0.02 against the null hypothesis of 15% PFS6 rate. If at least eight patients (at least 31.1%) demonstrated PFS6 among the 24 evaluable patients, the agent would be considered worthy of further testing in this mutation-defined grade 2 or 3 cohort. The PFS6 hypothesis was derived from historical benchmark data from a comprehensive review of prior trials of medical therapies in patients with meningiomas, where PFS6 for grade 2 or 3 meningiomas ranged from 0 to 29% (ref. ^1^). Twenty-four evaluable patients provided at least 89% power to detect a true RR of at least 20%, with a significance level of 0.021 against the null hypothesis of 2.5% RR. If at least three responses (at least 12.5%) were observed among the 24 evaluable patients, the agent would be considered worthy of further testing in this mutation-defined treatment arm. Overaccrual was allowed to account for potential non-evaluable patients and to more fully assess secondary endpoints of interest. Secondary endpoints included overall survival and PFS, which were summarized with Kaplan–Meier curves and estimates^44^. AEs, graded according to CTCAE v.4.0, were summarized as the number, frequency and severity of each event, the number and frequency of patients who experienced any AE, any AEs graded 3 or greater, and any AEs graded 4 or greater.

Data collection and statistical analyses were conducted by the Alliance Statistics and Data Management Center. Medidata Rave Electronic Data Capture was used to collect the clinical trial data. SAS v.9.4 was used for the data analysis. Data quality was ensured by review of the data by the Alliance Statistics and Data Management Center and by the study chairperson according to Alliance policies. All analyses were based on the study database frozen on 6 November 2024. Of note, this is a small phase 2 study; as such, we were not powered to do any specific subset analyses based on sex.

This study is monitored by the study team on a monthly basis. Reports containing a summary of accrual and AEs according to treatment arm are reviewed.

### Targeted molecular profiling

Molecular profiling was conducted using anchored multiplex PCR technology for single-nucleotide variant, insertion or deletion (indel), and copy number detection in genomic DNA using the ArcherDx platform and Illumina NextSeq next-generation sequencing, as described previously^16,45–49^. Briefly, after histopathological review of tumor enrichment, genomic DNA was isolated from a FFPE tumor specimen, enzymatically sheared, end-repaired, adenylated and ligated with a half-functional adapter. Two hemi-nested reactions were used to generate a sequencing library targeting hotspots and full exons. Illumina NextSeq 2 × 150 bp paired-end sequencing data were aligned to the hg19 human genome reference. MuTect1 (ref. ^46^), LoFreq^50^, GATK^47–49^ and a laboratory-developed hotspot caller, were applied for single-nucleotide variant and indel variant detection. If one of the callers was positive, the variant call was flagged as positive. All such variants were manually reviewed by a molecular pathologist to ensure their accuracy. A copy number caller using a coverage distribution from a panel of normals was used to detect copy gain and loss. Variants were reported with Human Genome Variation Society protein and DNA nomenclature, followed by the referenced Ensembl transcript ID.

### FISH

1p loss was assessed using FISH with FFPE tumor specimens. Briefly, 5-μm sections of FFPE tumor material were prepared, and an hematoxylin and eosin section reviewed, to select regions for hybridization that contained a majority of tumor cells. A dual-color FISH assay was performed using the Vysis LSI 1p36/LSI 1q25 Dual Color Probe (Abbott Molecular). Signal quantitation in 50 tumor cells was used to generate a 1p-to-1q ratio, with a ratio of less than 0.75 diagnosed as loss, and 0.75 or greater as maintenance.

### IHC

IHC for protein biomarkers was performed using 5-μm FFPE sections on a Leica Bond III automated stainer (Leica Biosystems). Stains were scored by a board-certified pathologist (A.J.I.). The following antibodies were used: CDK4 clone DCS-35 (1:300 dilution, cat. no. sc-23896, Santa Cruz Biotechnology); cyclin D1 clone EP12 (prediluted, cat. no. PA0046, Leica Biosystems); Ki-67 clone MM1 (prediluted, cat. no. PA0118, Leica Biosystems); p16 clone E6H4 (1:4 dilution, cat. no. 725-4793, Roche Diagnostics).

### Statistical analysis of exploratory biomarkers

Patients were included in this analysis if they were eligible and had at least one posttreatment disease response assessment. Those with best response of stable diseases, partial response or complete response were classified as having CB, and those with best response of progressive disease were classified as no CB. Summary statistics were generated; Wilcoxon rank-sum and Fischer’s exact test *P* values were computed to test for differences in the distribution of continuous and categorical biomarkers across CB versus no CB groups. The association between biomarkers and CB was quantified using univariable logistic regression. The biomarkers were modeled as follows: Ki-67 as continuous; cyclin D1 as categorical (2 versus 3); p16 as continuous (0–3) and categorical (0, 1 versus 2, 3); CDK4 as continuous (0–3) and categorical (0, 1 versus 2, 3); 1p-to-1q FISH ratio as continuous (log_2_-transformed); 1p FISH mutation status as categorical (maintenance versus loss); and *CDKN2A* as categorical (normal versus mutation/deletion).

### Reporting summary

Further information on research design is available in the Nature Portfolio Reporting Summary linked to this article.

## Online content

Any methods, additional references, Nature Portfolio reporting summaries, source data, extended data, supplementary information, acknowledgements, peer review information; details of author contributions and competing interests; and statements of data and code availability are available at 10.1038/s41591-025-04141-4.

## Supplementary information

Supplementary Information Supplementary Tables 1–6, protocol document and summary of amendments to protocol A071401.

Reporting Summary

## Figures and Tables

**Extended Data Table 1:**
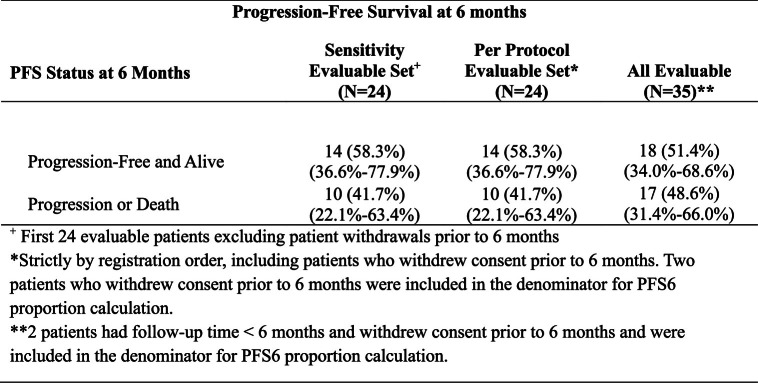
Progression-free survival at 6 months (PFS6) across all patients

**Extended Data Table 2:**
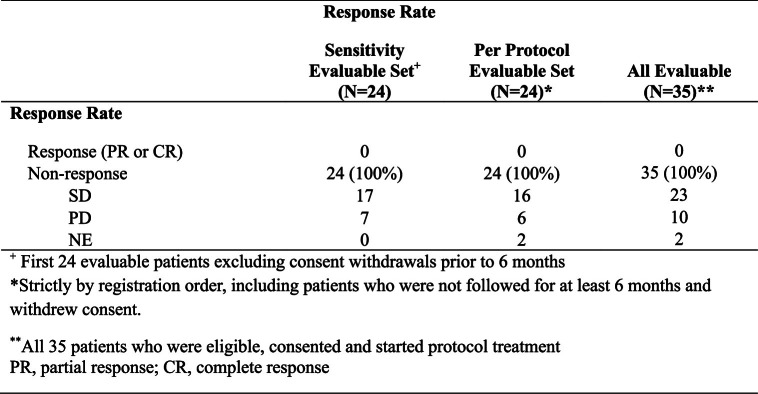
Response rate across all patients

**Extended Data Table 3:**
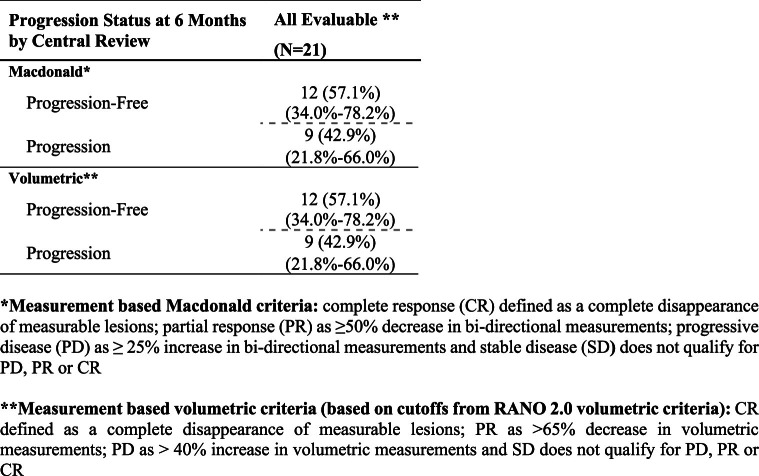
Progression-free rate at 6 months per measurement-based central imaging review among evaluable patients with central review available for pre- and post 6 months

**Extended Data Table 4:**
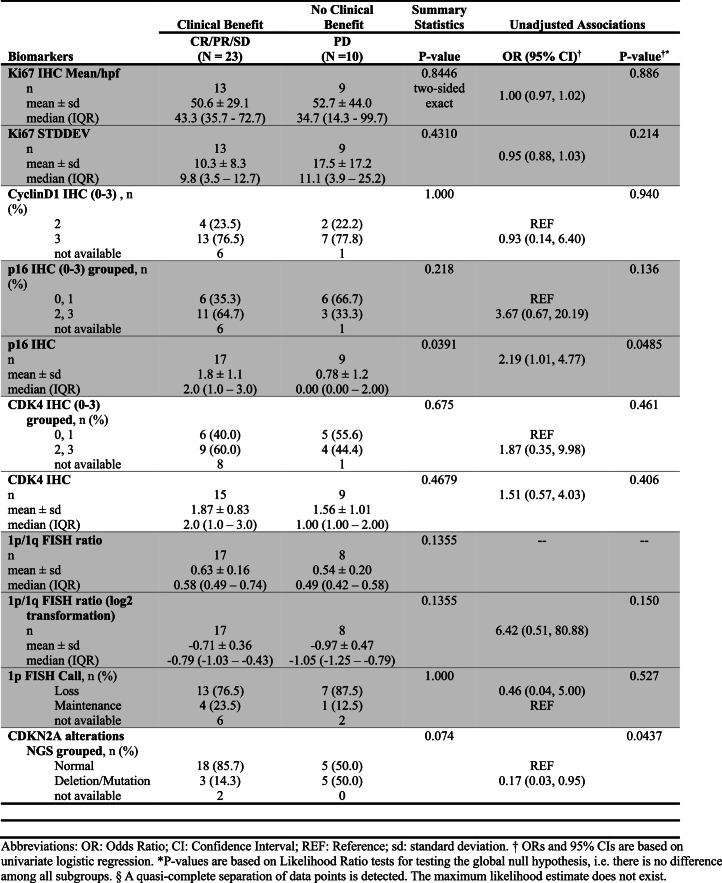
Association between clinical benefit and biomarkers among patients evaluable for primary endpoint

## Data Availability

De-identified patient data may be requested from the Alliance for Clinical Trials in Oncology via concepts@alliancenctn.org if data are not publicly available. Requests are acknowledged within 24 h of receipt and then sent for internal review. A formal review process includes verifying the availability of data, conducting a review of any existing agreements that may have implications for the project and ensuring that any transfer complies with the institutional review board. The investigator will be required to sign a data release form before transfer.
