# Supplementary material for: Abemaciclib in meningiomas with somatic NF2 or CDK pathway alterations: the phase 2 Alliance A071401 trial
Source: Nat Med. 2026 Jan 16;32(2):717–24. doi: 10.1038/s41591-025-04141-4 (PMC12920099; doi:10.1038/s41591-025-04141-4)
Supplement: Supplementary file 1 — Supplementary Tables 1–6, protocol document and summary of amendments to protocol A071401. [file 41591_2025_4141_MOESM1_ESM.pdf]

nature medicine

Supplementary information https://doi.org/10.1038/s41591-025-04141-4

Abemaciclib in meningiomas with somatic *NF2* or CDK pathway alterations: the phase 2 Alliance A071401 trial

In the format provided by the authors and unedited

Supplementary Table 1: Demographics in the Safety Population, Evaluable Full Set, Sensitivity Evaluable Set and Per Protocol Evaluable Set

	Safety Population (N=36)[a]	Evaluable Full Set (N=35)[b]	Sensitivity Evaluable Set (N=24)[c]	First 24 patients enrolled (N=24)[d]
**Age**				
Mean (SD)	62.9 (11.8)	62.9 (12.0)	62.2 (13.3)	61.9 (12.8)
Median	63.5	64.0	61.5	63.5
Q1, Q3	54.5, 73.5	54.0, 74.0	52.5, 74.0	52.5, 71.5
Range	(39.0-84.0)	(39.0-84.0)	(39.0-84.0)	(39.0-84.0)
**Gender**				
Female	22 (61.1%)	22 (62.9%)	14 (58.3%)	14 (58.3%)
Male	14 (38.9%)	13 (37.1%)	10 (41.7%)	10 (41.7%)
**Race**				
Asian	1 (2.8%)	1 (2.9%)	1 (4.2%)	1 (4.2%)
Black or African American	5 (13.9%)	5 (14.3%)	5 (20.8%)	5 (20.8%)
Native Hawaiian or Pacific Islander	1 (2.8%)	1 (2.9%)	0 (0.0%)	0 (0.0%)
Not reported: patient refused or not available	1 (2.8%)	1 (2.9%)	1 (4.2%)	1 (4.2%)
Unknown: Patient unsure	2 (5.6%)	2 (5.7%)	0 (0.0%)	0 (0.0%)
White	26 (72.2%)	25 (71.4%)	17 (70.8%)	17 (70.8%)
**Ethnicity**				
Hispanic or Latino	6 (16.7%)	6 (17.1%)	4 (16.7%)	2 (8.3%)
Not Hispanic or Latino	28 (77.8%)	27 (77.1%)	20 (83.3%)	22 (91.7%)
Unknown: Patient is unsure of	2 (5.6%)	2 (5.7%)	0 (0.0%)	0 (0.0%)
**ECOG Performance Status**				
0	10 (27.8%)	10 (28.6%)	6 (25.0%)	7 (29.2%)
1	22 (61.1%)	21 (60.0%)	15 (62.5%)	14 (58.3%)
2	4 (11.1%)	4 (11.4%)	3 (12.5%)	3 (12.5%)
**Central Review W.H.O. Grade**				
Grade 1	1 (2.8%)	0 (0.0%)	0 (0.0%)	0 (0.0%)
Grade 2	20 (55.6%)	20 (57.1%)	14 (58.3%)	15 (62.5%)
Grade 3	15 (41.7%)	15 (42.9%)	10 (41.7%)	9 (37.5%)
**Tumor Grade (on-study form)**				
G1	1 (2.8%)	0 (0.0%)	0 (0.0%)	0 (0.0%)
G2	22 (61.1%)	22 (62.9%)	14 (58.3%)	15 (62.5%)
G3	13 (36.1%)	13 (37.1%)	10 (41.7%)	9 (37.5%)
**Status of tumor at registration**				
Both	9 (25.0%)	9 (25.7%)	6 (25.0%)	7 (29.2%)
Progressive measurable disease	19 (52.8%)	18 (51.4%)	14 (58.3%)	14 (58.3%)

	Safety Population (N=36)[a]	Evaluable Full Set (N=35)[b]	Sensitivity Evaluable Set (N=24)[c]	First 24 patients enrolled (N=24)[d]
Residual measurable disease	8 (22.2%)	8 (22.9%)	4 (16.7%)	3 (12.5%)
**Multifocal disease**				
Yes	17 (47.2%)	17 (48.6%)	11 (45.8%)	12 (50.0%)
No	19 (52.8%)	18 (51.4%)	13 (54.2%)	12 (50.0%)
**Corticosteroid therapy at study entry**				
No	28 (77.8%)	27 (77.1%)	18 (75.0%)	19 (79.2%)
Yes	8 (22.2%)	8 (22.9%)	6 (25.0%)	5 (20.8%)
**Previous surgery related to this tumor**				
Yes	36 (100.0%)	35 (100.0%)	24 (100.0%)	24 (100.0%)
**Prior radiation therapy for this tumor**				
Yes	34 (94.4%)	33 (94.3%)	22 (91.7%)	23 (95.8%)
No	2 (5.6%)	2 (5.7%)	2 (8.3%)	1 (4.2%)
**Prior systemic (cancer) therapy for this tumor**				
Yes	13 (36.1%)	13 (37.1%)	9 (37.5%)	9 (37.5%)
No	23 (63.9%)	22 (62.9%)	15 (62.5%)	15 (62.5%)
**Prior systemic (cancer) therapy for this tumor - details**				
Abemaciclib; Everolimus; Octreotide; VT3989	1 (2.8%)	1 (2.9%)	1 (4.2%)	1 (4.2%)
Bevacizumab	3 (8.3%)	3 (8.6%)	2 (8.3%)	2 (8.3%)
Bevacizumab; Nivolumab + Bevacizumab	1 (2.8%)	1 (2.9%)	1 (4.2%)	1 (4.2%)
Bevacizumab; Octreotide	2 (5.6%)	2 (5.7%)	1 (4.2%)	1 (4.2%)
Capivasertib	1 (2.8%)	1 (2.9%)	1 (4.2%)	0
Everolimus; Octreotide	1 (2.8%)	1 (2.9%)	1 (4.2%)	1 (4.2%)
Ipilumumab; Mvasi; Nivolumab	1 (2.8%)	1 (2.9%)	0	1 (4.2%)
AZD2014	1 (2.8%)	1 (2.9%)	1 (4.2%)	1 (4.2%)
Nivolumab	1 (2.8%)	1 (2.9%)	1 (4.2%)	1 (4.2%)
Octreotide; Pembrolizumab	1 (2.8%)	1 (2.9%)	0	0
**Any prior cancer diagnosis**				
Yes	5 (13.9%)	5 (14.3%)	3 (12.5%)	3 (12.5%)
No	31 (86.1%)	30 (85.7%)	21 (87.5%)	21 (87.5%)
**Any prior cancer diagnosis - details**				
ALL (39 yrs prior to reg): WBRT and systemic chemotherapy	1 (2.8%)	1 (2.9%)	1 (4.2%)	1 (4.2%)
Breast cancer (8 yrs prior to reg): double mastectomy	1 (2.8%)	1 (2.9%)	1 (4.2%)	1 (4.2%)
Hodgkin Lymphoma (29 yrs prior to reg): ABVD, MOPP, and Mantle RT	1 (2.8%)	1 (2.9%)	0	0

	Safety Population (N=36)[a]	Evaluable Full Set (N=35)[b]	Sensitivity Evaluable Set (N=24)[c]	First 24 patients enrolled (N=24)[d]
Skin Cancer (52 yrs prior to reg): surgery	1 (2.8%)	1 (2.9%)	0	1 (4.2%)
Skin Cancer (6 yrs prior to reg): Local excision	1 (2.8%)	1 (2.9%)	1 (4.2%)	0
**Number of prior treatment modalities**				
1	2 (5.6%)	2 (5.7%)	2 (8.3%)	1 (4.2%)
2	21 (58.3%)	20 (57.1%)	13 (54.2%)	14 (58.3%)
3	13 (36.1%)	13 (37.1%)	9 (37.5%)	9 (37.5%)

[a]All patients who received any quantity of study drug

[b]All 35 patients who are eligible, consented and started protocol treatment

[c]Excluding patient withdrawals prior to 6 months

[d]First 24 evaluable patients including patient withdrawals prior to 6 months

Supplementary Table 2: Treatment summary

Treatment Summary - Abemaciclib<br>All patients who started protocol treatment (N=36)	
**Number of Treatment Cycles Received**	
Mean (SD)	8.9 (9.9)
Median (IQR)	4.5 (2.0, 13.0)
Range	(1.0-35.0)
**% of Planned Dose Received (all cycles)**	
Mean (SD)	86.6 (20.1)
Median (IQR)	95.3 (81.3, 99.6)
Range	(21.0-100.0)
**Dose Modifications in at least one cycle**	
No	20 (55.6%)
Yes	16 (44.4%)
**N of Cycles with Dose Modifications (per patient)**	
N	16
Mean (SD)	1.4 (0.6)
Median (IQR)	1.0 (1.0, 2.0)
Range	(1.0-3.0)
**Reasons for Dose Modifications**	
Gastrointestinal disorders	8
Unplanned: gastrointestinal disorders	5
Metabolism and nutrition disorders	2
Unplanned: blood and lymphatic system disorders	1
Unplanned: general disorders and administration site conditions	1
Unplanned: investigations	1
Unplanned: respiratory, thoracic and mediastinal disorders	1
Hepatobiliary disorders	1
Investigations	1
Other, not per protocol	1
Respiratory, thoracic and mediastinal disorders	1
**Treatment omission in at least one cycle**	
No	12 (33.3%)
Yes	24 (66.7%)
**N of Cycles with Treatment omission (per patient)**	
N	24
Mean (SD)	3.5 (3.3)
Median (IQR)	2.0 (1.5, 4.5)
Range	(1.0-14.0)
**Reasons for Dose Omissions**	
Other, not per protocol	53
Gastrointestinal disorders	14
General disorders and administration site conditions	4
Immune system disorders	3
Eye disorders	2

Treatment Summary - Abemaciclib<br>All patients who started protocol treatment (N=36)	
Infections and infestations	2
Nervous system disorders	2
Investigations	1
Respiratory, thoracic and mediastinal disorders	1
Vascular disorders	1
**Treatment delay in at least one cycle**	
No	24 (66.7%)
Yes	12 (33.3%)
**N of Cycles with Treatment delay (per patient)**	
N	12
Mean (SD)	1.3 (0.7)
Median (IQR)	1.0 (1.0, 1.5)
Range	(1.0-3.0)
**Reasons for Dose Delays**	
Gastrointestinal disorders	3
General disorders and administration site conditions	2
Hepatobiliary disorders	2
Investigations	2
Other, not per protocol	2
Blood and lymphatic system disorders	1
Infections and infestations	1
Missing	1
Respiratory, thoracic and mediastinal disorders	1
Social circumstances	1

Supplementary Table 3: Listing of all grade 1 or greater adverse events regardless of attribution

Listing of Grade 1+ Adverse Events<br>Maximum Grade per Patient Per Event<br>Regardless of Attribution<br>Number of Evaluable Patients: 36					
	Grade of Adverse Event				
	1-Mild	2-Mod	3-Severe	4-LifeThr	5-Lethal
	N (%)	N (%)	N (%)	N (%)	N (%)
**Hematologic Adverse Events**					
**Blood/Bone Marrow**					
Anemia	6 (17%)	3 (8%)	2 (6%)	0 (0%)	0 (0%)
Blood and lymph sys disorders - Oth Spec	0 (0%)	1 (3%)	0 (0%)	0 (0%)	0 (0%)
Lymphocyte count decreased	2 (6%)	1 (3%)	2 (6%)	0 (0%)	0 (0%)
Neutrophil count decreased	5 (14%)	4 (11%)	2 (6%)	0 (0%)	0 (0%)
Platelet count decreased	3 (8%)	1 (3%)	0 (0%)	0 (0%)	0 (0%)
White blood cell decreased	4 (11%)	4 (11%)	1 (3%)	0 (0%)	0 (0%)
**Non-Hematologic Adverse Events**					
**Cardiac disorders**					
Chest pain - cardiac	1 (3%)	0 (0%)	0 (0%)	0 (0%)	0 (0%)
**Eye disorders**					
Blurred vision	0 (0%)	0 (0%)	1 (3%)	0 (0%)	0 (0%)
Eye disorders - Other, specify	1 (3%)	0 (0%)	0 (0%)	0 (0%)	0 (0%)
Floaters	1 (3%)	0 (0%)	0 (0%)	0 (0%)	0 (0%)
**Gastrointestinal disorders**					
Constipation	4 (11%)	0 (0%)	0 (0%)	0 (0%)	0 (0%)
Diarrhea	14 (39%)	12 (33%)	3 (8%)	0 (0%)	0 (0%)
Dry mouth	0 (0%)	1 (3%)	0 (0%)	0 (0%)	0 (0%)
Dyspepsia	8 (22%)	0 (0%)	0 (0%)	0 (0%)	0 (0%)
Gastrointestinal disorders - Oth spec	1 (3%)	0 (0%)	0 (0%)	0 (0%)	0 (0%)
Intra-abdominal hemorrhage	0 (0%)	0 (0%)	0 (0%)	1 (3%)	0 (0%)
Mucositis oral	0 (0%)	1 (3%)	0 (0%)	0 (0%)	0 (0%)
Nausea	11 (31%)	6 (17%)	0 (0%)	0 (0%)	0 (0%)
Stomach pain	1 (3%)	0 (0%)	0 (0%)	0 (0%)	0 (0%)
Toothache	1 (3%)	0 (0%)	0 (0%)	0 (0%)	0 (0%)
Vomiting	8 (22%)	3 (8%)	1 (3%)	1 (3%)	0 (0%)
**Gen disord and admin site cond**					
Edema limbs	0 (0%)	1 (3%)	1 (3%)	0 (0%)	0 (0%)
Facial pain	1 (3%)	0 (0%)	0 (0%)	0 (0%)	0 (0%)
Fatigue	20 (56%)	6 (17%)	2 (6%)	0 (0%)	0 (0%)

Listing of Grade 1+ Adverse Events
Maximum Grade per Patient Per Event
Regardless of Attribution
Number of Evaluable Patients: 36

	Grade of Adverse Event				
	**1-Mild N (%)**	**2-Mod N (%)**	**3-Severe N (%)**	**4-LifeThr N (%)**	**5-Lethal N (%)**
Fever	0 (0%)	1 (3%)	0 (0%)	0 (0%)	0 (0%)
Gait disturbance	0 (0%)	1 (3%)	1 (3%)	0 (0%)	0 (0%)
Gen disord and admin site conds-Oth spec	0 (0%)	0 (0%)	1 (3%)	0 (0%)	1 (3%)
Pain	1 (3%)	0 (0%)	0 (0%)	0 (0%)	0 (0%)
**Infections and infestations**					
Enterocolitis infectious	0 (0%)	1 (3%)	0 (0%)	0 (0%)	0 (0%)
Lung infection	0 (0%)	0 (0%)	2 (6%)	0 (0%)	0 (0%)
**Inj, pois and proced complic**					
Fall	2 (6%)	0 (0%)	1 (3%)	0 (0%)	0 (0%)
Stomal ulcer	1 (3%)	0 (0%)	0 (0%)	0 (0%)	0 (0%)
Wound complication	1 (3%)	0 (0%)	0 (0%)	0 (0%)	0 (0%)
**Investigations**					
Alanine aminotransferase increased	2 (6%)	0 (0%)	2 (6%)	1 (3%)	0 (0%)
Alkaline phosphatase increased	0 (0%)	1 (3%)	0 (0%)	0 (0%)	0 (0%)
Aspartate aminotransferase increased	1 (3%)	1 (3%)	1 (3%)	1 (3%)	0 (0%)
Blood bilirubin increased	0 (0%)	1 (3%)	0 (0%)	0 (0%)	0 (0%)
Creatinine increased	1 (3%)	7 (19%)	0 (0%)	0 (0%)	0 (0%)
Investigations - Other, specify	2 (6%)	0 (0%)	0 (0%)	0 (0%)	0 (0%)
Weight gain	1 (3%)	0 (0%)	0 (0%)	0 (0%)	0 (0%)
Weight loss	8 (22%)	2 (6%)	1 (3%)	0 (0%)	0 (0%)
**Metabol and nutrition disord**					
Anorexia	1 (3%)	6 (17%)	0 (0%)	0 (0%)	0 (0%)
Dehydration	1 (3%)	1 (3%)	1 (3%)	0 (0%)	0 (0%)
Hyperglycemia	0 (0%)	1 (3%)	1 (3%)	0 (0%)	0 (0%)
Hyperkalemia	0 (0%)	0 (0%)	1 (3%)	0 (0%)	0 (0%)
Hypernatremia	1 (3%)	0 (0%)	0 (0%)	0 (0%)	0 (0%)
Hypertriglyceridemia	1 (3%)	0 (0%)	0 (0%)	0 (0%)	0 (0%)
Hypoalbuminemia	2 (6%)	0 (0%)	1 (3%)	0 (0%)	0 (0%)
Hypocalcemia	1 (3%)	0 (0%)	1 (3%)	0 (0%)	0 (0%)
Hypokalemia	3 (8%)	1 (3%)	0 (0%)	0 (0%)	0 (0%)
Hyponatremia	6 (17%)	0 (0%)	1 (3%)	0 (0%)	0 (0%)
Hypophosphatemia	0 (0%)	1 (3%)	1 (3%)	0 (0%)	0 (0%)
Metabolism, nutrition disord - Oth spec	1 (3%)	0 (0%)	0 (0%)	0 (0%)	0 (0%)
**Musculosk and conn tiss disord**					

Listing of Grade 1+ Adverse Events
Maximum Grade per Patient Per Event
Regardless of Attribution
Number of Evaluable Patients: 36

	Grade of Adverse Event				
	**1-Mild N (%)**	**2-Mod N (%)**	**3-Severe N (%)**	**4-LifeThr N (%)**	**5-Lethal N (%)**
Arthralgia	6 (17%)	0 (0%)	1 (3%)	0 (0%)	0 (0%)
Generalized muscle weakness	1 (3%)	2 (6%)	1 (3%)	0 (0%)	0 (0%)
Muscle weakness lower limb	0 (0%)	0 (0%)	1 (3%)	0 (0%)	0 (0%)
Muscle weakness upper limb	0 (0%)	1 (3%)	0 (0%)	0 (0%)	0 (0%)
**Nervous system disorders**					
Ataxia	0 (0%)	1 (3%)	0 (0%)	0 (0%)	0 (0%)
Cognitive disturbance	0 (0%)	1 (3%)	0 (0%)	0 (0%)	0 (0%)
Depressed level of consciousness	0 (0%)	0 (0%)	1 (3%)	0 (0%)	0 (0%)
Dizziness	1 (3%)	0 (0%)	1 (3%)	0 (0%)	0 (0%)
Dysesthesia	1 (3%)	0 (0%)	0 (0%)	0 (0%)	0 (0%)
Dysgeusia	2 (6%)	0 (0%)	0 (0%)	0 (0%)	0 (0%)
Edema cerebral	0 (0%)	0 (0%)	0 (0%)	2 (6%)	0 (0%)
Headache	9 (25%)	7 (19%)	1 (3%)	0 (0%)	0 (0%)
Intracranial hemorrhage	0 (0%)	1 (3%)	0 (0%)	0 (0%)	0 (0%)
Memory impairment	0 (0%)	1 (3%)	0 (0%)	0 (0%)	0 (0%)
Muscle weakness left-sided	0 (0%)	1 (3%)	0 (0%)	0 (0%)	0 (0%)
Seizure	0 (0%)	2 (6%)	4 (11%)	0 (0%)	0 (0%)
Stroke	1 (3%)	0 (0%)	0 (0%)	0 (0%)	0 (0%)
**Psychiatric disorders**					
Agitation	0 (0%)	1 (3%)	0 (0%)	1 (3%)	0 (0%)
Anxiety	1 (3%)	0 (0%)	0 (0%)	0 (0%)	0 (0%)
Confusion	1 (3%)	0 (0%)	1 (3%)	0 (0%)	0 (0%)
Insomnia	1 (3%)	0 (0%)	0 (0%)	0 (0%)	0 (0%)
Irritability	1 (3%)	0 (0%)	0 (0%)	0 (0%)	0 (0%)
**Renal and urinary disorders**					
Acute kidney injury	0 (0%)	0 (0%)	1 (3%)	0 (0%)	0 (0%)
Proteinuria	1 (3%)	0 (0%)	0 (0%)	0 (0%)	0 (0%)
Renal and urinary disorders - Oth spec	0 (0%)	1 (3%)	0 (0%)	0 (0%)	0 (0%)
Renal calculi	0 (0%)	0 (0%)	1 (3%)	0 (0%)	0 (0%)
Urinary retention	0 (0%)	1 (3%)	0 (0%)	0 (0%)	0 (0%)
Urinary tract obstruction	0 (0%)	0 (0%)	1 (3%)	0 (0%)	0 (0%)
**Respirat, thor, mediast disord**					
Cough	1 (3%)	1 (3%)	0 (0%)	0 (0%)	0 (0%)
Dyspnea	1 (3%)	0 (0%)	0 (0%)	0 (0%)	0 (0%)

Listing of Grade 1+ Adverse Events
Maximum Grade per Patient Per Event
Regardless of Attribution
Number of Evaluable Patients: 36

	Grade of Adverse Event				
	**1-Mild N (%)**	**2-Mod N (%)**	**3-Severe N (%)**	**4-LifeThr N (%)**	**5-Lethal N (%)**
Hiccups	0 (0%)	1 (3%)	0 (0%)	0 (0%)	0 (0%)
Hypoxia	0 (0%)	1 (3%)	0 (0%)	0 (0%)	0 (0%)
Pneumonitis	0 (0%)	1 (3%)	0 (0%)	0 (0%)	0 (0%)
Postnasal drip	1 (3%)	0 (0%)	0 (0%)	0 (0%)	0 (0%)
Resp, thoracic, mediastinal - Oth spec	0 (0%)	0 (0%)	0 (0%)	1 (3%)	0 (0%)
Respiratory failure	0 (0%)	0 (0%)	0 (0%)	2 (6%)	0 (0%)
Sore throat	1 (3%)	0 (0%)	0 (0%)	0 (0%)	0 (0%)
Stridor	0 (0%)	0 (0%)	1 (3%)	0 (0%)	0 (0%)
Wheezing	1 (3%)	0 (0%)	0 (0%)	0 (0%)	0 (0%)
**Skin and subcutan tiss disord**					
Dry skin	8 (22%)	1 (3%)	0 (0%)	0 (0%)	0 (0%)
Pruritus	4 (11%)	3 (8%)	0 (0%)	0 (0%)	0 (0%)
Rash maculo-papular	6 (17%)	2 (6%)	0 (0%)	0 (0%)	0 (0%)
Skin hyperpigmentation	1 (3%)	0 (0%)	0 (0%)	0 (0%)	0 (0%)
**Vascular disorders**					
Hypertension	0 (0%)	1 (3%)	2 (6%)	0 (0%)	0 (0%)
Hypotension	0 (0%)	0 (0%)	1 (3%)	0 (0%)	0 (0%)
Thromboembolic event	0 (0%)	1 (3%)	2 (6%)	0 (0%)	0 (0%)

Supplementary Table 4: Listing of all commonly-occuring (≥10%) grade 1 or greater adverse events regardless of attribution

	Safety Population (N=36)
**Grade 1+ Commonly Occurring AEs (≥10%), any attribution**	
Diarrhea	29 (80.6%)
Fatigue	28 (77.8%)
Headache	17 (47.2%)
Nausea	17 (47.2%)
Vomiting	13 (36.1%)
Anemia	11 (30.6%)
Neutrophil count decreased	11 (30.6%)
Weight loss	11 (30.6%)
Dry skin	9 (25.0%)
White blood cell decreased	9 (25.0%)
Creatinine increased	8 (22.2%)
Dyspepsia	8 (22.2%)
Rash maculo-papular	8 (22.2%)
Anorexia	7 (19.4%)
Arthralgia	7 (19.4%)
Hyponatremia	7 (19.4%)
Pruritus	7 (19.4%)
Seizure	6 (16.7%)
Alanine aminotransferase increased	5 (13.9%)
Lymphocyte count decreased	5 (13.9%)
Aspartate aminotransferase increased	4 (11.1%)
Constipation	4 (11.1%)
Hypokalemia	4 (11.1%)
Platelet count decreased	4 (11.1%)
Generalized muscle weakness	4 (11.1%)

Supplementary Table 5: Cause of death for patients on study

	All patients enrolled (N=39)	Safety Population (N=36)	Per Protocol Evaluable Set (N=24)
**Vital Status**			
Alive	26	23	14
Dead	13	13	10
**Cause of Death**			
Tumor	9	9	7
Progressive disease	1	1	1
Other: Possible aspiration event during hospitalization	1	1	1
Unknown	2	2	1

Supplementary Table 6: Hospital-associated adverse events

Patient no.	Adverse event(s) leading to hospitalization
1	muscle weakness lower limb (grade 3 unlikely); agitation (grade 4 unlikely); dizziness (grade 3 unlikely and grade 3 possible)
2	thromboembolic event (grade 2 not related)
3	vomiting (grade 4 definite); diarrhea (grade 3 definite); seizure (grade 3 not related)
4	hyperkalemia (grade 3 possible); acute kidney injury (grade 3 not related)
5	seizure (grade 3 not related), confusion (grade 3 unlikely), edema cerebral (grade 4 not related), hyperglycemia (grade 3 unlikely), thromboembolic event (grade 3 possible), dehydration (grade 2 possible), intra-abdominal hemorrhage (grade 4 not related) and intracranial hemorrhage (grade 2 not related)
6	alanine aminotransferase increase (grade 4 definite); aspartate aminotransferase increase (grade 4 definite); alkaline phosphatase increase (grade 2 definite)
7	headache (grade 3 not related), General disorders and administration site conditions - Other specify disease progression (grade 5 not related)
8	seizure (grade 3 not related), edema cerebral (grade 4 not related), muscle weakness left-sided (grade 3 not related), fall (grade 2 not related)

Clinical Protocol Document

ALLIANCE FOR CLINICAL TRIALS IN ONCOLOGY

PROTOCOL UPDATE TO ALLIANCE A071401

PHASE II TRIAL OF SMO/AKT/NF2 INHIBITORS IN PROGRESSIVE MENINGIOMAS WITH SMO/AKT/NF2/CDKx/CCNx MUTATIONS

X	**Update:**	X	**Status Change:**
X	Eligibility changes		Activation
X	Therapy / Dose Modifications / Study Calendar changes		Closure
X	Informed Consent changes		Suspension / temporary closure
X	Scientific / Statistical Considerations changes	X	Reactivation
	Data Submission / Forms changes		
X	Editorial / Administrative changes		
	Other:		

If your site utilizes the CIRB as your IRB of record
No recommended IRB level of review is provided by the Alliance since the CIRB is the IRB of record for this trial. The site has 30 days after the posting of this amendment to implement it at their site. Please refer to the amendment application and CIRB guidelines for further instructions.

If your site utilizes a local IRB as your IRB of record
Expedited IRB approval is allowed. The proposed changes in this amendment are minor and do not affect the overall risk/benefit ratio. IRB approval (or disapproval) is required within 90 days. Please follow your local IRB guidelines.

UPDATES TO THE PROTOCOL

Cover Page
- The title of the study has been updated to include CDKx and CCNx mutations.
- The phrase *"Abemaciclib (NSC #783671)"* has been added to the list of *"Industry-supplied agents."*

Cancer Trials Support Unit (CTSU) Address and Contact Information
All text included in the table has been revised with updated CTSU language.

Schema

- The vismodegib box has been grayed out to indicate that the arm is closed. Additionally, the date accrual to the vismodegib arm was closed has been added below the drug name.
- Two new boxes have been added to include the new mutational group and treatment regimen added with this update. They read:"NF2, CDKN2A, CDK4, CDK6, CCND1, CCND2, CCND3, or CCNE1 (As of Update #09)" and "Abemaciclib 200 mg PO BID."

Table of Contents

The table of contents has been updated.

Section 1.2 (Genetic analysis of meningioma)

New seventh and eighth sentences have been added to the first paragraph, which read: "Furthermore, high grade and progressive meningiomas have loss of CDKN2A, which is part of the CDK pathway and many of these CDKN2A alterations co-occur in NF2 mutated meningiomas. NF2 loss and CDKN2A loss have been demonstrated to promote meningioma progression in preclinical models." Four new references (#18-21) have been added as a result of this new language, and all subsequent references have been renumbered throughout the text and in the references list in Section 16.0.

Section 1.3 (Available Agents that Act on SMO, PTCH1, AKT1, and NF-2 Mutated Tumors)

A new fifth paragraph has been added to provide information regarding abemaciclib. The new paragraph begins "Abemaciclib is a selective and potent small molecule CDK4 and CDK6 dual…"

Section 1.10 (Abemaciclib)

A new section has been added to provide background information on abemaciclib. Subsequent sections have been renumbered accordingly.

Section 1.11 (Clinical Experience with Abemaciclib)

A new section has been added to describe the clinical experience with abemaciclib. Subsequent sections have been renumbered accordingly.

Section 1.14 (Impact of the Trial)

The underlined language has been added to the first sentence: "Based on the biomarker work, we have designed a phase 2 study of vismodegib (SMO inhibitor), AZD5363 (AKT inhibitor), GSK2256098 (a FAK inhibitor), or abemaciclib (CDK inhibitor) in patients with recurrent or progressive meningiomas harboring genetic alterations in SMO/PTCH1, AKT1/PIK3CA/PTEN, NF2, or the CDK pathway (CDK4/CDK6/CCND1/CCND2/CCND3/CCNE1/CDKN2A), respectively."

Section 2.1 (Primary Objectives)

A new primary objective for the CDK inhibitor arm has been added as Section 2.1.4.

Section 3.1 (On-study guidelines)

The reproductive considerations for abemaciclib have been added as a new fourth subsection under the "In addition:" heading.

Section 3.3.1 (Documentation of Disease)

In the third bullet of the third criterion titled "**Post radiation patients**," the underlined language has been added to the second sentence "If the progressive meningioma lesion has been radiated, at least 24…"

Section 3.3.3 (Prior Treatment)

The fifth bullet has been updated to state that “For patients treated with external beam radiation, interstitial brachytherapy or radiosurgery, an interval > 4 weeks must have elapsed from completion of radiation treatment to registration.” Previously, this was an interval greater than 2 weeks.

Section 3.3.8 (Concomitant medications [Only regarding NF2/CDKN2A/CDK4/CDK6/CCND1/CCND2/CCND3/CCNE1/AKT1/PIK3CA/PTEN genetic alterations])
A new third bullet has been added to address concomitant use of CYP3A inducers and inhibitors for patients enrolled to the abemaciclib arm.

Section 3.3.10 (Required Initial Laboratory Values)
The third footnote (***) has been revised to indicate that triplicate EKG is required if the patient is assigned to the AZD5363 arm. Patients on all other arms require a single EKG. Previously, a single EKG was required for all arms.

Section 3.3.11 (Comorbid Conditions)
A new section has been added to state that patients cannot enroll if they have uncontrolled medical comorbidities.

Section 3.4 (Additional Registration Eligibility Criteria for Abemaciclib Arm)
A new section has been added to define additional eligibility criteria for patients who are assigned to the abemaciclib arm.

Section 4.0 (Patient Registration)
This entire section has been revised with updated CTSU language.

Section 4.7 (Treatment Assignments and Patient Cohort)

- The word “abemaciclib” has been added to the list of agents in the second sentence of the first paragraph.
- A new seventh bullet has been added under the third paragraph that reads: “Group 7: NF2 mutation/CDKN2A copy number loss/CDK4, CDK6, CCND1, CCND2, CCND3, or CCNE1 copy number gain – abemaciclib.”

Section 5.0 (Study Calendar)
The entire study calendar has been updated to include the abemaciclib arm. Specifically, the following changes have been made:

- In Footnote 6, the phrase “and ≤ 7 days prior to initiation of abemaciclib for patients with *NF2* mutation” has been added to the second sentence to indicate that patients need to take a pregnancy test within 7 days prior to beginning treatment on abemaciclib.
- Footnote 9 has been added to provide direction for patients on the abemaciclib arm. Footnote 9 has been added under “Day 1 of each cycle (cycle is 28 days)” for “Complete blood count, differential, platelets” and under “Day 1 of each cycle (cycle is 28 days)” for “Chemistry (Creatinine, AST, ALT, Alk. Phos., Bili, glucose) HbA1c (if required).”
- Footnote B has been revised to provide direction for patients on the AZD5363 and abemaciclib arm. Footnote B has been added under “Post Treatment Follow Up” for “EKG.”
- Footnote E has been added to indicate that these items on the study calendar are only required of patients enrolled in the abemaciclib arm. Footnote E has been added under “Post treatment follow up” for “Complete Blood Count, Differential, Platelets” and “Chemistry (Creatinine, AST, ALT, Alk. Phos., Bili, glucose) HbA1c (if required).”

Section 6.1 (Data Collection and Submission)

- This entire section has been revised with updated CTSU language.
- A new Section 6.1.2, regarding the Data Quality Portal, has been added.

Section 6.3 (CT and MR Imaging Data Submission)

The language under **"1) TRIAD based data transfer"** has been revised with updated CTSU language.

Section 7.0 (Treatment Plan/Intervention)

A new fourth bullet has been added below the third paragraph entitled "Treatment will be administered as follows." The new bullet reads: "In patients harboring alterations in the CDK pathway or in NF2 (after Update #9), a CDK inhibitor (abemaciclib) will be administered."

Section 7.3 (Arc C [AKT1/PIK3CA/PTEN mutation – AZD6363])

The frequency of the dosing in the table has been corrected from "…repeated every cycle" to "…repeated weekly."

Section 7.4 (Arm D [CDK4, CDK6, CDKN2A, CCND1, CCND2, CCND3, CCNE1 alterations-Abemaciclib])

This section has been added to provide instructions for patients enrolled to the abemaciclib arm. Subsequent sections have been renumbered.

Section 7.5 (Important interaction information for patients receiving GSK2256098, AZD5363, and abemaciclib)

- The title of this section has been revised to add abemaciclib.
- Section 7.5.1
  - Abemaciclib has been added to the list of agents in the first and second sentence.
  - A new third paragraph has been added to provide direction if co-administration of abemaciclib with a strong CYP3A inhibitor is unavoidable.
- Section 7.5.2
  - Abemaciclib has been added to the list of agents in the first and second sentence.

Section 8.1.5 (Diarrhea)

Information regarding diarrhea management for patients enrolled to the abemaciclib arm has been added as the third paragraph.

Section 8.1.14 (Reproductive Considerations)

Directions on pregnancy prevention for patients enrolled to the abemaciclib arm has been added as the fourth sentence of the paragraph.

Section 8.1.15 (Liver Toxicity)

- The section has been retitled to Liver Toxicity, previously Hepatitis.
- A final sentence has been added that reads: "For abemaciclib specific hepatic toxicity, please see Section 8.5.5."
- Two new third and fourth paragraphs and a table regarding hepatic monitoring tests has been added to direct sites on how to monitor patients with hepatic abnormalities.

Section 8.4.1 (Dose Levels)

The dose levels have been modified so that the first dose reduction will be 320mg and the second dose reduction will be 200mg.

Section 8.5 (Abemaciclib Dose Modifications)
This entire section has been added to provide information about abemaciclib dose modifications.

Section 9.3.1 (Phase I and Early Phase 2 Studies)

In the fifth bullet under the section titled "**Expedited Adverse Event Reporting for Suspected Exposure to Agent that May Cause Serious or Life-threatening Birth Defects for Patients Receiving Vismodegib,"** AZD5363 has been replaced with GDC-0449.

Section 9.5 (Comprehensive Adverse Events and Potential Risks list [CAEPR] for AZD5363 [NSC 782347])

The CAEPR for AZD5363 has been updated.

Section 10.3 (AZD5363 [NSC # 782347, IND # 126926])

- Under *Formulation*, the AZD5363 supplied dose has been updated to 160 (previously 80) or 200mg tablets. Both doses will be available in 60 (previously 30) tablet or 76 (previously 60) tablet bottles.
- Directions for obtaining the investigator brochure for AZD5363 has been added to the end of the section.

Section 10.4 (Abemaciclib [LY2835219, VERENZIO™, NSC#783671])
This entire section has been added to provide drug information regarding abemaciclib.

Section 13.1 (Study Design)

- In the first sentence of the first paragraph, "Grade I-III" has been added after "…PTEN-mutated meningioma."
- The following text was deleted from the first sentence of the first paragraph: "in regard to two co-primary endpoints."
- The former second ("The co-primary endpoints are response rate (RR) and progression-free survival at 6 months [PFS6] after starting treatment."), fourth ("Patients with recurrent or progressive Grade I-III meningiomas will be eligible for this trial."), and fifth ("Samples will undergo central pathology review. Patient's tumor samples will undergo mutational testing.") sentence have been removed.
- In the new third sentence, "confirmed by central molecular review" has been added after "…PTEN mutations."
- In the fourth sentence, for clarity, the words "up to" have been added after "there will be."
- A few fifth sentence has been added, which reads: "Below is a table to summarize the analysis and primary or co-primary endpoints per arm."
- The former second paragraph regarding how the study is powered for the RR endpoint and PFS6 endpoint has been removed.
- A table has been added to summarize the analysis cohorts and primary or co-primary endpoints per arm.
- The new second paragraph (former third paragraph) below the table now reads: "There are no planned interim analyses. The sample size calculations were done with EAST v6.3 and PASS 15.01."

Section 13.2 (Statistical Design and Analysis for the Primary Endpoint)

- The first sentence of Section 13.2.1 now reads: "The primary end points used in this study are progression-free survival at 6 months (PFS6) and response rate (RR)."

- The statistical design for the abemaciclib arm has been added to Section 13.2.2 as paragraphs 10-12.
- In Section 13.2.2, under Overall power and family-wise alpha consideration, the over-all type I error bound of is 19%, formerly 15%.
- The analysis plan for abemaciclib has been added to Section 13.2.3 as the fifth paragraph.

Section 13.3.1 (Sample Size)

- The first sentence of the second paragraph has been updated to reflect that there will be a total of 108 evaluable patients (previously 84) and 24 patients in the NF2-abemaciclib arm.
- The underlined phrase has been added to the second sentence of the third paragraph: "We anticipate accruing an additional 3 patients in NF2 mutation – GSK2256098 grade II/III cohort and NF2, CDKx, CCNx mutations– abemaciclib grade II/III cohort to account for ineligibilities or cancellations."

Section 16.0 (References)

References 18-21 have been added, found in the text under Section 1.2.

Appendix IV (Patient Medication Diaries)

- A medication diary for abemaciclib has been added as Appendix IV-D. The cover page of this appendix has been updated accordingly.

Appendix VII (Central Laboratory Genotype Testing Procedures)

Information regarding the abemaciclib arm has been added as the first row in the table under **Target Mutations.**

Appendix VIII (Patient Drug Information Handouts and Wallet Cards)

A drug information handout and wallet card for abemaciclib has been added as Appendix VIII-C. The cover page of this appendix has been updated accordingly.

UPDATES TO THE MODEL CONSENT

Why is this study being done?

- Language explaining abemaciclib has been added as the fifth paragraph
- In the last paragraph, the total number of people taking place in the study has been updated to 108, previously 84.

What are the study groups?

- A sentence has been added to indicate that the vismodegib arm has been closed to new patient participation
- A new fifth paragraph regarding patients with a genetic change in the CDK pathway receiving abemaciclib has been added.
- "CDK – abemaciclib" has been added to the list of medications in the first sentence of the seventh paragraph.
- Abemaciclib has been added to the schema table.

What extra tests and procedures will I have if I take part in this study?

Under "Before you begin the study:" clarification has been added to the third bullet point, which states that patients who are unable to undergo a brain MRI can have a brain CT done.

What possible risks can I expect from taking part in this study?

The risk list for abemaciclib has been added.

<u>What are the costs of taking part in this study?</u>
Abemaciclib has been added to the list of drugs in the first sentence of the first paragraph.

A replacement protocol document and model consent form have been issued

---

ATTACH TO THE FRONT OF EVERY COPY OF THIS PROTOCOL

ALLIANCE FOR CLINICAL TRIALS IN ONCOLOGY

ALLIANCE A071401

PHASE II TRIAL OF SMO/AKT/NF2 INHIBITORS IN PROGRESSIVE MENINGIOMAS WITH SMO/AKT/NF2/CDKx/CCNx MUTATIONS

Industry-supplied agents:
Vismodegib (NSC # 747691), GSK2256098 (NSC #783781), AZD5363 (NSC #782347), Abemaciclib (NSC #783671)
IND #126926; IND holder: Alliance

ClinicalTrials.gov Identifier: NCT 02523014

Study Chair
Priscilla K. Brastianos, MD
Massachusetts General Hospital
55 Fruit Street, Yawkey 9E
Boston, MA 02215
Tel: 617-643-1938 Fax: 617-643-2591
pbrastianos@partners.org

Study Co-Chair & Neuro-Oncology Committee Chair
Evanthia Galanis, MD
Mayo Clinic
200 First Street Southwest
Rochester, Minnesota 55905
Tel: 507-284-1370 Fax: 507- 284-1902
galanis.evanthia@mayo.edu

Community Oncology Co-chair
Suriya Jeyapalan, MD
Tufts University
Tel:617-667-9898
sjeyapalan@tuftsmedicalcenter.org

Neuropathology Co-Chair
Caterina Giannini, MD, PhD
Mayo Clinic
Tel: 507-538-1181
giannini.caterina@mayo.edu

Neuropathology Co-Chair & Correlative Co-Chair
Sandro Santagata, MD, PhD
Brigham and Women’s Hospital
Tel: 617-525-5686
ssantagata@partners.org

Correlative Co-Chair
John Iafrate, MD, PhD
Massachusetts General
Tel: 617-726-0166
aiafrate@partners.org

Correlative Committee Chair
Jann N. Sarkaria, MD
Mayo Clinic
Tel: 507-284-8227
sarkaria.jann@mayo.edu

Imaging Co-chair
Elizabeth Gerstner, MD
Massachusetts General Hospital
Tel: 617-643-1938
egerstner@partners.org

Radiation Oncology Co-Chair
Paul Brown, MD
Mayo Clinic
Tel: 507-284-3551
Brown.paul@mayo.edu

Neurosurgical Co-Chairs
Ian Dunn, MD
Brigham and Women’s Hospital
Tel: 617-732-5633
idunn@partners.org

Neurosurgical Co-Chairs
Fred Barker, MD
Massachusetts General Hospital
Tel: 617-724-8772
barker@helix.mgh.harvard.edu

Primary Statistician
Karla Ballman Ph.D.
Tel: 646-962-8023
kab2053@med.cornell.edu

Secondary Statistician
Erin Twohy, MS
Tel: 507-293-2485
twohy.erin@mayo.edu

Protocol Coordinator
Sakuni Taniya Silva
Tel: 773-834-4091 Fax: 312-345-0117
stsilva@uchicago.edu

Data Manager
Meagan Odegaard
Tel: 507-284-4124 Fax: 507-284-1902
odegaard.meagan@mayo.edu

Participating Groups: ALLIANCE / Alliance for Clinical Trials in Oncology (lead), ECOG-ACRIN / ECOG-ACRIN Cancer Research Group, NRG / NRG Oncology and SWOG / SWOG

Study Resources:

Expedited Adverse Event Reporting<br>http://eapps-ctep.nci.nih.gov/ctepaers/	Medidata Rave® iMedidata portal<br>https://login.imedidata.com
**OPEN (Oncology Patient Enrollment Network)**<br>https://open.ctsu.org	**Biospecimen Management System**<br>http://bioms.allianceforclinicaltrialsinoncology.org

Protocol Contacts:

A071401 Nursing Contact
Wanda L. DeKrey, RN, OCN
Altru Cancer Center
Tel: 701-780-6520
wdekrey@altru.org

A071401 Pharmacy Contact
Heidi D. Finnes, PharmD, BCOP
Mayo Clinic
Tel: 507-538-7066
finnes.heidi@mayo.edu

Alliance Biorepository at Mayo Clinic
Paraffin-embedded tissue:
Helen Tollefson
Pathology Coordinator
Tel: 507-266-0724 Fax: 507-266-7240
Tollefson.Helen@mayo.edu

Non-paraffin biospecimens:
Roxann Neumann, RN, BSN, CCRP
Biospecimens Accessioning and Processing (BAP)
Tel: 507-538-0602
neumann.roxann@mayo.edu

Alliance Imaging contact
IROC Ohio
The Ohio State University
Wright Center of Innovation
395 W. 12th Ave., RM #414
Columbus, OH 43240
Tel: 614-293-9151 Fax: 614-293-9275
Alliance071401@irocohio.org

Protocol-related questions may be directed as follows:

Questions	Contact (via email)
Questions regarding patient eligibility, treatment, and dose modification:	Study Chair, Nursing Contact, Protocol Coordinator, and (where applicable) Data Manager
Questions related to data submission, RAVE or patient follow-up:	Data Manager
Questions regarding the protocol document and model informed consent:	Protocol Coordinator
Questions related to IRB review	Alliance Regulatory Inbox regulatory@allianceNCTN.org
Questions regarding CTEP-AERS reporting:	Alliance Pharmacovigilance Inbox pharmacovigilance@alliancenctn.org
Questions regarding status of central pathology review and biomarker testing:	Sandro Santagata, MD, PhD & John Iafrate, MD, PhD

CANCER TRIALS SUPPORT UNIT (CTSU) ADDRESS AND CONTACT INFORMATION

<table>
<tr><th>For regulatory requirements:</th><th>For patient enrollments:</th><th>For study data submission:</th></tr>
<tr><td>Regulatory documentation must be submitted to the CTSU via the Regulatory Submission Portal.
(Sign in at www.ctsu.org, and select the Regulatory > Regulatory Submission.)

Institutions with patients waiting that are unable to use the Portal should alert the CTSU Regulatory Office immediately at 1-866-651-2878 to receive further instruction and support.

Contact the CTSU Regulatory Help Desk at 1-866-651-2878 for regulatory assistance.</td><td>Refer to the patient enrollment section of the protocol for instructions on using the Oncology Patient Enrollment Network (OPEN). OPEN can be accessed at https://www.ctsu.org/OPEN_SYSTEM/ or https://OPEN.ctsu.org.

Contact the CTSU Help Desk with any OPEN-related questions at ctsucontact@westat.com.</td><td>Data collection for this study will be done exclusively through Medidata Rave. Refer to the data submission section of the protocol for further instructions.</td></tr>
<tr><td colspan="3">The most current version of the study protocol and all supporting documents must be downloaded from the protocol-specific page located on the CTSU members' website (https://www.ctsu.org). Access to the CTSU members' website is managed through the Cancer Therapy and Evaluation Program - Identity and Access Management (CTEP-IAM) registration system and requires log on with CTEP-IAM username and password.</td></tr>
<tr><td colspan="3"><u>For clinical questions (i.e., patient eligibility or treatment-related)</u> see the Protocol Contacts, Page 2.</td></tr>
<tr><td colspan="3"><u>For non-clinical questions (i.e., or questions unrelated to patient eligibility, treatment, or clinical data submission</u> contact the CTSU Help Desk by phone or e-mail:
CTSU General Information Line – 1-888-823-5923, or ctsucontact@westat.com. All calls and correspondence will be triaged to the appropriate CTSU representative.</td></tr>
<tr><td colspan="3">The CTSU website is located at https://www.ctsu.org.</td></tr>
</table>

Phase II Trial Of SMO/AKT/NF2 Inhibitors In Progressive Meningiomas With SMO/AKT/ NF2 Mutations

Pre-Registration Eligibility Criteria (see Section 3.2)
Local diagnosis of meningioma and have tissue available
for central path review and integral biomarker testing.

Registration Eligibility Criteria (See Section 3.3)
- Presence of specific SMO/PTCH1, NF2 or AKT1/PIK3CA./PTEN mutations (see Section 3.3.1)
- Progressive or residual disease as defined in Section 3.3.1
- Measurable disease as defined by a bi-dimensionally measurable
- main lesion on MRI or CT images (MRI preferred) (See Section 3.3.2)
- No chemotherapy, other investigational agents within 28 days of study treatment.
- No other concurrent investigational agents or other meningioma-directed therapy (chemotherapy, radiation)
- > 24 weeks must have elapsed from completion of radiation treatment (XRT, brachytherapy, radiosurgery) to registration
- Steroid dosing stable for at least 4 days
- Recovered to CTCAE grade 1 or less toxicity
- No craniotomy 28 days prior to or after registration
- Not pregnant and not nursing
- ECOG Performance Status ≤ 2
- Stable for lesions for 6 months for patients with history of NF. See Section 3.3.7.
- No metastatic meningiomas (as defined by extracranial meningiomas).
- No history of allergic reactions attributed to compounds of similar biologic composition to assigned study drug
- For abemaciclib arm: Hemoglobin ≥ 8 g/dL
- For abemaciclib arm: Patients who received chemotherapy must have recovered (Common Terminology Criteria for Adverse Events [CTCAE] Grade ≤1) from the acute effects of chemotherapy except for residual alopecia or Grade 2 peripheral neuropathy prior to randomization. A washout period of at least 21 days is required between last chemotherapy dose and randomization (provided the patient did not receive radiotherapy). Patients who received adjuvant radiotherapy must have completed and fully recovered from the acute effects of radiotherapy. A washout period of at least 14 days is required between end of radiotherapy and randomization.
- For abemaciclib arm: A female of childbearing potential, must have a negative serum pregnancy test within 7 days of the first dose of abemaciclib and agree to use a highly effective contraception method during the treatment period and for 3 weeks following the last dose of abemaciclib. Please see Section 3.4.3.
- For abemaciclib arm: Patients with active bacterial infection (requiring intravenous [IV] antibiotics at time of initiating study treatment), fungal infection, or detectable viral infection (such as known human immunodeficiency virus positivity or with known active hepatitis B or C [for example, hepatitis B surface antigen positive] are excluded. Screening is not required for enrollment.
- Patients with personal history of any of the following conditions: syncope of cardiovascular etiology, ventricular arrhythmia of pathological origin (including, but not limited to, ventricular tachycardia and ventricular fibrillation), or sudden cardiac arrest, are excluded.

Required Initial Laboratory Values
Require initial laboratory values as indicated in Section 3.3.11.

Schema

1 Cycle = 28 Days

Note: Pregnancy prevention must start 4 weeks prior to study drug.

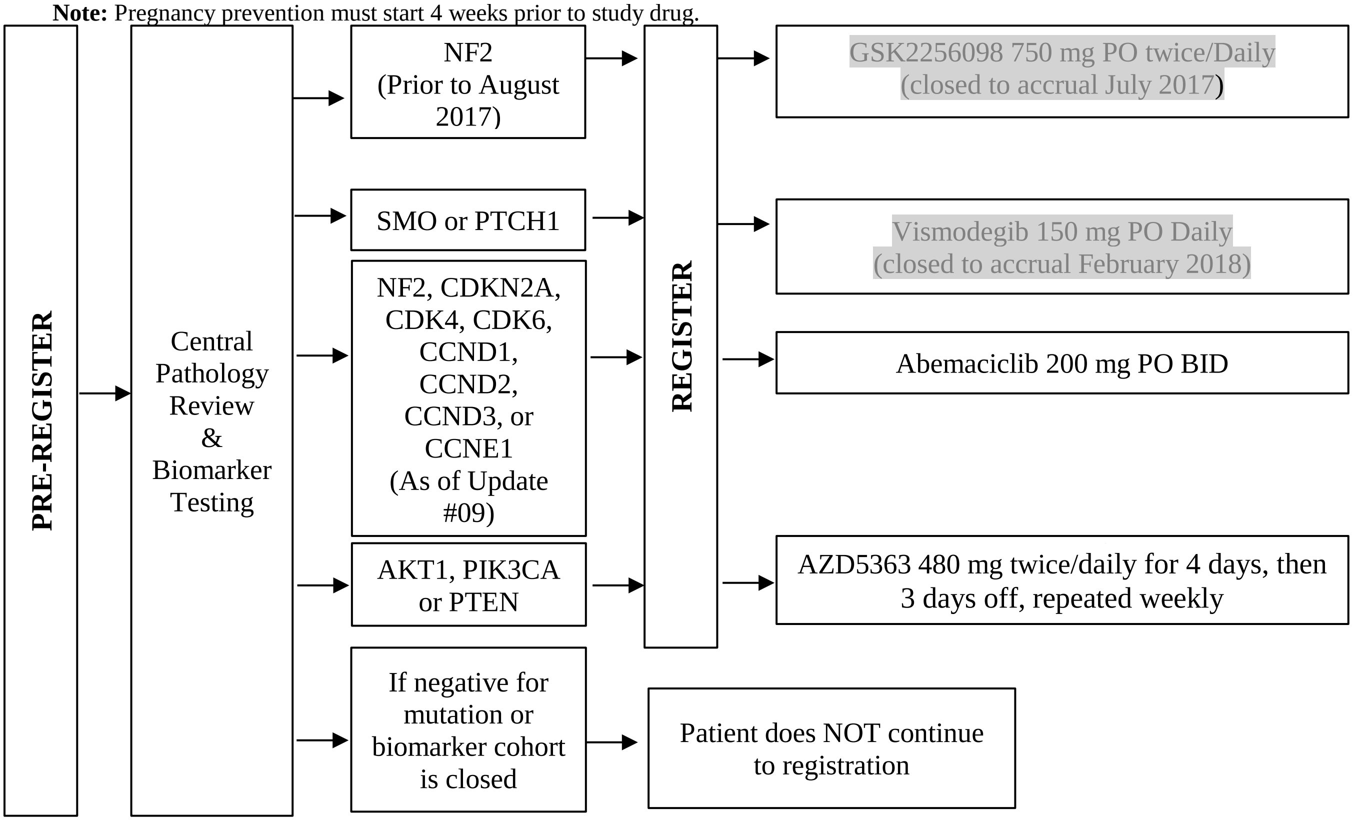

Treatment is to continue until disease progression or unacceptable adverse event. Patients discontinuing treatment for reasons other than progressive disease, will continue following the Study Calendar for disease assessments until progressive disease is documented, for a maximum of 2 years. Patients will be followed for survival up to a maximum of 5 years from registration.

Please refer to the full protocol text for a complete description of the eligibility criteria and treatment plan.

Table of Contents

SECTION .... **PAGE**

1.0 BACKGROUND **9**
1.1 The natural history of meningioma 9
1.2 Genetic analysis of meningioma 9
1.3 Available Agents that Act on SMO, PTCH1, AKT1, and NF-2 Mutated Tumors 10
1.4 Vismodegib (GDC-0449) 11
1.5 Clinical Experience with Vismodegib 12
1.6 AZD5363 16
1.7 Clinical Experience with AZD5363 as Monotherapy 17
1.8 GSK2256098 19
1.9 Clinical Experience with GSK2256098 20
1.10 Abemaciclib 21
1.11 Clinical Experience with Abemaciclib 22
1.12 Registration Quality of Life (QOL) Measurements 25
1.13 Central Radiology Review 25
1.14 Impact of the Trial 25

2.0 OBJECTIVES **25**
2.1 Primary objectives 25
2.2 Secondary objectives 26
2.3 Correlative science objectives 26

3.0 PATIENT SELECTION **27**
3.1 On-study guidelines 27
3.2 Pre-Registration Eligibility Criteria 29
3.3 Registration Eligibility Criteria 30
3.4 Additional Registration Eligibility Criteria for Abemaciclib Arm 33

4.0 PATIENT REGISTRATION **34**
4.1 CTEP Registration Procedures 34
4.2 CTSU Site Registration Procedures 35
4.3 Patient Pre-Registration Requirements 37
4.4 Patient Registration Procedures 38
4.5 Patient Registration/Randomization Procedure 38
4.6 Registration to Correlative and Companion Studies 39
4.7 Treatment Assignments and Patient Cohorts 39

5.0 STUDY CALENDAR **41**

6.0 DATA AND SPECIMEN SUBMISSION **44**
6.1 Data Collection and Submission 44
6.2 Specimen collection and submission 45
6.3 CT and MR Imaging Data Submission 52

7.0 TREATMENT PLAN/INTERVENTION **55**
7.1 Arm A (SMO/PTCH1 mutation - Vismodegib) 55
7.2 Arm B (NF2 mutation – GSK2256098) 55
7.3 Arm C (AKT1/PIK3CA/PTEN mutation – AZD6363) 56
7.4 Arm D (CDK4, CDK6, CDKN2A, CCND1, CCND2, CCND3, CCNE1 alterations-Abemaciclib) 56
7.5 Important interaction information for patients receiving GSK2256098, AZD5363, and abemaciclib 56

8.0 DOSE AND TREATMENT MODIFICATIONS 58
8.1 Ancillary therapy, concomitant medications, and supportive care 58
8.2 Vismodegib Dose Modifications 62
8.3 GSK2256098 dose modifications 63
8.4 AZD5363 dose modifications 65
8.5 Abemaciclib Dose Modifications 67

9.0 ADVERSE EVENTS 69
9.1 Routine adverse event reporting 69
9.2 CTCAE Routine Reporting Requirements 70
9.3 Expedited Adverse Event Reporting (CTEP-AERS) 71
9.4 Comprehensive Adverse Events and Potential Risks list (CAEPR) for GDC-0449 (Vismodegib, NSC 747691) 74
9.5 Comprehensive Adverse Events and Potential Risks list (CAEPR) for AZD5363 (NSC 782347) 76

10.0 DRUG INFORMATION 78
10.1 Vismodegib (GDC-0449, Erivedge®, NSC# 747691, IND#126926) IND holder: Alliance 78
10.2 GSK2256098 (NSC# 783781, IND #126926) 79
10.3 AZD5363 (NSC # 782347, IND #126926) 81
10.4 Abemaciclib (LY2835219, VERENZIO™, NSC#783671) 82

11.0 MEASUREMENT OF EFFECT 86
11.1 Schedule of Evaluations: 86
11.2 Definitions of Measurable and Non-Measurable Disease 86
11.3 Guidelines for Evaluation of Measurable Disease 86
11.4 Measurement of Treatment/Intervention Effect 87
11.5 Definitions of analysis variables 89

12.0 END OF TREATMENT/INTERVENTION 90
12.1 Duration of Treatment 90
12.2 Definitions and Follow-up Requirements 90
12.3 Extraordinary Medical Circumstances 90

13.0 STATISTICAL CONSIDERATIONS 91
13.1 Study Design 91
13.2 Statistical Design and Analysis for the Primary Endpoint 92
13.3 Sample size, accrual time and study duration 95
13.4 Supplementary Analysis plans 96
13.5 Monitoring the Study 96
13.6 Study Reporting 97
13.7 Descriptive Factors 97
13.8 Inclusion of Women and Minorities 97

14.0 CORRELATIVE AND COMPANION STUDIES 98
14.1 Correlative Studies using Biospecimens (Alliance A071401-ST1) 98
14.2 Imaging Biomarkers of Response (A071401-IM1) 102

15.0 GENERAL REGULATORY CONSIDERATIONS AND CREDENTIALING 103

16.0 REFERENCES 104

APPENDIX I: REGISTRATION FATIGUE/UNISCALE ASSESSMENTS 107

APPENDIX II: REQUIRED CONSENSUS MRI ACQUISITION PARAMETERS 108

APPENDIX III: 1.5T & 3T ADVANCED MRI PROTOCOL FOR SITES ACQUIRING DCE IMAGING 112

APPENDIX IV: PATIENT MEDICATION DIARIES 117
Appendix IV-A: Patient medication diary - Vismodegib 118
Appendix IV-B: Patient medication diary - GSK2256098 120
Appendix IV-C: Patient medication diary – AZD5363 122
Appendix IV-D: Patient medication diary – Abemaciclib 124

APPENDIX V: CONCOMITANT MEDICATIONS PROHIBITED OR FOR USE WITH CAUTION WITH AZD5363 126

APPENDIX VI: POSSIBLE PRENATAL EXPOSURE TO TERATOGEN REPORT 131

APPENDIX VII: CENTRAL LABORATORY GENOTYPE TESTING PROCEDURES 132

APPENDIX VIII: PATIENT DRUG INFORMATION HANDOUTS AND WALLET CARDS ... 135
Appendix VIII-A: For patients receiving GSK2256098 136
Appendix VIII-B: For patients receiving AZD5363 138
Appendix VIII-C: For patients receiving Abemaciclib 140

APPENDIX IX: ALGORITHMS FOR MANAGEMENT OF TOXICITIES WITH AZD5363 143

1.0 BACKGROUND

1.1 The natural history of meningioma

Meningiomas are the most common primary brain tumor, with a prevalence of 170,000 cases in the US and an annual incidence of 18,000 new cases. Most meningiomas are of the typical (Grade I) variety. However, depending on their location within the nervous system, grade I meningiomas can cause significant morbidity or mortality. Even after surgical resection, recurrence rates can be as high as 20%[1-3], and patients with Grade I tumors have reduced long-term survival.

Approximately 20% of meningiomas are atypical (Grade II) and anaplastic (Grade III), defined by increased mitoses, necrosis, higher nuclear to cytoplasmic ratios, or histologic appearance resembling carcinoma, sarcoma, or melanoma[4]. Recurrence rates for Grade II and III meningiomas are 40% and 80%, respectively[3]. The prognosis of atypical and anaplastic meningiomas is poor, with 5-year overall survival rates between 47-65%[5,6].

Treatment options, particularly for the atypical or anaplastic meningiomas, are limited[5]. Radiation is frequently used as an adjunct to surgery; however, there are no effective chemotherapeutic options when surgery and radiation fail to offer durable long-term disease control[7]. Traditional cytotoxic agents have minimal activity in this setting[8,9], and targeted agents in unselected patients[10] have demonstrated modest benefit at best. Response rate has been 0% in nearly all studies of systemic therapy in recurrent tumors of all grades[9,11-13]. A poor understanding of what drives meningioma development has hampered the development of therapeutic agents to supplement surgery and radiation. Therefore these patients have limited therapeutic options and effective treatments are greatly needed.

Agent	Result
Irinotecan[8]	0% response rate, 5 month TTP
Hydroxyurea[9]	0% response rate, 2 month median PFS
Imatinib + Hydroxyurea[10]	0% response rate, 7month median PFS
Interferon[12]	0% response, 7month TTP
Gefitinib[13]	0% response, 16 week median PFS
Erlotinib[13]	0% response, 9 week median PFS

1.2 Genetic analysis of meningioma

The tumor suppressor *NF2* is disrupted in approximately half of meningiomas. A subset of meningiomas lacking *NF2* alterations harbor recurrent oncogenic mutations in *AKT1,* a member of the PI3K/AKT/mTOR pathway, and *SMO,* a key component of the hedgehog pathway[14,15]. Specifically, 8-13% of meningiomas have recurrent oncogenic mutations in *AKT1* (E17K)[14,15] and 15% exhibit immunohistochemical evidence of PI3K/AKT/mTOR pathway activation[14]. Five percent of meningiomas harbor mutations in *SMO* (W535L and L412F)[14,15] and 10% exhibit evidence of Hedgehog pathway activation (Figure 1)[14]. Additionally, 7% of NF2-wildtype meningiomas harbor oncogenic alterations in PIK3CA[16]. Notably, AKT1, SMO and PIK3CA pathway mutations occur in meningiomas of the skull base, which are historically the most difficult to treat surgically[15,17]. Furthermore, high grade and progressive meningiomas have loss of CDKN2A, which is part of the CDK pathway and many of these CDKN2A alterations co-occur in NF2 mutated meningiomas[18,19,20,21]. NF2 loss and CDKN2A loss have been demonstrated to promote meningioma progression in preclinical models[20].

Based on this data, there are several potential biomarkers that have now been identified in meningioma. As this is a disease which lacks effective therapies, the potential for biomarker driven therapy is of great interest.

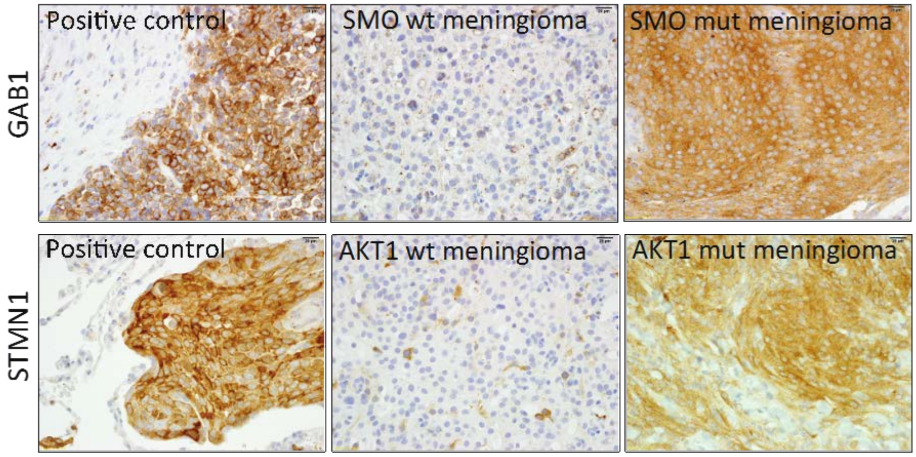

Figure 1: Immunohistochemistry indicates activation of the Hedgehog (GAB1) and AKT-mTOR (STMN1) pathways in meningiomas harboring *SMO* and *AKT1* mutations, respectively. (Brastianos et al. Nature Genetics 2013)

1.3 Available Agents that Act on SMO, PTCH1, AKT1, and NF-2 Mutated Tumors

Therapies that target these mutations are currently in clinical use in other cancers. SMO and PTCH1 mutations lead to aberrant activation of the Hedgehog (Hh) pathway. Evidence suggests that antagonism of excessive Hh signaling may provide a route to unique mechanism-based anticancer therapies[22]. Vismodegib, a small-molecule inhibitor of SMO, is associated with tumor responses in patients with basal-cell carcinoma, the majority of which have genetic alterations in the hedgehog signaling pathway. In a Phase II trial of vismodegib in locally advanced or metastatic basal cell carcinoma, response rates ranged from 30-43%[23]. Vismodegib is well-tolerated. Common adverse events of any grade of vismodegib included muscle spasms (68%), alopecia (63%), dysgeusia (51%), weight loss (46%), fatigue (36%), nausea (29%), decrease in appetite (23%) and diarrhea (22%). Serious adverse events were reported in 25% of patients.

AKT is a serine/threonine protein kinase with 3 isoforms (AKT1, AKT2, and AKT3) that participate in multiple pathways regulating several cellular processes, including survival, proliferation, tissue invasion, and metabolism. The importance of AKT-mediated pathways in tumor proliferation, survival, and resistance to chemotherapy and targeted agents, make AKT kinases promising targets for therapeutic intervention. AZD5363 is a novel, potent, selective inhibitor of the kinase activity of AKT (also known as protein kinase B). AZD5363 acts on cancers by blocking signaling through the AKT cellular survival pathway, leading to inhibition of cell proliferation and increased apoptosis. The most commonly reported AEs, regardless of dose, schedule or causality are: diarrhea, decreased appetite, hyperglycaemia, fatigue, maculo-papular rash, vomiting and nausea. In the AKT1 mutant expansion of a Phase I study of AZD5363, the most common grade >3 adverse events were hyperglycemia (24%), diarrhea (17%) and rash (15.5%)[24]

FAK is a nonreceptor protein tyrosine kinase that integrates signals from integrins and growth factor receptors; it regulates proliferation, survival, migration, invasion, and cancer stem cell renewal[25]. It is overexpressed in many cancers, including anaplastic meningiomas[26]. Low merlin product (the protein product of NF2) predicts sensitivity to FAK inhibition, likely because of the disrupted balance between cell-extracellular matrix and cell-cell interactions[27, 28]. Given the

predominance of NF2 inactivating events in meningiomas[14], we chose to evaluate FAK inhibition as a potential therapy for patients with *NF2*-mutated tumors.

GSK2256098 is a potent small molecule inhibitor of focal adhesion kinase (FAK) that is in clinical development for the treatment of cancer. As of August 2014, there was 1 completed Phase I study in healthy volunteers and two ongoing Phase I studies in subjects with solid tumors. In a Phase I single arm study in subjects with advanced, refractory solid tumors, subjects received continuous daily oral doses of GSK2256098 at doses ranging from 80 mg to 1500 mg BID. Overall, in 62 patients, the most frequently reported AEs associated with continuous oral BID dosing of GSK2256098 were nausea (76%), diarrhea (65%), vomiting (58%), decreased appetite (47%), proteinuria (26%), fatigue (24%), asthenia (23%), hyperbilirubinaemia (23%), constipation (21%) and hypercholesterolemia (21%). In the 55 patients that underwent at least one post-dose imaging assessment, stable disease (SD) was achieved by 28 subjects 14 mesothelioma subjects, 4 subjects with ovarian cancer and, 2 subjects with colorectal cancer and 1 subject with each of the following cancers: bile duct, kidney, melanoma, nasopharynx, non-small cell lung cancer, pancreas, renal cell, and thyroid. (GSK Investigator's Brochure).

Abemaciclib is a selective and potent small molecule CDK4 and CDK6 dual inhibitor with abroad antitumor activity pre-clinically and clinically. Importantly, abemaciclib has good CNS penetration and potentially provides a unique opportunity to treat primary brain tumors which have CDK pathway alterations, such as meningiomas.

1.4 Vismodegib (GDC-0449)

The hedgehog (Hh) signaling pathway is a crucial mediator of embryogenesis[29]. Signaling is initiated by the binding of the secreted morphogen, Hh, to its receptor, patched 1 (Ptch1). In the unbound state, Ptch1 inhibits Smoothened (SMO), a G-protein coupled phosphoprotein receptor, by preventing its localization to the cell surface; however, in the presence of the Hh ligand, the Hh-Ptch1 complex is internalized and the repression of Ptch1 on SMO is relieved. Surface localization of SMO is thought to initiate a signaling cascade, leading to the activation of the glioma-associated (*Gli*) family of zinc finger transcription factors, many of which are involved in proliferation, survival, and angiogenesis.

Aberrant activation of the Hh pathway in cancers is caused by mutations in the pathway or through Hh overexpression, termed either ligand-independent or ligand-dependent, respectively[30,31]. Past studies have identified mutations in the Hh receptor components, Ptch1 or SMO in basal cell carcinoma (BCC), medulloblastoma, and meningiomas resulting in pathway activation[14,32,33]. Excessive or inappropriate expression of the Hh ligand has been found in a significant proportion of patients with sporadic cancers of the gastrointestinal tract, pancreas, lung and prostate, suggesting that disruption of Hh signal transduction could potentially be beneficial in a broad array of tumor types[34,35]. Evidence suggests that antagonism of excessive Hh signaling may provide a route to unique mechanism-based anticancer therapies, blocking tumor growth and stimulating tumor regression without toxic effects on normal adjacent tissue[22].

Vismodegib is a small-molecule antagonist of the Hh signal pathway. Specifically, vismodegib binds to and inhibits SMO, blocking Hh signal transduction. *In vitro* and *in vivo* preclinical studies have demonstrated inhibition of Hh signaling following vismodegib administration. Vismodegib has demonstrated efficacy against a variety of primary human tumor xenografts, including colorectal cancer (CRC) and pancreatic adenocarcinoma, and tumor cell-line xenograft models. Inhibition of Hh signaling in xenograft models has been correlated with a decrease in tumor growth.

Vismodegib has been studied in several clinical trials including a phase 1 clinical trial in patients with advanced solid malignancies[36], which demonstrated that vismodegib is well-tolerated with no dose-limiting toxicities (DLTs) observed at any of the doses tested (150, 270, and 540 mg of

vismodegib). A Phase II study in basal cell carcinoma demonstrated response rates of 30-43% in locally advanced and metastatic basal cell carcinoma, with a well-tolerated toxicity profile[23].

1.5 Clinical Experience with Vismodegib

SHH3925g

A Phase I, company-sponsored clinical trial (SHH3925g) assessed the safety and pharmacokinetics (PK) of vismodegib and responses of 68 patients with solid tumors[37]. Thirty-three of the 68 patients had metastatic or locally advanced BCC. They received oral vismodegib at one of three doses: 17 patients received 150 mg per day, 15 patients received 270 mg per day, and 1 patient received 540 mg per day (median duration of treatment was 9.8 months).

Pharmacokinetic and pharmacodynamics studies with concentration-time profiles at 150 mg, 270 mg, or 540 mg showed that patients achieved Cmax by Day 2, with little decline in concentrations over the ensuing 6-day washout period. Also there was similar steady-state levels of vismodegib across all dosing cohorts, indicating non-linearity in PK with regard to dose. The PK study demonstrated high-affinity, reversible binding to AAG and binding to albumin, in addition to solubility-limited absorption and slow metabolic elimination properties.

EKG studies were performed and showed no apparent relationship between plasma vismodegib concentrations and prolongation of the QT interval.

The rate of grade 4 events for the trial was 9%. Of the 33 patients with basal cell carcinoma, 18 had an objective response to vismodegib, 2 had complete responses (CRs) and 16 had partial responses (PRs). The other 15 patients had either stable disease (11 patients) or progressive disease (4 patients). A patient with medulloblastoma also responded.

SHH4318g

Study SHH4318g was an open-label study planned for a single refractory medulloblastoma pediatric patient. The cerebrospinal fluid (CSF) concentration of vismodegib reached a maximum concentration on Day 14 with a value of 14.9 ng/mL. The estimated unbound concentration of vismodegib in CSF was similar to the unbound concentration measured in plasma, suggesting that effective levels of drug reached the CNS.

SHH4476g

A multicenter, international, two-cohort, nonrandomized, pivotal, Phase II, company-sponsored study (SHH4476g) enrolled patients with metastatic BCC and those with locally advanced BCC who had inoperable disease or for whom surgery was inappropriate (because of multiple recurrences and a low likelihood of surgical cure, or substantial anticipated disfigurement)[23]. In 33 patients with metastatic BCC, the independently assessed response rate was 30% (95% confidence interval [CI], 16 to 48; p=0.001). In 63 patients with locally advanced BCC, the independently assessed response rate was 43% (95% CI, 31 to 56; p<0.001), with complete responses in 13 patients (21%). The median duration of response was 7.6 months in both cohorts at the time of data cutoff.

Muscle spasms, alopecia, dysgeusia, weight loss, and fatigue occurred in more than 30% of patients, whereas serious adverse events (SAEs) were reported in 25% of patients. Seven deaths due to AEs were reported and none of the deaths were related to vismodegib. Based on the results of this pivotal trial, vismodegib was approved on January 30, 2012 by the United States Food and Drug Administration (US FDA) for the treatment of adults with metastatic basal cell carcinoma, or with locally advanced basal cell carcinoma that has recurred following surgery or who are not candidates for surgery, and who are not candidates for radiation[38].

Vismodegib Most Frequent Treatment Emergent Adverse Events (>10% of patients) at the 18-Month Update

Adverse Event, n (%)	NCI CTCAE Grade, (N = 104)					
	**Total**	**1**	**2**	**3**	**4**	**5**
**Any adverse events**	**104 (100.0)**	**11 (10.6)**	**38 (36.5)**	**34 (32.7)**	**13 (12.5)**	**7 (6.7)**
**Muscle spasms**	74 (71.2)	49 (47.1)	19 (18.3)	6 (5.8)	0	0
**Alopecia**	68 (65.4)	48 (46.2)	20 (19.2)	n/a	n/a	n/a
**Dysgeusia**	57 (54.8)	31 (29.8)	26 (25.0)	n/a	n/a	n/a
**Weight decreased**	53 (51.0)	29 (27.9)	17 (16.3)	7 (6.7)	n/a	n/a
**Fatigue**	44 (42.3)	32 (30.8)	7 (6.7)	4 (3.8)	1 (1.0)	0
**Nausea**	34 (32.7)	25 (24.0)	9 (8.7)	0	0	0
**Decreased appetite**	28 (26.9)	18 (17.3)	7 (6.7)	3 (2.9)	0	0
**Diarrhea**	28 (26.9)	20 (19.2)	5 (4.8)	3 (2.9)	0	0
**Constipation**	20 (19.2)	14 (13.5)	6 (5.8)	0	0	0
**Cough**	20 (19.2)	16 (15.4)	4 (3.8)	0	0	0
**Vomiting**	18 (17.3)	15 (14.4)	3 (2.9)	0	0	0
**Arthralgia**	17 (16.3)	12 (11.5)	4 (3.8)	1 (1.0)	0	0
**Headache**	15 (14.4)	12 (11.5)	3 (2.9)	0	0	0
**Nasopharyngitis**	13 (12.5)	11 (10.6)	2 (1.9)	0	0	0
**Squamous cell carcinoma**	12 (11.5)	3 (2.9)	5 (4.8)	3 (2.9)	0	0
**Ageusia**	12 (11.5)	8 (7.7)	4 (3.8)	n/a	n/a	n/a
**Hypogeusia**	11 (10.6)	10 (9.6)	1 (1.0)	n/a	n/a	n/a

Expanded Access Study (SHH4811g) in Advanced BCC

An open-label, single-arm, multicenter, expanded access study (SHH4811g) of an oral repeating dose of vismodegib was conducted in patients with locally advanced or metastatic BCC, who are otherwise without satisfactory treatment options. Safety of vismodegib and objective response in patients with measurable disease (RECIST v1.0) were assessed[39]. The observed objective response rates were 46.4% (95% CI, 33.0%, 60.3%) for patients with laBCC (n = 56) and 30.8% (95% CI, 17.0%, 47.6%) for patients with mBCC (n = 39). Complete response, partial response and stable disease were observed in 10.7%, 35.7%, and 48.2%, respectively, of patients with laBCC; no patients in this cohort exhibited PD as best response. For patients with mBCC, complete response, partial response and stable disease rates observed were 5.1%, 25.6%, and 51.3%, respectively. 7.7% of patients in this cohort exhibited PD as best response. Among patients with laBCC who responded the median and mean times to response were 2.6 months and 3.5 months, respectively; and among patients with mBCC who responded the median and mean times to response were 2.6 months and 3.8 months, respectively.

As of the final analysis data cutoff date of 23 April 2012, 116 of 119 safety evaluable patients (97.5%) experienced at least one adverse event (22 SAEs total). Adverse events that had the highest reported occurrences (≥ 20%) in 119 safety evaluable patients were: muscle spasms (84 patients; 70.6%), dysgeusia (84 patients; 70.6%), alopecia (69 patients; 58.0%), and diarrhea (30 patients; 25.2%). Only one of 22 SAEs (4.5%) was assessed as being related to treatment with vismodegib: a Grade 3 muscle spasm, reported in 1 patient. There have been three deaths among 119 safety-evaluable patients (2.5%); 2 assessed as unrelated to study drug, and 1 patient with PD.

SHH4811g Vismodegib Expanded Access Study Common Treatment-Emergent Adverse Events

**TEAEs (n=120)**	**Median Time to AE Onset, Days** (95% CI)*	**All AEs, n (%)**	**Gr 1, n (%)**	**Gr 2, n (%)**	**Gr 3, n (%)**	**Gr 4, n (%)**	**Gr 5, n (%)**
**Muscle spasms**	37 (28-44)	84 (70.0)	63 (52.5)	19 (15.8)	2 (1.7)	–	–
**Dysgeusia**	41 (30-51)	84 (70.0)	68 (56.7)	16 (13.3)	n/a	n/a	n/a
**Alopecia**	87 (74-104)	69 (57.5)	57 (47.5)	12 (10.0)	n/a	n/a	n/a
**Diarrhea**	38 (22-116)	30 (25.0)	23 (19.2)	5 (4.2)	1 (0.8)	1 (0.8)	–
**Nausea**	30 (11-130)	23 (19.2)	19 (15.8)	4 (3.3)	–	–	–
**Fatigue**	42 (16-120)	23 (19.2)	14 (11.7)	8 (6.7)	1 (0.8)	–	–
**Weight decreased**	175 (114-293)	19 (15.8)	12 (10.0)	7 (5.8)	–	–	–

*For those patients experiencing the TEAE

MO25616

Study MO25616 (“STEVIE”) is an ongoing Phase II open-label, single-arm, multicenter (ex-United States) study of vismodegib in patients with locally advanced or metastatic BCC who are otherwise without satisfactory treatment options. As of 19 Oct 2012, data were available for 300 patients (278 patients with laBCC and 22 patients with mBCC) with follow-up information for at least 3 months after treatment. The median age of the 300 patients was 72.5 years.

As of 19 Oct 2012, 278 of 300 safety-evaluable patients (92.7%) experienced Grade 3 to 5 treatment-emergent adverse events. The most frequently reported adverse events (≥ safety-evaluable patients), regardless of relationship to study drug, in descending order of frequency, were: muscle spasms (178 patients; 59.3%); alopecia (148 patient; 49.3%); dysgeusia (123 patients; 41.0%); ageusia (77 patients; 25.7%); and asthenia (70 patients; 23.3%).

As of 19 Oct 2012, 53 of 300 patients (17.7%) experienced 74 SAEs. There were 13 deaths reported in the study; 2 were due to disease progression (1 patient each for laBCC and mBCC), 9 were due to AEs assessed by the investigator as unrelated to study drug, 1 was due to an AE assessed by the investigator as related to treatment (cardiopulmonary failure) and 1 was due to “other reason” (multi-organ failure).

A total of 251 of 300 patients had RECIST-measurable disease at baseline and at least one post-baseline assessment. Preliminary efficacy data analysis showed an investigator assessed best overall response (complete response + partial response) of 144/251 (57%). The median time to first best response was 57 days (range, 13 to 363 days).

STEVIE MO25616 Treatment-Emergent Adverse Events (>10% of patients)

Most common TEAEs[a]	All (n=300)	Grade 3	Grade 4
Muscle spasms	178 (59.3%)	15 (5.0%)	0
Alopecia	148 (49.3%)	3 (1.0%)*	n/a
Dysgeusia	123 (41.0%)	6 (2.0%)	0
Ageusia	77 (25.7%)	9 (3.0%)	2 (0.7%)*
Asthenia	70 (23.3%)	5 (1.7%)	0
Weight decreased	48 (16.0%)	3 (1.0%)	0
Decreased appetite	47 (15.7%)	4 (1.3%)	0
Nausea	43 (14.3%)	0	0
Fatigue	38 (12.7%)	5 (1.7%)	0

*Adverse events shown here are as reported by the investigator.

Pharmacokinetics of vismodegib

Briefly, vismodegib’s pharmacokinetic profile is a result of high affinity, reversible binding to Alpha-1 acid Glycoprotein (AAG) and binding to albumin, in addition to solubility limited absorption and slow metabolic elimination properties[40]. Initiation of less frequent administration schedules than the approved dose and schedule of vismodegib of 150 mg orally once daily,(i.e. 150 mg three times weekly or 150 mg once weekly dosing), was associated with marked decrease in the pharmacologically active unbound fraction. Unbound steady-state vismodegib concentrations were 60% and 85% lower for the TIW and QW dose groups, respectively, relative to the QD dose group[36]. Such decreases may be associated with loss of vismodegib activity based on findings from nonclinical models. Integrated PK/PD modeling of vismodegib in

xenograft models has revealed a steep relationship between pathway modulation (GLI1 inhibition) and anti-tumor effect, suggesting that even small reductions in exposure could lead to dramatic loss in vismodegib activity. Dose reduction of vismodegib is not permitted as there is only a 150-mg capsule strength available.

1.6 AZD5363

AZD5363 is a potent, selective inhibitor of the kinase activity of the serine/threonine AKT/PKB (protein kinase B). AKT is part of the AGC family of kinases. Mammalian cells express three closely related AKT isoforms: AKT1 (PKBα), AKT2 (PKBβ) and AKT3 (PKBγ), all encoded by different genes. AKT is a node of multiple signaling pathways promoting tumorigenesis, inhibiting apoptosis, impacting on cell cycle and promoting invasion and migration. The PI3K/AKT/PTEN pathway is frequently deregulated in cancer and drives tumor growth and cell survival. All 3 AKT isoforms are activated in different tumor types including breast, prostate, ovarian, pancreatic and gastric cancers, and this activation is often associated with resistance to established cancer therapies as well as advanced disease and/or poor prognosis. AKT activation in tumors is largely due to input from other signaling pathways upstream of AKT (e.g. mutation of oncogenes such as Ras, Bcr-abl, mutation of receptor tyrosine kinases such as EGFR, amplification of Her2, loss of PTEN function, mutations of PI3K) (AstraZeneca Investigator's Brochure).

AZD5363 is a potent inhibitor of AKT1, 2 and 3 (half maximal inhibitory concentration of the drug [IC50] <10 nM). Non-clinical in vitro and in vivo assays have demonstrated inhibition of phosphorylation of the AKT substrates GSK3β and PRAS40, tumor cell proliferation and xenograft tumor growth models[41]. With treatment of nude mice bearing BT474c xenografts there was time- and dose-dependent inhibition of phosphorylation of the AKT substrates PRAS40 and GSK3β, and the downstream biomarker S6, after single oral doses of 100 and 300 mg/kg. Chronic oral treatment of nude mice bearing a variety of established and primary xenografts with AZD5363, resulted in dose-dependent tumor growth inhibition. Pharmacokinetic exposure increased dose proportionally in the dog but more than dose proportionally in the rat. Minimal accumulation was seen after multiple daily dosing in both the rat and dog. AZD5363 free fraction in human serum albumin and human α1-acid glycoprotein were 29.5% and 66.5-75.8%, respectively. In vitro the major human metabolite was a direct glucuronide conjugate (via UGT1A9 and UGT2B7). CYP3A4 was mainly responsible for the formation of monooxygenated metabolites, with contributions from CYP2C9 and CYP3A5. AZD5363 produced reversible inhibition of CYP2D6, CYP3A4/5, CYP2C9, CYP2B6 and CYP2C19. Time-dependent inhibition of CYP3A4/5 was observed. In a rat quantitative whole-body autoradiography study there was persistence of radioactivity up to at least 168 and 504 hours after dosing in pigmented skin and the uveal tract of the eye respectively. AZD5363 has been found in an in vitro assay to inhibit Organic Cation Transporter 2 (OCT2), found in the human kidney. AZD5363 is also an in vitro inhibitor of BCRP and OATP1B1. The risk of a clinical interaction due to BCRP inhibition is considered low. There is a potential for AZD5363 to cause drug-drug interactions with substrates of OATP1B1 and consequently guidance for the use of sensitive substrates (statins) has been provided in this study. In a clinical setting this has the potential to increase a patient's serum creatinine level, and also to increase the plasma concentration of drugs known to be excreted by this transporter, including metformin. Metformin is currently recommended for the management of hyperglycaemia occurring in patients participating in studies of AZD5363.

The key findings in the toxicology studies were as follows:

- AZD5363 was not an in vitro mutagen, but it increased the incidence of micronucleated immature erythrocytes in the bone marrow of rats at 150 mg/kg/day.

- AZD5363 absorbs light in the ultraviolet spectrum.
- AZD5363 was well tolerated in rats at daily doses of up to 100 mg/kg/day, for up to 28 days. Decreases in body weight and food consumption, increases in plasma levels of glucose and insulin and polyuria, glycosuria and proteinuria were noted. Histopathological changes in the liver (hypertrophy and glycogen accumulation), pancreas (hypertrophy/hyperplasia), male reproductive tract (reduced weight and degeneration), ovaries and uterus (decreased weight), bone marrow and thymus (hypocellularity) and hypertrophy in the pituitary, thyroid and adrenals were seen. The majority of these findings were reversible, but the changes in the testes and bone marrow had not reversed at the end of the 28 day recovery period.
- Increased plasma enzyme levels (aspartate aminotransferase [AST], alanine aminotransferase [ALT] and glutamate dehydrogenase) were present in rats at dose levels above 30 mg/kg/day, together with a single incidence of liver necrosis at 100 mg/kg/day, and 2 incidences of liver necrosis at 150 mg/kg/day in a dose range finding study. The changes in plasma enzyme levels were reversible and necrosis was not present at the end of the 28 day recovery period.
- Daily oral doses of AZD5363 to dogs for up to 28 days at doses up to 30 mg/kg/day were tolerated, but were associated with clinical signs and reduced body weight and food consumption. Increases in plasma levels of glucose and insulin were noted and QTcR was increased at 30 mg/kg/day (reversible), together with evidence of polyuria.
- Secondary and Safety Pharmacology studies have been carried out to investigate the effects of AZD5363 on related and unrelated receptors and enzymes, the cardiovascular, central and peripheral nervous, respiratory, gastrointestinal and renal systems. The key findings were that:
    - AZD5363 is active at the hERG (human Ether-à-go-go Related Gene) channel with an IC50 of 73 uM.
    - There was an increase in cardiac contractility and QTcR (reversible) at doses of 30 and 40 mg/kg in the dog cardiovascular model.
    - Following a single dose of 100 or 150 mg/kg, there were effects on spontaneous activity and touch response (Central Nervous System assessment); inhibition of gastric emptying (both) and decreased intestinal transit (150 mg/kg only); and glucosuria, diuresis and increased fractional excretion of electrolytes (renal study). Further details are provided in the Investigators' Brochure.

1.7 Clinical Experience with AZD5363 as Monotherapy

To date 5 clinical studies sponsored by AstraZeneca using AZD5363 have been initiated as of the cut-off date of 04 October 2017. A total of 456 patients have been treated with AZD5363 as either monotherapy or in combination dosing with paclitaxel in the studies that are included in this IB. Of these 5 studies, 2 are ongoing, 2 have completed (Studies D3610C00004 and D3610C00007), and 1 (Study D3610C00003) has been terminated early following an unscheduled interim analysis that indicated that AZD5363 was unlikely to generate a positive efficacy signal in this setting. In addition, 18 investigator sponsored studies are planned or

have commenced recruitment. The AEs of hyperglycaemia, rash, diarrhea, hypersensitivity, stomatitis, dry skin, and pruritus are expected for AZD5363. Additional AEs that are commonly reported for AZD5363, (those affecting >30% in the pooled monotherapy intermittent group), irrespective of causality, are decreased appetite, nausea, vomiting, and fatigue. Patients enrolled

in the AZD5363 clinical studies have significant comorbidities, multiple concomitant medications, and have been exposed to other anticancer treatment before receiving AZD5363.

Hyperglycaemia: Hyperglycaemia is a frequent clinical observation, but is transient and reversible on cessation of treatment. To date, the overall incidence of hyperglycaemia (i.e. at least 1 post baseline laboratory report of glucose above the ULN) is 95.6% in the intermittent monotherapy 480 mg BD 4 days on 3 days off dosing schedule, 100% in the intermittent 400 mg BD 4 days on 3 days off combination with paclitaxel schedule, and 92.3% in the intermittent 400 mg BD 4 days on; 3 days off combination with fulvestrant schedule. The majority of cases occurred within the first week of study treatment (88.5% in patients treated with AZD5363 monotherapy 480 mg BD intermittent dosing, 100% in patients treated with AZD5363 400 mg BD intermittent with paclitaxel, and 84.6% in patients treated with AZD5363 400 mg BD intermittent with fulvestrant). The proportion of patients who received metformin for elevated glucose levels ranged from 0% to 31.7% (AZD5363 intermittent monotherapy).

Rash: Rash is a frequent clinical observation. To date, the overall incidence of rash (an SMQ term including the preferred terms of rash, erythema, rash erythematous, rash macular, rash maculo-papular, rash papular, rash pruritic) is 46.4% in the intermittent monotherapy 480 mg BD 4 days on 3 days off dosing schedule, 57.1% in the intermittent 400 mg BD 4 days on; 3 days off combination with paclitaxel schedule, and 26.9% in the intermittent 400 mg BD 4 days on 3 days off combination with fulvestrant schedule. The proportion of patients with an AE of rash of CTCAE Grade 3 or above was 16.9%, 14.3%, and 11.5%, respectively. In the intermittent monotherapy 480 mg BD 4 days on; 3 days off dosing schedule, 39.3% of patients had a rash that was considered to be causally related to AZD5363. Seven patients (3.8%) had an SAE of rash, and 7 (3.8%) discontinued AZD5363 treatment due to rash. A total of 51 patients (27.9%) received treatment for rash.

Diarrhea: Diarrhea is a frequent clinical observation. To date, the overall incidence of diarrhea (relating to the SMQ term diarrhea) is 79.8% in the intermittent monotherapy 480 mg BD 4 days on; 3 days off dosing schedule, 85.7% in the intermittent 400 mg BD 4 days on; 3 days off combination with paclitaxel schedule, and 57.7% in the intermittent 400 mg BD 4 days on; 3 days off combination with fulvestrant schedule. The proportion of patients with an AE of diarrhea of CTCAE Grade 3 or above was 16.9%, 28.6%, and 3.8%, respectively. In the intermittent monotherapy 480 mg BD 4 days on; 3 days off dosing schedule, 74.3% of patients had an AE of diarrhea that was considered to be causally related to AZD5363. Twelve patients (6.6%) had an SAE of diarrhea, and 2 (1.1%) discontinued AZD5363 treatment due to diarrhea. A total of 108 patients (59.0%) received treatment for diarrhea.

Hypersensitivity: Hypersensitivity is a commonly reported clinical observation being reported in 4 patients on the intermittent monotherapy dose of 480 mg BD 4 days on; 3 days off, and for 1 patient on the intermittent 400 mg BD 4 days on; 3 days off combination with paclitaxel schedule. Symptoms of hypersensitivity included rash in association with 1 or more of AEs of the following AEs: flushing, pruritus, urticaria, throat itchiness, pyrexia, and facial and/or lip oedema. In all patients, hypersensitivity or related AEs were considered causally related to AZD5363, and in 4 patients the reported AEs were considered serious, leading to hospitalization or prolonged hospitalization. One patient had a reported history of allergy to heat and cold. In all patients, the symptoms resolved with AZD5363 discontinuation and treatment with antihistamines and steroids.

Stomatitis: Stomatitis is a very common clinical observation, affecting 32 patients (14.0%) in AZD5363 intermittent monotherapy, 10 patients (26.3%) in combination with paclitaxel, and 2 patients (7.7%) in combination with fulvestrant. In AZD5363 intermittent monotherapy, the majority of stomatitis AEs were Grade 1 or 2 (91.4%), and the maximum severity of AEs experienced by patients was CTCAE Grade 3 (8.6%). There were 3 AEs leading to dose

interruption, and of these, all resulted in a positive de-challenge, and in the 2 patients subsequently re-administered the study drug, both received a reduced AZD5363 dose and both experienced negative re-challenge. The median duration of all AEs was 15 days.

Dry skin: Dry skin is a very common clinical observation, affecting 27 patients (11.8%) in AZD5363 intermittent monotherapy, 6 patients (15.8%) in combination with paclitaxel, and 1 patient (3.8%) in combination with fulvestrant. In AZD5363 intermittent monotherapy, the majority of dry skin AEs were Grade 1 (92.9%), and the maximum severity of AEs experienced by patients was CTCAE Grade 2 (7.1%). There were no AEs leading to dose interruption, reduction, or study discontinuation. The median duration of all AEs was 91.5 days.

Pruritus: Pruritus is a common clinical observation, affecting 21 patients (9.2%) in AZD5363 intermittent monotherapy group, 7 patients (18.4%) in combination with paclitaxel, and none in combination with fulvestrant. In AZD5363 intermittent monotherapy, the majority of pruritus AEs were CTCAE Grade 1 or 2, and 1 AE was Grade 3. There were no AEs leading to dose interruption or study discontinuation. The median duration of all AEs was 37 days.

Preliminary efficacy results: In general, the AZ-sponsored studies reported in this protocol have been primarily designed to determine the MTDs and recommended doses for Phase 2 for the various proposed AZD536 dosing schedules in monotherapy and in combination therapy. As such, the studies have recruited patients from advanced cancer populations that have been heavily pretreated and who show resistance to a number of prior therapies. None of the dose-finding parts of the studies presented here have selected for patients whose tumors harbor PIK3CA or AKT mutations. However, tumor response data are available from 131 patients from Studies D3610C00001 (Parts A and B) and D3610C00004. These results show that 3 of the 131 patients had RECIST partial responses (PRs): 1 whose tumor was positive for PIK3CA mutation and 2 where the tumor tested positive for AKT1 mutation. Tumor mutation status was not systematically determined in these Phase 1 studies. The expansion parts of Study D3610C00001 (Parts C and D) recruited an advanced cancer population selected for patients with proven PIK3CA (Part C) or AKT1 (Part D) mutations. In patients with ER+ breast cancer, 1 patient had a confirmed RECIST response of PR in Part C (PIK3CA mutation). In patients with gynecological cancer, 2 patients with PIK3CA-mutant cancer had a confirmed RECIST response of PR (AstraZeneca Investigator's Brochure).In patients with AKT1 E17K-mutant tumors (n=52) and a median of five lines of prior therapy, the median PFS was 5.5 months (95% Cl, 2.9 to 6.9 months), 6.6 months (95% Cl, 1.5 to 8.3 months), and 4.2 months (95% Cl, 2.1 to 12.8 months) in patients with estrogen receptor-positive breast, gynecologic, and other solid tumors, respectively.[24]

Notably, durable disease control (with a minor radiographic response) on AZD5363 monotherapy was demonstrated in a patient with a recurrent meningioma harboring an AKT1 mutation [42].

1.8 GSK2256098

Focal adhesion kinase (FAK) is a non-receptor tyrosine kinase that integrates signals from integrins and growth factor receptors. FAK has been reported to have a role in the regulation of cell survival, signaling, growth, adhesion, migration, and invasion[43]. Overexpression of FAK mRNA and/or protein has been documented in many solid human tumors[43]. FAK is key in suppressing suspension-induced cell death (anoikis) with both phosphorylation of FAK and FAK kinase activity being important[44]. Suppressing FAK activity by antisense oligonucleotides or antibody injection leads to induction of apoptosis[45,46].

Because of the role of FAK in cancer progression, invasion, and metastasis, several small molecule inhibitors of FAK are in clinical development. GSK2256098 is a potent, reversible inhibitor of FAK enzymatic activity with an apparent Ki = 0.4 nM against the purified enzyme.

GSK2256098 is very selective with no significant inhibitory activity from screening approximately 300 kinases. GSK2256098 inhibits phospho-FAK (pFAK, (Y397)) in a concentration-dependent manner in human tumor cells. The IC50 value determined in the human ovarian OVCAR8 cell line is 15 nM. FAK remains inhibited in the continued presence of compound for up to 24 hours, the longest time point evaluated. Evaluations of GSK2256098 in other cell lines that are derived from different tissues of origin (lung and brain) have very similar IC50 values. GSK2256098 does not inhibit the growth of human tumor cell lines tested in a standard two dimensional cell culture growth assay. Importantly, growth inhibitory activity for GSK2256098 is observed in a concentration and a tumor cell line dependent manner when evaluated in anchorage independent cell growth assays.

Oral administration of GSK2256098 induces a PD response (pFAK, (Y397) inhibition) in tumor tissue from mice bearing human tumors. Dose and time dependent inhibition of pFAK was observed in subcutaneous xenograft models with mice bearing OVCAR8 (ovarian) or U87MG (glioma) human tumors. The amount of pFAK inhibition correlates well with the concentrations of GSK2256098 in blood. (GSK Investigator's Brochure).

1.9 Clinical Experience with GSK2256098

FAK113581

FAK113581 was a single ascending dose, placebo-controlled study in healthy subjects at doses ranging from 20 – 240 mg. Safety evaluations included AE reporting, clinical laboratory tests (hematology, chemistry, urinalysis), vital signs (blood pressure and heart rate), 12-lead EKGs, clinical monitoring and observations. AEs were reported in 18 of 28 subjects (64%) who received GSK2256098 and in 4 of 10 subjects (40%) who received placebo. The most frequently reported AEs were headache (5 subjects, 13%; 3 subjects in active dose groups) and lethargy (3 subjects, 8%; all active dose groups). Adverse events were distributed across all dose groups, with no obvious dose-related trends, and resolved prior to completion of the study. Except for six Grade 2 (moderate severity) AEs in three subjects, all adverse events were Grade 1 (mild) severity. The Grade 2 AE were: 1 subject with headache and presyncope in the placebo group, 1 subject with post procedural discomfort and soft tissue injury in the 80 mg Group, 1 subject with urinary casts and proteinuria in the 140 mg with food group. Clinically significant changes in clinical laboratory values, vital signs, and EKGs were not observed, with the exception of a single post-dose, reversible increase in proteinuria after a single oral dose of GSK2256098 of 140 mg. No deaths, SAEs, or premature withdrawals due to AEs were reported.

FAK113517

Sixty-two subjects were enrolled in Study FAK113517, Parts 1, 2, and 3, a multiple ascending dose, single arm study in subjects with advanced, refractory solid tumors. Subjects received continuous daily oral doses of GSK2256098 at doses ranging from 80 mg to 1500 mg BID. Overall in Parts 1, 2 and 3 of FAK113517, the most frequently reported AEs associated with continuous oral BID dosing of GSK2256098 were nausea (76%), diarrhea (65%), vomiting (58%), decreased appetite (47%), proteinuria (26%), fatigue (24%), asthenia (23%), hyperbilirubinaemia (23%), constipation (21%) and hypercholesterolemia (21%).

All AEs at the 80 mg BID, 160 mg BID, and 600 mg BID dose levels were Grade 1 or 2. Twenty-six subjects had AEs Grade 3. There were two Grade 4 AEs: reversible, exercise-induced increase in blood creatinine phosphokinase level and cerebral vascular accident. There were no Grade 5 AEs. There were two AEs with unknown toxicity Grade (blister in one subject and Dupuytren's Contracture in another). AEs related to changes in clinical laboratory tests occurred at the 300 mg, 750 mg, 1000 mg, 1250 mg and 1500 mg BID dose levels. The most common (occurring in >10% of subjects) were proteinuria (26% of subjects), hyperbilirubinaemia (23%), hypercholesterolemia (21%), hematuria (16%), hypertriglyceridaemia (15%), anemia (13%),

hypoalbuminaemia (10%) and hypomagnesaemia (10%). All AEs related to clinical laboratory tests were Grade 1, Grade 2 or Grade 3, except for a single Grade 4, reversible, exercise-induced AE of increase in blood creatinine phosphokinase level. The MTD of GSK2256098 was established at 1000 mg BID based upon an overall assessment of safety and tolerability. During the dose escalation phase of the study, dose-limiting toxicities (DLTs) were reported by 4 subjects. One subject enrolled in the 1000 mg BID dosing cohort, experienced intermittent, Grade 2 urine protein/creatinine ratio increased. One subject enrolled in the 1250 mg BID dosing cohort experienced intermittent, Grade 2 nausea, vomiting and fatigue (even though only one DLT was experienced at the 1250 mg dose, the overall tolerability was poor, eliminating this as the recommended dose). Two subjects enrolled in the 1500 mg BID dosing cohort experienced dose-limiting AEs; one subject experienced Grade 2 fatigue and one subject experienced intermittent, Grade 3 asthenia. Five subjects had events that led to permanent discontinuation of study medication. One subject enrolled in the 1000mg BID dosing level experienced intermittent, Grade 2 decreased appetite. One subject enrolled in the 1250 mg BID dosing level experienced intermittent, Grade 2 asthenia. One subject enrolled in the 1500 mg BID dosing level, experienced intermittent, Grade 3 loss of consciousness. Two subjects enrolled in the 1000 mg BID Dose Expansion dosing cohort experienced AEs that lead to permanent discontinuation of study medication. One subject experienced a single episode of Grade 3 bile duct obstruction and a single episode of Grade 1 cholangitis, and one subject experienced a single episode of Grade 3 interstitial lung disease. Fourteen SAEs were reported (12 subjects), all at the MTD or higher. Pleural effusion (n=2) was the only event reported for more than one subject. There were no fatal AEs or SAEs. Four subjects (6%) died on study, all attributable to disease progression.

Pharmacokinetics of GSK2256098

In subjects with solid tumors, GSK2256098 is rapidly absorbed with a time to maximal concentration of about 1 to 3 hours. At the 1000 mg dose level, the geometric mean half-life was 4.4 h (25% CV). (GSK Investigator’s Brochure).

1.10 Abemaciclib

During the cell cycle, the G1 restriction point controls entry into S phase and is essential for maintaining control of cell division. The cyclin-dependent kinases (CDKs), CDK4 and CDK6, participate in a complex with D-type cyclins to initiate the transition through the G1 restriction point by phosphorylating and inactivating the retinoblastoma (Rb) tumor-suppressor protein. Alterations in this pathway occur frequently in human cancers and involve (1) loss of CDK inhibitors by mutation or epigenetic silencing, (2) mutation/overexpression of either CDK4 and CDK6 or cyclinD, or (3) inactivation of Rb. These alterations render cells less dependent on mitogenic signaling for proliferation. With the possible exception of those tumors with complete inactivation of Rb, which functions downstream of the CDK4 and CDK6–cyclinD complex, all of these cancers are potentially sensitive to pharmacologic inhibition of CDK4 and CDK6. From a therapeutic standpoint, the goal of inhibiting CDK4 and CDK6 with a small molecule inhibitor is to prevent cell cycle progression through the G1 restriction point, thus arresting tumor growth.

Abemaciclib represents a selective and potent small molecule CDK4 and CDK6 dual inhibitor with a broad antitumor activity in preclinical pharmacology models, acceptable physical and pharmacokinetic (PK) properties, and an acceptable toxicity profile in nonclinical species. Formally, abemaciclib refers to the freebase whereas LSN2813542 refers to abemaciclib mesylate; however, abemaciclib is used for uniformity throughout this Investigator’s Brochure (IB), except when important for experimental clarity. This compound demonstrates significant inhibition of tumor growth as monotherapy in multiple human xenograft models including models for: breast cancer, colorectal cancer, glioblastoma multiforme, acute myeloid leukemia, melanoma, mantle cell lymphoma (MCL), and non–small-cell lung cancer (NSCLC). Although characterized by a different constellation of genomic mutations, each of these human xenografts

has an intact, functional Rb protein. Xenograft growth inhibition is generally dose dependent from 25 to 100 mg/kg following daily oral administration for 21 to 28 days. Additional nonclinical studies in xenograft models for human NSCLC and melanoma also show that abemaciclib may be used in combination with standard cytotoxic or targeted therapies to improve efficacy of these agents.

Preclinical data: In nonclinical species, abemaciclib distributes efficiently to the brain and potentially provides a unique opportunity to treat primary brain tumors as well as cancers that have metastasized to the brain. As a result of its brain exposure, treatment with abemaciclib in a rat orthotropic brain tumor model produces statistically significant and dose-dependent improvement in survival. Abemaciclib demonstrates moderate-to-high bioavailability in preclinical species. In repeat-dose toxicokinetic studies, abemaciclib showed generally dose-dependent exposure with no gender differences. Abemaciclib is highly metabolized, and hepatic elimination plays a major role in the clearance of abemaciclib and its metabolites in rats, dogs, and humans. In humans, the terminal elimination half-life (t 1/2) in plasma ranges from approximately 17 to 38 hours across the dose range tested. At a single dose of 200 mg, the mean apparent oral clearance (CL/F) is 38.3 L/hour with a high interindividual variability (105% coefficient of variation [CV]) and the apparent volume of distribution is large at 1300 L (96% CV). In the xenograft models and skin biopsies from patients, abemaciclib inhibited phosphorylated Rb (pRb) and topoisomerase II alpha (TopoIIα) at clinically relevant doses and exposures.

In preclinical species, the primary target organs for toxicity (associated with up to 3 months of continuous daily dosing) are bone marrow (resulting in pancytopenia), gastrointestinal tract, lymphoid tissues, and male reproductive tract. All of these changes demonstrated complete or partial reversibility during the recovery period. In humans, the most common (≥10%) treatment-emergent adverse events (TEAEs) possibly related to the study drug for patients who received single agent abemaciclib include diarrhea, nausea, fatigue, neutropenia, vomiting, decreased appetite, leukopenia, thrombocytopenia, anemia, abdominal pain, and blood creatinine increased. Increased rates of skeletal and cardiac variations and malformations, accompanied by decreased fetal weights, were observed in an embryo–fetal development study of abemaciclib in rats. See Investigator Brochure for more details.

1.11 Clinical Experience with Abemaciclib

As of September 22, 2016, 12 clinical studies were completed (7 in healthy subjects and 5 in patients) and 19 clinical studies are ongoing (3 in healthy subjects and 16 in patients) with abemaciclib. The PK profile of abemaciclib was studied using data available from a total of 222 patients enrolled in Study I3Y-MC-JPBA (JPBA). A general overview of abemaciclib PK based on several studies in healthy subjects and cancer patients (Table 6.2) is presented below. In Study JPBA, the mean concentration time profiles for abemaciclib and its metabolites at a dose of 200 mg in cancer patients are depicted in **Figure 4**:

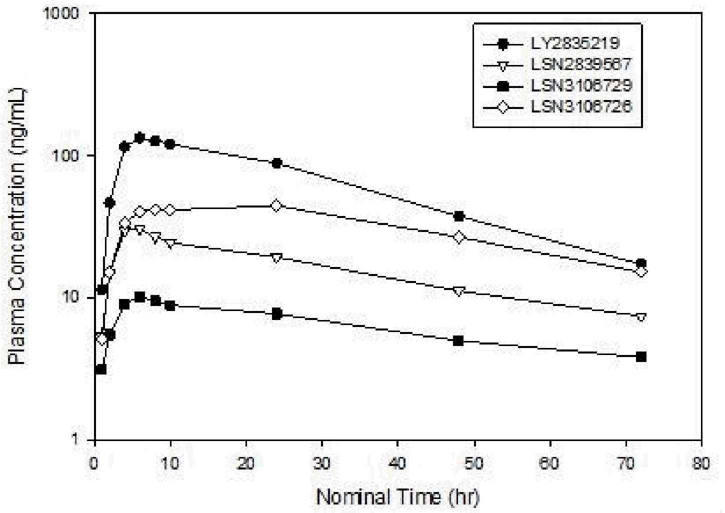

Figure 4: Abemaciclib and its metabolites' exposures after a single 200-mg dose in Study JPBA.

Safety Data: SAEs are summarized below for events that were experienced by patients up to 22 September 2016 based on data available in LSS. Per LSS, as of 22 September 2016, 405 patients who received abemaciclib in Studies JPBA, JPBB, JPBC, JPBE, JPBH, JPBJ, JPBK, JPBN, JPBO, JPBX, JPBY, JPBZ, and JPCB experienced 724 SAEs. A total of 167 patients in 11 of these studies experienced 264 SAEs that were possibly related to the study drug. SAEs that were possibly related to study drug and were experienced by 2 or more patients who received abemaciclib monotherapy are summarized in **Table 2**:

Table 2: Serious Adverse Events Possibly Related to Study Drug Experienced by 2 or More Cancer Patients Treated with Abemaciclib Monotherapy

Event Preferred Term	Number of Patients
Nausea	9
Acute kidney injury	7
Dehydration	7
Vomiting	6
Pneumonia[a]	6
Thrombocytopenia[b]	6
Neutropenia[c]	6
Anaemia	5
Blood creatinine increased	4
Lung infection	4
Sepsis	4
Abdominal pain[d]	3
Confusional state	3
Decreased appetite	3
Hypokalaemia	3
Pneumonitis	3
Pyrexia	2
Encephalopathy	2

Enterocolitis infectious	2
Febrile neutropenia	2
General physical health deterioration	2
Leukopenia[e]	2
Pancreatitis	2
Pancytopenia	2
Renal failure	2

a Pneumonia = events of pneumonia, pneumocystis jirovecii pneumonia, and pneumonia fungal.
b Thrombocytopenia = events of thrombocytopenia and platelet count decreased.
c Neutropenia = events of neutropenia and neutrophil count decreased.
d Abdominal pain = events of abdominal pain and abdominal pain upper.
e Leukopenia = events of leukopenia and white blood cell decreased.

Cumulatively in healthy subjects, the most common (≥5%) AEs that were possibly related to study drug included headache (13.1%), nausea (10.0%), diarrhea (6.9%), and vomiting (5.4%). One healthy subject had an SAE of syncope possibly related to study drug. No deaths were reported in these studies. One subject in Study JPBF, who received 200-mg abemaciclib and 600-mg rifampin, discontinued due to mild (CTCAE Grade 1) vomiting.

Validated safety data for 870 patients treated with abemaciclib in Studies JPBA, JPBB, JPBC, JPBE, JPBH, JPBJ, JPBN, and JPBY are available (Lilly Investigator Brochure). Overall, the most common TEAEs (≥10%) possibly related to study drug for patients receiving abemaciclib as a single agent are diarrhea, nausea, fatigue, neutropenia, vomiting, decreased appetite, thrombocytopenia, anemia, leukopenia, abdominal pain, and blood creatinine increased. In Study JPBH, when abemaciclib was combined with endocrine therapy (anastrozole, letrozole, tamoxifen, exemestane), diarrhea has been reported in 98.5% of the metastatic breast cancer patients; and in 86.0% of the metastatic breast cancer patients when combined with targeted agents (exemestane + everolimus, or trastuzumab). As a result of the higher incidence of diarrhea, the recommended abemaciclib dose is 150 mg Q12H when combined with these agents. Furthermore, it was noted in Study JPBY (abemaciclib 150 mg Q12H in combination with anastrozole), diarrhea was reduced at 52% when patients received loperamide prophylactically. The majority of deaths reported for patients in the presented studies with available safety data were due to disease progression. Per LSS as of 22 September 2016, 167 patients in the ongoing and completed cancer patient studies experienced 264 SAEs that were reported as possibly related to study drug. SAEs that were possibly related to study drug and experienced by ≥5 patients receiving abemaciclib monotherapy included diarrhea (14 patients); nausea (9 patients); acute kidney injury and dehydration (7 patients each); anemia, pneumonia, and vomiting (6 patients each); and neutropenia and thrombocytopenia (5 patients each). SAEs that were possibly related to study drug and experienced by ≥5 patients receiving abemaciclib combinations included neutropenia (5 patients; abemaciclib with pemetrexed) and dehydration (5 patients; abemaciclib with endocrine therapy).

In the recently published MONARCH 1 study, a Phase II study of single agent abemaciclib in refractory metastatic breast cancer, the most common treatment-related AEs of any grade were diarrhea, fatigue, and nausea. Discontinuation of abemaciclib from AEs was infrequent (7.6%).67

Efficacy Data: The results suggest that abemaciclib has clinical activity in patients with NSCLC and metastatic breast cancer. A summary of the results for patients in these populations is provided below. In Study JPBA (Phase I study of abemaciclib in patients with advanced cancer), two of 68 patients with NSCLC (2.9%) had a partial response (PR), including 1 patient who received abemaciclib 150 mg Q12H and 1 patient who received abemaciclib 200 mg Q12H.

Among the remaining patients, 31 (45.6%) had stable disease (SD), 16 (23.5%) had PD, and 19 (27.9%) were not evaluable. In the metastatic breast cancer cohort, 11 of the 47 patients had a PR including 7 at the 150-mg Q12H dose and 4 at the 200-mg Q12H dose. One patient (2.1%, this patient received 150 mg Q12H) had a complete response (CR). Among the remaining patients, 11 (23.4%) had SD for ≥24 weeks, 10 (21.3%) had SD for <24 weeks, 11 (23.4%) had PD, and 3 (6.4%) were not evaluable.

1.12 Registration Quality of Life (QOL) Measurements

QOL measurements of fatigue and overall perception of QOL are routinely included in Alliance studies and will be assessed upon registration in this study. Evidence has arisen indicating that baseline single-item assessments of fatigue and overall QOL are strong prognostic indicators for survival in cancer patients, independent of performance status. This evidence was derived from two separate meta-analyses recently presented at ASCO, the first involving 23 NCCTG and Mayo Clinic Cancer Center oncology clinical trials, the second involving 43 clinical trials. Routine inclusion of these measures should be considered similar to that of including performance status, either as stratification or prognostic covariates. It will take approximately one minute to complete this measure.[47, 48]

1.13 Central Radiology Review

Scans will be collected, stored and centrally reviewed as a secondary endpoint. As this is the first trial to evaluate the role of these inhibitors in meningiomas, central review and storage of images will allow us to collect additional information on the effect of these agents radiographically, in particular, to look tumor volumetric changes which may be more informative than area measurements. Furthermore, as the radiographic response to these agents is not known, central review of the meningiomas will allow us to more uniformly assess the response of therapy.

1.14 Impact of the Trial

Based on the biomarker work, we have designed a phase 2 study of vismodegib (SMO inhibitor), AZD5363 (AKT inhibitor), GSK2256098 (a FAK inhibitor), or abemaciclib (CDK inhibitor) in patients with recurrent or progressive meningiomas harboring genetic alterations in SMO/PTCH1, AKT1/PIK3CA/PTEN, NF2, or the CDK pathway (CDK4/CDK6/CCND1/CCND2/CCND3/CCNE1/CDKN2A), respectively. This study represents a novel therapeutic approach in meningioma, a disease with a critical need for effective therapy.

2.0 OBJECTIVES

2.1 Primary objectives

2.1.1 To determine the activity of a SMO inhibitor in patients with meningiomas harboring SMO and PTCH1 mutations as measured by 6-month PFS and response rate.

2.1.2 To determine the activity of a FAK inhibitor in patients with meningiomas harboring NF2 mutations as measured by 6-month PFS and response rate.

2.1.3 To determine the activity of an AKT inhibitor in patients with meningiomas harboring AKT1/PIK3CA/PTEN mutations as measured by 6-month PFS and response rate.

2.1.4 To determine the activity of a CDK inhibitor in patients with meningiomas harboring alterations in the CDK pathway or NF2 alterations as measured by 6-month PFS and response rate.

2.2 Secondary objectives

2.2.1 To determine overall survival and progression-free survival of SMO, FAK, AKT and CDK inhibitors in patients with meningioma.

2.2.2 To determine adverse event rates of SMO, FAK, AKT and CDK inhibitors in patients with meningioma.

2.2.3 To determine the activity of SMO, FAK, AKT and CDK inhibitor as measured by response rate by central radiology review.

2.3 Correlative science objectives

2.3.1 To evaluate genetic and histological biomarkers in meningioma that are associated with treatment response, toxicity, and/or clinical variables, as well circulating/cell-free biomarkers.

2.3.2 To evaluate dynamic contrast enhanced MRI during treatment with SMO, FAK, AKT and CDK inhibitors for meningioma.

2.3.3 To evaluate volumetric response by central radiology review.

3.0 PATIENT SELECTION

For questions regarding eligibility criteria, see the Study Resources page. Please note that the Study Chair cannot grant waivers to eligibility requirements.

3.1 On-study guidelines

This clinical trial can fulfill its objectives only if patients appropriate for this trial are enrolled. All relevant medical and other considerations should be taken into account when deciding whether this protocol is appropriate for a particular patient. Physicians should consider the risks and benefits of any therapy, and therefore only enroll patients for whom this treatment is appropriate.

The following may seriously increase the risk to the patient entering this protocol:

- Psychiatric illness which would prevent the patient from giving informed consent.
- Medical condition such as untreated or uncontrolled fungal, bacterial or viral infections (including HIV), active bleeding diathesis, uncontrolled diabetes mellitus, hypertension, or cardiac disease which, in the opinion of the treating physician, would make this protocol unreasonably hazardous for the patient.
- Any other concurrent severe and/or uncontrolled medical condition that would, in the treating physician’s judgment, cause unacceptable safety risks, contraindicate patient participation in the clinical study, or compromise compliance with the protocol.
- Patients with a “currently active” second malignancy other than non-melanoma skin cancers. Patients are not considered to have a “currently active” malignancy if they have completed therapy and are free of disease for ≥ 3 years.
- Patients who cannot swallow oral formulations of the agent(s).

In addition:

Reproductive considerations, vismodegib:

<u>Serious or Life-threatening Birth Defect Effects of Vismodegib</u>

Studies have demonstrated that inhibition of the Hh pathway in embryos results in brain, facial, and other midline defects, including holoprosencephaly or microencephaly, cyclopia, absent nose, cleft palate, tooth abnormalities, and bone development abnormalities (Bale, 2002). While the effects of vismodegib on the developing human fetus at the recommended therapeutic dose are unknown, women of childbearing potential and men must agree to use two methods of contraception (i.e., barrier contraception and another method of contraception) prior to study entry, for the duration of study participation, and for 24 months following treatment (for women) and 2 months (for men).

Vismodegib may impair fertility. Amenorrhea has been observed in clinical trials in women of childbearing potential. Based on animal studies, reversibility of fertility impairment is unknown. Fertility preservation strategies should be discussed with women of childbearing potential prior to starting treatment with vismodegib. Effects on testes and epididymides characterized by mild to moderate germ cell degeneration in seminiferous tubules, relative paucity of spermatozoa, and increased cellular debris in epididymides were observed in male dogs at all dose levels tested and were consistent with the pharmacologic activity of the drug. There were no changes in Leydig or Sertoli cells in any animal. Evidence of partial recovery was noted after a 4-week recovery period.

Germ cell degeneration in male patients is likely to occur at pharmacologically active doses. There is no specific mitigation strategy for this vismodegib toxicity; however, male patients should be made aware of it during the consent process. Although this effect is expected to be

reversible with discontinuation of dosing, long-term effects on male fertility cannot be excluded at this time.

Women of child-bearing potential must use two forms of contraception (including 1 form of barrier contraception) starting at least 4 weeks prior to study entry, for the duration of study participation, and for at least 24 months post-treatment. Appropriate methods of birth control include abstinence, combination hormonal contraceptives, subcutaneous hormonal implant, hormonal patch, hormonal contraceptives (levonorgestre-releasing intrauterine system, medroxyprogesterone acetate depot), tubal sterilization, intrauterine device, vasectomy or barrier method. Acceptable forms of barrier contraception include the following: Any male condom (with spermicide) or diaphragm (with spermicide). Should a woman become pregnant or suspect she is pregnant while participating in this study, she should inform her treating physician immediately. Women should not breastfeed children for 24 months after the last dose of vismodegib.

Vismodegib is present in semen. It is not known if the amount of vismodegib in semen can cause embryo-fetal harm. Advise male patients to use condoms, even after a vasectomy, to avoid drug exposure to pregnant partners and female partners of reproductive potential initiated prior to registration, for the duration of study participation, for 2 months after the final dose of vismodegib. Advise males of the potential risk to an embryo or fetus if a female partner of reproductive potential is exposed to vismodegib. Advise males not to donate semen during therapy with and for 2 months after the final dose of vismodegib.

See Section 9.3.1 for reporting requirements.

Due to the teratogenic potential of vismodegib, all patients should not donate blood or blood products during the study and for 24 months after discontinuation of vismodegib

Reproductive considerations, GSK2256098

GSK2256098 has not been tested in pregnant or lactating women.

Women of child-bearing potential and men with female partners of childbearing potential must use two forms of contraception (i.e., barrier contraception and one other method of contraception) at least 4 weeks prior to study entry, for the duration of study participation, and for at least 6 months post-treatment. Appropriate methods of birth control include abstinence, oral contraceptives, implantable hormonal contraceptives or double barrier method (diaphragm plus condom). Should a woman become pregnant or suspect she is pregnant while participating in this study, she should inform her treating physician immediately. Men treated or enrolled on this protocol must also agree to use adequate contraception initiated prior to registration, for the duration of study participation, and 6 months after completion of drug administration.

Reproductive considerations, AZD5363

In an animal model, AZD5363 causes testicular pathology. AZD5363 had an adverse effect on embryonic survival and early postnatal growth when administered to pregnant rats. Use of two forms of highly reliable contraceptives by female patients of child-bearing potential is required throughout the study and for at least 4 weeks after the last dose of study drug. Appropriate methods of birth control include true abstinence, combination hormonal contraceptives, subcutaneous hormonal implant, hormonal patch, hormonal contraceptives (levonorgestre-releasing intrauterine system, medroxyprogesterone acetate depot), (as it is not known if AZD5363 has the capacity to affect the metabolism of hormonal contraceptives, hormonal contraception should be combined with a barrier method of contraception), tubal sterilization, intrauterine device, vasectomy or barrier method. Acceptable forms of barrier contraception include the following: Any male condom (with spermicide) or diaphragm (with spermicide). Male patients are required to use barrier methods of contraception. It is not known whether the preclinical changes seen in the male animal reproductive organs, after treatment with AZD5363

will be fully reversible or will permanently affect the ability to produce healthy sperm following treatment. Male patients wishing to father children should be advised to arrange for freezing of sperm samples prior to the start of study treatment. Breastfeeding women are also excluded from study entry.

Reproductive considerations, abemaciclib:

General Guidance for Women of Child Bearing Potential and/or Use of Contraceptive Methods

Based on findings in animals, abemaciclib can cause fetal harm when administered to a pregnant woman. In animal studies, abemaciclib was teratogenic and caused decreased fetal weight at maternal exposures that were similar to human clinical exposure based on the area under the plasma concentration versus time curve (AUC) at the recommended human dose. Therefore, teratogenicity is considered an important potential risk for abemaciclib. There are no available human data informing the drug-associated risk. Advise pregnant women of the potential risk to a fetus. Additionally, there are no available data on effects of breastfeeding. Advise a nursing woman to discontinue breastfeeding during treatment with abemaciclib. The following instructions must be included in investigator-sponsored protocols.

- A female of childbearing potential, must have a negative serum pregnancy test within 7 days of the first dose of abemaciclib and agree to use a highly effective contraception method during the treatment period and for 3 weeks following the last dose of abemaciclib.
- Contraceptive methods may include an intrauterine device [IUD] or barrier method. If condoms are used as a barrier method, a spermicidal agent should be added as a double barrier protection.
- Cases of pregnancy that occur during maternal exposures to abemaciclib should be reported. If a patient or spouse/partner is determined to be pregnant following abemaciclib initiation, she must discontinue treatment immediately. Data on fetal outcome and breast-feeding are to be collected for regulatory reporting and drug safety evaluation.

Drug interactions:

Vismodegib and GSK2256098 are substrates of P-glycoprotein (PgP). The clinical significance of any drug interaction is unknown to date. The risk that AZD5363 will cause drug-drug interactions with substrates of P-gp is low.

Drugs that alter the pH of the upper GI tract (e.g. proton pump inhibitors, H2-receptor antagonists, and antacids) may alter the solubility of vismodegib and reduce its bioavailability. Co-administration with a proton pump inhibitor, H2-receptor antagonist or antacid, systemic exposure of vismodegib may be decreased and the effect on efficacy is unknown to date.

See also Section 8.1 for other ancillary care and potential interactions for GSK2256098, vismodegib, and AZD5363.

3.2 Pre-Registration Eligibility Criteria

Use the spaces provided to confirm a patient's eligibility by indicating Yes or No as appropriate. It is not required to complete or submit the following pages.

When calculating days of tests and measurements, the day a test or measurement is done is considered Day 0. Therefore, if a test were done on a Monday, the Monday four weeks later would be considered Day 28.

___ **3.2.1 Tissue available for central pathology review and biomarker testing**

This review is mandatory prior to registration to confirm eligibility.

Patients must have local diagnosis of meningioma (any grade) and have FFPE tumor block OR meningioma tissue slides available for submission for central pathology review and

biomarker testing by MGH/DFCI (a CLIA-certified lab). This review is mandatory prior to registration to confirm eligibility. See Section 6.2 for details on slide/block submission.

3.3 Registration Eligibility Criteria

___ 3.3.1 Documentation of Disease

Histologic Documentation: Histologically proven intracranial meningioma as documented by central pathology review.

Molecular Documentation: Presence of SMO, PTCH1, NF2, CDKN2A, AKT1, PIK3CA, PTEN mutations, CDKN2A copy number loss, CDK4, CDK6, CCND1, CCND2, CCND3, or CCNE1 copy number gain in tumor sample as documented specifically by the central laboratory, regardless of whether prior genotype testing outside of the central laboratory was performed. See Sections 4.4, 4.5 and Appendix VIII for further details.

Progressive OR residual disease, as defined by the following:

- **Residual measurable disease (**see also Section 3.3.2**):** Residual measurable disease immediately after surgery without requirement for progression. For Grade I disease, progression pre-operatively needs to be documented, with an increase in size of the measurable primary lesion on imaging by 25% or more (bidirectional area). The change must occur between scans separated by no more than 14 months. For patients with SMO/PTCH1 mutations enrolling to receive vismodegib, the change can occur between scans separated by up to 25 months. Residual measurable disease will be defined by bidimensionally measurable lesions with clearly defined margins by MRI scans, with a minimum diameter of 10mm in both dimensions. See Section 11.2.
- **Progressive measurable disease (**see also Section 3.3.2**):** Progression defined as an increase in size of the measurable primary lesion on imaging by 25% or more (bidirectional area). The change must occur between scans separated by no more than 12 months.
- **Post radiation patients**: Patients with measurable and progressive meningioma who have received radiation are potentially eligible, but need to show evidence of progressive disease after completion of radiation. If the progressive meningioma lesion has been radiated, at least 24 weeks must have elapsed from completion of radiation to registration. (See Section 3.3.3). If the progressive lesion is outside of the radiation field, then an interval of at least 2 weeks must have elapsed from completion of radiation to registration.

___ 3.3.2 Measurable disease

Measurable disease is defined by a bidimensionally measurable main lesion on MRI or CT images (MRI preferred) with clearly defined margins. Multifocal disease is allowed.

For measurable disease, refer to Section 11.0.

___ 3.3.3 Prior Treatment

- Prior medical therapy is allowed but not required.
- No limit on number of prior therapies.
- No chemotherapy, or other investigational agents within 28 days prior to registration.
- No other concurrent investigational agents or other meningioma-directed therapy (chemotherapy, radiation) while on study. Additionally, no cases of nitrosourea or mitomycin C within 6 weeks prior to registration.

- For patients treated with external beam radiation, interstitial brachytherapy or radiosurgery, an interval > 4 weeks must have elapsed from completion of radiation treatment to registration. If the progressive lesion is outside of the radiation field, then an interval of at least 2 weeks must have elapsed from completion of radiation to registration. (See 3.3.1).
- Steroid dosing stable for at least 4 days.
- Recovered to CTCAE grade 1 or less toxicity from other agents with exception of alopecia and fatigue.
- No craniotomy 28 days prior to and after registration.

___ **3.3.4 Not pregnant and not nursing**

A female of childbearing potential is a sexually mature female who: 1) has not undergone a hysterectomy or bilateral oophorectomy; or 2) has not been naturally postmenopausal for at least 12 consecutive months (i.e., has had menses at any time in the preceding 12 consecutive months). Please reference Section 5.0 (Study calendar) for details on pregnancy monitoring during the duration of the trial. **Also refer to Section 3.1 for agent-specific reproductive considerations and contraceptive requirements**.

___ **3.3.5 For patients with NF2/CDKN2A/AKT1/PIK3CA/PTEN mutation, CKDN2A copy number loss, or CDK4/CDK6/CCND1/CCND2/CCND3/CCNE1 copy number gain:** Age ≥ 18 years

For patients with SMO/PTCH1 mutation: Age ≥ 30 years

___ **3.3.6 ECOG Performance Status ≤ 2**

___ **3.3.7 Patient history:**

- Patients with history of NF may have other stable CNS tumors (schwannoma, acoustic neuroma or ependymoma) if lesions have been stable for 6 months.
- No metastatic meningiomas (as defined by extracranial meningiomas outside of CNS) allowed. Spinal meningiomas are allowed.
- No history of allergic reactions attributed to compounds of similar or biologic composition to assigned study drug.
- No known active hepatitis B or C
- No current Child Pugh Class B or C liver disease
- No uncontrolled gastric ulcer disease (Grade 3 gastric ulcer disease within 28 days of registration)
- No uncontrolled hypertension defined as BP > 140/90
- No abdominal fistula, GI perforation, or intra-abdominal abscess within 28 days prior to registration
- No major surgery within 28 days prior to registration for any patients with AKT1/PIK3CA/PTEN mutations receiving AZD5363.
- For patients going on to receive AZD5363 (i.e. enrolled after Update #08)

  Patients should not have any of the following cardiac criteria:
  - Any clinically important abnormalities in rhythm, conduction, or morphology of resting EKG (e.g., complete left bundle branch block, third degree heart block).
  - Any factors that increase the risk of QTc prolongation or risk of arrhythmic events such as heart failure, hypokalemia, potential for Torsade de Pointes, congenital long QT syndrome, family history of long QT syndrome, or

unexplained sudden death under 40 years of age, or any concomitant medication known to prolong the QT interval

- o Experience any of the following procedures or conditions in the preceding 6 months: coronary artery bypass graft, angioplasty, vascular stent, myocardial infarction, angina pectoris, congestive heart failure NYHA Class ≥ II.
- o Uncontrolled hypertension (SBP < 90 mmHg and/or DBP < 50 mmHg).
- o Cardiac injection fraction outside institutional range of normal or < 50% (whichever is higher) as measured by echocardiogram (or MUGA scan if an echocardiogram can’t be performed or is inconclusive). LVEF below lower limit of normal for site.

Patients should not have any of the following criteria

- o With the exception of alopecia, any unresolved toxicities from prior therapy greater than CTCAE grade 1 at the time of registration.
- o Hemoglobin < 9 g/dL (<5.59 mmol/L). Note: any blood transfusion must be ≥ 14 days prior to the determination of a hemoglobin ≥ 9 g/dL (≥5.59 mmol/L).
- o Proteinuria 3+ on dipstick analysis or > 500 mg/24 hours
- o Refractory nausea and vomiting, chronic gastrointestinal diseases, inability to swallow the formulated product or previous significant bowel resection that would preclude adequate absorption of AZD5363.
- o History of hypersensitivity to active or inactive excipients of AZD5363 or drugs with a similar chemical structure or class to AZD5363.
- o Current disease or condition known to interfere with absorption, distribution, metabolism, or excretion of drugs.
- o Past medical history of interstitial lung disease, drug-induced insterstitial lung disease, radiation pneumonitis which required steroid treatment, or any evidence of clinically active interstitial lung disease.
- o Previous allogeneic bone marrow transplant.
- o Known immunodeficiency syndrome.

___ **3.3.8 Concomitant medications (Only regarding NF2/CDKN2A/CDK4/CDK6/CCND1/CCND2/CCND3/CCNE1/AKT1/PIK3CA/PTEN genetic alterations)**

- Chronic concomitant treatment with strong inhibitors of CYP3A4 inhibitors must discontinue the drug for 14 days prior to registration on the study for patients with NF2 mutation enrolled to GSK2256098, as well as for patients with AKT1/PIK3CA/PTEN mutations enrolled to AZD5363. See Section 7.0 for more information.
- For NF2 patients going on to receive GSK2256098 and for patients with AKT1/PIK3CA/PTEN mutations enrolled to AZD5363: Concomitant treatment with strong CYP3A4 inducers or CYP2D6 substrates is not allowed. Patients must discontinue the drug 14 days prior to registration. See Section 7.0 for more information.
- For NF2 patients going on to receive abemaciclib: Avoid concomitant use of CYP3A inducers and strong CYP3A inhibitors. Use caution with coadministered moderate or weak CYP3A inhibitors. See Section 7.0 for more information.

___ **3.3.9 Diabetic status**

- **For patients with NF2 or SMO/PTCH1 mutations**: No uncontrolled diabetes defined as a known diabetic with HBA1C >7.5 OR fasting glucose > 140 mg/dL.
- **For patients with AKT1/PIK3CA/PTEN mutations**:
    - o Glycosylated hemoglobin (HbA1C) < 8.0% (63.9 mmol/mol)

- o No Type 1 diabetes mellitus
- o No requirement for insulin for routine diabetic management and control
- o No requirement for more than two oral hypoglycaemic medications for routine diabetic management and control
- o Patients with a pre-existing diagnosis of Type 2 diabetes mellitus must have fasting glucose < 9.3 mmol/L (167mg/dL). Fasting is defined as no caloric intake for at least 8 hours.
- o Patients without a pre-existing diagnosis of Type 2 diabetes mellitus must have fasting glucose ≥ 7.0 mmol/L (126 mg/dL). Fasting is defined as no caloric intake for at least 8 hours.

___ **3.3.10 Required Initial Laboratory Values:**

Absolute Neutrophil Count (ANC)	≥ 1,500/mm^3
Platelet Count	≥ 100,000/mm^3
Creatinine OR	≤ 1.5 mg/dl x ULN OR
Calc. Creatinine Clearance	> 50 mL/min
UPC	≤ 45mg/mmol
Total Bilirubin	≤ 1.5 x ULN*
AST / ALT	≤ 2.5 x ULN
Sodium, Potassium, Magnesium, Total Calcium (corrected for serum albumin) & Phosphorus	Within normal limits per institutional guidelines
QTcF**	< 450 msec
Mean Resting Heart Rate (determined from EKG)	50-100 BPM ***

* Except in case of Gilbert's disease

** QT calculated using Fridericia formula: QTc = QT/(RR^0.33), where RR = 60/HR.

*** Must be obtained from 12-lead EKG defined by a triplicate EKG for patients assigned to the AZD5363 arm. Patients assigned to all other arms will require a single EKG.

___ **3.3.11 Comorbid Conditions**

No uncontrolled medical comorbidities per investigator discretion (e.g. interstitial lung disease, severe dyspnea at rest or requiring oxygen therapy, history of major surgical resection involving the stomach or small bowel, or pre-existing Crohn's disease or ulcerative colitis or a preexisting chronic condition resulting in baseline Grade 2 or higher diarrhea).

3.4 Additional Registration Eligibility Criteria for Abemaciclib Arm

3.4.1 Hemoglobin ≥ 8 g/dL

Patients may receive erythrocyte transfusions to achieve this hemoglobin level at the discretion of the investigator. Initial treatment must not begin earlier than the day after the erythrocyte transfusion.

3.4.2 Prior Treatment

Patients who received chemotherapy must have recovered (Common Terminology Criteria for Adverse Events [CTCAE] Grade ≤1) from the acute effects of chemotherapy except for residual alopecia or Grade 2 peripheral neuropathy prior to registration. A washout period

of at least 28 days is required between last chemotherapy dose and registration (provided the patient did not receive radiotherapy).

Patients who received adjuvant radiotherapy must have completed and fully recovered from the acute effects of radiotherapy. A washout period of at least 28 days is required between end of radiotherapy and registration.

3.4.3 No active bacterial infection (requiring intravenous [IV] antibiotics at time of initiating study treatment), fungal infection, or detectable viral infection (such as known human immunodeficiency virus positivity or with known active hepatitis B or C [for example, hepatitis B surface antigen positive]). Screening is not required for enrollment in the absence of symptoms.

3.4.4 No personal history of any of the following conditions: syncope of cardiovascular etiology, ventricular arrhythmia of pathological origin (including, but not limited to, ventricular tachycardia and ventricular fibrillation), or sudden cardiac arrest.

4.0 PATIENT REGISTRATION

4.1 CTEP Registration Procedures

Food and Drug Administration (FDA) regulations and National Cancer Institute (NCI) policy require all individuals contributing to NCI-sponsored trials to register and to renew their registration annually. To register, all individuals must obtain a Cancer Therapy Evaluation Program (CTEP) Identity and Access Management (IAM) account at https://ctepcore.nci.nih.gov/iam. In addition, persons with a registration type of Investigator (IVR), Non-Physician Investigator (NPIVR), or Associate Plus (AP) (i.e., clinical site staff requiring write access to OPEN, Rave, or acting as a primary site contact) must complete their annual registration using CTEP's web-based Registration and Credential Repository (RCR) at https://ctepcore.nci.nih.gov/rcr.

RCR utilizes five person registration types.

- IVR — MD, DO, or international equivalent;
- NPIVR — advanced practice providers (e.g., NP or PA) or graduate level researchers (e.g., PhD);
- AP — clinical site staff (e.g., RN or CRA) with data entry access to CTSU applications (e.g., Roster Update Management System (RUMS), OPEN, Rave,);
- Associate (A) — other clinical site staff involved in the conduct of NCI-sponsored trials; and
- Associate Basic (AB) — individuals (e.g., pharmaceutical company employees) with limited access to NCI-supported systems.

RCR requires the following registration documents:

Documentation Required	IVR	NPIVR	AP	A	AB
FDA Form 1572	✓	✓			
Financial Disclosure Form	✓	✓	✓		
NCI Biosketch (education, training, employment, license, and certification)	✓	✓	✓		
GCP training	✓	✓	✓		
Agent Shipment Form (if applicable)	✓				
CV (optional)	✓	✓	✓		

An active CTEP-IAM user account and appropriate RCR registration is required to access all CTEP and Cancer Trials Support Unit (CTSU) websites and applications. In addition, IVRs and NPIVRs must list all clinical practice sites and Institutional Review Boards (IRBs) covering their practice sites on the FDA Form 1572 in RCR to allow the following:

- Addition to a site roster;
- Assign the treating, credit, consenting, or drug shipment (IVR only) tasks in OPEN;
- Act as the site-protocol Principal Investigator (PI) on the IRB approval; and
- Assign the Clinical Investigator (CI) role on the Delegation of Tasks Log (DTL).

In addition, all investigators act as the Site-Protocol PI, consenting/treating/drug shipment, or as the CI on the DTL must be rostered at the enrolling site with a participating organization (i.e., Alliance).

Additional information is located on the CTEP website at https://ctep.cancer.gov/investigatorResources/default.htm. For questions, please contact the **RCR Help Desk** by email at RCRHelpDesk@nih.gov.

4.2 CTSU Site Registration Procedures

This study is supported by the NCI CTSU.

IRB Approval

For CTEP and Division of Cancer Prevention (DCP) studies open to the National Clinical Trials Network (NCTN) and NCI Community Oncology Research Program (NCORP) Research Bases after March 1, 2019, all U.S.-based sites must be members of the NCI Central Institutional Review Board (NCI CIRB). In addition, U.S.-based sites must accept the NCI CIRB review to activate new studies at the site after March 1, 2019. Local IRB review will continue to be accepted for studies that are not reviewed by the CIRB, or if the study was previously open at the site under the local IRB. International sites should continue to submit Research Ethics Board (REB) approval to the CTSU Regulatory Office following country-specific regulations.

Sites participating with the NCI CIRB must submit the Study Specific Worksheet for Local Context (SSW) to the CIRB using IRBManager to indicate their intent to open the study locally. The NCI CIRB's approval of the SSW is automatically communicated to the CTSU Regulatory Office, but sites are required to contact the CTSU Regulatory Office at CTSURegPref@ctsu.coccg.org to establish site preferences for applying NCI CIRB approvals across their Signatory Network. Site preferences can be set at the network or protocol level.

Questions about establishing site preferences can be addressed to the CTSU Regulatory Office by emailing the email address above or calling 1-888-651-CTSU (2878).

Sites using their local IRB or REB, must submit their approval to the CTSU Regulatory Office using the Regulatory Submission Portal located in the Regulatory section of the CTSU website. Acceptable documentation of local IRB/REB approval includes:

- Local IRB documentation;
- IRB-signed CTSU IRB Certification Form; and/or
- Protocol of Human Subjects Assurance Identification/IRB Certification/Declaration of Exemption Form.

In addition, the Site-Protocol Principal Investigator (PI) (i.e. the investigator on the IRB/REB approval) must meet the following criteria to complete processing of the IRB/REB approval record:

- Holds an Active CTEP status;
- Rostered at the site on the IRB/REB approval and on at least one participating roster;
- If using NCI CIRB, rostered on the NCI CIRB Signatory record;
- Includes the IRB number of the IRB providing approval in the Form FDA 1572 in the RCR profile; and
- Holds the appropriate CTEP registration type for the protocol.

Additional Requirements
Additional requirements to obtain an approved site registration status include:

- An active Federal Wide Assurance (FWA) number;
- An active roster affiliation with the Lead Protocol Organization (LPO) or a Participating Organization (PO); and
- Compliance with all protocol-specific requirements (PSRs).

4.2.1 Downloading Site Registration Documents

Download the site registration forms from the protocol-specific page located on the CTSU members' website. Permission to view and download this protocol and its supporting documents is restricted based on person and site roster assignment. To participate, the institution and its associated investigators and staff must be associated with the LPO or a PO on the protocol.

- Log on to the CTSU members' website (https://www.ctsu.org) using your CTEP-IAM username and password;
- Click on *Protocols* in the upper left of your screen
  - o Enter the protocol number in the search field at the top of the protocol tree, or
  - o Click on the By Lead Organization folder to expand, then select Alliance, and protocol number A071801.
- Click on *Documents*, select *Site Registration*, and download and complete the forms provided. (Note: For sites under the CIRB initiative, IRB data will load automatically to the CTSU as described above.)

4.2.2 Requirements for A071401 Site Registration

- IRB approval (For sites not participating via the NCI CIRB; local IRB documentation, an IRB-signed CTSU IRB Certification Form, Protocol of Human Subjects Assurance Identification/IRB Certification/Declaration of Exemption Form, or combination is accepted)

4.2.3 Submitting Regulatory Requirements

- Submit required forms and documents to the CTSU Regulatory Office via the Regulatory Submission Portal on the CTSU website.
- To access the Regulatory Submission Portal log on to the CTSU members' website → Regulatory → Regulatory Submission.
- Institutions with patients waiting that are unable to use the Regulatory Submission Portal should alert the CTSU Regulatory Office immediately at 1-866-651-2878 in order to receive further instruction and support.

4.2.4 Checking Your Site's Registration Status

You can verify your site's registration status on the members' side of the CTSU website.

- Log on to the CTSU members' website;
- Click on *Regulatory* at the top of your screen;
- Click on *Site Registration;*
- Enter your 5-character CTEP Institution Code and click on Go.

Note: The status shown only reflects institutional compliance with site registration requirements as outlined above. It does not reflect compliance with protocol requirements for individuals participating on the protocol or the enrolling investigator's status with the NCI or their affiliated networks.

4.3 Patient Pre-Registration Requirements

- **Informed consent:** the patient must be aware of the neoplastic nature of his/her disease and willingly consent after being informed of the procedure to be followed, the experimental nature of the therapy, alternatives, potential benefits, side-effects, risks, and discomforts. Current human protection committee approval of this protocol and a consent form is required prior to patient consent and registration.
- **Cohort Status and Accrual:** Prior to discussing protocol entry with prospective patients, site staff must go to the A071401 study page on the CTSU web site to check cohort status and accrual. Site staff should also take into account any local diagnosis and biomarker results as they consider patients for study pre-registration. Contact the protocol coordinator and study chair for any questions about cohort status and accrual.
- **Central pathology review and central molecular laboratory submission:** Patients must have tissue available for analysis in order to be pre-registered for this study. Patients may be pre-registered to this study on the basis of the diagnosis of recurrent or progressive meningioma made at the original institutions. ALL diagnostic H&E slides must be submitted together with 15-25 unstained slides cut from the FFPE block that contains representative tumor tissue. (See Section 6.2).

  Submission of these samples for central pathology review and for biomarker testing is MANDATORY for all patients pre-registered to this study.

Tissue submission must be accompanied by a completed "Central Pathology and Biomarker Results Form" found on the A071401 study page. **Failure to submit this form with the specimens will delay turnaround time for central review and biomarker testing.** The specimen will be centrally reviewed to confirm study eligibility and cohort assignment based on AKT1, PIK3CA, PTEN, NF2, SMO, PTCH1, CDKN2A, CDK4, CDK6, CCND1, CCND2, CCND3, CCNE1 gene status. Sites must use this form to confirm eligibility and arm assignment.

4.4 Patient Registration Procedures

- **Confirmation of eligibility by central review:** Sites will be notified via e-mail whether or not the patient is eligible within 21 days of receipt of a suitable patient tumor specimen by the MGH Translational Research Laboratory. If a sample has insufficient tissue amount and/or tumor cellularity, a request for an alternative sample will be made. If a sample fails testing due to poor tissue (nucleic acid) quality, a request for a specimen from an alternative surgery will be made. Specimens will be routed as outlined in Section 6.2 and testing will be performed as outlined in Appendix VIII. Patient eligibility will be based on central biomarker testing results, central pathology review and cohort slot availability.
- **Upon the completion of testing**: The results section of the "Central Pathology and Biomarker Results Form" will be completed by the pathologist and laboratory, scanned and sent via e-mail to the responsible CRA listed on the form.
- **After receiving the results form via e-mail:** The institution must forward the form to the Alliance Patient Registration office at random01@mayo.edu in order to register the patient. Once the form is forwarded to the Alliance Patient Registration Office and the Registration Eligibility Criteria have been met, the patient can be registered using the OPEN system (see below). Please note: Once accrual to the patient cohort has been completed, no additional patients may be able to register (even if the patient is deemed eligible from central testing because a slot was available at that time). The Alliance Registration Office will communicate this back to the site.
- **Registration:** Registration must occur within 28 days of receiving notification of patient eligibility from the central testing laboratory. Please keep in mind that spots cannot be held or saved on cohorts, patient registration is on a first-come basis. The same patient ID number obtained at pre-registration from the OPEN system should be used to register the patient. Please contact Alliance Patient Registration office at random01@mayo.edu or 507-284-4130 if registration problems occur.

4.5 Patient Registration/Randomization Procedure

The Oncology Patient Enrollment Network (OPEN) is a web-based registration system available on a 24/7 basis. OPEN is integrated with CTSU regulatory and roster data and with the Lead Protocol Organization (LPOs) registration/randomization systems or Theradex Interactive Web Response System (IWRS) for retrieval of patient registration/randomization assignment. OPEN will populate the patient enrollment data in NCI's clinical data management system, Medidata Rave.

Requirements for OPEN access:

- A valid CTEP-IAM account;
- To perform enrollments or request slot reservations: Be on a LPO roster, ETCTN Corresponding roster, or PO roster with the role of Registrar. Registrars must hold a minimum of an AP registration type;

- If a Delegation of Tasks Log (DTL) is required for the study, the registrar(s) must hold the OPEN Registrar task on the DTL for the site; and
- Have an approved site registration for a protocol prior to patient enrollment.

To assign an Investigator (IVR) or Non-Physician Investigator (NPIVR) as the treating, crediting, consenting, drug shipment (IVR only), or receiving investigator for a patient transfer in OPEN, the IVR or NPIVR must list the IRB number used on the site's IRB approval on their Form FDA 1572 in RCR. If a DTL is required for the study, the IVR or NPIVR must be assigned the appropriate OPEN-related tasks on the DTL.

Prior to accessing OPEN, site staff should verify the following:

- Patient has met all eligibility criteria within the protocol stated timeframes; and
- All patients have signed an appropriate consent form and HIPAA authorization form (if applicable).

Note: The OPEN system will provide the site with a printable confirmation of registration and treatment information. Please print this confirmation for your records.

Access OPEN at https://open.ctsu.org or from the OPEN link on the CTSU members' website. Further instructional information is in the OPEN section of the CTSU website at https://www.ctsu.org or https://open.ctsu.org. For any additional questions, contact the CTSU Help Desk at 1-888-823-5923 or ctsucontact@westat.com.

4.6 Registration to Correlative and Companion Studies

4.6.1 Registration to Substudies described in Section 14.0

There are 2 substudies within Alliance A071401. These correlative science studies **must be offered to all patients** enrolled on Alliance A071401 (although patients may opt to not participate). These substudies do not require separate IRB approval. The substudies included within Alliance A071401 is/are:

- Identification of molecular and histological biomarkers of response, Alliance A071401-ST1 (Section 14.1.)
- Imaging biomarkers of response, Alliance A071401-IM1 (Section 14.2)

If a patient answers "yes" to "My samples and related information may be kept in a Biobank for use in future health research," they have consented to participate in the substudy described in Section 14.1. The patient should be registered to Alliance A071401-ST1 at the same time they are registered to the treatment trial (A071401). Samples should be submitted per Section 6.2.

If a patient answers "yes" to "I choose to take part in the imaging study and allow my MRI scans to be analyzed for the optional research study," they have consented to participate in the substudy described in Section 14.2. The patient should be registered to Alliance A071401-IM1 at the same time they are registered to the treatment trial (A071401). Imaging should be submitted per Section 6.3.

4.7 Treatment Assignments and Patient Cohorts

Patients will be assigned to an arm of the trial based on the mutation status. Tumors will be screened for the presence of AKT1, PIK3CA, PTEN, NF2, PTCH1, SMO, CDKN2A, CDK4,

CDK6, CCND1, CCND2, CCND3, CCNE1 genetic alterations and if present, they will be assigned to the single agent vismodegib, GSK 2256098, AZD5363, or abemaciclib. Within each arm, there will be two cohorts of patients decided by Grade status (grade II and III; grade I). There are accrual goals for each individual cohort within an arm, see Section 13.3 for further accrual information.

If a patient has more than 1 mutation present, they must be enrolled to the least common mutation (NF2 is most common, SMO is least common). Contact the protocol coordinator and study chair prior to registration to confirm which arm should be selected for patient registration. Upon discontinuation of study agent, the patient will be permitted to enroll to the study with the agent matching the other mutation. Sites must contact the protocol coordinator and study chair prior to crossover/re-registration to confirm that study drug is available and to coordinate re-registration through the Alliance Registration Office.

Specific Treatment Groups:

- Group 1: NF2 mutation, Grade I – GSK2256098
  (Closed to new patient enrollment on 7/19/2017)
- Group 2: NF2 mutation, Grade II/III – GSK2256098
  (Closed to new patient enrollment on 7/19/2017)
- Group 3: SMO/PTCH1 mutation, Grade I – vismodegib
- Group 4: SMO/PTCH1 mutation, Grade II/III – vismodegib
- Group 5: AKT1/PIK3CA/PTEN mutation, Grade I – AZD5363
- Group 6: AKT1/PIK3CA/PTEN mutation, Grade II/III – AZD5363
- Group 7: NF2 mutation/CDKN2A copy number loss/CDK4, CDK6, CCND1, CCND2, CCND3, or CCNE1 copy number gain – abemaciclib

5.0 STUDY CALENDAR

Laboratory and clinical parameters during treatment are to be followed using individual institutional guidelines and the best clinical judgment of the responsible physician. It is expected that patients on this study will be cared for by physicians experienced in the treatment and supportive care of patients on this trial.

Pre-Study Testing Intervals

- To be completed ≤ 16 DAYS before registration: All laboratory studies, history and physical.
- To be completed < 28 DAYS before registration: Any scan which is utilized for tumor measurement per protocol.
- To be completed ≤ 42 DAYS before registration: Any baseline exams used for screening, which is not utilized for tumor measurement.

	Prior to Registration*	Day 1 of each cycle (cycle is 28 days)*	Post treatment follow up**	At PD, withdrawal, or removal***
**Tests & Observations**				
History and physical, weight, PS	X	X	X	
Height	X			
Pulse, Blood Pressure	X	X		
Adverse Event Assessment	X(1)	X(1)	X(1)	
Patient Medication Diary		X(2)	X(2)	
Registration Fatigue/Uniscale Assessment	X(3)			
ECHO/MUGA	A	A		
EKG	B	B	B	
**Laboratory Studies**				
Complete Blood Count, Differential, Platelets	X	X(9)	E	
Chemistry (Creatinine, AST, ALT, Alk. Phos., Bili, glucose) HbA1c (if required)	X(4)	X(4,9)	E	
Urine Protein	X(5)	X(5)		
Serum or Urine HCG	X(6)	X(6)		
Serologic Hepatitis B Surface Ag and Hepatitis C RNA (physician discretion, not required)	X			
Fasting cholesterol, triglycerides	C	C		
Electrolytes (Na, K, Mg, Ca [corrected for serum albumin], P,)	X	PRN	PRN	PRN
**Staging**				
Central review for eligibility (pathology and molecular)	X(7)			

MRI/CT Brain	X(8)	D(8)	D(8)	X(8)
**Correlative studies: For patients who consent to participate**				
Tissue and Blood samples	Archival tissue at baseline for banking and correlative science. Blood samples every 4 cycles, and at recurrence/progression. Tissue upon recurrence/progression, see Sections 6.2			
MR Imaging	DCE MRI imaging should be performed at sites with such capability. DCE MRI will be acquired as part of routine clinical imaging and would not be an extra set of images. See "MRI/CT Brain" under "Staging." See Section 14.2 and Appendix III.			

* Labs completed prior to registration may be used for day 1 of cycle 1 tests if obtained ≤ 16 days prior to treatment. For subsequent cycles, labs, tests and observations may be obtained +/- 3 days from scheduled day of assessment. Radiographic windows are +/- 7 days from scheduled day of assessment.

** Physical examination, adverse event assessment, and medication diary are required 4 weeks (+/- 7 days) after the end of treatment.

*** Patients are followed for survival every 6 months, for a maximum of 5 years from registration. Patients discontinuing for reasons other than progressive disease will have staging scans every 16 weeks (+/- 4 weeks) until they have reached 2 years post-registration or until documented progression. See also Section 12.

1 Solicited AEs are to be collected starting at baseline. Routine AEs are to be collected starting after registration. See Section 9.3 for expedited reporting of SAEs.

2 Medication diary should be completed by the patient throughout treatment, and should be collected at day 1 of every cycle starting with day 1 cycle 2. Use the appropriate appendix. See Appendix IV.

3 To be completed after pre-registration and ≤ 21 days prior to treatment, see Appendix I.

4 For patients with AKT1/PIK3CA/PTEN mutation, fasting glucose and glycosylated hemoglobin are required at baseline, and at Cycles 1 and 2 with fasting glucose at pre-dose and no fasting glucose at 4 hours post dose. Further glucose monitoring may be performed in Cycle 3 onwards based on patient results during Cycles 1 and 2 at the Investigator's discretion. Please see Appendix X for the suggested algorithm for glucose management with metformin. Glycosylated hemoglobin is then required every 12 weeks during treatment.

5 Required only for patients with NF2 mutation enrolling/enrolled on GSK2256098

6 For women of childbearing potential (see Sections 3.1 and 3.3.4). Must be done ≤ 7 days prior to registration for all patients, and ≤ 7 days prior to initiation of vismodegib for patients with *SMO* mutation, and ≤ 7 days prior to initiation of GSK 2256098 for patients with NF2 mutation, and ≤ 7 days prior to initiation of abemaciclib for patients with *NF2* mutation. While on vismodegib, WOCP must continue to receive pregnancy tests on day 1 of every cycle (+/- 3 days). For patients on AZD5363, required on Cycle 1, Day1 and at treatment discontinuation.

7 See Sections 4.4 and 6.2 for central review submission.

8 Scans can include either: 1) MRI Brain or 2) CT Brain. The CT evaluation option should ONLY be used for patients unable to undergo MR imaging because of non-compatible device. Also see Section 11.0. Supporting documentation is to be submitted, per Section 6.1.1. All MRIs must be submitted to the Imaging Core Laboratory within 6 months of acquisition (see Section 6.3). Images must be submitted from baseline to (and including) progression. For patients who go off study for reasons other than progression (i.e., toxicity) please continue to submit images until progression. All MRIs should follow the consensus MRI protocol outlined in Appendix II, unless the patient has consented to the optional DCE substudy, in which case all MRIs should follow the MRI protocol outlined in Appendix III.

9 For the abemaciclib arm: every 2 weeks for the first 2 months, monthly for the next 2 months, and as clinically indicated.

A Required only for patients enrolling/enrolled on AZD5363. For patients on AZD5363, required prior to registration, on cycle 4, and at discontinuation, withdrawal or removal.

B For patients with NF2 mutation enrolling/enrolled to receive GSK2256098, EKG must be performed at 2 time points: within 28 days prior to registration, and 1 hour after taking the first dose of GSK2256098. For patients with NF2 mutation enrolling/enrolled on abemaciclib: ECG should be performed if clinically indicated prior to registration, every cycle from the start of treatment, and post-treatment follow up. For patients enrolled on AZD5363, a triplicate EKG is required prior to registration.

C Required at baseline and every 6 cycles thereafter for patients with NF2 mutation enrolling/enrolled on GSK2256098. Required every 12 weeks for patients with AKT1/PIK3CA/PTEN mutations enrolled receiving AZD5363.

D Every 8 weeks (e.g. prior to Cycle 3 Day 1) for 1 year. Scans may be done within +/- 7 days of a scheduled time point. **Response assessment should include assessment of all sites of disease and use the same imaging method as was used at baseline.**

E Required for abemaciclib arm only.

6.0 DATA AND SPECIMEN SUBMISSION

6.1 Data Collection and Submission

Medidata Rave is a clinical data management system being used for data collection for this trial/study. Access to the trial in Rave is controlled through the CTEP-IAM system and role assignments. To access Rave via iMedidata:

- Site staff will need to be registered with CTEP and have a valid and active CTEP-IAM account; and
- Assigned one of the following Rave roles on the relevant Lead Protocol Organization (LPO) or Participating Organization roster at the enrolling site: Rave CRA, Rave Read Only, Rave CRA (LabAdmin), Rave SLA, or Rave Investigator. Refer to https://ctep.cancer.gov/investigatorResources/default.htm for registration types and documentation required.
    - To hold Rave CRA or Rave CRA (Lab Admin) role, site staff must hold a minimum of an AP registration type;
    - To hold Rave Investigator role, the individual must be registered as an NPIVR or IVR; and
    - To hold Rave Read Only role, site staff must hold an Associates (A) registration type.

If the study has a Delegation of Tasks Log (DTL), individuals requiring write access to Rave must also be assigned the appropriate Rave tasks on the DTL.

Upon initial site registration approval for the study in Regulatory Support System (RSS), all persons with Rave roles assigned on the appropriate roster will be sent a study invitation e-mail from iMedidata. To accept the invitation, site staff must log in to the Select Login (https://login.imedidata.com/selectlogin) using their CTEP-IAM username and password, and click on the *accept* link in the upper right-corner of the iMedidata page. Site staff will not be able to access the study in Rave until all required Medidata and study specific trainings are completed. Trainings will be in the form of electronic learnings (eLearnings), and can be accessed by clicking on the link in the upper right pane of the iMedidata screen. If an eLearning is required and has not yet been taken, the link to the eLearning will appear under the study name in iMedidata instead of the *Rave EDC* link; once the successful completion of the eLearning has been recorded, access to the study in Rave will be granted, and a *Rave EDC* link will display under the study name.

Site staff that have not previously activated their iMedidata/Rave account at the time of initial site registration approval for the study in RSS will also receive a separate invitation from iMedidata to activate their account. Account activation instructions are located on the CTSU website in the Rave section under the Rave resource materials (Medidata Account Activation and Study Invitation Acceptance). Additional information on iMedidata/Rave is available on the CTSU members' website in the Data Management > Rave section at www.ctsu.org/RAVE/ or by contacting the CTSU Help Desk at 1-888-823-5923 or by e-mail at ctsucontact@westat.com.

6.1.1 Supporting documentation

This study requires supporting documentation for diagnosis, response and progression. Supporting documentation will include pathology, radiology, reports and these must be submitted at the following time points:

Baseline: Imaging report, pathology report, operative report, clinic note and Central Pathology and Biomarker Results Form

Response: Imaging report

Progression: Imaging report, and pathology report if applicable

Relapse/recurrence: Pathology report and operative report if applicable

6.1.2 Data Quality Portal

The Data Quality Portal (DQP) provides a central location for site staff to manage unanswered queries and form delinquencies, monitor data quality and timeliness, generate reports, and review metrics.

The DQP is located on the CTSU members' website under Data Management. The Rave Home section displays a table providing summary counts of Total Delinquencies and Total Queries. DQP Queries, DQP Delinquent Forms and the DQP Reports modules are available to access details and reports of unanswered queries, delinquent forms, and timeliness reports. Review the DQP modules on a regular basis to manage specified queries and delinquent forms.

The DQP is accessible by site staff that are rostered to a site and have access to the CTSU website. Staff that have Rave study access can access the Rave study data using a direct link on the DQP.

To learn more about DQP use and access, click on the Help icon displayed on the Rave Home, DQP Queries, and DQP Delinquent Forms modules.

Note: Some Rave protocols may not have delinquent form details or reports specified on the DQP. A protocol must have the Calendar functionality implemented in Rave by the Lead Protocol Organization (LPO) for delinquent form details and reports to be available on the DQP. Site staff should contact the LPO Data Manager for their protocol regarding questions about Rave Calendaring functionality.

6.2 Specimen collection and submission

For all patients registered to Alliance A071401:

Central pathology review will be conducted on the diagnostic H&E slides to confirm the diagnosis of meningioma (WHO grade I to III). In addition, integral molecular testing to evaluate the presence of an eligible gene mutation will be performed on a single FFPE tissue block from the surgery that contains representative tumor tissue. These samples, together with an "Alliance A071401 Central Pathology and Biomarker Results Form" with the top portion fully completed, along with a pathology report for the specimen submitted, will be required for integral biomarkers testing as part of eligibility determination (see Section 6.2.2 for details on sample submission). **Testing will not be initiated until all specimens and required forms are received.**

Dr. John Iafrate's laboratory at Massachusetts General Hospital will perform integral molecular testing on the tumor sections. Genetic alterations in SMO, PTCH1, NF2, AKT1, PIK3CA, PTEN, CDKN2A, CDK4, CDK6, CCND1, CCND2, CCND3, and CCNE1 will be used as key entry criteria for enrollment in this trial. If an eligible mutation is found, the H&E slides will be forwarded to Dr. Sandro Santagata at Brigham and Women's Hospital/Dana-Farber Cancer Center for central pathology review.

Results will be returned to the site within 21 days of complete specimen receipt at Massachusetts General Hospital.

For patients registered to substudy A071401-ST1:

All participating institutions must ask patients for their consent to participate in the correlative substudies planned for Alliance A071401-ST1, although patient participation is optional. Biomarker studies will be performed. Rationale and methods for the scientific components of these studies are described in Sections 14.2. For patients who consent to participate, tissue and blood will be collected at the following time points listed in the second half of the table below:

	≤ 120 days before registration	≤ 90 days after registration	Every 4 cycles during tx*	Recurrence/ Progression	Storage/ Shipping conditions	For collection instructions, see	Submit to:
**Mandatory for <u>all</u> patients registered to A071401: (parent study)**							
ALL diagnostic H&E slides from original diagnosis[1]	X				Ambient	Section 6.2.2	MGH
15-25 unstained slides of tumor tissue cut from a single paraffin block containing at least 1 cm^2 of representative and viable tumor tissue [1,2]	X				Ambient		MGH
**For patients registered to A071401-ST1**, submit the following: Optional**							
One diagnostic H&E slide from ***recurrent/progressive tumor*** and one paraffin block (or 15 slides) from ***recurrent/progressive tumor***[3]	N/A	X		X	Ambient	Section 6.2.3	Mayo FFPE
**Whole Blood** (EDTA/lavender top)[4]	1 x 10 mL				Cool pack/ship over night	Section 6.2.4	Mayo BAP
**Whole blood for ctDNA plasma** (EDTA/lavender top)[4]	2 x 10 mL		2 x 10 mL***	2 x 10 mL	Dry Ice/ship over night or freeze	Section 6.2.4	Mayo BAP

1 Submit as soon as possible after surgery and pre-registration. These specimens are required for central pathology review and integral biomarker testing. For patients with specimens from multiple timepoints, the most recent specimen should be submitted.

2 For patients who consent to having their specimens banked (model consent question # 4), residual specimen from central pathology review and integral molecular testing will be sent to the Alliance Biorepository at Mayo Clinic.

3 New biopsy is not required, only submit if surgery is performed.

4 Whole blood and whole blood for ctDNA plasma must be shipped SEPARATELY due to shipping conditions (cool pack vs. frozen/dry ice). See Section 6.2.4 for instructions.

* Prior to Cycles 5, 9, 13, 17, etc.

** For correlative studies and future correlative studies as described in Section 14.1.

*** For patients receiving AZD5363, whole blood for ctDNA plasma is to be collected just at pre-dose on Cycle 1 Day 1, and pre-dose Cycle 2 Day 1. For all other patients, ctDNA is to be collected once every four cycles while on treatment.

6.2.1 Specimen submission using the Alliance Biospecimen Management System

USE OF THE ALLIANCE BIOSPECIMEN MANAGEMENT SYSTEM (BioMS) IS MANDATORY AND ALL SPECIMENS MUST BE LOGGED AND SHIPPED VIA THIS SYSTEM.

BioMS is a web-based system for logging and tracking all biospecimens collected on Alliance trials. Authorized individuals may access BioMS at the following URL: http://bioms.allianceforclinicaltrialsinoncology.org using most standard web browsers (Safari, Firefox, Internet Explorer). For information on using the BioMS system, please refer to the 'Help' links on the BioMS webpage to access the on-line user manual, FAQs, and training videos. To report technical problems, such as login issues or application errors, please contact: 1-855-55-BIOMS or Bioms@alliancenctn.org. For assistance in using the application or questions or problems related to specific specimen logging, please contact: 1-855-55-BIOMS or Bioms@alliancenctn.org.

After logging collected specimens in BioMS, the system will create a shipping manifest. This shipping manifest must be printed and placed in the shipment container with the specimens.

All submitted specimens must be labeled with the protocol number (A071401), Alliance patient number, patient's initials and date and type of specimen collected (e.g., serum, whole blood).

A copy of the Shipment Packing Slip produced by BioMS must be printed and placed in the shipment with the specimens.

Instructions for the collection of samples are included below. Please be sure to use a method of shipping that is secure and traceable. Extreme heat precautions should be taken when necessary.

ALL tumor tissue for Central Pathology Review and Integral Biomarker Testing:

Shipment on Monday through Thursday by overnight service to assure timely receipt is encouraged. Do not ship specimens on Fridays or Saturdays or for receipt on national holidays.

John Iafrate, MD/PhD
c/o Nancy Higgins
Massachusetts General Hospital
55 Fruit Street, GRJ-1015
Boston, MA 02114

For questions about receipt of specimens or status of central review and biomarker testing contact:

Specimen Receipt & Biomarker Testing:
Nancy Higgins
Tel: 617-643-8651
MGHTRLClinicalTrials@partners.org

Central Pathology Review:
Fiona Watkinson
Tel: 617-632-5482
FionaJ_Watkinson@DFCI.HARVARD.EDU

Blood submission for patients who agree to participate:

Shipment on Monday through Friday by overnight service to assure receipt is encouraged. Do not ship specimens on Saturdays. Ship samples to the following address:

Alliance BAP Freezer
ST-SL-16
150 Third Street SW
Rochester, MN 55902

For questions about blood submission contact:
Roxann Neumann, RN, BSN, CCRP
Tel: 507-538-0602
neumann.roxann@mayo.edu

Tissue submission for the correlative studies for patients who agree to participate:

Alliance Biorepository at Mayo Clinic FFPE Tissue
Attn: PC Office (Study A071401)
RO-FF-03-24-CC/NW Clinic
200 First Street Southwest
Rochester, MN 55905

For questions about tissue submission to Mayo FFPE contact:
Helen Tollefson
Tel: 507-266-0724 Fax: 507-266-7240
tollefson.helen@mayo.edu

6.2.2 Mandatory diagnostic tumor tissue sample and H&E slide submission for central laboratory tumor genotyping and central pathology review

Integral molecular testing is being used to determine the presence or absence of an eligible genetic alteration in the patient's pre-existing diagnostic tumor sample. It is required as part of the process to determine patient eligibility for this clinical trial. This testing will be performed on sections of tumor tissue that have been obtained from this diagnostic FFPE tissue block at the participating site from which the original diagnosis of meningioma was made. If an eligible mutation is identified, the entire series of original H&E slides obtained from that tumor surgery will undergo Central Pathology Review to further establish patient eligibility. Therefore, tissue sections from a representative diagnostic tumor block and the full series of H&E slides from that surgery must be submitted together for eligibility determination.

Please prepare the following items for shipment that are MANDATORY for tumor testing and central pathology review.

1) From the site where the original diagnosis of meningioma was made, contact the pathology department and request that tissue sections be cut from a tumor block that contains representative meningioma tumor tissue from the primary disease site. Prepare the tumor sections as follows *(unsectioned tissue blocks* ***will not be accepted****):*

- A total of 15-25 tissue sections should be cut at a 5µM thickness and mounted separately onto a positively-charged glass slide.

  NOTE: The sample should ideally consist of at least 1 cm^2 of variable tumor. For samples containing <1 cm^2 of tumor tissue, 20-25 sections are requested in order to ensure that adequate tumor tissue is available for informative testing, thereby minimizing testing failures and maximizing the chance of identifying an eligible mutation for patient accrual. However, the site's Pathology Department will determine how many sections can be obtained without depleting the tumor sample and that amount will be accepted for testing submission.
- The tissue should be cut using a new blade and using a fresh water bath to avoid contamination.
- Mount all tissue sections in the same orientation onto positively-charged glass microscope slides.
- Do NOT bake the slides.
- When possible, label each slide with:
  - Trial # (A071401)
  - Patient trial ID #
  - Patient initials

  Note: Internal surgical numbers are acceptable only if recorded on the "Alliance A071401 Central Pathology and Biomarker Results Form".

2) Also request from the pathology department the full series of original H&E slides prepared from the surgery where diagnosis was made.
   - If the original slides are not available, new H&E slides must be prepared for submission. Tissue blocks or tissue sections will not be accepted as an H&E slide substitution.
3) Include the following items together in the shipment container **(Testing will not be initiated until all specimens and completed paperwork are received):**
   - The BioMS shipping manifest
   - A copy of the pathology report for the enclosed specimen. This report should be de-identified by obscuring all PHI (names and dates) with white-out or a black magic marker, **labeling each page of the report with the Alliance patient ID**, and photocopying the report.
   - An "Alliance A071401 Central Pathology and Biomarker Results Form" with the top portion full completed.
   - The full series of diagnostic H&E slides placed in crush proof containers.
   - The 15-25 mounted sections of primary meningioma tissue, clearly-labeled, placed in crush proof containers.
4) Contact Nancy Higgins via e-mail at MGHTRLClinicalTrials@partners.org before shipment. Please indicate the clinical trial #(A071401), the samples that are being sent, the patient identifiers, and the FedEx shipping number.
5) Ship specimens at ambient conditions, taking heat precautions when necessary.
6) Send by overnight courier (FedEx) on Monday through Thursday to the following address. Do not ship specimens on Fridays or Saturdays, or for delivery on major holidays.

John Iafrate, MD/PhD
c/o Nancy Higgins
Massachusetts General Hospital Cancer Center
55 Fruit Street, GRJ-1015
Boston, MA 02114

7) For questions about receipt of specimens or status of biomarker testing, contact:
Nancy Higgins
MGH Translational Research/Biomarker Laboratory
Program Coordinator
Tel: 617-643-8651
MGHTRLClinicalTrials@partners.org

The ~21 day turnaround time for completed testing does not start until all materials have been successfully received into the Massachusetts General Hospital Translational Research Laboratory and does not include national holidays.

Residual material from central pathology and biomarker review will be batch shipped every three to five months to the Alliance Biorepository at Mayo FFPE for storage.

6.2.3 Recurrent/progression tissue submission to Alliance Biorepository at Mayo FFPE (A071401-ST1)

If upon enrollment patient has recurrent disease, then after registration (Step 1), with the patient's consent, and, when available, 1 recurrent FFPE tissue block and 1 H&E slide obtained from tumor specimens should be sent to the Alliance Biorepository at Mayo Clinic FFPE. While on study, if patient progresses or tumor recurs, and surgery is performed, then 1 FFPE tissue block and 1 H&E slide obtained from recurrent/progressive tumor specimens should be submitted. A new biopsy is not required.

Please label the tumor specimens with

1) Alliance study number (A071401)

2) Alliance patient ID number

3) Patient's initials

4) Date and time of specimen procurement

The Alliance has instituted special considerations for the small percentage of hospitals whose policy prohibits long-term storage of blocks, and the smaller percentage of hospitals whose policies prohibit release of any block. For those hospitals for which tumor tissue block submission is not feasible, please submit 15 unstained slides from the recurrent tissue block (or as many slides as possible if fewer than 15). Five micron sections are preferred. Slides need to be cut with a new blade and using a fresh water bath to avoid contamination. Label the slides with Alliance study number (A071401), Alliance patient ID number, patient initials, accession number, and order of sections *(include thickness of section if applicable)***.**

Please contact the Alliance Biorepository at Mayo Clinic FFPE contact if additional assurances with your hospital pathology department are required.

6.2.4 Blood sample submission (A071401-ST1)

For patients who consent to participate in A071401-ST1, whole blood will be used for the biomarker analyses described in Section 14.1.2.

Please note the whole blood for germline DNA and whole blood for circulating tumor DNA (ctDNA) must be shipped separately as the shipping conditions are different (cool pack vs. dry ice/frozen, respectively).

Whole blood for germline DNA

- Collect one 10 mL of venous blood at pre-registration in lavender top (EDTA anticoagulant) vacutainer tube(s). The tubes should be inverted approximately 8-10 times to mix the EDTA. **Refrigerate (please do not freeze)** sample until shipping. The sample should be placed in a biohazard bag and shipped according to IATA guidelines the same day as the blood is drawn on a **cold pack** (**please do not use dry ice**) by overnight courier service to the Alliance BAP Freezer. Batch shipping is not allowed, please indicate on the packing slip whether the frozen samples will be sent right away, or shipped at a later date.
- Whole blood samples should be sent to Alliance Biorepository at Mayo BAP per Section 6.2.1.

Label samples with the following identification:

1) Procurement date
2) Alliance patient number
3) Alliance study number (i.e., A071401-ST1)
4). Patient initials
5) Sample type

Whole blood for circulating tumor DNA (ctDNA) plasma

- Collect 2 x 10 mL whole blood in lavender top (EDTA anticoagulant) vacutainer tube(s) at pre-registration, every four cycles on study treatment (i.e. prior to cycles 5, 9, 13, 17, etc.) and at recurrence/progression. Invert approximately 8-10 times to mix the EDTA.
- 1st Centrifugation step: centrifuge the two EDTA tubes at 1500g (or lab standard) for 15 minutes **at ambient temperature**. After centrifugation, three different fractions are distinguishable: the upper clear plasma layer, the intermediate buffy coat layer containing concentrated leukocytes and the bottom layer of red cells. Draw off the plasma layers from the two centrifuged tubes, minimizing removal of the intermediate buffy coat layers, and transfer the plasma to the 15 mL conical tube.
- 2nd centrifugation step: centrifuge the 15 mL conical tube at 1500g (or lab standard) for 15 min **at ambient temperature. After centrifugation,** transfer ~1.5ml of the upper clear plasma layer into each 2ml cryovial tubes (up to 8 vials) *.
- Freeze sample at -80 °C until shipping. The sample should be placed in a biohazard bag and shipped according to IATA guidelines on dry ice within 30 days of the blood draw by overnight courier service to the Alliance BAP Freezer.
    - Samples may be batch shipped as long as they are shipped within 30 days from blood draw. A separate BioMS packing list for each specimen must be kept with the samples. Please be sure to note on the packing list the date that the refrigerated samples were submitted.
    - If –80°C is not available, temporary storage on dry ice or at –20°C prior to shipment is acceptable for up to approximately 48 hours.
- Plasma samples in cryovials should be sent to Alliance Biorepository at Mayo BAP per Section 6.2.1 **Label** samples with the following identification:

1) Procurement date
2) Alliance patient number
3) Alliance study number (i.e., A071401-ST1)

4) Patient initials
5) Sample type (be sure to label sample with "PPP")

*Cryovial Choices: Some examples of acceptable 2.0 mL cryovials are: Nalgene (Cat #5012-0020), Fisher (Cat #05-669-57), Corning (Cat #430488), VWR (Cat #16001-102). For questions about acceptable cryovial choices, contact the Mayo BAP as outlined in Section 6.2.1.

6.3 CT and MR Imaging Data Submission

Acquisition of MR imaging in a uniform manner for all patients across the study and for an individual patient on serial imaging is critical information for this trial. We have defined the consensus imaging parameters that are required for imaging on 1.5 T and 3 T MRI scanners and sites should follow these parameters for all patients irrespective of whether or not they enroll in the optional advanced imaging substudy (see Appendix II for required consensus acquisition parameters).

All MRI images will be transmitted electronically from each participating site to the Imaging and Radiation Oncology Core QA center at Ohio State University (IROC Ohio). All study MRIs performed before the approval of Update #5 should be transmitted electronically to IROC.

For patients who consent to participate (model consent question #1), DCE MRI should be performed at sites with such capability. The DCE MRI acquisition protocol is outlined in Appendix III.

For all patients, complete data sets in digital DICOM format and submit to IROC Ohio. Images must be submitted from the following time points: baseline to progression. See Section 5.0. For patients who go off study for reasons other than progression (i.e., toxicity) please continue to collect images until progression. **Institutions are permitted to batch ship images every six months. Sites must turn in images within 180 days of acquisition to be compliant with data submission.** BMP files, JPG files, or hard copies (films) are not acceptable. The raw data of the entire study should be saved until the scan is accepted by the Imaging Core Lab. De-identify the patient data using institutional procedures to remove patient name and medical record number while preserving the Alliance patient ID number and protocol number. The de-identified digital images may be temporarily burned to a CD or transferred to a PC based system.

Data should be transferred **electronically** to the IROC Ohio via TRIAD, Web Transfer or FTP Transfer:

1) TRIAD based data transfer

The standard TRIAD based data transfer approach will be provided separately through IROC efforts per the request by participating sites before their first data submission.

Transfer of Images and Data (TRIAD) is the American College of Radiology's (ACR) image exchange application. TRIAD provides sites participating in clinical trials a secure method to transmit images. TRIAD anonymizes and validates the images as they are transferred.

TRIAD Access Requirements:
Site staff that will be submitting images via TRIAD will need to register with CTEP and have a valid and active CTEP-IAM account.

- Must be registered as an Associate, Associate Plus, Non-Physician Investigator, or Investigator registration type. Refer to the CTEP Registration Procedures section for instructions on how to request a CTEP-IAM account and complete registration in Registration and Credential Repository (RCR).

To submit images, site staff must hold the TRIAD Site User role on an NCTN or ETCTN roster. Individuals requiring a TRIAD Site User role should contact the person holding a primary role at the site for their affiliated NCTN or ETCTN roster.

All individuals on the Imaging and Radiation Oncology Core provider roster have access to TRIAD, and may submit images for credentialing purposes, or for enrollments to which the provider is linked in OPEN.

TRIAD Installation:
To submit images, the individual holding the TRIAD Site User role will need to install the TRIAD application on their workstation. TRIAD installation documentation is available at https://triadinstall.acr.org/triadclient/.

This process can be done in parallel to obtaining your CTEP-IAM account username and password and RCR registration.

For questions, contact TRIAD Technical Support staff via email TRIAD-Support@acr.org or 1-703-390-9858.

2) Web Transfer (http://upload.imagingcorelab.com**)**

Any PCs with internet access and web browser (e.g., Internet Explorer, Mozilla Firefox) can be used to web transfer DICOM images and other required files to the Imaging Core Lab. The standard Web Transfer information will be provided separately through the specific trial e-mail, per the request by participating sites before their first data submission.

3) FTP Transfer

Any FTP software can be used to initiate access to the secure FTP Server of the Imaging Core Laboratory. The standard FTP access information will be provided separately through the specific trial e-mail, per the request by participating sites before their first data submission.

Mail/CD Shipment

Only if electronic data transfer approaches cannot be achieved, the de-identified images in digital DICOM format can be burned to a CD and mailed to the Imaging Core Lab. Submit only one patient's images per CD, with the patient's Alliance ID number, study type, date of scans, and name of submitting institution.

Submit these data to:

IROC Ohio
Attn: Alliance Trial A071401
The Ohio State University
Wright Center of Innovation
395 W. 12th Avenue, Suite 414
Columbus, Ohio, 43210
Tel: 614-293-9151
Fax: 614/293-9275

Once the imaging data submission is done, send an e-mail to the Imaging Core Lab at the specific trial email Alliance071401@irocohio.org to inform that the study has been submitted from the institution. Please include the basic information of submitted data sets as follows:

1) Alliance patient ID number

2) Scan time point (i.e., baseline)

3) Date of scans

4) Institution name

IROC Ohio will acknowledge receipt of the imaging data via email confirmation to the institution within 1 business day of receipt, and will notify the institution and Alliance imaging committee of the quality check report within 3 business days.

7.0 TREATMENT PLAN/INTERVENTION

Protocol treatment is to begin ≤ 7 days of registration. For questions regarding treatment, please see the study contacts page.

It is acceptable for individual chemotherapy doses to be delivered ≤ a 24-hour (business day) window before and after the protocol-defined date for Day 1 of a new cycle. For example, if the treatment due date is a Friday, the window for treatment includes the preceding Thursday through the following Monday. In addition, patients are permitted to have a new cycle of chemotherapy delayed up to 7 days for major life events (e.g., serious illness in a family member, major holiday, vacation that cannot be rescheduled) without this being considered a protocol violation. Documentation to justify this delay should be provided.

Treatment will be administered as follows:

- In patients with *SMO*-mutated or *PTCH1*-mutated meningiomas, protocol therapy will consist of vismodegib administered every day.
- In patients with *NF2*-mutated meningiomas who enrolled prior to August 2017, protocol therapy will consist of GSK2256098 administered twice daily, every day.
- In patients with *AKT1*-mutated, *PIK3CA*-mutated or *PTEN*-mutated meningiomas, protocol therapy will consist of AZD5363 administered twice-daily for four days, and then no treatment for 3 days, repeated weekly for one cycle.
- In patients harboring alterations in the CDK pathway or in NF2 (after Update #9), a *CDK* inhibitor (abemaciclib) will be administered

For all arms, each cycle will consist of 28 days. Treatment will continue until disease progression or unacceptable adverse event. Dose modifications will be instituted for toxicity as per Section 8.0. See Section 5.0 for schedule of assessments, Section 11.0 for response criteria and Section 12.0 for duration of treatment and follow-up.

7.1 Arm A (SMO/PTCH1 mutation - Vismodegib)

Agent	Dose	Route	Frequency
Vismodegib	150 mg	PO	Once daily

If a dose of vismodegib is missed, do not make up that dose; resume dosing with the next scheduled dose. Capsules should not be opened or crushed.

Note: If a patient is suspected to be pregnant, GDC-0449 should be IMMEDIATELY discontinued and the study physician contacted. A positive urine test must be confirmed by a serum pregnancy test. If it is confirmed that the patients is not pregnant, the patient may resume dosing with GDC-0449.

7.2 Arm B (NF2 mutation – GSK2256098)

Agent	Dose	Route	Frequency
GSK2256098	750 mg	PO	Twice/daily

If a dose of GSK2256098 is missed, do not make up that dose; resume dosing with the next scheduled dose. Capsules should not be opened or crushed.

Please note all supply of GSK2256098 for this study expires by September 2019. Currently, there are no plans for further supply of GSK2256098 to be manufactured, see Section 10.2.

7.3 Arm C (AKT1/PIK3CA/PTEN mutation – AZD6363)

Agent	Dose	Route	Frequency
AZD5363	480 mg	PO	Twice/daily for 4 days, then 3 days off, repeated weekly

- If a dose of AZD5363 is missed, do not make up that dose; resume dosing with the next scheduled dose. Tablets should not be crushed.
- Where possible, all doses of AZD5363 should be taken, at approximately the same times each day, with water in a fasted state from at least 2 hours prior to the dose to at least 1 hour post-dose.
- In the event that a patient vomits, the patient must not retake new tablets(s), but continue to take the next dose 12 hours later.

7.4 Arm D (CDK4, CDK6, CDKN2A, CCND1, CCND2, CCND3, CCNE1 alterations-Abemaciclib)

Agent	Dose	Route	Frequency
Abemaciclib	200 mg	Oral	Every 12 hours

- Patients should be instructed to take the abemaciclib capsules at approximately the same time each day.
- If vomiting occurs during the course of treatment, no re-dosing of the patient is allowed before the next scheduled dose
- Any doses that are missed (not taken within 2 hours of the intended time) should be skipped and should not be replaced or made up on a subsequent day.

7.5 Important interaction information for patients receiving GSK2256098, AZD5363, and abemaciclib

7.5.1 CYP3A4 Inhibitors

Chronic concomitant treatment with strong inhibitors of CYP3A4 is not allowed on this trial for patients receiving GSK2256098, abemaciclib, or AZD5363. The following drugs are EXAMPLES of strong inhibitors of CYP3A4 and are not allowed during treatment with GSK2256098, abemaciclib, or AZD5363.

- Indinavir
- Clarithromycin
- Ketoconazole

Because lists of these agents are constantly changing, please consult and review any drugs for their potential to inhibit CYP3A4. Examples of resources that may be utilized include

the product information for the individual concomitant drug in question, medical reference texts such as the Physicians' Desk Reference, the FDA, or your local institution's pharmacist.

For abemaciclib arm: If coadministration with a strong CYP3A inhibitor is unavoidable, reduce the abemaciclib dose to 100 mg twice daily or, in the case of ketoconazole, reduce the abemaciclib dose to 50 mg twice daily. In patients who have had a dose reduction to 100 mg twice daily due to adverse reactions, further reduce the abemaciclib dose to 50 mg twice daily. Avoid grapefruit or grapefruit juice. If a CYP3A inhibitor is discontinued, increase the abemaciclib dose (after 3-5 half-lives of the inhibitor) to the dose that was used before starting the inhibitor.

A wallet-size card providing information regarding potential drug interactions has been made available in Appendix IX

7.5.2 CYP3A4 Inducers

Chronic concomitant treatment with strong inducers of CYP3A4 is not allowed on this trial for patients receiving GSK2256098, abemaciclib, or AZD5363. The following drugs are EXAMPLES of strong inducers of CYP3A4 and are not allowed during treatment with GSK2256098, abemaciclib, or AZD5363.

- Rifampin
- Carbamazepine

Because lists of these agents are constantly changing, please consult and review any drugs for their potential to induce CYP3A4. Examples of resources that may be utilized include the product information for the individual concomitant drug in question, medical reference texts such as the Physicians' Desk Reference, the FDA, or your local institution's pharmacist.

A wallet-size card providing information regarding potential drug interactions has been made available in Appendix IX .

7.5.3 CYP2D6 Inhibitors

Based on clinical data, AZD5363 is a moderate inhibitor of CYP2D6. Therefore, drugs mainly metabolized by or sensitive to inhibition of CYP2D6 should not be coadministered with AZD5363. Examples of CYP2D6 substrates include:

- Desipramine
- Dextromethorphan
- Nebivolol

Because lists of these agents are constantly changing, please consult and review any drugs for their potential to be metabolized by or sensitive to inhibition of CYP2D6. Examples of resources that may be utilized include the product information for the individual concomitant drug in question, medical reference texts such as the Physicians' Desk Reference, the FDA, or your local institution's pharmacist.

A wallet-size card providing information regarding potential drug interactions has been made available in Appendix IX .

8.0 DOSE AND TREATMENT MODIFICATIONS

8.1 Ancillary therapy, concomitant medications, and supportive care

8.1.1 Patients should not receive any other agent which would be considered treatment for the primary neoplasm or impact the primary endpoint.

8.1.2 **Patients should receive full supportive care while on this study.** This includes blood product support, antibiotic treatment, and treatment of other newly diagnosed or concurrent medical conditions. All blood products and concomitant medications such as antidiarrheals, analgesics, and/or antiemetics received from the first day of study treatment administration until 30 days after the final dose will be recorded in the medical records.

8.1.3 **Treatment with hormones** or other chemotherapeutic agents may not be administered except for steroids given for adrenal failure; hormones administered for non-disease-related conditions (e.g., insulin for diabetes); intermittent use of dexamethasone as an antiemetic or if significant symptomatic edema on brain MRI.

8.1.4 **Antiemetics** may be used at the discretion of the attending physician, with the exception of steroids above.

8.1.5 **Diarrhea** management is per the discretion of the treating physician. Diarrhea could be managed conservatively with medications such as loperamide.

Patients with severe diarrhea should be assessed for intravenous hydration and correction of electrolyte imbalances.

Diarrhea management with abemaciclib: In the event of diarrhea, supportive measures should be initiated as early as possible. These include the following:

- At the first sign of loose stools, the patient should initiate anti-diarrheal therapy (for example, loperamide) and notify the investigator/site for further instructions and appropriate follow-up.
- Patients should also be encouraged to drink fluids (for example, 8 to 10 glasses of clear liquids per day).
- Site personnel should assess response within 24 hours.
- If diarrhea does not resolve with anti-diarrheal therapy within 24 hours to either baseline or Grade 1, abemaciclib should be suspended until diarrhea is resolved to baseline or Grade 1.
- When abemaciclib recommences dosing should be adjusted as outlined below.
- In severe cases of diarrhea, the measuring of neutrophil counts and body temperature and proactive management of diarrhea with antidiarrheal agents should be considered. If diarrhea is severe (requiring IV rehydration) and/or associated with fever or severe neutropenia, broad-spectrum antibiotics such as fluoroquinolones must be prescribed. Patients with severe diarrhea or any grade of diarrhea associated with severe nausea or vomiting should be carefully monitored and given intravenous fluid (IV hydration) and electrolyte replacement.

8.1.6 **Palliative radiation therapy may not** be administered during study enrollment.

Patients who require radiation therapy during protocol treatment will be removed from protocol therapy due to disease progression.

8.1.7 **Rash:** Rash prevention measures should be utilized. This includes avoidance of UV exposure with covering of skin and sunscreen, and application of lotion. If rash occurs, consider dermatology consultation to aid management, and topical measures can be utilized.

For AZD5363 specifically, clinical experience indicates that rash can be managed with treatment as outlined in the rash management algorithm (e.g., use of oral or topical steroids, use of oral antihistamine), as well as by interruptions or reductions in AZD5363 dosing. The need for an interruption or dose reduction of AZD5363 should be considered with reference to the Rash Management Algorithm in Appendix X. Of note, AZD5363 showed no evidence of phototoxicity so no special precautions are needed by patients with regard to sun exposure or use of sunbeds/tanning booths. However, investigators will be asked to probe the possibility of phototoxicity when rash AEs are reported.

8.1.8 Surgery: Patients who require surgery during protocol treatment may proceed as such, unless the surgery involves resection of meningioma. Study agent should be held prior to and after surgery, for a maximum of 28 days. If the patient requires an interruption of > 28 days, then they will be removed from protocol therapy.

8.1.9 Hyperglycemia: Glucose profiles should be performed at the relevant time points to adequately characterize emergent hyperglycemia and to allow appropriate clinical management of patients. A suggested algorithm for the management of hyperglycemia is provided in Appendix X.

8.1.10 Hyperlipidemia: Patients taking GSK2256098 should be treated with appropriate medical therapy for hyperlipidemia and avoiding concomitant medications that interact with study medication.

8.1.11 Hypertension: Patients taking GSK2256098 should maintain well-controlled blood pressure, especially in the setting of proteinuria.

8.1.12 Alliance Policy Concerning the Use of Growth Factors

The following guidelines are applicable unless otherwise specified in the protocol.

Blood products and growth factors should be utilized as clinically warranted and following institutional policies and recommendations. The use of growth factors should follow published guidelines of the American Society of Clinical Oncology 2006 Update of Recommendations for the Use of White Blood Cell Growth Factors: An Evidence-Based, Clinical Practice Guideline. J Clin Oncol 24(19): 3187-3205, 2006.

Epoetin (EPO): Use of epoetin in this protocol is permitted at the discretion of the treating physician. The use of epoetin should follow published guidelines of the American Society of Clinical Oncology 2010 Update of Recommendations on the Use of Epoetin and Darbepoetin in Adult Patients with Cancer. J Clin Oncol 28(33): 4996-5010, 2010.

Due to concerns regarding the inherent toxicity of EPO and the investigational agents employed in the protocol, use of EPO is strongly discouraged.

Filgrastim (G-CSF), tbo-filgrastim and sargramostim (GM-CSF)

1. Filgrastim (G-CSF)/tbo-filgrastim/pegfilgrastim and sargramostim (GM-CSF) treatment for patients on protocols that do not specify their use is discouraged.
2. Filgrastim/tbo-filgrastim/pegfilgrastim and sargramostim may not be used:
   a. To avoid dose reductions, delays or to allow for dose escalations specified in the protocol.
   b. For the treatment of febrile neutropenia the use of CSFs should not be routinely instituted as an adjunct to appropriate antibiotic therapy. However, the use of CSFs may be indicated in patients who have prognostic factors that are predictive of clinical deterioration such as pneumonia, hypotension, multi-organ dysfunction

(sepsis syndrome) or fungal infection, as per the ASCO guidelines. Investigators should therefore use their own discretion in using the CSFs in this setting. The use of CSF (filgrastim/tbo-filgrastim/pegfilgrastim or sargramostim) must be documented and reported.

c. If filgrastim/tbo-filgrastim/pegfilgrastim or sargramostim are used, they must be obtained from commercial sources.

8.1.13 Hypersensitivity reactions

Treat hypersensitivity reactions as per institutional standards. In the case of hypersensitivity reactions, specifically to AZD5363, discontinue and symptomatic/supportive therapy should be initiated (including with antihistamines and/or steroids) as considered appropriate by the investigator/treating physician. Any subsequent decision on re-challenge with AZD5363 at the same or a lower dose, with its potential for recurrence of such or more severe AEs should be carefully considered against the potential benefits to the individual patient from continuation of AZD5363 treatment. Further management should follow local guidelines on management of hypersensitivity reactions.

8.1.14 Reproductive Considerations

See Section 3.1 for details on teratogenicity of agents and reproductive considerations while on study agent. Please note that for patients receiving vismodegib they will need to continue pregnancy prevention for 6 months after discontinuation of study drug. For patients receiving AZD5363, they will need to continue pregnancy prevention for 4 weeks after discontinuation of study drug. For patients receiving abemaciclib, they will need to use pregnancy prevention during treatment and for 3 weeks after discontinuation of study drug.

8.1.15 Liver Toxicity

Liver toxicity is a known side effect of GSK2256098. Monitor for symptoms of fatigue, nausea, vomiting, right upper quadrant pain, fever, rash, and eosinophilia, especially in the presence of any LFT abnormalities.

If hepatic toxicity develops, it is recommended to repeat liver chemistries within 72 hours, then twice weekly until resolution. In addition, it is recommended that alternate etiologies of liver toxicity be investigated, including viral (hepatitis B&C&E, CMV, EBV), environmental (e.g. alcohol exposure), and intrinsic liver disease (for which liver imaging should be considered). For abemaciclib specific hepatic toxicity, please see Section 8.5.5.

To ensure patient safety the investigator should collect specific recommended clinical information and follow-up laboratory tests as shown below.

Details for hepatic monitoring depend upon the severity and persistence of observed laboratory test abnormalities. If a study patient experiences elevated ALT 5×ULN and elevated TBL 2×ULN, or ALT 8×ULN, liver tests, including ALT, AST, TBL, direct bilirubin, gamma glutamyl transferase (GGT), and creatine phosphokinase (CPK), should be repeated within 3 to 5 days to confirm the abnormality and to determine if it is increasing or decreasing. If the abnormality persists or worsens, clinical and laboratory monitoring should be initiated by the investigator, based on the hepatic monitoring tests below.

Hepatic Monitoring Tests for a Hepatic Treatment Emergent Abnormality

**Hepatic Hematology**	**Haptoglobin**
Hemoglobin	
Hematocrit	**Hepatic Coagulation**
RBC	Prothrombin Time
WBC	Prothrombin Time, INR
Neutrophils, segmented and bands	
Lymphocytes	**Hepatic Serologies**[a]
Monocytes	Hepatitis A antibody, total
Eosinophils	Hepatitis A antibody, IgM
Basophils	Hepatitis B surface antigen
Platelets	Hepatitis B surface antibody
	Hepatitis B Core antibody
**Hepatic Chemistry**	Hepatitis C antibody
Total bilirubin	Hepatitis E antibody, IgG
Direct bilirubin	Hepatitis E antibody, IgM
Alkaline phosphatase	
ALT	**Anti-nuclear antibody**
AST	**Anti-actin antibody**
GGT	**Anti-smooth muscle antibody**
CPK	

Abbreviations: ALT = alanine aminotransferase; AST = aspartate aminotransferase; CPK = creatine phosphokinase; GGT = gamma-glutamyl transferase; Ig = immunoglobulin; INR = international normalized ratio; RBC = red blood cells; WBC = white blood cells.

a Reflex/confirmation dependent on regulatory requirements and/or testing availability.

8.1.16 QT Prolongation

For patients receiving GSK2256098:

If a QTcF >500 msec (or >60 msec change in QTcF from baseline) is noted on a scheduled or unscheduled EKG, it is recommended to repeat the EKG to confirm the abnormality. Also, the following is recommended in patients with prolonged QTc:

Electrolytes, particularly potassium and magnesium, should be checked and corrected if abnormal.

Concomitant medications with a potential for QTc interval prolongation should be discontinued if clinically appropriate.

Consider cardiology consultation and continuous monitoring.

8.1.17 Use of metformin

Metformin is recommended for the management of hyperglycemia occurring in patients participating receiving AZD5363. Investigators should exercise caution in the dosing and management of patients receiving the metformin/AZD5363 combination and must be vigilant for signs of renal impairment and metformin toxicity, such as lactic acidosis and hypoglycemia, namely: lethargy, hypotension, poor urine output, drowsiness, irritation, tachypnea, sweating, diarrhea, and vomiting. Metformin should only be given on the days when AZD5363 is also given (the half-life of AZD5363 is approximately 8 to15 hours),

and should be withdrawn when treatment with AZD5363 is also withdrawn, unless otherwise clinically indicated.

Due to the potential interaction of metformin and AZD5363 by inhibition of OCT2, it is recommended that creatinine be monitored for patients taking both AZD5363 and metformin concurrently. Creatinine assessments should be conducted as part of the routine clinical chemistry with additional monitoring of creatinine at the discretion of the investigator.

8.2 Vismodegib Dose Modifications

- If multiple adverse events are seen, administer dose based on greatest modification required for any single adverse event observed.
- Modifications apply to treatment given in the preceding cycle and are based on adverse events observed since the prior dose.
- If study agent is held for 4 weeks, study agent will be discontinued.
- Descriptors below utilize CTCAE version 4.0 expedited reporting via CTEP-AERS may be required for some adverse events (See Section 9.0).

8.2.1 Vismodegib dose levels

Vismodegib dose modifications by dose level are not utilized due to the pharmacokinetic characteristics of the drug, see Section 1.4 and Section 1.5 for further description. Rather disruptions of dose are utilized to manage toxicity. If a treatment interruption occurs, and it is determined that vismodegib will be re-started, the original dose will be maintained (150 mg PO daily).

8.2.2 Musculoskeletal and connective tissue disorder adverse event:

- **Grade 3 myalgias, grade ≥ 3 muscle spasms (musculoskeletal disorders other):** Delay vismodegib until myalgia or muscle spasm improves to ≤ grade 1, then resume vismodegib at same dose.
- **Grade 3 arthralgia**: Delay vismodegib until arthralgia improves to ≤ grade 1, then resume vismodegib at same dose.

8.2.3 Gastrointestinal toxicities:

- **Grade ≥ 3 diarrhea**: Delay vismodegib until diarrhea improves to ≤ grade 1, then resume vismodegib at same dose.
- **Grade ≥ 3 nausea or vomiting**: Delay vismodegib until nausea/vomiting improves to ≤ grade 1, then resume vismodegib at same dose

8.2.4 Metabolism and Nutrition disorder:

- **Grade ≥ 3 anorexia:** Delay vismodegib until ≤ grade 2 then resume at same dose.

8.2.5 General disorders:

- **Grade 3 fatigue:** Delay vismodegib until ≤ grade 2 then resume at same dose.

8.2.6 Investigations:

- **Grade 3 weight loss:** Delay vismodegib until ≤ grade 2 then resume at same dose.

8.2.7 Other adverse event:

- For all other grade 3 or 4 nonhematologic events, hold dose until grade ≤ 1, then resume vismodegib at same dose.

8.3 GSK2256098 dose modifications

- If multiple adverse events are seen, administer dose based on greatest reduction required for any single adverse event observed. Reductions or increases apply to treatment given in the preceding cycle and are based on adverse events observed since the prior dose.
- GSK2256098 will not be re-escalated once reduced
- If dose reductions beyond dose level -2 is required or GSK2256098 is held for 4 weeks, GSK2256098 will be discontinued.
- If more than one of these toxicities apply, use the most stringent criteria (i.e., the greatest dose reduction.)
- If the patient experiences a significant adverse event requiring a dose reduction at the start of the next cycle, then the dose will remain lowered.
- Descriptors below utilize CTCAE version 4.0. Expedited reporting via CTEP-AERS may be required for some adverse events (See Section 9.0).

8.3.1 Dose Levels

Dose Level	Drug Name	Dose
0*	**GSK2256098**	750 mg po bid
-1	**GSK2256098**	500 mg po bid
-2	**GSK2256098**	500mg po in AM, 250mg po in pm

*Dose level 0 refers to the starting dose.

8.3.2 Gastrointestinal toxicities:

- **Grade ≥ 3 diarrhea**: Delay GSK2256098 until diarrhea improves to ≤ grade 1, then resume GSK2256098 with one dose level reduced.
- **Grade 3 nausea or vomiting**: Delay GSK2256098 until nausea or vomiting improves to ≤ grade 1, then resume GSK2256098 with one dose level reduced
- **Grade 2 esophageal, gastric, small intestine, or colonic ulcer:** Delay GSK2256098 until grade ≤ 1 then resume at same dose
- **Grade 3 esophageal, gastric, small intestine, or colonic ulcer:** Delay GSK2256098 until grade ≤ 1 then resume at one dose level reduced
- **Grade 4 esophageal, gastric, small intestine, or colonic ulcer:** Discontinue GSK2256098

8.3.3 Hepatic Toxicity:

- **For grade 2 ALT, AST,** delay GSK2256098 until ≤ grade 1, then resume at same dose.

- **For grade 3 or 4 ALT, AST** discontinue GSK2256098.

- **For grade 2 bilirubin,** delay GSK2256098 until grade ≤ 1 then resume at same dose.
- **For grade 3, 4 bilirubin,** discontinue GSK2256098.
- **For combined grade 2 AST/ALT and grade 2 bilirubin, discontinue GSK2256098.**

8.3.4 Metabolism and nutrition:

- **Grade 3, 4 anorexia:** Delay GSK2256098 until ≤ grade 2 then resume at one dose level reduced.

8.3.5 Renal and urinary disorders:

- **Grade 2 proteinuria:** Delay GSK2256098 until ≤ grade 1 then resume at same dose.
- **Grade 3 proteinuria:** Delay GSK2256098 until ≤ grade 1 then resume at one dose level reduced.
- **Grade 2 hematuria:** Delay GSK2256098 until ≤ grade 1 then resume at same dose.
- **Grade 3 hematuria:** Delay GSK2256098 until ≤ grade 1 then resume at one dose level reduced.
- **Grade 4 hematuria:** Discontinue GSK2256098

8.3.6 Dermatologic Toxicity:

- **For grade 2 pruritis,** delay GSK2256098 until grade ≤ 1, then resume at same dose
- **For grade 3 pruritis,** delay GSK2256098 until grade ≤ 1, then resume with one dose level reduced.
- **For grade 2 maculopapular rash,** delay GSK2256098 until grade ≤ 1, then resume at same dose
- **For grade 3 maculopapular rash,** delay GSK2256098 until grade ≤ 1, then resume with one dose level reduced.

8.3.7 Investigations:

- For either grade 3 Electrocardiogram QT corrected interval prolonged OR increase in QTc from baseline of > 60 msec, in patient without underlying bundle branch block, discontinue GSK2256098
- For grade 3 Electrocardiogram QT corrected interval prolonged in patient with underlying bundle branch block and baseline QTc < 450 msec, discontinue GSK2256098
- For patient with underlying bundle branch block and baseline QTc of 450 to 480 msec, discontinue GSK2256098 with QTc ≥ 530 msec
- For grade 4 Electrocardiogram QT corrected interval prolonged, discontinue GSK2256098

8.3.8 Other adverse event:

- For all other grade 3 or 4 nonhematologic events, hold dose until grade ≤ 1, then resume GSK2256098 at same dose.

8.4 AZD5363 dose modifications

- If multiple adverse events are seen, administer dose based on greatest reduction required for any single adverse event observed. Reductions or increases apply to treatment given in the preceding cycle and are based on adverse events observed since the prior dose.
- AZD5363 will not be re-escalated once reduced.
- If dose reductions beyond dose level -2 is required or AZD5363 is held for 28 days, AZD5363 will be discontinued.
- If more than one of these toxicities apply, use the most stringent criteria (i.e., the greatest dose reduction.)
- If the patient experiences a significant adverse event requiring a dose reduction at the start of the next cycle, then the dose will remain lowered.
- If the event/grade is not specified in the following sections, then dose modifications are not required.
- Descriptors below utilize CTCAE version 4.0 expedited reporting via CTEP-AERS may be required for some adverse events (See Section 9.0).

8.4.1 Dose Levels:

Dose Level	Drug Name	Dose
0*	AZD5363	480 mg bid orally 4 days on/3 days off
-1	AZD5363	320 mg bid orally 4 days on/3 days off
-2	AZD5363	200 mg bid orally 4 days on/3 days off

*Dose level 0 refers to the starting dose.

8.4.2 Hematologic toxicity:

- **Grade 4 neutropenia, febrile neutropenia:** Delay AZD5363 until grade ≤ 2 then resume with one dose level reduced

8.4.3 Diarrhea

Grade ≥ 3 diarrhea or that is clinically significant or intolerable and causally related to treatment with AZD5363, use institution guidelines for anti-diarrheal treatment. Suggestions below:

- If clinically appropriate or if toxicity does not improve to CTCAE Grade ≤2 or remains clinically intolerable, despite optimal treatment, withhold AZD5363 for up to 14 days.
- If toxicity improves to CTCAE Grade ≤2 or becomes clinically tolerable reinstate AZD5363, as clinically appropriate, at either the current dose or at a reduced dose (1 dose level) maintaining treatment for toxicity as necessary.
- Where a CTCAE Grade >3 or clinically significant or intolerable toxicity does not improve after 14 days of AZD5363 dose interruption, AZD5363 should be permanently discontinued.

- Recurrence: on recurrence of a CTCAE Grade ≥3 or clinically significant or intolerable toxicity, reinstate treatment as required.
- If toxicity improves to CTCAE Grade ≤2 or becomes clinically tolerable reinstate AZD5363, as clinically appropriate, at either the current dose or at a reduced dose (up to two dose level reductions) maintaining treatment for toxicity as necessary.
- If toxicity does not improve to CTCAE Grade ≤2 or remains clinically significant or intolerable, despite optimal treatment, withhold dose for up to 14 days until improvement of toxicity

8.4.4 Other Gastrointestinal toxicities:

For all other ≥ Grade 3 GI toxicity, treat toxicity as clinically appropriate and withhold dose up for up to 14 days until toxicity improves to CTCAE Grade ≤2 or becomes clinically tolerable. If toxicity improves to CTCAE Grade ≤1 reinstate AZD5363 at the current dose maintaining treatment for toxicity as necessary. If toxicity improves to CTCAE Grade 2 reinstate AZD5363 at a reduced dose (1 dose level) maintaining treatment for toxicity as necessary. Where a CTCAE Grade ≥3 or clinically significant or intolerable toxicity does not improve to a lower CTCAE Grade with AZD5363 dose interruption, AZD5363 should be permanently discontinued. The length of suspension before permanent discontinuation of study treatment may be determined at the discretion of the investigator (it's 14 days in most of our CSPs), appropriate to patient management." Additionally, for

- Grade 4 stomatitis: Discontinue AZD5363
- Grade 4 esophageal, gastric, small intestine, or colonic ulcer: Discontinue AZD5363

8.4.5 Hepatic toxicity:

Please see Appendix X Figure 3 for hepatic toxicity management.

8.4.5 Dermatologic toxicity:

A suggested algorithm for the management of dermatologic toxicity is provided in Appendix X.

8.4.6 Metabolism:

A suggested algorithm for the management of hyperglycemia toxicity is provided in Appendix X.

8.4.7 Other toxicities:

- For all other grade 3 or 4 events or clinically significant or intolerable and causally related to treatment with AZD5363 withhold dose for as many as 14 days until toxicity improves to CTCAE ≤ 2 or becomes clinically tolerable.
- If toxicity improves to CTCAE grade ≤ 1 reinstate AZD5363 at the current dose maintaining treatment for toxicity as necessary.
- If toxicity improves to CTCAE grade 2 reinstate AZD5363 at a reduced dose (1 dose level) maintaining treatment for toxicity as necessary.
- Where a CTCAE grade ≥ 3 or clinically significant or intolerable toxicity does not improve to a lower CTCAE grade with AZD5363 dose interruption, AZD5363 should be permanently discontinued. The length of suspension before permanent discontinuation of study treatment may be determined at the discretion of the investigator.

8.5 Abemaciclib Dose Modifications

- If multiple adverse events are seen, administer dose based on greatest reduction required for any single adverse event observed. Reductions or increases apply to treatment given in the preceding cycle and are based on adverse events observed since the prior dose.
- Abemaciclib will not be re-escalated once reduced.
- If dose reductions beyond dose level -2 is required or abemaciclib is held for 28 days, abemaciclib will be discontinued.
- If more than one of these toxicities apply, use the most stringent criteria (i.e., the greatest dose reduction.)
- If the patient experiences a significant adverse event requiring a dose reduction at the start of the next cycle, then the dose will remain lowered.
- Descriptors below utilize CTCAE version 5.0. Expedited reporting via CTEP-AERS may be required for some adverse events (See Section 9.0).

8.5.1 Abemaciclib Dose Levels

Dose Level	Drug Name	Dose
0*	Abemaciclib	200 mg PO every 12 hours
-1	Abemaciclib	150 mg PO every 12 hours
-2	Abemaciclib	100 mg PO every 12 hours

*Dose level 0 refers to the starting dose.

8.5.2 Hematologic Adverse Event

- **Grade 3 hematologic toxicity:** Dose interruption until recovery to grade ≤2. Re-initiate abemaciclib at the same dose. If toxicity recurs at grade 3: temporary dose interruption until recovery to grade ≤2 and reduce abemaciclib to the next lower dose level.
- **Grade 4 hematologic toxicity:** Dose interruption until recovery to grade ≤2. Re-initiate abemaciclib at the next lower dose level.
- **Regardless of severity:** if patient requires administration of blood cell growth factors, dose must be suspended for at least 48 hours after the last dose of blood cell growth factors was administered and until toxicity resolves to at least Grade 2. The dose must be reduced at the next lower dose level unless already performed for incidence of toxicity that leads to the use of growth factor

8.5.3 Non-hematologic adverse event (excluding ALT)(for diarrhea, see Section 8.5.4)

- **Grade 3 or 4 non-hematologic toxicity:** Dose interruption until recovery to grade ≤1. Reduce abemaciclib to the next lower dose level.
- **Persistent or recurrent Grade 2 toxicity that does not resolve with maximal supportive measures:** Suspend dose until toxicity resolves to baseline or Grade 1. Resume at next lower dose.

8.5.4 Diarrhea

- At the first sign of loose stools, start treatment with antidiarrheal agents, such as loperamide.

- **Persistent or recurrent Grade 2 diarrhea that does not resolve with maximal supportive measures within 24 hours to baseline or Grade 1:** Suspend dose until toxicity resolves to ≤Grade 1. Resume at next lower dose.
- **Grade 3 or 4 diarrhea or requires hospitalization:** Dose interruption until recovery to grade ≤1. Reduce abemaciclib to the next lower dose level

8.5.5 Hepatotoxicity, including increased ALT

- **Persistent or recurrent Grade 2 or Grade 3 (>5.0-20.0 x ULN) that does not resolve with maximal supportive measures within 7 days to baseline or Grade 1:** Suspend dose until recovery to grade ≤1. Resume abemaciclib at the next lower dose level.
- **Grade 3 (>5.0 x ULN) with total bilirubin >2 x ULN, in the absence of cholestasis or Grade 4 (> 20 x ULN):** Discontinue abemaciclib

Dose Modification and Management — Increased ALT

Monitor ALT prior to the start of abemaciclib therapy, every 2 weeks for the first 2 months, monthly for the next 2 months, and as clinically indicated.	
**CTCAE Grade**	**Abemaciclib Dose Modifications**
Grade 1 (>ULN-3.0 x ULN)<br>Grade 2 (>3.0-5.0 x ULN)	No dose modification is required.
Persistent or Recurrent Grade 2, or Grade 3 (>5.0-20.0 x ULN) that does not resolve with maximal supportive measures within 7 days to baseline or Grade 1	Suspend dose until toxicity resolves to baseline or Grade 1. Resume at next lower dose.
Grade 3 (>5.0 x ULN)<br>with total bilirubin >2 x ULN,<br>in the absence of cholestasis	Discontinue abemaciclib.
Grade 4 (>20.0 x ULN)	Discontinue abemaciclib.

8.5.6 General Guidance for Interstitial lung disease (ILD)/Pneumonitis Events

Interstitial lung disease (ILD) / pneumonitis has been identified as an adverse drug reaction for abemaciclib. Adverse events reported included events such as interstitial lung disease, pneumonitis, obliterative bronchiolitis, organizing pneumonia, pulmonary fibrosis. The majority of events were Grade 1 or Grade 2 with serious cases and fatal events reported.

Please ask patients to report any new or worsening pulmonary symptoms such as dyspnoea, cough and fever; these symptoms should be investigated and treated as per local clinical practice and/or guidelines (including corticosteroids as appropriate). Investigations may include imaging such as high resolution computer tomography (HRCT), broncheoalveolar lavage (BAL), and biopsy as clinically indicated.

For patients who develop radiological changes suggestive of pneumonitis and have few or no symptoms (Grade 1), abemaciclib should be continued without dose modification For ≥Grade 3 or persistent or recurrent Grade 2 ILD/pneumonitis events, abemaciclib should be suspended until toxicity resolves to baseline or Grade 1, and resumed at the next lower dose (see also Table: refer to dose adjustment table for non-hematological toxicities).

Dose Modification and Management — Interstitial Lung Disease/Pneumonitis

CTCAE Grade	Abemaciclib Dose Modifications
Grade 1 or 2	No dose modification is required.
Persistent or recurrent Grade 2 toxicity that does not resolve with maximal supportive measures within 7 days to baseline or Grade 1	Suspend dose until toxicity resolves to baseline or Grade 1. Resume at next lower dose.
Grade 3 or 4	Discontinue abemaciclib.

Dose Modification and Management — Nonhematologic Toxicities Excluding Diarrhea, ALT Increased, and ILD/Pneumonitis

<table>
<tr><th>CTCAE Grade</th><th>Abemaciclib Dose Modifications</th></tr>
<tr><td>Grade 1 or 2</td><td>No dose modification is required.</td></tr>
<tr><td>Persistent or recurrent Grade 2 toxicity that does not resolve with maximal supportive measures within 7 days to baseline or Grade 1</td><td rowspan="2">Suspend dose until toxicity resolves to baseline or Grade 1. Resume at next lower dose.</td></tr>
<tr><td>Grade 3 or 4</td></tr>
</table>

8.5.7 Other toxicities

Consider performing an analysis of serum potassium, calcium, phosphorus, and magnesium for all adverse reactions that are potentially associated/aggravated with electrolyte imbalance (e.g. diarrhea, nausea/vomiting, non-QT related cardiovascular events). If electrolyte values are below the lower limit of normal, interrupt abemaciclib administration, correct electrolytes with supplements as soon as possible, and repeat electrolyte testing until documented normalization of the electrolytes.

9.0 ADVERSE EVENTS

The prompt reporting of adverse events is the responsibility of each investigator engaged in clinical research, as required by Federal Regulations. Adverse events must be described and graded using the terminology and grading categories defined in the NCI's Common Terminology Criteria for Adverse Events (CTCAE), Version 4.0. However, the descriptions and grading scales found in the NCI Common Terminology Criteria for Adverse Events (CTCAE) version 5.0 will be utilized for expedited AE reporting beginning April 1, 2018. The CTCAE is available at ctep.cancer.gov/protocolDevelopment/electronic_applications/ctc.htm. Attribution to protocol treatment for each adverse event must be determined by the investigator and reported on the required forms. Please refer the NCI Guidelines: Adverse Event Reporting Requirements for further details on AE reporting procedures.

9.1 Routine adverse event reporting

Adverse event data collection and reporting, which are required as part of every clinical trial are done to ensure the safety of patients enrolled in the studies as well as those who will enroll in future studies using similar agents. Adverse events are reported in a routine manner at scheduled

times according to the study calendar in Section 5.0. For this trial, the Adverse Events: Solicited form is used for routine AE reporting in Rave.

Solicited Adverse Events: The following adverse events are considered "expected" and their presence/absence should be solicited, and severity graded, at baseline and for each cycle of treatment.

CTCAE v4.0 Term	CTCAE v4.0 System Organ Class (SOC)
Weight loss	Investigations
Proteinuria	Investigations
Neutrophil count decreased	Investigations
Fatigue	General disorders and administration site conditions
Anorexia	Metabolism and nutrition disorders
Diarrhea	Gastrointestinal disorders
Dyspepsia	Gastrointestinal disorders
Nausea	Gastrointestinal disorders
Vomiting	Gastrointestinal disorders
Arthralgia	Musculoskeletal and connective tissue disorders
Headache	Nervous system disorders
Anaphylaxis	Immune system disorders
Dry skin	Skin and subcutaneous disorders
Pruritus	Skin and subcutaneous disorders
Rash maculopapular	Skin and subcutaneous disorders
Stomal ulcer	Injury, poisoning and procedural complications

9.2 CTCAE Routine Reporting Requirements

In addition to the solicited adverse events listed in Section 9.1, the following table outlines the combinations of time points, grades and attributions of AEs that require routine reporting to the Alliance Statistics and Data Center.

***Combinations of CTCAE Grade & Attribution Required for Routine AE Data Submission on Case Report Forms (CRFs)**

Attribution	Grade 1	Grade 2	Grade 3	Grade 4	Grade 5
Unrelated			a	a	a
Unlikely			a	a	a
Possible	a	a	a, b	a, b	a, b

Probable	a	a	a, b	a, b	a, b
Definite	a	a	a, b	a, b	a, b

a) Adverse Events: Other CRF - Applies to AEs occurring between registration and within 30 days of the patient's last treatment date, or as part of the Clinical Follow-Up Phase.
b) Adverse Events: Late CRF - Applies to AEs occurring greater than 30 days after the patient's last treatment date.

9.3 Expedited Adverse Event Reporting (CTEP-AERS)

Investigators are required by Federal Regulations to report serious adverse events as defined in the table below. Alliance investigators are required to notify the Investigational Drug Branch (IDB), the Alliance Central Protocol Operations Program, the Study Chair, and their Institutional Review Board if a patient has a reportable serious adverse event. The descriptions and grading scales found in the revised NCI Common Terminology Criteria for Adverse Events (CTCAE) version 5.0 will be utilized for AE reporting beginning April 1, 2018. All appropriate treatment areas should have access to a copy of the CTCAE version 5.0. A copy of the CTCAE version 5.0 can be downloaded from the CTEP web site http://ctep.cancer.gov/protocolDevelopment/electronic_applications/ctc.htm. All reactions determined to be "reportable" in an expedited manner must be reported using the Cancer Therapy Evaluation Program Adverse Event Reporting System (CTEP-AERS).
For further information on the NCI requirements for SAE reporting, please refer to the 'NCI Guidelines for Investigators: Adverse Event Reporting Requirements' document published by the NCI.

Note: All deaths on study require both routine and expedited reporting regardless of causality. Attribution to treatment or other cause should be provided.

9.3.1 Phase 1 and Early Phase 2 Studies: Expedited Reporting Requirements for Adverse Events that Occur on Studies under an IND/IDE within 30 Days of the Last Administration of the Investigational Agent/Intervention [1, 2]

FDA REPORTING REQUIREMENTS FOR SERIOUS ADVERSE EVENTS (21 CFR Part 312)

NOTE: Investigators **MUST** immediately report to the sponsor (NCI) **ANY** Serious Adverse Events, whether or not they are considered related to the investigational agent(s)/intervention (21 CFR 312.64)

An adverse event is considered serious if it results in **ANY** of the following outcomes:

1) Death
2) A life-threatening adverse event
3) An adverse event that results in inpatient hospitalization or prolongation of existing hospitalization for ≥ 24 hours
4) A persistent or significant incapacity or substantial disruption of the ability to conduct normal life functions
5) A congenital anomaly/birth defect.
6) Important Medical Events (IME) that may not result in death, be life threatening, or require hospitalization may be considered serious when, based upon medical judgment, they may jeopardize the patient or subject and may require medical or surgical intervention to prevent one of the outcomes listed in this definition. (FDA, 21 CFR 312.32; ICH E2A and ICH E6).

ALL SERIOUS adverse events that meet the above criteria MUST be immediately reported to the NCI via CTEP-AERS within the timeframes detailed in the table below.

<table>
<tr><th>Hospitalization</th><th>Grade 1 and Grade 2 Timeframes</th><th>Grade 3-5 Timeframes</th></tr>
<tr><td>Resulting in Hospitalization ≥ 24 hrs</td><td>10 Calendar Days</td><td rowspan="2">24-Hour 5 Calendar Days</td></tr>
<tr><td>Not resulting in Hospitalization ≥ 24 hrs</td><td>Not required</td></tr>
</table>

<u>Expedited AE reporting timelines are defined as:</u>

- o "24-Hour; 5 Calendar Days" - The AE must initially be reported via CTEP-AERS within 24 hours of learning of the AE, followed by a complete expedited report within 5 calendar days of the initial 24-hour report.
- o "10 Calendar Days" - A complete expedited report on the AE must be submitted within 10 calendar days of learning of the AE.

[1]Serious adverse events that occur more than 30 days after the last administration of investigational agent/intervention and have an attribution of possible, probable, or definite require reporting as follows:

Expedited 24-hour notification followed by complete report within 5 calendar days for:

- All Grade 3, 4, and Grade 5 AEs

Expedited 10 calendar day reports for:

- Grade 2 AEs resulting in hospitalization or prolongation of hospitalization

[2] For studies using PET or SPECT IND agents, the AE reporting period is limited to 10 radioactive half-lives, rounded UP to the nearest whole day, after the agent/intervention was last administered. Footnote "1" above applies after this reporting period.

- Expedited AE reporting timelines defined:
  - ➢ "24 hours; 5 calendar days" – The investigator must initially report the AE via CTEP-AERS ≤ <u>24 hours</u> of learning of the event followed by a complete CTEP-AERS report ≤ <u>5 calendar days</u> of the initial 24-hour report.
  - ➢ "10 calendar days" - A complete CTEP-AERS report on the AE must be submitted ≤ <u>10 calendar days</u> of the investigator learning of the event.
- Any medical event equivalent to CTCAE grade 3, 4, or 5 that precipitates hospitalization (or prolongation of existing hospitalization) must be reported regardless of attribution and designation as expected or unexpected with the exception of any events identified as protocol-specific expedited adverse event reporting exclusions (see below).
- Any event that results in persistent or significant disabilities/incapacities, congenital anomalies, or birth defects must be reported via CTEP-AERS if the event occurs following treatment with an agent under a CTEP IND.
- Use the NCI protocol number and the protocol-specific patient ID provided during trial registration on all reports.

Additional Instructions or Exclusion to CTEP-AERS Expedited Reporting Requirements for Phase 1 and Early Phase 2 Trials Utilizing an Agent Under a non-CTEP IND:

- All adverse events reported via CTEP-AERS (i.e., serious adverse events) should also be forwarded to your local IRB.

- Grade 1-3 fatigue and hospitalization resulting from such do not require expedited reporting via CTEP-AERS reporting, but should be reported via routine AE reporting.
- Grade 3 fatigue does not require expedited reporting via CTEP-AERS reporting, but should be reported via routine AE reporting
- Grade 1-3 alopecia and hospitalization resulting from such do not require expedited reporting via CTEP-AERS reporting, but should be reported via routine AE reporting.
- Grade 3 alopecia does not require expedited reporting via CTEP-AERS reporting, but should be reported via routine AE reporting
- Reporting of cases of secondary AML/MDS is to be done using the NCI/CTEP Secondary AML/MDS Report Form.
- All new malignancies must be reported via CTEP-AERS whether or not they are thought to be related to either previous or current treatment. All new malignancies should be reported, i.e. solid tumors (including non-melanoma skin malignancies), hematologic malignancies, myelodysplastic syndrome/acute myelogenous leukemia, and in situ tumors. In CTCAE version 5.0, the new malignancies (both second and secondary) may be reported as one of the following: (1) Leukemia secondary to oncology chemotherapy, (2) Myelodysplastic syndrome, (3) Treatment-related secondary malignancy, or (4) Neoplasms benign, malignant and unspecified-other. Whenever possible, the CTEP-AERS reports for new malignancies should include tumor pathology, history or prior tumors, prior treatment/current treatment including duration, any associated risk factors or evidence regarding how long the new malignancy may have been present, when and how the new malignancy was detected, molecular characterization or cytogenetics of the original tumor (if available) and of any new tumor, and new malignancy treatment and outcome, if available.
- CTEP-AERS reports should be submitted electronically.
- Pregnancy Loss
    - Pregnancy loss is defined in CTCAE as “Death in utero.” Any Pregnancy loss should be reported expeditiously, as Grade 4 “Pregnancy loss” under the Pregnancy, puerperium and perinatal conditions SOC.
    - A Pregnancy loss should NOT be reported as a Grade 5 event under the Pregnancy, puerperium and perinatal conditions SOC, as currently CTEPAERS recognizes this event as a patient death.
    - A neonatal death should be reported expeditiously as Grade 4, “Death neonatal” under the General disorders and administration SOC.
- Note: A death on study requires both routine and expedited reporting, regardless of causality. Attribution to treatment or other cause must be provided.
    - Death due to progressive disease should be reported as Grade 5 “Disease progression” in the system organ class (SOC) “General disorders and administration site conditions.” Evidence that the death was a manifestation of underlying disease (e.g., radiological changes suggesting tumor growth or progression: clinical deterioration associated with a disease process) should be submitted.

Expedited Adverse Event Reporting for Suspected Exposure to Agent that May Cause Serious or Life-threatening Birth Defects for Patients Receiving Vismodegib

- CTEP considers **any possible prenatal exposure to vismodegib**, a reportable expedited adverse event that should be reported to CTEP-AERS as a 24-hour notification followed by a complete report within 5 calendar days.

- Any patient suspected of being pregnant or fathering a child, (i.e. any female patient or female partner of a male patient, respectively), or should any lapse in contraception occur, should stop taking vismodegib until it is confirmed that pregnancy has not occurred.
- Pregnancies that occur up to 24 months after the last dose of vismodegib will be followed until the outcome of the pregnancy is known.
- The adverse event (pregnancy) should be reported as a Grade 4 event using CTCAE 5.0 as follows:
    - Endocrine disorders - Other (Prenatal exposure to a possible teratogen)
    - A completed "Possible Prenatal Exposure to Teratogen Report" Form (Appendix VII; note that "AdEERS" has been replaced by "CTEP-AERS") should be attached to the complete CTEP-AERS Report. This form may also be faxed to CTEP along with any relevant supporting medical information at *301-230-0159* (alternative FAX Number: *301-897-7404*).
    - This form should be submitted for any female patient or any female partner of a male patient who becomes pregnant during therapy or up to 24months after the last dose of vismodegib.
- Any congenital anomaly/birth defect in a child conceived to a female patient or to a female partner of a male patient exposed to GDC-0449 during treatment or within 24 months after the last dose of vismodegib should be reported as an expedited adverse event to CTEP-AERS as a 24-hour notification followed by a complete report within 5 calendar days.
- Abortion, whether accidental, therapeutic, or spontaneous, should always be classified as serious. Any abortion occurring during the study or within 24 months after the last dose of vismodegib to a female patient or to a female partner of a male patient exposed to the agent during treatment or within 24 months after the last dose of vismodegib should be reported as an expedited adverse event to CTEP-AERS as a 24-hour notification followed by a complete report within 5 calendar days.

9.4 Comprehensive Adverse Events and Potential Risks list (CAEPR) for GDC-0449 (Vismodegib, NSC 747691)

The Comprehensive Adverse Events and Potential Risks list (CAEPR) provides a single list of reported and/or potential adverse events (AE) associated with an agent using a uniform presentation of events by body system. Refer to the 'CTEP, NCI Guidelines:

Adverse Event Reporting Requirements' http://ctep.cancer.gov/protocolDevelopment/electronic_applications/docs/aeguidelines.pdf for further clarification. *Frequency is provided based on 1893 patients.* Below is the CAEPR for GDC-0449 (Vismodegib).

Version 2.5, December 22, 2016[1]

**Adverse Events with Possible Relationship to GDC-0449 (Vismodegib) (CTCAE 4.0 Term) [n= 1893]**		
**Likely (>20%)**	**Less Likely (<=20%)**	**Rare but Serious (<3%)**
GASTROINTESTINAL DISORDERS		
	Abdominal pain	
	Constipation	
	Diarrhea	
	Dyspepsia	
	Nausea	
	Vomiting	

Adverse Events with Possible Relationship to GDC-0449 (Vismodegib) (CTCAE 4.0 Term) [n= 1893]		
**Likely (>20%)**	**Less Likely (<=20%)**	**Rare but Serious (<3%)**
GENERAL DISORDERS AND ADMINISTRATION SITE CONDITIONS		
Fatigue		
INVESTIGATIONS		
	CPK increased	
Weight loss		
METABOLISM AND NUTRITION DISORDERS		
Anorexia		
	Dehydration	
MUSCULOSKELETAL AND CONNECTIVE TISSUE DISORDERS		
	Arthralgia	
Musculoskeletal and connective tissue disorder - Other (muscle spasms/twitching)		
		Musculoskeletal and connective tissue disorder - Other (premature epiphyseal closure)
NERVOUS SYSTEM DISORDERS		
Dysgeusia		
REPRODUCTIVE SYSTEM AND BREAST DISORDERS		
Irregular menstruation[2]		
SKIN AND SUBCUTANEOUS TISSUE DISORDERS		
Alopecia		

[1]This table will be updated as the toxicity profile of the agent is revised. Updates will be distributed to all Principal Investigators at the time of revision. The current version can be obtained by contacting PIO@CTEP.NCI.NIH.GOV. Your name, the name of the investigator, the protocol and the agent should be included in the e-mail.

[2]Irregular menstruation was observed in 30% (3 of 10) women of child bearing age and/or in 28% (18 of 64) women who had menses at baseline who were enrolled in studies of advanced BCC.

Adverse events reported on GDC-0449 (Vismodegib) trials, but for which there is insufficient evidence to suggest that there was a reasonable possibility that GDC-0449 (Vismodegib) caused the adverse event:

BLOOD AND LYMPHATIC SYSTEM DISORDERS - Anemia; Febrile neutropenia; Thrombotic thrombocytopenic purpura
CARDIAC DISORDERS - Atrial fibrillation; Atrial flutter; Cardiac arrest; Heart failure; Myocardial infarction; Pericardial tamponade; Sinus bradycardia
EYE DISORDERS - Keratitis; Retinal vascular disorder
GASTROINTESTINAL DISORDERS - Abdominal distension; Ascites; Dry mouth; Dysphagia; Esophageal pain; Esophagitis; Flatulence; Gastritis; Gastroesophageal reflux disease; Gastrointestinal disorders - Other (thrush); Gastrointestinal hemorrhage[49]; Gastrointestinal pain; Ileus; Mucositis oral; Pancreatitis; Stomach pain
GENERAL DISORDERS AND ADMINISTRATION SITE CONDITIONS - Edema limbs; Facial pain; Fever; Injection site reaction; Non-cardiac chest pain; Pain
HEPATOBILIARY DISORDERS - Cholecystitis; Hepatic failure; Portal hypertension

INFECTIONS AND INFESTATIONS - Infection[50]
INJURY, POISONING AND PROCEDURAL COMPLICATIONS - Bruising; Hip fracture
INVESTIGATIONS - Alanine aminotransferase increased; Alkaline phosphatase increased; Aspartate aminotransferase increased; Blood bilirubin increased; Cholesterol high; Creatinine increased; GGT increased; INR increased; Investigations - Other (brain natriuretic peptide increased); Investigations - Other (elevated LDH); Lipase increased; Lymphocyte count decreased; Neutrophil count decreased; Platelet count decreased; White blood cell decreased
METABOLISM AND NUTRITION DISORDERS - Hypercalcemia; Hyperglycemia; Hyperkalemia; Hypermagnesemia; Hypernatremia; Hypoalbuminemia; Hypocalcemia; Hypoglycemia; Hypokalemia; Hypomagnesemia; Hyponatremia; Hypophosphatemia
MUSCULOSKELETAL AND CONNECTIVE TISSUE DISORDERS - Arthritis; Back pain; Flank pain; Generalized muscle weakness; Muscle weakness lower limb; Musculoskeletal and connective tissue disorder - Other (muscle tightness/stiffness); Myalgia; Neck pain; Pain in extremity; Trismus
NEOPLASMS BENIGN, MALIGNANT AND UNSPECIFIED (INCL CYSTS AND POLYPS) - Leukemia secondary to oncology chemotherapy; Treatment related secondary malignancy
NERVOUS SYSTEM DISORDERS - Ataxia; Cognitive disturbance; Dizziness; Dysesthesia; Headache; Intracranial hemorrhage; Movements involuntary; Nervous system disorders - Other (amimia); Olfactory nerve disorder; Paresthesia; Peripheral motor neuropathy; Peripheral sensory neuropathy; Seizure; Stroke; Syncope; Tremor
PSYCHIATRIC DISORDERS - Agitation; Anxiety; Confusion; Depression; Hallucinations; Insomnia; Psychosis
RENAL AND URINARY DISORDERS - Acute kidney injury; Renal hemorrhage
REPRODUCTIVE SYSTEM AND BREAST DISORDERS - Erectile dysfunction
RESPIRATORY, THORACIC AND MEDIASTINAL DISORDERS - Aspiration; Cough; Dyspnea; Epistaxis; Hiccups; Hypoxia; Pleural effusion; Pneumonitis; Postnasal drip; Pulmonary edema; Respiratory, thoracic and mediastinal disorders - Other (COPD); Respiratory, thoracic and mediastinal disorders - Other (oropharyngeal pain); Sneezing; Sore throat
SKIN AND SUBCUTANEOUS TISSUE DISORDERS - Hyperhidrosis; Nail ridging; Pruritus; Rash acneiform; Rash maculo-papular; Skin and subcutaneous tissue disorders - Other (hair color changes); Skin and subcutaneous tissue disorders - Other (psoriasis); Skin and subcutaneous tissue disorders - Other (skin exfoliation); Skin ulceration; Stevens-Johnson syndrome
VASCULAR DISORDERS - Hypertension; Hypotension; Thromboembolic event; Vasculitis

Note: GDC-0449 (Vismodegib) in combination with other agents could cause an exacerbation of any adverse event currently known to be caused by the other agent, or the combination may result in events never previously associated with either agent.

9.5 Comprehensive Adverse Events and Potential Risks list (CAEPR) for AZD5363 (NSC 782347)

The Comprehensive Adverse Events and Potential Risks list (CAEPR) provides a single list of reported and/or potential adverse events (AE) associated with an agent using a uniform presentation of events by body system. Refer to the 'CTEP, NCI Guidelines: Adverse Event Reporting Requirements' http://ctep.cancer.gov/protocolDevelopment/electronic_applications/docs/aeguidelines.pdf for further clarification. Frequency is provided based on 297 patients. Below is the CAEPR for AZD5363.

Version 2.2, May 10, 2017[1]

**Adverse Events with Possible Relationship to AZD5363 (CTCAE 4.0 Term) [n= 297]**		
**Likely (>20%)**	**Less Likely (<=20%)**	**Rare but Serious (<3%)**
GASTROINTESTINAL DISORDERS		
Diarrhoea		
Nausea		
Vomiting		
	Stomatitis[2]	
IMMUNE SYSTEM DISORDERS		
		Drug hypersensitivity
METABOLISM AND NUTRITION DISORDERS		
Hyperglycaemia		
Decreased appetite		
SKIN AND SUBCUTANEOUS TISSUE DISORDERS		
Rash[3]		
	Dry skin	
	Pruritus	

[1]This table will be updated as the toxicity profile of the agent is revised. Updates will be distributed to all Principal Investigators at the time of revision. The current version can be obtained by contacting PIO@CTEP.NCI.NIH.GOV. Your name, the name of the investigator, the protocol and the agent should be included in the e-mail.
[2]Stomatitis may include Aphthous ulcer, Aphthous stomatitis, Mouth ulceration
[3]Rash may include Erythema, Rash, Rash erythematous, Rash macular, Rash maculo-papular, Rash papular, Rash pruritic

Adverse events reported on AZD5363 trials (reported in >10% of patients in the 480 mg 4 days on/3 days off dose group), but for which there is insufficient evidence to suggest that there was a reasonable possibility that AZD5363 caused the adverse event:

BLOOD AND LYMPHATIC SYSTEM DISORDERS - Anaemia
GASTROINTESTINAL DISORDERS – Abdominal pain, Constipation
GENERAL DISORDERS AND ADMINISTRATION SITE CONDITIONS – Fatigue, Pyrexia, Oedema peripheral
INVESTIGATIONS - Blood creatinine increased
MUSCULOSKELETAL AND CONNECTIVE TISSUE DISORDERS - Back pain
NERVOUS SYSTEM DISORDERS – Headache, Dizziness
RENAL AND URINARY DISORDERS - Proteinuria
RESPIRATORY, THORACIC AND MEDIASTINAL DISORDERS - Cough

Important Potential Risks, based on nonclinical studies.

CARDIAC DISORDERS – QT Prolongation

Note: AZD5363 in combination with other agents could cause an exacerbation of any adverse event currently known to be caused by the other agent, or the combination may result in events never previously associated with either agent.

10.0 DRUG INFORMATION

Investigators ordering and/or dispensing supplied agents at any time for study treatment must be currently registered with PMB, DCTD, NCI. A registered investigator must co-sign for other non-registered personnel prescribing the supplied agents.

10.1 Vismodegib (GDC-0449, Erivedge®, NSC# 747691, IND#126926) IND holder: Alliance

Procurement

Vismodegib is an investigational, oral small molecule inhibitor of SMO that will be provided by Genentech and distributed by McKesson Specialty Pharmacy Use the order form on the A071401 webpage to order vismodegib.

Formulation

Vismodegib is supplied as 150 mg capsules. Each vismodegib capsule contains 150 mg vismodegib and the following inactive ingredients: microcrystalline cellulose PH101, lactose monohydrate, sodium lauryl sulfate, povidone K29/32, sodium starch glycolate, talc, magnesium stearate, and purified water. The capsule shell contains gelatin, titanium dioxide, red iron oxide, and black iron oxide.

Preparation, Storage and Stability

All drug supplies should be stored in a secure location, at room temperature. Do not store above 30ºC. Information on the shelf life of the capsules is provided on the label.

Administration

Vismodegib is taken orally once daily with or without food. If a dose is missed, do not make up that dose; resume dosing with the next scheduled dose.

Drug Accountability

The NCI Investigational Agent Accountability Record Form for Oral Agents should be utilized.

Drug Interactions

Vismodegib is a minor substrate of CYP2C8 and CYP3A4 *in vitro*. CYP inhibition is not predicted to alter vismodegib systemic exposure as similar steady-state plasma vismodegib concentrations were observed in patients in clinical trials concomitantly treated with CYP3A4 inducers (e.g. carbamazepine, modafinil, phenobarbital) and CYP3A4 inhibitors (e.g. erythromycin, fluconazole). Vismodegib is not a potent inhibitor of CYP1A2, CYP2B6, CYP2D6, and CYP3A4/5. In vitro, studies indicate vismodegib is an inhibitor of CYP2C8, CYP2C9, CYP2C19 and BCRP. However, vismodegib was found to have low potential for inhibiting CYP2C8 and CYP2C9 as systemic exposure of rosiglitazone and oral contraceptives were not altered with concomitant vismodegib. Vismodegib does not induce CYP1A2, CYP2B6 or CYP3A4/5 in human hepatocytes.

Vismodegib is a substrate of the efflux transporter P-glycoprotein. Co-administration of vismodegib and drugs that inhibit P-glycoprotein (e.g. clarithromycin, itraconazole, erythromycin, verapamil, diltiazem) may increase systemic exposure and toxicity of vismodegib.

Solubility is altered and bioavailability is reduced with the coadministration of vismodegib and drugs that alter the pH of the upper GI tract (e.g. proton pump inhibitors, H2-receptor antagonists, and antacids). No formal clinical studies have been conducted to evaluate the effect of gastric pH altering agents on vismodegib. Coadminister drugs that alter the pH of the upper GI tract with caution as systemic exposure of vismodegib may be decreased and the effect on efficacy is unknown.

Pharmacokinetics

Absorption: Bioavailability is 31.8%. Absorption is saturable. Vismodegib may be taken without regard to meals because the systemic exposure of vismodegib at steady state is not affected by food.

Distribution: $V_{d:}$ 16.4 to 26.6L. Plasma protein binding is greater than 99%.

Metabolism: Greater than 98% of the total circulating drug related components are parent drug. Metabolic pathways include oxidation, glucuronidation and pyridine ring cleavage. Two metabolites recovered in the feces are produced *in vitro* by CYP2C9 and CYP3A4/5.

Half-life elimination: 4 days after continuous once daily dosing; 12 days after a single dose

Excretion: Feces (82%); urine (4.4%)

Adverse Events

See CAEPR in Section 9.4.

Nursing Guidelines

Patients should be advised to keep their supply of vismodegib in a secure location to prevent accidental or deliberate use by others.

Patients should be thoroughly counseled and informed of the teratogenic potential of vismodegib. See Section 3.1.

Vismodegib has the potential to inhibit drugs that are substrates of the CYP2C8, CYP2C9 and CYP2C19 pathways. Assess patient's current medication list, including over the counter agents.

Patients may experience fatigue while on this agent. Instruct patient in energy conserving lifestyle.

Dysgeusia can be seen and is usually mild.

Alopecia has been seen and may be complete (scalp, eyelashes, eyebrows, etc.). Warn patients of this possibility.

Gastrointestinal side effects, including nausea, vomiting (with resulting dehydration) and dyspepsia have been reported. Treat symptomatically and monitor for effectiveness.

Muscle pain and spasms can occur. Treat symptomatically and monitor for effectiveness.

Patients should be instructed to take their pills at the same time each day with or without food. These must be swallowed whole and cannot be crushed or opened for any reason. Patients should not take missed doses, but just resume with the next scheduled dose.

10.2 GSK2256098 (NSC# 783781, IND #126926)

Procurement

GSK2256098 is an investigational, oral small molecule inhibitor of focal adhesion kinase (FAK) supplied by GlaxoSmithKline and distributed by McKesson Specialty Pharmacy. Use the order form on the A071401 webpage to order GSK2256098.

GSK2256098 supply has an expiry of September 2019 (at the latest). Currently, there are no plans for further supply to be manufactured, therefore supply of GSK2256098 for this study will expire September 2019.

Formulation

GSK2256098 is supplied as 250 mg capsules. The capsule filling contains microcrystalline cellulose, croscarmellose sodium, and magnesium stearate. The pink opaque hard gelatin capsule (250 mg) is composed of red iron oxide, titanium dioxide and gelatin

Preparation, Storage and Stability

All drug supplied should be stored in a secure location, at room temperature protected from light. GSK2256098 can be stored at up to 30 degrees Celsius (86 degrees Fahrenheit).

Administration

GSK2256098 is taken orally twice daily with or without food. If a dose of GSK2256098 is missed, do not make up that dose; resume dosing with the next scheduled dose. Capsules should not be opened or crushed. Retain GSK2256098 in the bottle provided until use.

Drug Accountability

The NCI Investigational Agent Accountability Record Form for Oral Agents should be utilized.

Upon completion of the trial, all remaining drug at the site must be destroyed as per local institutional policy, and notification of destruction must be sent to pharmaffairs@alliancenctn.org within 90 days of trial completion.

*Drug Interaction*s

No in vivo studies have been performed specifically to evaluate potential interactions with drugs that may be co-administered with GSK2256098.

GSK2256098 is primarily metabolized by CYP3A4 and is a substrate of membrane transporter P-glycoprotein. Strong inhibitors of CYP3A4 may increase exposure of GSK256098. Strong inducers of CYP3A4 may decrease exposure of GSK2256098.

GSK2256098 is an inhibitor of CYP2C8, CYP2C9 and CYP3A4, UGT1A1 and of the membrane transporter protein OATP1B1. GSK2256098 is an inducer of CYP3A4 and CYP2B6. GSK2256098 may also inhibit transport of P-glycoprotein substrates. Caution should be used with concomitant medications that are substrates with narrow therapeutic indices of CYP2C8, CYP2C9, CYP3A4, UGT1A1 and P-glycoprotein.

Pharmacokinetics

Absorption: Rapidly absorbed with a time to maximal concentration of 1 to 3 hours. Following administration of a high-fat meal, the Tmax was delayed and mean Cmax decreased by 55% relative to the fasted state; the change in AUC (15% decrease) was not clinically meaningful. Food decreased the rate, but not the extent of GSK2256098 absorption.

Distribution: Plasma protein binding is 89.2%.

Metabolism: Hepatic via CYP3A4 (98.8%) with a minimal contribution from CYP2C8.

$T_{1/2}$: Mean half-life was 4.4 hours (at 1000 mg dose level).

Adverse Events

The most frequently reported adverse events associated with continuous oral BID dosing of GSK2256098 were nausea (76%), diarrhea (65%), vomiting (58%), decreased appetite (47%), proteinuria (26%), fatigue (24%), asthenia (23%), hyperbilirubinemia (23%), constipation (21%) and hypercholesterolemia (21%).

Special Warnings and Precautions for Use

Patients receiving GSK2256098 should avoid direct sunlight or UV exposure while receiving study medication. In case of direct exposure, the use of protective clothing, sun glasses and sunscreen is recommended.

Nursing Guidelines

Instruct patients that GSK2256098 may be taken with or without food.

Do not crush or open capsules.

Agent should be taken twice daily. Missed doses should not be made up.

While formal drug to drug studies have not been performed, drugs that are strong inducers of CYP3A4 may decrease the exposure of agent. Assess patient’s concomitant medications and instruct patients to report any new medications to the study team.

Gastrointestinal side effects were common, including nausea, diarrhea and vomiting. Treat symptomatically and assess for effectiveness of intervention.

Instruct patients that fatigue and asthenia may occur. Instruct patient in energy conserving lifestyle.

Monitor bilirubin and instruct patients to report any jaundice symptoms to the study team immediately.

Patients should be instructed to avoid direct sunlight or UV exposure. Patients should be instructed on the use of protective clothing, sun glasses and sunscreen.

Monitor bilirubin and instruct patients to report any jaundice symptoms to the study team immediately. Patients should be instructed to avoid direct sunlight or UV exposure. Patients should be instructed on the use of protective clothing, sun glasses and sunscreen.

10.3 AZD5363 (NSC # 782347, IND #126926)

Procurement

AZD5363 is an investigational oral, potent, selective inhibitor of serine/threonine specific protein kinase (AKT) supplied by AstraZeneca and distributed by McKesson Specialty Pharmacy. Use the order form on the A071401 webpage to order AZD5363. Outdated or remaining drug is to be destroyed on-site as per procedures in place at each institution.

Formulation

AZD5363 is supplied as 160 or 200 mg film-coated tablets. AZD5363 tablets are packaged in high-density polyethylene (HDPE) bottles containing 60 tablets or 76 tables (for both 160mg and 200 mg dose). AZD5363 film-coated tablets contain AZD5363, microcrystalline cellulose, mannitol, croscarmellose sodium and magnesium stearate. The tablet film-coat contains polyvinyl alcohol, titanium dioxide, polyethylene glycol, talc, yellow iron oxide, red iron oxide and black iron oxide.

Preparation, Storage and Stability

All drug supplied should be stored in a secure location. AZD5363 should be stored below 30ºC.

Administration

AZD5363 is taken orally twice daily for four consecutive days followed by 3 days off (i.e. Days 1 through 4, 8 through 11, 15 through 18, 22 through 25 of a 28-day cycle). Patients receiving AZD5363 should be instructed to fast from 2 hours before AZD5363 dosing to 1 hour after dosing. If a dose of AZD5363 is missed, do not make up that dose; resume dosing with the next scheduled dose. Tablets should not be crushed. Retain AZD5363 in the bottle provided until use.

Drug Interactions

In vitro studies indicate that AZD5363 is a substrate of CYP3A4 although glucuronidation may be the major metabolic route. Coadministration of CYP3A4 inhibitors (particularly strong CYP3A4 inhibitors like voriconazole, posaconazole, itraconazole) may increase exposure of AZD5363 and AZD5363 toxicity. Inducers of CYP3A4 (especially strong inducers like carbamazepine, phenytoin) may decrease exposure of AZD5363 and reduce AZD5363 efficacy.

AZD5363 is a time-dependent inhibitor of CYP3A4 which may result in increased exposure of drugs metabolized by CYP3A4. Use caution concomitantly administering medications that are sensitive substrates of CYP3A4 (e.g. lovastatin, simvastatin, fentanyl, triazolam) as this may put patients at increased risk of toxicity from the CYP3A4 substrate. AZD5363 is also a moderate inhibitor of CYP2D6 in vitro. This may increase exposure of drugs metabolized by CYP2D6 (e.g. dextromethorphan, nortriptyline, venlafaxine, metoprolol, tramadol) with the potential to increase the toxicity of these drugs when co-administered.

Pharmacokinetics

Absorption: The results of effects of food on AZD5363 indicate that the presence of food may reduce the rate of absorption of AZD5363, the extent of absorption may be comparable. The clinical relevance of the food effect is currently unknown. It is currently recommended AZD5363 be taken on an empty stomach (i.e. patients to fast from 2 hours before dosing to 1 hour after dosing).
Distribution: Time to steady state was 1 hour with the tablet formulation.
Metabolism: AZD5363 is metabolized via CYP3A4 although glucuronidation may be the major metabolic route.
Excretion: Elimination is mainly via the feces.
$T_{1/2}$: Mean half-live was 8.4 to 11.2 hours.

Adverse Events

See CAEPR in Section 9.5.

Nursing Guidelines

Instruct patients to fast 2 hours prior to and for 1 hour after dose of AZD5363. Missed doses should not be made up. Also tablets should not be crushed and should remain in the original bottle (do not use pill containers, etc).

AZD5363 can cause elevations in blood sugar. Monitor blood glucose closely especially in patients who are diabetic and/or have a pre-disposition to diabetes.

Monitor blood pressure as hypotension has been seen in animal studies.

Gastrointestinal side effects including diarrhea, constipation, nausea, vomiting, and abdominal pain have been seen. Treat symptomatically and monitor for effectiveness.

Patients may experience rash and dry skin. Instruct patients on proper skin moisture methods and that they should report any rash to the study team.

Patients may experience fatigue, instruct patient in energy conserving lifestyle.

Monitor CBC w/diff. Anemia has been seen.

Investigator Brochure Availability
The investigator brochure for AZD5363 may be obtained by contacting the Alliance Central Protocol Operations Program office at protocols@alliancenctn.org.

10.4 Abemaciclib (LY2835219, VERENZIO™, NSC#783671)

Abemaciclib has been approved by U.S. Food and Drug Administration (FDA) in September 2017 for the following indications:

- In combination with fulvestrant for the treatment of women with hormone receptor (HR)-positive, human epidermal growth factor receptor 2 (HER2)-negative advanced or metastatic breast cancer with disease progression following endocrine therapy
- As monotherapy for the treatment of adult patients with HR-positive, HER2-negative advanced or metastatic breast cancer with disease progression following endocrine therapy and prior chemotherapy in the metastatic setting

In February, 2018, U.S. FDA has approved abemaciclib in combination with an aromatase inhibitor (AI) as initial endocrine-based therapy for the treatment of postmenopausal women with hormone receptor-positive (HR+), human epidermal growth factor receptor 2-negative (HER2-) advanced or metastatic breast cancer.

Procurement

Abemaciclib will be provided by Eli Lilly and company and distributed by McKesson Specialty Pharmacy. Use the abemaciclib order form on the A071701 study page.

Formulation

Chemical name: 2-Pyrimidinamine,N-[5-[(4-ethyl-1-piperazinyl)methyl]-2-pyridinyl]-5-fluoro-4-[4¬ fluoro-2-methyl-1-(1-methylethyl)-1H-benzimidazol-6-yl]-N-(5-((4-ethylpiperazin-1-yl)methyl)pyridine-2-yl)-5-fluoro-4-(4-fluoro-1-isopropyl-2-methyl-1Hbenzo[d]imidazole-6-yl)pyrimidin-2-amine

Molecular formula: $C_{27}H_{32}F_2N_8$

Molecular weight: 506.59 g/mol

Solubility: 0.001 mg/mL in water, 22.313 mg/mL in ethanol

Mode of action: Abemaciclib is a selective and potent inhibitor of cyclin-dependent kinases 4 and 6 (CDK4 and CDK 6).

These kinases are activated upon binding to D-cyclins, which initiates the transition through the G1 restriction point by phosphorylating and inactivating the retinoblastoma (Rb) tumor-suppressor protein. The inhibition of CDK4 and CDK6 is to prevent cell cycle progression through the G1 restriction point, thus arresting tumor growth.

Abemaciclib demonstrates significant inhibition of tumor growth as monotherapy in multiple human xenograft models including models for: breast cancer, colorectal cancer, glioblastoma multiforme, acute myeloid leukemia, melanoma, mantle cell lymphoma (MCL), and non-small-cell lung cancer (NSCLC). In nonclinical species, abemaciclib distributes efficiently to the brain and potentially provides a unique opportunity to treat primary brain tumors as well as cancers that have metastasized to the brain.

Description: practically white to yellow solid

How supplied: Abemaciclib is supplied as 50 mg in tablet formulation.

Tablet product: A modified oval, immediate release, aqueous film-coated abemaciclib tablets in 50 mg strength with the following inactive ingredients: microcrystalline cellulose, lactose monohydrate, croscarmellose sodium, silicon dioxide, sodium stearyl fumarate, color mixture beige (50 mg tablets). Each bottle contains 60 tablets.

Storage and Stability

Storage: store at 20°C to 25°C (68°F to 77°F); excursions permitted to 15°C to 30°C (59°F to 86°F).

Stability: stable at room temperature

Administration

Abemaciclib should be administered orally twice daily with or without food at approximately the same times every day. If the patient vomits or misses a dose of abemaciclib, instruct the patient to take the next dose at its scheduled time.

Patients should swallow whole tablets and not chew, crush, or split tablets before swallowing.

Drug accountability

Manual or electronic NCI Investigational Agent Accountability Record Form for Oral agents should be used to document receipt, dispensing, patient's returns, and disposal of study drugs.

Drug interactions

Abemaciclib is predominantly cleared by oxidative metabolism via CYP3A. Clinical drug interaction studies with a CYP3A4 inhibitor and CYP3A inducer significantly altered the PK of abemaciclib and its circulating major metabolites.

CYP3A inducers: Avoid concomitant use of CYP3A inducers and consider alternative agents.

CYP3A4 inhibitors: Strong CYP3A4 inhibitors increased the exposure of abemaciclib plus its active metabolites to a clinically meaningful extent and may lead to increased toxicity. Co-administration of abemaciclib with strong CYP3A inducers decreased the plasma concentrations of abemaciclib plus its active metabolites and may lead to reduced activity. Avoid concomitant use of strong CYP3A inhibitors (for example, voriconazole) and use caution with coadministered moderate (for example, ciprofloxacin) or weak (for example, ranitidine) CYP3A inhibitors. If coadministration with a strong CYP3A inhibitor is unavoidable, reduce the abemaciclib dose to 100 mg twice daily or, in the case of ketoconazole, reduce the abemaciclib dose to 50 mg twice daily. In patients who have had a dose reduction to 100 mg twice daily due to adverse reactions, further reduce the abemaciclib dose to 50 mg twice daily. Avoid grapefruit or grapefruit juice. If a CYP3A inhibitor is discontinued, increase the abemaciclib dose (after 3-5 half-lives of the inhibitor) to the dose that was used before starting the inhibitor.

Pharmacokinetics

The pharmacokinetics of abemaciclib were characterized in patients with solid tumors, including metastatic breast cancer, and in healthy subjects. Following single and repeated twice daily dosing of 50 mg to 200 mg of abemaciclib, the increase in plasma exposure (AUC) and Cmax was approximately dose proportional. Steady state was achieved within 5 days following repeated twice daily dosing, and the estimated geometric mean accumulation ratio was 2.3 (50% CV) and 3.2 (59% CV) based on C_{max} and AUC, respectively.

Absorption: In the dose range of 50 to 275 mg, abemaciclib absorption is slow, with a T_{max} ranging from 4 to 6 hours, and AUC and C_{max} generally increased in a dose-proportional manner after a single dose and also with multiple twice daily doses. The mean absolute bioavailability of abemaciclib after a single oral dose of 200 mg is 45%.

Effect of food: In a study of the effect of a high-fat meal using the proposed commercial formulation, abemaciclib exposure increased by 27% based on AUC and by 35% based on C_{max}. A high-fat meal did not change the interindividual variability in PK. Although the change in AUC and C_{max} in the food effect study was statistically significant, it was small relative to the variability in exposure in the cancer patient population, and therefore not considered to be clinically relevant. Based on available data, abemaciclib can be taken without regard to meals.

Distribution: In vitro, abemaciclib was bound to human plasma proteins, serum albumin, and alpha-1-acid glycoprotein in a concentration independent manner from 152 ng/mL to 5066 ng/mL.

Metabolism: Hepatic metabolism is the main route of clearance for abemaciclib. Abemaciclib is metabolized to several metabolites primarily by cytochrome P450 (CYP) 3A4, with formation of N-desethylabemaciclib (M2) representing the major metabolism pathway. Additional metabolites include hydroxyabemaciclib (M20), hydroxy-N-desethylabemaciclib (M18), and an oxidative metabolite (M1).

Elimination: The geometric mean apparent oral clearance (CL/F) of abemaciclib was 38.3 L/h (105% CV), and the mean plasma elimination half-life T1/2 for abemaciclib in patients at a dose of 200 mg was 21 (12 – 63) hours. The mean T1/2 ranged from 17 to 38 hours with no consistent trends related to dose, suggesting no dose dependent change in CL.

Excretion: After a single 150 mg oral dose of radiolabeled abemaciclib in healthy subjects, approximately 81% of the dose was recovered in feces and approximately 3.4% recovered in urine. The majority of the dose eliminated in feces was metabolites indicating that abemaciclib is highly metabolized.

Adverse Events

Common known potential toxicities, >10%

Fatigue, diarrhea, nausea, loss of appetite, neutropenia, leukopenia, vomiting, anemia that may require blood transfusion, thrombocytopenia, inflammation or ulcers inside the mouth, alopecia

Occasional known potential toxicities, < 10%

Increased creatinine: Abemaciclib has been shown to increase serum creatinine due to inhibition of renal tubular transporters without affecting glomerular function (as measured by iohexol clearance). In clinical studies, increases in serum creatinine occurred within the first month of abemaciclib dosing, remained elevated but stable through the treatment period, were reversible upon treatment discontinuation, and were not accompanied by changes in markers of renal function, such as blood urea nitrogen (BUN), cystatin C, or calculated glomerular filtration rate based on cystatin C.

Nursing Guidelines

1. Abemaciclib can be taken with or without regard to food. Patients should be instructed to take at the same time each day. Do not crush, chew, or split tabs; they should be taken whole. Patients should be instructed not to take pills that are not intact.
2. Do not make up missed or vomited doses. Instruct patient to take the next dose at its scheduled time.
3. Assess patient’s concomitant medications, including OTC. Patients who are taking strong or moderate CYP3A inhibitors will need to reduce their dose of abemaciclib according to protocol instructions.
4. Gastrointestinal side effects, including diarrhea, nausea, abdominal pain, and vomiting are common. Treat symptomatically and monitor for effectiveness.
5. Monitor CBC w/diff as cytopenias are commonly seen. Instruct patients to report any signs or symptoms of infection, and/or excessive bruising/bleeding to the study team.
6. Increased incidences of VTEs have been seen in patients receiving abemaciclib. Instruct patients to report any swelling of extremities, and /or SOB, DOE or chest pain to the study team immediately and/or seek out emergency medical care.
7. Headache has been seen. Treat symptomatically and monitor for effectiveness.

Investigator Brochure Availability

The investigator brochure for abemaciclib may be obtained by contacting the Alliance Central Protocol Operations Program office at protocols@alliancenctn.org.

11.0 MEASUREMENT OF EFFECT

Response and progression will be evaluated in this study using the new international criteria proposed by MacDonald guidelines.[51] Primary endpoint will be based on local radiology review. Central radiology review will be carried out for measurement of secondary endpoint.

11.1 Schedule of Evaluations:

For the purposes of this study, patients should be reevaluated every 8 weeks while on treatment. In addition to a baseline scan, confirmatory scans should also be obtained 8 weeks following initial documentation of objective response.

Supporting documentation of response should be submitted, per Section 6.1.1.

11.2 Definitions of Measurable and Non-Measurable Disease

11.2.1 Measurable Disease

Bidimensionally measurable lesions with clearly defined margins by MRI scans, with a minimum diameter of 10mm in both dimensions.

For multifocal intracranial disease, no more than 5 target measurable lesions (each >=10mm in diameter in both dimensions) should be selected for measurement. Target lesions should be selected on the basis of their size (lesions with longest diameter), be representative of other lesions and lend themselves to reproducible repeated measurements.

11.2.2 Non-Measurable Disease

Unidimensionally measurable lesions, masses with margins not clearly defined.

11.3 Guidelines for Evaluation of Measurable Disease

11.3.1 Measurement Methods:

- All measurements should be recorded in metric notation (i.e., decimal fractions of centimeters) using a ruler or calipers.
- The same method of assessment and the same technique must be used to characterize each identified and reported lesion at baseline and during follow-up.

11.3.2 Acceptable Modalities for Measurable Disease:

- **Conventional CT and MRI:** This guideline has defined measurability of lesions on CT scan based on the assumption that CT slice thickness is 5 mm or less. If CT scans have slice thickness greater than 5 mm, the minimum size for a measurable lesion should be twice the slice thickness.

  As with CT, if an MRI is performed, the technical specifications of the scanning sequences used should be optimized for the evaluation of the type and site of disease. The lesions should be measured on the same pulse sequence. Ideally, the same type of scanner should be used and the image acquisition protocol should be followed as closely as possible to prior scans. Body scans should be performed with breath-hold scanning techniques, if possible.

11.3.3 Measurement at Follow-up Evaluation:

- A subsequent scan must be obtained every 8 weeks following initial documentation of an objective status of either complete response (CR) or partial response (PR).

- In the case of stable disease (SD), follow-up measurements must have met the SD criteria at least once after study entry at a minimum interval of 8 weeks (see Section 11.4.4).

11.4 Measurement of Treatment/Intervention Effect

11.4.1 Measurable lesions

Bidimensionally enhancing measurable lesions with clearly defined margins by MRI or CT scan. Necrosis or cystic changes (nonenhancing disease) should not be included in the measurement of tumor area.

11.4.2 Non-measurable lesions

Non-measurable sites of disease (Section 11.2.2) should also be recorded at baseline. These lesions should be followed in accord with Section 11.4.3.3.

11.4.3 Response Criteria

11.4.3.1 All measurable lesions followed by CT/MRI must be measured on re-evaluation at evaluation times specified in Section 11.1. Specifically, a change in objective status to either a PR or CR cannot be done without re-measuring measurable lesions.

Note: Non-measurable lesions should be evaluated at each assessment, especially in the case of first response or confirmation of response.

11.4.3.2 Evaluation of Measurable Lesions

Current Response Criteria for Malignant Gliomas (Macdonald Criteria)[51]

Response	Criteria
Complete response	Requires all of the following: complete disappearance of all enhancing measurable and nonmeasurable disease sustained for at least 4 weeks; no new lesions; no corticosteroids; and stable or improved clinically
Partial response	Requires all of the following: ≥ 50% decrease compared with baseline in the sum of products of perpendicular diameters of all measurable enhancing lesions sustained for at least 4 weeks; no new lesions; stable or reduced corticosteroid dose; and stable or improved clinically
Stable disease	Requires all of the following: does not qualify for complete response, partial response, or progression; and stable clinically
Progression	Defined by any of the following: ≥ 25% increase in sum of the products of perpendicular diameters of enhancing lesions; any new lesion; or clinical deterioration

- **Complete Response (CR):** All of the following must be true:
  a. Disappearance of all enhancing lesions on consecutive magnetic resonance imaging (MRI) or CT at least 8 weeks apart. No new lesions. No evidence of non-measurable disease. All measurable and nonmeasurable lesions and sites must be assessed using the same techniques at baseline. Patients must be on no steroids. Neurologically/clinically stable or improved
- **Partial Response (PR):**
  a. ≥ 50% decrease under baseline in the sum of products of perpendicular diameters all enhancing measurable lesions. No progression of nonmeasurable disease. No new lesions. All measurable and non-measurable lesions and sites must be assessed using the same techniques as baseline.

Patients must be on stable or decreasing dose of steroids. Neurologically/clinically stable or improved

- **Progression (PD):** At least one of the following must be true:
  a. ≥ 25% increase in the sum of products of all enhancing measurable lesions over smallest sum observed (over baseline if no decrease) using the same techniques as baseline, OR clear worsening of any nonmeasurable disease, OR appearance of any new lesion/site, OR clear clinical worsening or failure to return for evaluation due to death or deteriorating condition (unless clearly unrelated to this cancer)
  b. >25% increase in the sum of products of all measurable enhancing lesions but < 50% increase in the sum of products of all measurable enhancing lesions AND no new lesions/sites in scans obtained AND no clear clinical deterioration: When all of these conditions are met, the patient may continue on drug for 4 weeks. Imaging must be repeated 4 weeks later (+/- 7 days). If imaging at 4 weeks shows continued growth of greater than 25%, then patient has definitive progression.
- **Stable Disease (SD):** Neither sufficient shrinkage to qualify for PR, nor sufficient increase to qualify for PD taking as reference the MSD. Stable clinically.

11.4.3.3 Evaluation of Non-Measurable Lesions

- **Complete Response (CR):** All of the following must be true:
  a. Disappearance of all measurable lesions.
- **Non-CR/Non-PD:** Persistence of one or more non-measurable lesions.
- **Progression (PD):**

  Unequivocal progression of existing non-measurable lesions

  (NOTE: Equivocal progression of a nonmeasureable lesion should not normally trump change in a measurable lesion. It must be representative of overall disease status change.)

11.4.4 Overall Objective Status

The overall objective status for an evaluation is determined by combining the patient's status on measurable lesions and non-measurable lesions and new disease as defined in the following tables:

For Patients with Measurable Disease

Measurable lesions	Non-measurable lesions	New Sites of Disease	Overall Objective Status
CR	CR	No	CR
CR	Non-CR/Non-PD	No	PR
PR	CR<br>Non-CR/Non-PD	No	PR
CR/PR	Not All Evaluated*	No	PR**
SD	CR<br>Non-CR/Non-PD<br>Not All Evaluated*	No	SD
Not all Evaluated	CR<br>Non-CR/Non-PD<br>Not All Evaluated*	No	Not Evaluated (NE)
PD	Unequivocal PD<br>CR<br>Non-CR/Non-PD<br>Not All Evaluated*	Yes or No	PD
CR/PR/SD/PD/Not all Evaluated	Unequivocal PD	Yes or No	PD
CR/PR/SD/PD/Not all Evaluated	CR<br>Non-CR/Non-PD<br>Not All Evaluated*	Yes	PD

* See Section 11.4.3.1
** See Section 11.4.3.2

11.4.5 Symptomatic neurologic deterioration: Patients with global deterioration of health status requiring discontinuation of treatment without objective evidence of disease progression at that time, and not either related to study treatment or other medical conditions, should be reported as PD due to "symptomatic deterioration." Every effort should be made to document the objective progression even after discontinuation of treatment due to symptomatic deterioration.

A patient is classified as having PD due to "symptomatic deterioration" if any of the following occur that are not either related to study treatment or other medical conditions:

- Worsening of neurologic examination.
- Worsening of tumor-related and/or neurologic symptoms.
- Decline in performance status of >1 level on ECOG scale.
- Increased steroid dosage lasting > 14 days
- Increase in seizure frequency or severity lasting > 14 days

11.5 Definitions of analysis variables

Formal definitions of variables used in analyses can be found in the Statistical Considerations section of the protocol.

12.0 END OF TREATMENT/INTERVENTION

12.1 Duration of Treatment

12.1.1 CR, PR, or SD: Patients who are in CR, PR or SD, as assessed by local radiology review, will continue on therapy until progression of disease, excessive toxicity requiring the patient to come off of treatment, or the patient withdraws from treatment. After treatment is discontinued, patients will be followed per the study calendar in Section 5.0.

12.1.2 Disease Progression: Remove from protocol therapy any patient with disease progression. Document details, including tumor measurements, on data forms.

After disease progression, patients should be followed for survival per the study calendar (Section 5.0).

12.1.3 Discontinuation of study agent: If the patient discontinues study agent, patients should be followed for disease progression and survival per the study calendar (Section 5.0).

12.2 Definitions and Follow-up Requirements

Definition of ineligible patients: A study participant who is registered to the trial but does not meet all of the eligibility criteria is deemed to be ineligible. Patients who are deemed ineligible may continue protocol treatment, provided the treating physician, study chair, and executive officer agree there are no safety concerns if the patient were to continue protocol treatment. Notification of the local IRB may be necessary per local IRB policies.

Definition of clinical follow-up: The follow-up period where the study participant is no longer receiving treatment, but is still following the study calendar for tests, exams, and correlative endpoints (e.g., specimen collection, quality of life, disease assessments as required by the study).

Definition of survival only follow-up: The follow-up period where the study participant is monitored for long-term endpoints, is no longer receiving study treatment, and is not required to follow the study calendar for tests, exams, and correlative endpoints (e.g. specimen collection, quality of life, disease assessments as required by the study). In this follow-up period, there is a schedule in which case report forms should be submitted, but the physician visits are based on the standard of care.

12.2.1 Follow-up for Ineligible Patients

Study participants who are registered to the trial but deemed ineligible must complete follow-up requirements as specified below.

Baseline, on-study and off-treatment notice data submission required.

12.2.2 Follow-up for Patients Never Receiving Protocol Intervention

Study participants who are registered to the trial but who never go on to receive study intervention must still complete follow-up requirements as specified below.

Baseline, on-study and off-treatment notice data submission required.

12.3 Extraordinary Medical Circumstances

If, at any time the constraints of this protocol are detrimental to the patient's health and/or the patient no longer wishes to continue protocol therapy, protocol therapy shall be discontinued. In this event:

- Document the reason(s) for discontinuation of therapy on data forms.

- Follow the patient for protocol endpoints as required by the Study Calendar.

13.0 STATISTICAL CONSIDERATIONS

13.1 Study Design

This is a prospective, one-stage phase 2 study evaluating the efficacy of SMO, FAK, CDK or AKT inhibitors in patients with SMO/PTCH1-mutated, NF2-mutated or AKT1/PIK3CA/PTEN-mutated meningiomas (Grade I-III), respectively. There will be a separate phase 2 arm for each of the four inhibitor agents. Patients harboring SMO/PTCH1, NF2 or AKT1/PIK3CA/PTEN mutations (confirmed by central molecular review) and who meet eligibility criteria will be enrolled. Within each arm defined by tumor mutation and paired inhibitor, there will be up to two different patient cohorts based on histology: grade I versus II/III meningiomas. Below is a table to summarize the analysis cohorts and primary or co-primary endpoints per arm.

Treatment Arm	Eligible Alterations	Cohort(s)	Primary Endpoint(s)
GSK2256098	NF2 (Enrollment Prior to August 2017)	Grade I<br>Grade II/III	• PFS at 6 months (Powered and evaluated separately by grade cohorts)<br>• Response Rate (Powered and evaluated by the entire arm – cohorts are pooled)
Vismodegib	SMO or PTCH1	Grade I<br>Grade II/III	• PFS at 6 months (Powered and evaluated separately by grade cohorts)<br>• Response Rate (Powered and evaluated by the entire arm – cohorts are pooled)
AZD5363	AKT1, PIK3CA, PTEN	Grade I<br>Grade II/III	• PFS at 6 months (Powered and evaluated separately by grade cohorts)<br>• Response Rate (Powered and evaluated by the entire arm – cohorts are pooled)
Abemaciclib	NF2, CDKN2A, CDK4, CDK6, CCND1, CCND2, CCND3, or CCNE1	Grade II/III Only	• PFS at 6 months (Powered and evaluated for a grade II/III cohort only)

			• Response Rate (Powered and evaluated for the grade II/III cohort only)

There are no planned interim analyses. The sample size calculations were done with EAST v6.3 and PASS 15.01.

In Update #06, the grade II/III cohort within NF2 mutation arm – GSK2256098 is expanded to a larger sample size compared to the grade II/III cohort within SMO/PTCH1 mutation – vismodegib or AKT1/PIK3CA/PTEN mutation – AZD5363 arms.

13.2 Statistical Design and Analysis for the Primary Endpoint

13.2.1 Primary endpoints

The primary end points used in this study are progression-free survival at 6 months (PFS6) and response rate (RR). PFS6 is defined as number of patients not having progressive disease or death within six months of the first day of treatment divided by the total number of evaluable patients. RR is defined as the number of responses divided by the total number of evaluable patients. A patient will be deemed to have a response if they have a confirmed PR or CR. Contrast-enhanced cranial magnetic resonance imaging (MRI) will be performed every 8 weeks. Standard response criteria will be used (see Section 11.0).

All patients meeting the eligibility criteria that have signed the consent form and have begun treatment will be considered evaluable for the analysis of primary endpoint. Patients, who do not experience progressive disease but withdraw protocol treatment and re-registered onto the second tumor mutation arm, will not be evaluable for PFS6 endpoint for the efficacy analysis for the first tumor mutation arm. In the event of re-registration of patients from one mutation arm to another, additional sensitivity analyses may be performed.

Safety Analysis (SA) population: All patients who received any quantity of study drug. Patients will be grouped according to treatment received.

13.2.2 Statistical Design

<u>SMO/PTCH1 mutation – Vismodegib arm:</u>

Within the SMO/PTCH1 mutation – vismodegib treatment arm, a total of 24 evaluable patients will provide at least 89% power to detect a true response rate of at least 20%, with a significance level of .021 against the null hypothesis of 2.5% response rate. If at least 3 responses (at least 12.5%) are observed among the 24 evaluable patients, the agent will be considered worthy of further testing in this mutation defined treatment arm.

Within each grade II/III cohort of the SMO/PTCH 1 mutation – vismodegib treatment arm a total of 12 evaluable patients will provide least 89% power to detect a true 6 month PFS rate of at least 55%, with a significance level of .024 against the null hypothesis of 15% 6 month PFS rate. If at least 5 patients (at least 42%) demonstrate 6 month PFS, among the 12 evaluable patients, the agent will be considered worthy of further testing in this mutation defined grade II/III cohort.

Within each grade I cohort of the SMO/PTCH1 mutation – vismodegib treatment arm a total of 12 evaluable patients will provide at least 79% power to detect a true 6 month PFS rate of at least 65%, with a significance level of .014 against the null hypothesis of 25% 6 month PFS rate. If at least 7 patients (at least 58%) demonstrate 6 month PFS, among the

12 evaluable patients, the agent will be considered worthy of further testing in this mutation defined grade I cohort.

NF2 mutation – GSK2256098 arm:

Within NF2 mutation – GSK2256098 treatment arm, a total of 36 evaluable patients will provide at least 94% power to detect a true response rate of at least 20%, with a significance level of .012 against the null hypothesis of 2.5% response rate. If at least 4 responses (at least 11.1%) are observed among the 36 evaluable patients, the agent will be considered worthy of further testing in this mutation defined treatment arm.

Within grade II/III cohort of the NF2 mutation – GSK2256098 treatment arm, a total of 24 evaluable patients will provide least 85% power to detect a true 6 month PFS rate of at least 41.5%, with a significance level of .02 against the null hypothesis of 15% 6 month PFS rate. If at least 8 patients (at least 31.1%) demonstrate 6 month PFS, among the 24 evaluable patients, the agent will be considered worthy of further testing in this mutation defined grade II/III cohort.

Within grade I cohort of the NF2 mutation – GSK2256098 treatment arm, a total of 12 evaluable patients will provide at least 79% power to detect a true 6 month PFS rate of at least 65%, with a significance level of .014 against the null hypothesis of 25% 6 month PFS rate. If at least 7 patients (at least 58%) demonstrate 6 month PFS, among the 12 evaluable patients, the agent will be considered worthy of further testing in this mutation defined grade I cohort

AKT1/PIK3CA/PTEN mutation – AZD5363 arm:

Within the AKT1/PIK3CA/PTEN mutation defined treatment arm a total of 24 evaluable patients will provide at least 89% power to detect a true response rate of at least 20%, with a one-sided significance level of .021 against the null hypothesis of 2.5% response rate. If at least 3 responses (at least 12.5%) are observed among the 24 evaluable patients, the agent will be considered worthy of further testing in this mutation defined treatment arm.

Within each grade II/III cohort of the AKT1/PIK3CA/PTEN mutation defined treatment arm a total of 12 evaluable patients will provide least 89% power to detect a true 6 month PFS rate of at least 55%, with a significance level of .024 against the null hypothesis of 15% 6 month PFS rate. If at least 5 patients (at least 42%) demonstrate 6 month PFS, among the 12 evaluable patients, the agent will be considered worthy of further testing in this mutation defined grade II/III cohort.

Within each grade I cohort of the AKT1/PIK3CA/PTEN mutation defined treatment arm a total of 12 evaluable patients will provide at least 79% power to detect a true 6 month PFS rate of at least 65%, with a significance level of .014 against the null hypothesis of 25% 6 month PFS rate. If at least 7 patients (at least 58%) demonstrate 6 month PFS, among the 12 evaluable patients, the agent will be considered worthy of further testing in this mutation defined grade I cohort.

NF2, CDKx, CCNx mutations Abemaciclib - arm:

Genetic alterations in cyclin-dependent kinases (CDKs) and the cyclins themselves largely co-occur with NF2 mutations. Therefore, within the NF2 mutation –abemaciclib treatment arm and initially independent of any specific cyclin and CDK co-alterations a total of 24 evaluable patients will provide at least 89% power to detect a true response rate of at least 20%, with a significance level of .021 against the null hypothesis of 2.5% response rate. If at least 3 responses (at least 12.5%) are observed among the 24 evaluable patients, the agent will be considered worthy of further testing in this mutation defined treatment arm.

Within grade II/III cohort of the NF2 mutation - abemaciclib treatment arm, a total of 24 evaluable patients will provide least 85% power to detect a true 6 month PFS rate of at least 41.5%, with a significance level of .02 against the null hypothesis of 15% 6 month PFS rate. If at least 8 patients (at least 31.1%) demonstrate 6 month PFS, among the 24 evaluable patients, the agent will be considered worthy of further testing in this mutation defined grade II/III cohort.

There will not be a grade I cohort for this treatment arm.

Overall power and family-wise alpha consideration:

Thus, the trial has 89% to 94% power to detect a promising response rate in the mutation defined treatment groups, 79% power to detect a promising 6 month PFS rate in either mutation defined grade I cohort, and 85% to 89% power to detect a promising 6 month PFS rate in mutation defined grade II/III cohort, with an over-all type I error bound of 19%, against the over-all null hypothesis that neither agent is active with respect to either response rate or PFS.

13.2.3 Analysis Plan

The final analysis will be done after the last evaluable patient had been followed for 6 months from the start of her/his treatment for 6 month PFS endpoint, and after all evaluable patients are off protocol treatment for RR endpoint in each analysis cohorts defined according to mutation status and tumor grade.

The treatment will be considered active within a SMO/PTCH1 mutation – vismodegib group if any of the following occurs:

- There are 3 or more responses observed among the 24 evaluable patients in the treatment arm
- Within the grade II/III cohort of a treatment arm, 5 or more evaluable patients are progression-free at 6 months from treatment initiation out of the 12 evaluable patients
- Within the grade I cohort of a treatment arm, 7 or more evaluable patients are progression-free at 6 months from treatment initiation out of the 12 evaluable patients

The treatment will be considered active within a NF2 mutation – GSK2256098 group if any of the following occurs:

- There are 4 or more responses observed among the 36 evaluable patients in the treatment arm
- Within the grade II/III cohort of a treatment arm, 8 or more evaluable patients are progression-free at 6 months from treatment initiation out of the 24 evaluable patients
- Within the grade I cohort of a treatment arm, 7 or more evaluable patients are progression-free at 6 months from treatment initiation out of the 12 evaluable patients

The treatment will be considered active within a AKT1/PIK3CA/PTEN mutation – AZD5363 group if any of the following occurs:

- There are 3 or more responses observed among the 24 evaluable patients in the treatment arm

- Within the grade II/III cohort of a treatment arm, 5 or more evaluable patients are progression-free at 6 months from treatment initiation out of the 12 evaluable patients
- Within the grade I cohort of a treatment arm, 7 or more evaluable patients are progression-free at 6 months from treatment initiation out of the 12 evaluable patients

The treatment will be considered active within NF2, CDKx, CCNx mutations– abemaciclib group if any of the following occurs:

- There are 3 or more responses observed among the 24 evaluable patients in the treatment arm
- There are 8 or more evaluable patients who are progression-free at 6 months from treatment initiation out of the 24 evaluable patients

In addition, point estimates will be generated for response rates within each treatment arm with corresponding 95% binomial confidence intervals. Point estimates and 95% binomial confidence intervals will also be generated for the six month progression-free survival rate within each cohort of each treatment arm. Kaplan-Meier curves will be generated for progression-free survival for each cohort within each treatment arm. There will be no formal comparison of primary and secondary endpoints among the arms and sub-cohorts of patients.

13.3 Sample size, accrual time and study duration

13.3.1 Sample Size

The study design to be utilized is fully described in Section 13.1.

There will be total of 108 (24 in SMO/PTCH1- vismodegib, 36 in NF2 – GSK2256098, 24 in AKT1/PIK3CA/PTEN – AZD5363, and 24 in NF2 – abemaciclib- arms, respectively) evaluable patients. To reach this accrual goal, we expect to pre-register a maximum of 212 patients for the NF2 mutation arms and a maximum of 843 patients for the SMO/PTCH1 mutation – vismodegib arm, and a maximum of 422 patients for the AKT1/PIK3CA/PTEN mutation – AZD5363 arm. Note, the maximum required number of patients to pre-register is 843 for all arms. These values are based on the current mutation rates within the meningioma population (see Section 1.2) and a conversion rate of 57%.

We anticipate accruing an additional 2 patients in each analysis cohorts of SMO/PTCH1 mutation – vismodegib grade I, SMO/PTCH1 mutation - vismodegib grade II/III, - NF2 mutation – GSK2256098 grade I, AKT1/PIK3CA/PTEN mutation – AZD5363 grade I and AKT1/PIK3CA/PTEN mutation – AZD5363 grade II/III to account for ineligibilities or cancellations. We anticipate accruing an additional 3 patients in NF2 mutation – GSK2256098 grade II/III cohort and NF2, CDKx, CCNx mutations– abemaciclib grade II/III cohort to account for ineligibilities or cancellations. Thus the maximum target accrual is 124 ((12+2)+(12+2)+(24+3)+(12+2)+(12+2)+(12+2)+(24+3) in total. In the event that the additional accrual does not produce the required number of evaluable patients, we retain the option of continuing accrual until that goal is met.

13.3.2 Accrual Rate and Accrual Duration

This phase II study is designed to accrue 24 SMO/PTCH1 mutation, - 60 NF2, and 24 AKT1/PIK3CA/PTEN mutation evaluable patients, over approximately 2-3 years. There is little information available for what the precise accrual rate will be. However our goal is

to accrue the - 108 evaluable patients (with an anticipated maximum of - 124 patients to get - 108 evaluable) within 4 years.

13.3.2 Primary Endpoint Completion Date for Clinical Trials.gov Reporting

For purpose of ClinicalTrail.gov reporting, the Primary Endpoint Completion Date (PECD) for this study is the time the last patient registered has been followed for at least six months.

13.4 Supplementary Analysis plans

Overall survival and progression free-survival will be summarized for each cohort and mutation, separately, within each treatment group with Kaplan-Meier curves and estimates. No formal comparison will be made among the cohorts or mutations of the treatment groups.

Adverse events (AEs) will be summarized for each treatment group and histology grade subgroups if it is applicable. They will be summarized as the number and frequency of each event type and grade. In addition the AEs will be summarized as the number and frequency of patients who experience any AE (including SAEs), AEs of grade 3+, and AEs of grade 4+. This analysis will be purely descriptive.

The response rate determined by central review (including bi-dimensional review for secondary endpoints and volumetric analysis for exploratory aims) will be estimated for each cohort within each treatment group with proportion of patients who achieve CR/PR deemed by central review. The ninety-five percent two-sided confidence intervals will be calculated according to approach of Duffy and Santner. No formal comparison will be made among the cohorts of the treatment groups.

13.5 Monitoring the Study

13.5.1 Adverse Event Stopping Rule

The stopping rule specified below is based on the knowledge available at study development. We note that the Adverse Event Stopping Rule may be adjusted in the event of either (1) the study re-opening to accrual or (2) at any time during the conduct of the trial and in consideration of newly acquired information regarding the adverse event profile of the treatment(s) under investigation. The study team may choose to suspend accrual because of unexpected adverse event profiles that have not crossed the specified rule below.

Accrual will be temporarily suspended to this study if at any time we observe events considered at least possibly related to study treatment (i.e., an adverse event with attribute specified as "possible", "probable", or "definite") that satisfy the following:

if 5 or more patients in the first 20 treated patients (or 25% of all patients after 20 are accrued) experience a grade 4 or higher non-hematologic adverse event.

We note that we will review grade 4 and 5 adverse events deemed "unrelated" or "unlikely to be related", to verify their attribution and to monitor the emergence of a previously unrecognized treatment-related adverse event.

13.5.2 Accrual Monitoring Stopping Rule

The study design assumes that 1-2 patients will accrue per month to each treatment arm, leading to a 2-3 year total accrual period. At 12 months, if the total accrual is below 50% of the expected (fewer than 6 patients) the study team will evaluate with the scientific question will be of interest at the completion of the accrual period. It is deemed that the scientific question is likely not to have relevance at the end of the projected accrual period,

the study may close. Otherwise, the study team will continue to boost the accrual rates. The accrual rate will be checked every subsequent 6 months and the likelihood that the study questions will still be of interest at the end of the trial will be evaluated and based on this, a decision will be made whether or not to continue accrual to the study.

13.6 Study Reporting

13.6.1 This study will be monitored by the study team on a monthly basis upon enrollment of the first patient. Reports containing a summary of adverse events by treatment arm will be reviewed. The study team will also monitor the accrual rate.

13.6.2 This study will be monitored by the Clinical Data Update System (CDUS) version 3.0. Cumulative protocol and patient-specific CDUS data will be submitted electronically to CTEP on a quarterly basis, either by FTP burst of data or via the CDS web application. Reports are due January 31, April 30, July 31, and October 31. Instructions for submitting data using the CDUS can be found on the CTEP Web site (http://ctep.cancer.gov/reporting/cdus.html).

Note: This study has been assigned to CDUS-Abbreviated reporting, no adverse events (routine or expedited) is required to be reported via CDUS.

13.6.3 This study will be monitored by the Food and Drug Administration due to the Investigational New Drug (IND) status of the agent. An IND report will be produced and submitted to the Regulatory Affairs Manager within 60 days of the anniversary date that the IND went into effect**.**

13.6.4 Results Reporting on ClinicalTrials.gov: At study activation, this study will have been registered within the “ClinicialTrails.gov” web site. The Primary and Secondary Endpoints (ie, “Outcome Measures”) along with other required information for this study will be reported on ClinicalTrials.gov.

13.7 Descriptive Factors

As mentioned above, patients will be assigned to a treatment arm based on the mutation status of their tumor. In addition, they will be assigned to a cohort within each treatment arm based on their tumor grade (grade I versus grade II/III). There are not additional descriptive factors.

13.8 Inclusion of Women and Minorities

This study will be available to all eligible patients, regardless of race, gender, or ethnic origin. There is no information currently available regarding differential effects of this regimen in subsets defined by race, gender, or ethnicity, and there is no reason to expect such differences to exist. Therefore, although the planned analysis will, as always, look for differences in treatment effect based on racial and gender groupings, the sample size is not increased in order to provide additional power for subset analyses.

The geographical region served by the Alliance, has a population which includes approximately 18% minorities. Based on national statistics involving similar meningiomas, we expect about 15% of patients will be classified as minorities by race and about 60% of patients will be women. Expected sizes of racial by gender subsets for patients to this study are shown in the following table. Note that these values are for the maximum number of patients (-124) that we expect to register.

**DOMESTIC PLANNED ENROLLMENT REPORT**

Racial Categories	Ethnic Categories				Total
	Not Hispanic or Latino		Hispanic or Latino		
	Female	Male	Female	Male	
American Indian/ Alaska Native	2	1			3
Asian	3	1			4
Native Hawaiian or Other Pacific Islander	1	1			2
Black or African American	5	3			8
White	55	36	9	7	107
More Than One Race					
**Total**	66	42	9	7	124

**Ethnic Categories:**	**Hispanic or Latino –** a person of Cuban, Mexican, Puerto Rican, South or Central American, or other Spanish culture or origin, regardless of race. The term “Spanish origin” can also be used in addition to “Hispanic or Latino.”<br>**Not Hispanic or Latino**
**Racial Categories:**	**American Indian or Alaskan Native –** a person having origins in any of the original peoples of North, Central, or South America, and who maintains tribal affiliations or community attachment.<br>**Asian –** a person having origins in any of the original peoples of the Far East, Southeast Asia, or the Indian subcontinent including, for example, Cambodia, China, India, Japan, Korea, Malaysia, Pakistan, the Philippine Islands, Thailand, and Vietnam. (Note: Individuals from the Philippine Islands have been recorded as Pacific Islanders in previous data collection strategies.)<br>**Black or African American –** a person having origins in any of the black racial groups of Africa. Terms such as “Haitian” or “Negro” can be used in addition to “Black or African American.”<br>**Native Hawaiian or other Pacific Islander –** a person having origins in any of the original peoples of Hawaii, Guam, Samoa, or other Pacific Islands.<br>**White –** a person having origins in any of the original peoples of Europe, the Middle East, or North Africa.

14.0 CORRELATIVE AND COMPANION STUDIES

There are 2 sub-studies within Alliance A071401 (A071401-ST1 and A071401-IM1). The correlative science studies **must be offered** to patients enrolled on A071401. All patients are encouraged to participate.

14.1 Correlative Studies using Biospecimens (Alliance A071401-ST1)

14.1.1 Exploratory Identification of Molecular Biomarkers of Response

14.1.1.1 Background

Understanding the genomic context in which oncogenic mutations occur will be of great importance in this prospective, phase 2 study evaluating the efficacy of SMO , FAK, AKT and CDK inhibitors in patients with SMO/PTCH1-mutated, NF2-mutated or AKT1/PIK3CA/PTEN-mutated meningiomas. Concurrent mutations in other driver oncogenes or tumor suppressors will be critical to evaluate as will the complexity of the genome of these tumors. The presence or absence of additional

genomic changes may influence the behavior of tumor to the proposed therapies. The proposed correlative analyses will allow us to investigate the full extent of genomic aberrations occurring in the study cohort and may allow for improved treatment implementation.

14.1.1.2 Objectives

To support our work on this clinical trial we are currently characterizing two cohorts of patients using a range of genomic technologies in an effort to have a 'database' that will serve as a point of reference and comparison for our clinical trial samples. On these cohorts we are performing high resolution array comparative genomic hybridization arrays, targeted sequencing of over 500 cancer associated genes and genome wide methylation analyses. One cohort contains over 150 patients that have had meningioma resected during the last two years at Brigham and Women's Hospital, and therefore contains meningioma of various histologic subtypes, grade and mutational status (NF2, SMO, PTCH1, PIK3CA, PTEN, AKT1, TRA7, KLF4). The other cohort will contain over 100 samples from grade I and grade II meningioma that have all recurred or progressed. Data from these two well annotated cohorts will provide us with a broad and integrated view of the genomics of meningioma subclassification, grading and prognostics – a view that will serve as a backdrop and a point of reference for comparing genomic aberrations observed in our clinical trial samples.

We propose to perform several genome wide tests on both the primary meningioma resection sample and any samples from recurrent or progressive tumors. The goal is to identify genetic biomarkers that will predict response to therapy. If normal patient DNA is available we will perform whole exome sequencing on these samples. If normal patient DNA is unavailable we will perform targeted sequencing that is limited to roughly 500 cancer-associated genes. In addition, if sufficient DNA is available from the samples, we will perform genome wide methylation profiling. Using the cohorts mentioned above, we currently have plans to compare the reliability of making copy number calls from data acquired using methylation profiling arrays (comparing to the same samples that have been analyzed by aCGH). If methylation profiling data is adequate for making copy number calls, we will only analyze these samples with methylation arrays, otherwise we will collect data from those arrays and from high resolution 1x1M Agilent CGH Microarray chips to confidently identify tumor-specific genomic copy number changes. Starting at screening, and then every other cycle thereafter, we will collect blood to evaluate for circulating DNA in plasma and we will perform targeted sequencing to identify oncogenic mutations.

We will examine this data for genetic biomarkers (chromosome losses, methylation site signatures, combinations of point mutations) to help identify possible biomarkers that will identify those that will respond to therapy and those that will have an aggressive course. We will look for common mutations that could predict resistance to the therapies. When possible and where relevant, tissue sections may be analyzed for immunohistochemical markers to evaluate pathway activation in support of genetic findings. These studies could provide suggestions as to other therapies that might aid tumors that do not respond to SMO, FAK, AKT or CDK inhibition.

14.1.1.3 Methods for Exploratory Correlative Studies

Whole Exome Sequencing and Analyses

Whole exome sequencing will be performed following DNA fragmentation and purification and then library preparation with DNA barcoding. Read pairs will be aligned to the hg19 reference sequence and somatic variant calling will be performed within the Firehose environment and will be annotated to genes and compared to events in the Catalogue of Somatic Mutations in Cancer (COSMIC).

Targeted Sequencing and Analyses

Targeted sequencing of over 500 cancer associated genes will be performed on DNA that has been fragmented and used for library preparation with DNA barcoding. Read pairs will be aligned to the hg19 reference sequence and somatic variant calling will be performed within the Firehose environment and will be annotated to genes and compared to events in the Catalogue of Somatic Mutations in Cancer (COSMIC).

Copy number variation

Array - based comparative genomic hybridization (aCGH) will be performed using the stock 1x1M Agilent SurePrint G3 Human CGH Microarray chip to identify tumor-specific genomic copy number changes. Genomic DNA isolated from the FFPE specimen submitted will be hybridized with genomic DNA isolated from a reference DNA sample representing a pool of karyotypically normal individuals (Promega, Madison, WI). The array platform contains 963,029 probes spaced across the human genome with a 2.1 kb overall median probe spacing and a 1.8 kb probe spacing in RefSeq genes.

Methylation arrays

Genome wide methylation profiling will be performed with Illumina 450K arrays that contain over 450,000 sites targeting all CpG islands and shores, miRNA promoter regions, and disease-associated regions associated with GWAS studies, among other regions. This array represents the most effective and most comprehensive method of direct DNA methylation profiling. This technology uses bisulfite conversion and single base-pair extension to determine a percent methylation at individual CpG sites. It permits for rapid clustering of samples based on the global methylation patterns observed.

Data obtained from Illumina 450K arrays will be averaged (percent methylation) across a given CpG island and clustered using unsupervised hierarchical methods and the most variant probes (~1500). Supervised clustering across subgroups of this disease will also be performed to examine whether subgroup-specific methylation patterns exist.

Integration of data will be performed using the integrated genome viewer from the Broad Institute.

Histopathologic and immunohistochemical analysis

All samples available from this cohort (including primary and recurrent tumors) will be classified by WHO tumor classification criteria for tumor type, subtype and grade and mitoses (highest count per 10 high powered fields) will be evaluated. Histologic features for each of the grading criteria that are used in determining WHO grade will be individually recorded as will the presence of brain invasion. For each sample we will perform a formal count of the Mib1 proliferative index using Aperio digital scanning. We can then analyze the data for histologic correlates of response to therapy.

We will also generate a tissue microarray from all samples with sufficient tissue. The TMA will be used for MIB1 staining, as well as for immunohistochemical staining with markers of PI3K and SMO pathway activation. Our group has demonstrated that immunohistochemical staining with *GAB1* and *STMN1* in meningiomas correlated with *SMO* and *AKT1* mutation status[14].

14.1.1.4 Statistical Considerations

Exploratory analyses on biomarkers will be conducted. Validation of findings in our clinical trial samples can be conducted as needed on archived tissue from meningiomas that have not recurred in over 5-10 years. Because the skull base programs at BWH and MGH are very robust (roughly 40-50 surgeries of skull based meningioma per year), we have ample local resources to compile adequate comparison groups. Our cohort of 150 cases contains over 50 skull based meningioma and we will shortly have an in-depth view of their genomics.

Data obtained from the Illumina 450K arrays will be averaged (percent methylation) across a given CpG island. The most variant ~1500 probes will be used for clustering by unsupervised hierarchical methods. Supervised clustering across subgroups of these samples will also be performed to explore possibly correlations with clinicopathologic parameters. Similar approaches will be used for analysis of aCGH data.

For histologic and immunohistochemical features, as data permits (mitoses, Mib1 profilerative index, etc.), analyses of biomarkers will be summarized by descriptive statistics, including mean, median and standard deviation. Statistical analysis will use Fisher's exact test (association of dichotomous factors), or t-test (comparison of means). Data analysis will be conducted using Prism GraphPad Software and significance will be defined as $P<0.05$.

Differences in these biomarkers between responders and non-responders will be compared with parametric or nonparametric techniques as permitted by the data. For correlative analyses, Cox proportional hazards model will be used to explore the relationship between these biomarkers measured at baseline and PFS and OS. Logistic regression will be used to explore the relationship between these biomarkers measured at baseline and the binary outcome of alive and progression-free at 6 months (APF6)

14.1.2 Circulating tumor DNA (ct-DNA)

14.1.2.1 Background

There is increasing evidence that tumor DNA representing the mutational status of tumor cells can be obtained through the isolation of circulating DNA from blood specimens of patients with cancer[52, 43.] An assay has been developed to identify the major mutations in the *AKT1* gene on the basis of the analysis of circulating tumor DNA (ctDNA) in plasma. Recent analyses have also suggested the feasibility of next-generation sequencing to look more broadly at cancer-specific mutation in ctDNA.

14.1.2.2 Methods

Blood samples will be collected at various timepoints to evaluate oncogenic mutations at baseline and the emergence of new mutations after treatment. Mutations will be evaluated in relevant genes, including but not limited to *AKT1* and *SMO.*

ctDNA will be extracted from plasma samples collected from patients at diagnosis and will be used for the detection of oncogenic mutations using the qRT PCR assays

or other technologies such as next-generation sequencing for meningioma- related oncogenes and tumor suppressors. The prevalence of the mutations measured at baseline and after treatment may provide information on response or resistance to therapy as well as information regarding potential changes in AKT and SMO mutation status during tumor evolution.

14.1.2.3 Statistical Considerations

Exploratory analyses will be conducted. The rates of the different mutations will be summarized by descriptive statistics, the frequency and relative frequency. The agreement with the identification of a mutation in the ctDNA and in tumor tissue will be evaluated using Kappa statistics. Differences in mutation rates between responders and non-responders will be compared with parametric or nonparametric techniques as permitted by the data. Cox proportional hazards model will be used to explore the relationship between the identified mutations and PFS and OS. Logistic regression will be used to explore the relationship between the identified mutations and tumor response status. Finally, the frequency of mutations over time will also be evaluated in a descriptive manner.

14.2 Imaging Biomarkers of Response (A071401-IM1)

14.2.1 Background

We propose to investigate dynamic contrast enhanced (DCE) MRI as an early biomarker of treatment response. Using DCE MRI, physiological parameters related to the tumor vasculature can be calculated. Of particular interest are the parameters K^{trans} which measures vascular permeability and blood vessel surface area and Ve which measures the volume of extravascular extracellular space (the “leakage” space). Meningiomas are known to have high expression of VEGF so have a rich blood supply and avidly enhance on contrast enhanced MRI.[53] In addition, Ve is elevated in meningiomas.[54] Thus, looking at change in tumor vasculature and interstitial space is an intriguing technique to assess meningioma response to therapy. Given that stable disease, i.e. no significant change in meningioma size, is often the best outcome with meningiomas within the first 6-12months of therapy, having an early physiological biomarker of response to any new therapy would be very useful.

Although little data exists in meningiomas, K^{trans} has shown promise in assessing early response to treatment in glioblastoma.[55] Meningiomas are characterized by high vascular permeability that can be measured by DCE MRI and a decrease in K^{trans} has been associated with response to radiation.[56, 57]DCE may also be helpful in distinguishing atypical from typical meningiomas.[52] Thus, we propose to include DCE MRI to determine if change in K^{trans} or Ve can serve as an early physiological biomarker of response.

14.2.2 Objectives

1. To determine if baseline Ktrans or Ve is associated with response.
2. To determine if change in Ktrans or Ve is associated with response.

14.2.3 Methods

For patient who consent to participate, DCE MRI imaging should be performed at sites with such capability. See Appendix III.

DCE MRI will be acquired as part of routine clinical imaging. First, a T1 map of the tissue of interest is created using multiple flip angles (ex. 2, 5, 10, 15, 30 degrees) with a fast gradient echo technique. Dynamic T1-weighted images are then acquired by sampling the

same slab of tissue at a temporal rate of approx. 5 seconds or less at a flip angle of 10 degrees. After acquiring a sufficient number of time points to establish a reliable baseline, a bolus injection of typically 0.1 mMol/kg of gadopentetate-dimeglumine is administered. We continue to acquire dynamic data for 3-5 minutes post contrast injection for a total scan time of 6-10 minutes. Pharmacokinetic modeling of the data is then used to calculate the DCE-based parameters including K^{trans} and Ve.

MRI imaging will be routed from sites to the IROC and then to the Quantitative Tumor Imaging (QTIM) lab for data analysis. Experts in the lab will extract the physiological parameters from the DCE imaging as well as routine measurements such as meningioma volume, volume of FLAIR hyperintensity, and diffusion imaging parameters (ex. ADC). This lab is run by Dr. Elizabeth Gerstner and has expertise in analyzing sophisticated MRI data. QTIM has been responsible for image analysis for multicenter trials including an Adult Brain Tumor Consortium trial in recurrent glioblastoma and a multicenter schwannoma study. Dr. Gerstner is also PI on an ACRIN study looking at FMISO PET and advanced MRI in newly diagnosed glioblastoma so has experience in collaborating on multicenter trials. Furthermore, QTIM has a long track record studying advanced imaging in glioblastoma.[55, 58,59, 60,61]

14.2.4 Statistical Considerations

Exploratory analyses will be conducted. As data permit, analyses of the MRI parameters will be summarized by descriptive statistics, including mean, median and standard deviation. Differences in these parameters between responders and non-responders will be compared with parametric or nonparametric techniques as permitted by the data. For correlative analyses, Cox proportional hazards model will be used to explore the relationship between parameters measured at baseline and PFS and OS. Logistic regression will be used to explore the relationship between parameters measured at baseline and the binary outcome of alive and progression-free at 6 months (APF6).

Overall, the plan is to determine whether the baseline measurements are prognostic for the entire group. We will determine whether there is an association between the baseline measurements and response adjusting for the patient cohort. A priori we do not think that the association between the baseline measurements and response will depend on the type of mutation and treatment. However, if the association between baseline measurements and response are not found to be statistically significantly associated, we will do subgroup analyses to determine if there might be differences in the association between baseline variables and response among the groups. Specifically, we will do perform the analysis for each group separately. We will also do tests for interaction between the cohort group and the baseline variable, though these will likely not have sufficient power due to the small sample sizes.

The overall plan for determining whether a change in the parameters from baseline is associated with response is to first evaluate the association with the change between baseline and first MRI time point (8 weeks into therapy). An additional analysis will be done that uses all the available MRI assessment time points. To account for multiple MRI time points, we will treat the change in MRI from baseline as a time-dependent variable (which could change at each assessment).

15.0 GENERAL REGULATORY CONSIDERATIONS AND CREDENTIALING

None

16.0 REFERENCES

1. Mahmood A, Qureshi NH, Malik GM. Intracranial meningiomas: analysis of recurrence after surgical treatment. Acta Neurochir (Wien) 1994;126:53-8.
2. Mathiesen T, Lindquist C, Kihlstrom L, Karlsson B. Recurrence of cranial base meningiomas. Neurosurgery 1996;39:2-7; discussion 8-9.
3. Perry A, Stafford SL, Scheithauer BW, Suman VJ, Lohse CM. Meningioma grading: an analysis of histologic parameters. Am J Surg Pathol 1997;21:1455-65.
4. Willis J, Smith C, Ironside JW, Erridge S, Whittle IR, Everington D. The accuracy of meningioma grading: a 10-year retrospective audit. Neuropathol Appl Neurobiol 2005;31:141-9.
5. Sughrue ME, Sanai N, Shangari G, Parsa AT, Berger MS, McDermott MW. Outcome and survival following primary and repeat surgery for World Health Organization Grade III meningiomas. J Neurosurg 2010;113:202-9.
6. Rosenberg LA, Prayson RA, Lee J, et al. Long-term experience with World Health Organization grade III (malignant) meningiomas at a single institution. Int J Radiat Oncol Biol Phys 2009;74:427-32.
7. Wen PY, Quant E, Drappatz J, Beroukhim R, Norden AD. Medical therapies for meningiomas. Journal of neuro-oncology 2010;99:365-78.
8. Chamberlain MC, Tsao-Wei DD, Groshen S. Salvage chemotherapy with CPT-11 for recurrent meningioma. Journal of neuro-oncology 2006;78:271-6.
9. Chamberlain MC, Johnston SK. Hydroxyurea for recurrent surgery and radiation refractory meningioma: a retrospective case series. Journal of neuro-oncology 2011;104:765-71.
10. Reardon DA, Norden AD, Desjardins A, et al. Phase II study of Gleevec(R) plus hydroxyurea (HU) in adults with progressive or recurrent meningioma. Journal of neuro-oncology 2012;106:409-15.
11. Chamberlain MC. Hydroxyurea for recurrent surgery and radiation refractory high-grade meningioma. Journal of neuro-oncology 2012;107:315-21.
12. Chamberlain MC, Glantz MJ. Interferon-alpha for recurrent World Health Organization grade 1 intracranial meningiomas. Cancer 2008;113:2146-51.
13. Norden AD, Raizer JJ, Abrey LE, et al. Phase II trials of erlotinib or gefitinib in patients with recurrent meningioma. Journal of neuro-oncology 2010;96:211-7.
14. Brastianos PK, Horowitz PM, Santagata S, et al. Genomic sequencing of meningiomas identifies oncogenic SMO and AKT1 mutations. Nature genetics 2013.
15. Eccles SA, Aboagye EO, Ali S, et al. Critical research gaps and translational priorities for the successful prevention and treatment of breast cancer. Breast Cancer Res 2013;15:R92.
16. Abedalthagafi M, Bi WL, Aizer AA, et al. Oncogenic PI3K mutations are as common as AKT1 and SMO mutations in meningioma. Neuro-oncology 2016;18:649-55.
17. Sahm F, Bissel J, Koelsche C, et al. AKT1E17K mutations cluster with meningothelial and transitional meningiomas and can be detected by SFRP1 immunohistochemistry. Acta neuropathologica 2013;126:757-62.
18. Perry A, Banerjee R, Lohse CM, Kleinschmidt-DeMasters BK, Scheithauer BW. A role for chromosome 9p21 deletions in the malignant progression of meningiomas and the prognosis of anaplastic meningiomas. Brain Pathol 2002;12:183-90.
19. Bostrom J, Meyer-Puttlitz B, Wolter M, et al. Alterations of the tumor suppressor genes CDKN2A (p16(INK4a)), p14(ARF), CDKN2B (p15(INK4b)), and CDKN2C (p18(INK4c)) in atypical and anaplastic meningiomas. Am J Pathol 2001;159:661-9.
20. Peyre M, Salaud C, Clermont-Taranchon E, et al. PDGF activation in PGDS-positive arachnoid cells induces meningioma formation in mice promoting tumor progression in combination with Nf2 and Cdkn2ab loss. Oncotarget 2015;6:32713-22.
21. Goutagny S, Yang HW, Zucman-Rossi J, et al. Genomic profiling reveals alternative genetic pathways of meningioma malignant progression dependent on the underlying NF2 status. Clin Cancer Res 2010;16:4155-64.

22. Rubin LL, de Sauvage FJ. Targeting the Hedgehog pathway in cancer. Nature reviews Drug discovery 2006;5:1026-33.
23. Sekulic A, Migden MR, Oro AE, et al. Efficacy and safety of vismodegib in advanced basal-cell carcinoma. The New England journal of medicine 2012;366:2171-9.
24. Hyman DM, Smyth LM, Donoghue MT, et al. AKT Inhibition in Solid Tumors With AKT1 Mutations. Journal of Clinical Oncology 2017:JCO. 2017.73. 0143.
25. Lee BY, Timpson P, Horvath LG, Daly RJ. FAK signaling in human cancer as a target for therapeutics. Pharmacology & therapeutics 2014.
26. Gupta R, Nalla AK, Gogineni VR, et al. uPAR/cathepsin B overexpression reverse angiogenesis by rescuing FAK phosphorylation in uPAR/cathepsin B down regulated meningioma. PLoS One 2011;6:e17123.
27. Shapiro IM KV, Vidal CM, et al. Merlin deficiency predicts FAK inhibitor sensitivity: a synthetic lethal relationship. Sci Transl Med 2014;6:237ra68.
28. Shah NR, Tancioni I, Ward KK, et al. Analyses of merlin/NF2 connection to FAK inhibitor responsiveness in serous ovarian cancer. Gynecologic oncology 2014;134:104-11.
29. Ingham PW, McMahon AP. Hedgehog signaling in animal development: paradigms and principles. Genes & development 2001;15:3059-87.
30. Evangelista M, Tian H, de Sauvage FJ. The hedgehog signaling pathway in cancer. Clinical cancer research : an official journal of the American Association for Cancer Research 2006;12:5924-8.
31. Chari NS, McDonnell TJ. The sonic hedgehog signaling network in development and neoplasia. Advances in anatomic pathology 2007;14:344-52.
32. Romer J, Curran T. Targeting medulloblastoma: small-molecule inhibitors of the Sonic Hedgehog pathway as potential cancer therapeutics. Cancer research 2005;65:4975-8.
33. Lupi O. Correlations between the Sonic Hedgehog pathway and basal cell carcinoma. International journal of dermatology 2007;46:1113-7.
34. Berman DM, Karhadkar SS, Maitra A, et al. Widespread requirement for Hedgehog ligand stimulation in growth of digestive tract tumours. Nature 2003;425:846-51.
35. Karhadkar SS, Bova GS, Abdallah N, et al. Hedgehog signalling in prostate regeneration, neoplasia and metastasis. Nature 2004;431:707-12.
36. LoRusso PM, Rudin CM, Reddy JC, et al. Phase I trial of hedgehog pathway inhibitor vismodegib (GDC-0449) in patients with refractory, locally advanced or metastatic solid tumors. Clinical cancer research : an official journal of the American Association for Cancer Research 2011;17:2502-11.
37. Von Hoff DD, LoRusso PM, Rudin CM, et al. Inhibition of the hedgehog pathway in advanced basal-cell carcinoma. N Engl J Med 2009;361:1164-72.
38. Erivedge [package insert]. South san Francisco, CA: Genentech, Inc.; 2012.
39. Weiss GJ, Oro AE, Chang ALS, et al. xpanded access study of locally advanced BCC and metastatic BCC patients treated with the Hedgehog-pathway inhibitor vismodegib. ESMO 2012:Abstract 1111PD.
40. Graham RA, Hop CE, Borin MT, et al. Single and multiple dose intravenous and oral pharmacokinetics of the hedgehog pathway inhibitor vismodegib in healthy female subjects. British journal of clinical pharmacology 2012;74:788-96.
41. Davies BR, Greenwood H, Dudley P, et al. Preclinical pharmacology of AZD5363, an inhibitor of AKT: pharmacodynamics, antitumor activity, and correlation of monotherapy activity with genetic background. Molecular cancer therapeutics 2012;11:873-87.
42. Weller M, Roth P, Sahm F, et al. Durable Control of Metastatic AKT1-Mutant WHO Grade 1 Meningothelial Meningioma by the AKT Inhibitor, AZD5363. Journal of the National Cancer Institute 2017;109:djw320.
43. McLean GW, Carragher NO, Avizienyte E, Evans J, Brunton VG, Frame MC. The role of focal-adhesion kinase in cancer - a new therapeutic opportunity. Nat Rev Cancer 2005;5:505-15.
44. Frisch SM, Vuori K, Ruoslahti E, Chan-Hui PY. Control of adhesion-dependent cell survival by focal adhesion kinase. The Journal of cell biology 1996;134:793-9.

45. Liu XJ, Yang L, Wu HB, Qiang O, Huang MH, Wang YP. Apoptosis of rat hepatic stellate cells induced by anti-focal adhesion kinase antibody. World journal of gastroenterology : WJG 2002;8:734-8.
46. Xu LH, Owens LV, Sturge GC, et al. Attenuation of the expression of the focal adhesion kinase induces apoptosis in tumor cells. Cell growth & differentiation : the molecular biology journal of the American Association for Cancer Research 1996;7:413-8.
47. Sloan JA, Liu H, Sargent DJ, et al. A patient-level pooled analysis of the prognostic significance of baseline fatigue for overall survival (OS) among 3,915 patients participating in 43 North Central Cancer Treatment Group (NCCTG) and Mayo Clinic Cancer Center (MC) oncology clinical trials. . Journal of clinical oncology : official journal of the American Society of Clinical Oncology 2009;27:Suppl 507.
48. Tan AD, Novotny PJ, Kaur JS, et al. A patient-level meta-analytic investigation of the prognostic significance of baseline quality of life (QOL) for overall survival (OS) among 3,704 patients participating in 24 North Central Cancer Treatment Group (NCCTG) and Mayo Clinic Cancer Center (MC) oncology clinical trials. Journal of clinical oncology : official journal of the American Society of Clinical Oncology 2008;26:Suppl 9515.
49. Glass P, Brennan T, Wang J, et al. Neurodevelopmental deficits among infants and toddlers with sickle cell disease. J Dev Behav Pediatr 2013;34:399-405.
50. Saunders DP, Epstein JB, Elad S, et al. Systematic review of antimicrobials, mucosal coating agents, anesthetics, and analgesics for the management of oral mucositis in cancer patients. Support Care Cancer 2013;21:3191-207.
51. Macdonald DR, Cascino TL, Schold SC, Jr., Cairncross JG. Response criteria for phase II studies of supratentorial malignant glioma. J Clin Oncol 1990;8:1277-80.
52. Yang S LM, Zagzag D, et al. Dynamic contrast-enhanced perfusion MR imaging measurements of endothelial permeability: differentiation between atypical and typical meningiomas. AJNR Am J Neuroradiol 2003;24:1554-9.
53. Lamszus K LU, Schmidt NO, Stavrou D, Ergun S, Westphal M. Vascular endothelial growth factor, hepatocyte growth factor/scatter factor, basic fibroblast growth factor, and placenta growth factor in human meningiomas and their relation to angiogenesis and malignancy. Neurosurgery 2000;46:938-47
54. Zhu XP LK, Kamaly-Asl ID, et al. Quantification of endothelial permeability, leakage space, and blood volume in brain tumors using combined T1 and T2* contrast-enhanced dynamic MR imaging. J Magn Reson Imaging 2000;11:575-85.
55. Sorensen AG BT, Zhang WT, et al. A "vascular normalization index" as potential mechanistic biomarker to predict survival after a single dose of cediranib in recurrent glioblastoma patients. Cancer research 2009;69:5296-300.
56. Hawighorst H ER, Knopp MV, et al. Intracranial meningeomas: time- and dose-dependent effects of irradiation on tumor microcirculation monitored by dynamic MR imaging. Magn Reson Imaging 1997;15:423-32.
57. Ludemann L GW, Wurm R, Wust P, Zimmer C. Quantitative measurement of leakage volume and permeability in gliomas, meningiomas and brain metastases with dynamic contrast-enhanced MRI. Magn Reson Imaging 2005;23:833-41.
58. Batchelor TT SA, di Tomaso E, et al. AZD2171, a pan-VEGF receptor tyrosine kinase inhibitor, normalizes tumor vasculature and alleviates edema in glioblastoma patients. Cancer Cell 2007;11:83-95.
59. Sorensen AG EK, Polaskova P, et al. Increased survival of glioblastoma patients who respond to antiangiogenic therapy with elevated blood perfusion. Cancer research 2012;72:402-7.
60. Gerstner ER CP, Wen PY, Jain RK, Batchelor TT, Sorensen G. Infiltrative patterns of glioblastoma spread detected via diffusion MRI after treatment with cediranib. Neuro Oncol 2010;12:466-72.
61. Batchelor TT GE, Emblem KE, et al. Improved tumor oxygenation and survival in glioblastoma patients who show increased blood perfusion after cediranib and chemoradiation. Proc Natl Acad Sci U S A 2013;110:19059-64.

APPENDIX I: REGISTRATION FATIGUE/UNISCALE ASSESSMENTS

Registration Fatigue/Uniscale Assessments

At patient registration, this form is to be administered by a nurse/CRA, completed by the patient, and entered into Medidata Rave at the time of registration.

If needed, this appendix can be adapted to use as a source document. A booklet containing this assessment does not exist – please do not order this booklet.

How would you describe:

your level of fatigue, on the average in the past week including today?

0	1	2	3	4	5	6	7	8	9	10
No Fatigue										Fatigue as bad as it can be

your overall quality of life in the past week including today?

0	1	2	3	4	5	6	7	8	9	10
As bad as it can be										As good as it can be

APPENDIX II: REQUIRED CONSENSUS MRI ACQUISITION PARAMETERS

The MRI acquisition protocols defined in the following table have been defined by an international consensus panel. These parameters have been reviewed and adopted by the National Cancer Institute (NCI) and the Federal Drug Administration (FDA), and these acquisition parameters are required for all national and FDA drug-registration trials. The specific acquisition parameters, the sequence of imaging acquisition, and the plane of imaging are all **required** as explicitly stated in these protocols.

For any patients enrolled prior to Update #05 (or with images acquired prior to Update #05), MRI parameters should remain consistent with baseline or prior image acquisition protocols.

1.5T Protocol:

	**Ax FLAIR**	**Ax DWI**	**3D T1 Pre**	**Contrast Injection [a]**	**Ax T2**	**3D T1 Post[b]**
**Sequence**	TSE[c] – (turbo dark fluid)	EPI[f]	MPRAGE[d,e]		TSE[c]	MPRAGE[d,e]
**Plane**	Axial	Axial	Sagittal/Axial		Axial	Sagittal/Axial
**Mode**	2D	2D	3D		2D	3D
**TR [ms]**	>6000	>5000	2100[g]		>3500	2100[g]
**TE [ms]**	100-140	Min	Min		100-120	Min
**TI [ms]**	2200		1100[h]			1100[h]
**Flip Angle**	90/≥160	90/180	10-15		90/180	10-15
**Frequency**	≥256	128	≥172		≥256	≥172
**Phase**	≥256	128	≥172		≥256	≥172
**NEX**	≥1	≥1	≥1		≥1	≥1
**Frequency Direction**	A/P	R/L	A/P		A/P	A/P
**FOV**	240mm	240mm	256mm (for ≤1.5mm isotropic)[j]		240mm	256mm (for ≤1.5mm isotropic)[j]
**Slice Thickness**	≤4mm	≤4mm	≤1.5mm[j]		≤4mm	≤1.5mm[j]
**Gap/Spacing**	0	0	0		0	0
**Diffusion Options[i]**		*b* = 0, 500, and 1000 s/mm^2				
**Parallel Imaging**	Up to 2x	Up to 2x	Up to 2x		Up to 2x	Up to 2x
**Scan Time (Approx)**	4-5 min	3-5 min	5-8 min		3-5 min	5-8 min

[a] 0.1 mmol/kg or up to 20cc (single, full dose) of MR contrast.
[b] Post-contrast 2D axial T1-weighted images should be collected with identical parameters to pre-contrast 2D axial T1-weighted images
[c] TSE = turbo spin echo (Siemens & Philips) is equivalent to FSE (fast spin echo; GE, Hitachi, Toshiba)
[d] MPRAGE = magnetization prepared rapid gradient-echo (Siemens & Hitachi) is equivalent to the inversion recovery SPGR (IR-SPGR or Fast SPGR with inversion activated; GE), 3D turbo field echo (TFE; Philips), or 3D fast field echo (3D Fast FE; Toshiba).
[e] A 3D acquisition without inversion preparation will result in different contrast compared with MPRAGE or another IR-prepped 3D T1-weighted sequences and therefore should be avoided.

[f] In the event of significant patient motion, a radial acquisition scheme may be used (e.g. BLADE [Siemens], PROPELLER [GE], MultiVane [Philips], RADAR [Hitachi], or JET [Toshiba]); however, this acquisition scheme is can cause significant differences in ADC quantification and therefore should be used only if EPI is not an option.
[g] For Siemens and Hitachi scanners. GE, Philips, and Toshiba scanners should use a TR = 5-15ms for similar contrast.
[h] For Siemens and Hitachi scanners. GE, Philips, and Toshiba scanners should use a TI = 400-450ms for similar contrast.
[i] Older model MR scanners that are not capable of >2 *b*-values should use *b* = 0 and 1000 s/mm^2.
[j] FOV and matrix size should be chosen to keep resolution *less than* 1.5mm isotropic voxel size. Note that all voxel measurements should be equal in x, y, and z dimensions.

Acronyms:
Ax = Axial; ADC = apparent diffusion coefficient. FLAIR = fluid attenuated inversion recovery; DWI = diffusion-weighted imaging; 3D = three dimensional; TSE = turbo spin echo; EPI = echo planar imaging; MPRAGE = magnetization prepared rapid gradient-echo; A/P = anterior to posterior; R/L = right to left; NEX = number of excitations or averages; FOV = field of view

3T Protocol:

	Ax FLAIR	Ax DWI	3D T1 Pre	Contrast Injection [a]	Ax T2	3D T1 Post[b]
**Sequence**	TSE[c] – (turbo dark fluid)	EPI[f]	MPRAGE[d,e]		TSE[c]	MPRAGE[d,e]
**Plane**	Axial	Axial	Axial/Sagittal		Axial	Axial/Sagittal
**Mode**	2D	2D	3D		2D	3D
**TR [ms]**	>6000	>5000	2100[g]		>2500	2100[g]
**TE [ms]**	100-140	Min	Min		80-120	Min
**TI [ms]**	2500		1100[h]			1100[h]
**Flip Angle**	90/≥160	90/180	10-15		90/≥160	10-15
**Frequency**	≥256	128	256		≥256	256
**Phase**	≥256	128	256		≥256	256
**NEX**	≥1	≥1	≥1		≥1	≥1
**Frequency Direction**	A/P	R/L	A/P		A/P	A/P
**FOV**	240mm	240mm	256mm (for 1mm isotropic)[i]		240mm	256mm (for 1mm isotropic) [i]
**Slice Thickness**	3mm	3mm	1mm[i]		3mm	1mm[i]
**Gap/Spacing**	0	0	0		0	0
**Diffusion Options**		*b* = 0, 500, and 1000 s/mm^2				
**Parallel Imaging**	Up to 2x	Up to 2x	Up to 2x		Up to 2x	Up to 2x
**Scan Time (Approx)**	4-5 min	3-5 min	5-8 min		3-5 min	5-8 min

[a] 0.1 mmol/kg or up to 20cc (single, full dose) of MR contrast.

[b] Post-contrast 3D axial T1-weighted images should be collected with identical parameters to pre-contrast 3D axial T1-weighted images

[c] TSE = turbo spin echo (Siemens & Philips) is equivalent to FSE (fast spin echo; GE, Hitachi, Toshiba)

[d] MPRAGE = magnetization prepared rapid gradient-echo (Siemens & Hitachi) is equivalent to the inversion recovery SPGR (IR-SPGR or Fast SPGR with inversion activated; GE), 3D turbo field echo (TFE; Philips), or 3D fast field echo (3D Fast FE; Toshiba).

[e] A 3D acquisition without inversion preparation will result in different contrast compared with MPRAGE or another IR-prepped 3D T1-weighted sequences and therefore should be avoided.

[f] In the event of significant patient motion, a radial acquisition scheme may be used (e.g. BLADE [Siemens], PROPELLER [GE], MultiVane [Philips], RADAR [Hitachi], or JET [Toshiba]); however, this acquisition scheme is can cause significant differences in ADC quantification and therefore should be used only if EPI is not an option.

[g] For Siemens and Hitachi scanners. GE, Philips, and Toshiba scanners should use a TR = 5-15ms for similar contrast.

[h] For Siemens and Hitachi scanners. GE, Philips, and Toshiba scanners should use a TI = 400-450ms for similar contrast.

[i] FOV and matrix size should be chosen to keep resolution at 1mm isotropic voxel size. Note that all voxel measurements should be equal in x, y, and z dimensions.

<u>Acronyms:</u>
Ax = Axial; ADC = apparent diffusion coefficient. FLAIR = fluid attenuated inversion recovery; DWI = diffusion-weighted imaging; 3D = three dimensional; TSE = turbo spin echo; EPI = echo planar imaging; MPRAGE = magnetization prepared rapid gradient-echo; A/P = anterior to posterior; R/L = right to left; NEX = number of excitations or averages; FOV = field of view

APPENDIX III: 1.5T & 3T ADVANCED MRI PROTOCOL FOR SITES ACQUIRING DCE IMAGING

1.5T ADVANCED MRI PROTOCOL FOR SITES ACQUIRING DCE IMAGING

	**Ax FLAIR**[j]	**Ax DWI**	**Ax T2**[h,i]	**3D T1 Pre**[b]	**T1 Map**	*Contrast Injection*[a]	**DCE**[a]	**3D T1w Post**[b]
Sequence	TSE[c] (turbo dark fluid)	EPI[g]	TSE[c]	MPRAGE [e,f]	3D-FLASH[d]		3D-FLASH[d]	MPRAGE [e,f]
Plane	Axial	Axial	Axial	Sagittal/ Axial	Axial		Axial	Sagittal/ Axial
Mode	2D	2D	2D	3D	3D		3D	3D
TR [ms]	>6000	>5000	>3500	2100[m]	8.7		8.7	2100[m]
TE [ms]	100-140	Min	80-120	Min	Min		Min	Min
TI [ms]	2200			1100[n]				1100[n]
Flip Angle [Degrees]	90/≥160	90/180	90/≥160	10-15	5/10/15 /30		24	10-15
Frequency	≥256	128	≥256	≥172	128		128	≥172
Phase	≥256	128	≥256	≥172	84		84	≥172
NEX	≥1	≥1	≥1	≥1	Average =4, 1 Rep.		Average=1 (~ 71 Reps; ~6sec/rep; 60 sec baseline)	≥1
Frequency Direction	A/P	R/L	A/P	A/P	R/L		R/L	A/P
FOV	240	240	240	256	256 X 208		256 X 208	256
Slice Thickness	≤4mm[l]	≤4mm[l]	≤4mm[l]	≤1.5mm	4mm		4mm	≤1.5mm
Gap/Spacing	0	0	0	0	0		0	0
Diffusion Options[p]		*b* = 0, 500, 1000 s/mm^2						
Parallel Imaging	Up to 2x	Up to 2x	Up to 2x	Up to 2x	Yes- acceleration factor = 2		Yes- acceleration factor = 2	Up to 2x
Scan Time	4-5 min	3-5	3-5 min	5-8 min	< 30 sec ea		7 min	5-8 min
**Slices**					28		28	

[a] After 60 seconds of baseline image acquisition, a bolus injection of 0.1 mMol/kg of Gadolinium chelated contrast agent is administered as part of the DCE acquisition. Continue to acquire dynamic data for 6 minutes post contrast injection for a total scan time of 7 minutes. Use of a power injector is desirable at an injection rate of 3.5 mL/sec, followed by 20-mL saline flush of the same rate.
[b] Post-contrast 3D T1-weighted images should be collected with equivalent parameters to pre-contrast 3D T1-weighted images
[c] TSE = turbo spin echo (Siemens & Philips) is equivalent to FSE (fast spin echo; GE, Hitachi, Toshiba)
[d] FLASH = fast low angle shot (FLASH; Siemens) is equivalent to the spoil gradient recalled echo (SPGR; GE) or T1- fast field echo (FFE; Philips), fast field echo (FastFE; Toshiba), or the radiofrequency

spoiled steady state acquisition rewound gradient echo (RSSG; Hitachi). A fast gradient echo sequence without inversion preparation is desired.
[e] MPRAGE = magnetization prepared rapid gradient-echo (Siemens & Hitachi) is equivalent to the inversion recovery SPGR (IR-SPGR or Fast SPGR with inversion activated or BRAVO; GE), 3D turbo field echo (TFE; Philips), or 3D fast field echo (3D Fast FE; Toshiba).
[f] A 3D acquisition without inversion preparation will result in different contrast compared with MPRAGE or another IR-prepped 3D T1-weighted sequences and therefore should be avoided.
[g] In the event of significant patient motion, a radial acquisition scheme may be used (e.g. BLADE [Siemens], PROPELLER [GE], MultiVane [Philips], RADAR [Hitachi], or JET [Toshiba]); however, this acquisition scheme is can cause significant differences in ADC quantification and therefore should be used only if EPI is not an option. Further, this type of acquisition takes considerable more time.
[h] Dual echo PD/T2 TSE is optional for possible quantification of tissue T2.
[i] Additional sequences can be substituted into this time slot, so long as 3D post-contrast T1-weighted images are collected between 4 and 8 min after contrast injection.
[j] 3D FLAIR is an optional alternative to 2D FLAIR, with sequence parameters as follows per EORTC guidelines: 3D TSE/FSE acquisition; TE=90-140ms; TR=6000-10000ms; TI=2000-2500ms (chosen based on vendor recommendations for optimized protocol and field strength); GRAPPA≤2; Fat Saturation; Slice thickness ≤ 1.5mm; Orientation Sagittal or Axial; FOV ≤ 250 mm x 250 mm; Matrix ≥ 244x244.
[k] Choice of TI should be chosen based on the magnetic field strength of the system (e.g. TI ≈ 2000ms for 1.5T and TI ≈ 2500ms for 3T).
[l] In order to ensure comparable SNR older 1.5T MR systems can use contiguous (no interslice gap) images with 5mm slice thickness or increase NEX for slice thickness ≤4mm.
[m] For Siemens and Hitachi scanners. GE, Philips, and Toshiba scanners should use a TR = 5-15ms for similar contrast.
[n] For Siemens and Hitachi scanners. GE, Philips, and Toshiba scanners should use a TI = 400-450ms for similar contrast.
[p] Older model MR scanners that are not capable of >2 *b*-values should use b = 0 and 1000 s/mm^2.

Acronyms:
Ax = Axial; ADC = apparent diffusion coefficient. FLAIR = fluid attenuated inversion recovery; DWI = diffusion-weighted imaging; 3D = three dimensional; TSE = turbo spin echo; EPI = echo planar imaging; SS-EPI = single-shot echo planar imaging; GE-EPI = gradient echo planar imaging; 2DFL = two-dimensional FLASH (fast low angle shot) gradient recalled echo; MPRAGE = magnetization prepared rapid gradient-echo; A/P = anterior to posterior; R/L = right to left; NEX = number of excitations or averages; FOV = field of view; TE = echo time; TR = repetition time; TI = inversion time; PD = proton density

3T ADVANCED MRI PROTOCOL FOR SITES ACQUIRING DCE IMAGING

	**Ax FLAIR**[j]	**Ax DWI**	**Ax T2**[h,i]	**3D T1 Pre**[b]	**T1 Map**	*Contrast Injection* [a]	**DCE**[a]	**3D T1 Post**[b]
Sequence	TSE[c] (turbo dark fluid)	EPI[g]	TSE[c]	MPRAGE[e, f]	3D-FLASH[d]		3D-FLASH[d]	MPRAGE[e, f]
Plane	Axial	Axial	Axial	Axial/ Sagittal/	Axial		Axial	Axial/ Sagittal
Mode	2D	2D	2D	3D	3D		3D	3D
TR [ms]	>6000	>5000	>2500	2100[m]	5.6		5..6	2100[m]
TE [ms]	100-140	Min	80-120	Min	Min		Min	Min
TI [ms]	2500			1100[n]				1100[n]
Flip Angle [Degrees]	90/≥160	90/180	90/≥160	10-15	5/10/15 /30		24	10-15
Frequency	≥256	128	≥256	256	128		128	256
Phase	≥256	128	≥256	256	62		62	256
NEX	≥1	≥1	≥1	≥1	Average =4, 1 Rep.		Average=1 (98 Reps; 4.3sec/rep; 60 sec baseline)	≥1
Frequency Direction	A/P	R/L	A/P	A/P	R/L		R/L	A/P
FOV	240	240	240	256 (for 1mm isotropic)	256 X 208		256 X 208	256 (for 1mm isotropic)
Slice Thickness	3mm[l]	3mm[l]	3mm[l]	1mm	3mm		3mm	1mm
Gap/Spacing	0	0	0	0	0		0	0
Diffusion Options		*b* = 0, 500, 1000 s/mm^2 ≥3 directions						
Parallel Imaging	Up to 2x	Up to 2x	Up to 2x	Up to 2x	Yes- acceleratio n factor = 2		Yes- acceleration factor = 2	Up to 2x
Scan Time (Approx)	4-5 min	3-5 min	3-5 min	5-8 min	< 30 sec ea		7 min	5-8 min
**Slice**					36		36	

[a] After 60 seconds of baseline image acquisition, a bolus injection of 0.1 mMol/kg of Gadolinium chelated contrast agent is administered as part of the DCE acquisition. Continue to acquire dynamic data for 6 minutes post contrast injection for a total scan time of 7 minutes. Use of a power injector is desirable at an injection rate of 3.5cc/sec, followed by 20-mL saline flush of the same rate.
[b] Post-contrast 3D T1-weighted images should be collected with equivalent parameters to pre-contrast 3D T1-weighted images
[c] TSE = turbo spin echo (Siemens & Philips) is equivalent to FSE (fast spin echo; GE, Hitachi, Toshiba)
[d] FLASH = fast low angle shot (FLASH; Siemens) is equivalent to the spoil gradient recalled echo (SPGR; GE) or T1- fast field echo (FFE; Philips), fast field echo (FastFE; Toshiba), or the radiofrequency spoiled steady state acquisition rewound gradient echo (RSSG; Hitachi). A fast gradient echo sequence without inversion preparation is desired.
[e] MPRAGE = magnetization prepared rapid gradient-echo (Siemens & Hitachi) is equivalent to the inversion recovery SPGR (IR-SPGR or Fast SPGR with inversion activated or BRAVO; GE), 3D turbo field echo (TFE; Philips), or 3D fast field echo (3D Fast FE; Toshiba).
[f] A 3D acquisition without inversion preparation will result in different contrast compared with MPRAGE or another IR-prepped 3D T1-weighted sequences and therefore should be avoided.
[g] In the event of significant patient motion, a radial acquisition scheme may be used (e.g. BLADE [Siemens], PROPELLER [GE], MultiVane [Philips], RADAR [Hitachi], or JET [Toshiba]); however, this acquisition scheme is can cause significant differences in ADC quantification and therefore should be used only if EPI is not an option. Further, this type of acquisition takes considerable more time.
[h] Dual echo PD/T2 TSE is optional for possible quantification of tissue T2.
[i] Additional sequences can be substituted into this time slot, so long as 3D post-contrast T1-weighted images are collected between 4 and 8 min after contrast injection.
[j] 3D FLAIR is an optional alternative to 2D FLAIR, with sequence parameters as follows per EORTC guidelines: 3D TSE/FSE acquisition; TE=90-140ms; TR=6000-10000ms; TI=2000-2500ms (chosen based on vendor recommendations for optimized protocol and field strength); GRAPPA≤2; Fat Saturation; Slice thickness ≤ 1.5mm; Orientation Sagittal or Axial; FOV ≤ 250 mm x 250 mm; Matrix ≥ 244x244.

[m] For Siemens and Hitachi scanners. GE, Philips, and Toshiba scanners should use a TR = 5-15ms for similar contrast.
[n] For Siemens and Hitachi scanners. GE, Philips, and Toshiba scanners should use a TI = 400-450ms for similar contrast.

Acronyms:
Ax = Axial; ADC = apparent diffusion coefficient. FLAIR = fluid attenuated inversion recovery; DWI = diffusion-weighted imaging; 3D = three dimensional; TSE = turbo spin echo; EPI = echo planar imaging; SS-EPI = single-shot echo planar imaging; GE-EPI = gradient echo planar imaging; 2DFL = two-dimensional FLASH (fast low angle shot) gradient recalled echo; MPRAGE = magnetization prepared rapid gradient-echo; A/P = anterior to posterior; R/L = right to left; NEX = number of excitations or averages; FOV = field of view; TE = echo time; TR = repetition time; TI = inversion time; PD = proton density

Sequence and Parameter Justification:

BASIC STANDARD PROTOCOL

1) Pre and Post-Contrast 3D T1-Weighted MPRAGE
    a. Recommended sequence from ADNI
    b. Available from all major MRI vendors
    c. EORTC and ACRIN approved sequence
    d. Allows for volumetric estimations of enhancing tumor volume
    e. Allows for calculation of T1 subtraction map-defined enhancing tumor volume
    f. Allows for longitudinal registration of MR scans in the same patient over time
2) T2-Weighted Turbo Spin Echo (TSE) (Optional: Dual Echo PD/T2 TSE)
    a. Available from all major MRI vendors
    b. EORTC and ACRIN approved sequence
    c. Recommended sequence from ADNI (dual echo)
    d. Dual echo may allow for quantitative estimation of "effective" T2 relaxation rate
    e. $T2^{eff}$ has been shown to be sensitive to various pathologies, including AD, TBI, MCI, stroke, MS, psychiatric diseases, etc.
    f. Allows for current RANO evaluations
    g. Dual echo may allow for objective definition of non-enhancing tumor based on $T2^{eff}$
3) Axial T2-Weighted FLAIR
    a. Allows for increased sensitivity for T2 abnormalities
    b. EORTC and ACRIN approved sequence
    c. Allows for RANO evaluations
4) Axial Diffusion Weighted Imaging (DWI)
    a. EORTC and ACRIN approved sequence
    b. Choice of *b*-values and acquisition parameters are in compliance with the recommendations from the NCI-ISMRM consensus meeting for DWI as a cancer biomarker.

OPTIONAL VARIATIONS TO PROTOCOL

These optional sequences are included as they provide more detail on anatomy (FLAIR) or tissue cellularity (DTI) than the 2D FLAIR and DWI sequences but are not always available on every scanner.

5) 3D FLAIR
    a. EORTC approved (optional) sequence
    b. Allows for 1mm isotropic FLAIR
    c. Experimental & may not be available on all MR systems
6) Diffusion Tensor Imaging (DTI)
    a. Advanced modification to the standard DWI sequence
    b. Allows for measures of "diffusion anisotropy", or fractional anisotropy (FA), which has been shown to correlate with cellularity and response to therapy
    c. Routinely used for pre-surgical planning
    d. Can be used to estimate "fiber tracts" via DT tractography
    e. DTI sequences with a high number of directions are important for accurately estimating low FA, which is of interest for tumors

APPENDIX IV: PATIENT MEDICATION DIARIES

The following medication diaries are included within this appendix:

Appendix IV-A: Patient medication diary – vismodegib

Appendix IV-B: Patient medication diary – GSK2256098

Appendix IV-C: Patient medication diary – AZD5363

Appendix IV-D: Patient medication diary – abemaciclib

Appendix IV-A: Patient medication diary - Vismodegib

INSTRUCTIONS TO THE PATIENT:

1. Complete one form for each 4 week-period while you take **vismodegib**.
2. You will take your dose of **vismodegib** daily.
3. Record the date, the number of capsules you took, and when you took them. Record doses as soon as you take them; do not batch entries together at a later time.
4. If a dose is missed, do not make up that dose; resume dosing with the next scheduled dose.
5. Capsules should not be opened or crushed.
6. If you have any comments or notice any side effects, please record them in the Comments column. If you make a mistake while you write, please cross it out with one line, put your initials next to it, and then write the corrected information next to your initials. Example: - SB 9:30 am
7. Please return this form to your physician when you go for your next appointment.

Day	Date	Time of daily dose	# of capsules taken	Comments
1				
2				
3				
4				
5				
6				
7				
8				
9				
10				
11				
12				
13				
14				
15				
16				
17				
18				
19				
20				
21				
22				
23				
24				
25				
26				
27				
28				

Patient's Signature	Date
**Physician's Office will complete this section:**<br>1. Date patient started protocol treatment ______<br>2. Date patient was removed from study ______<br>3. Total number of capsules taken this month (each size) ______<br>4. Physician/Nurse/Data Manager's Signature ______	

Appendix IV-B: Patient medication diary - GSK2256098

INSTRUCTIONS TO THE PATIENT:

1. Complete one form for each 4 week-period while you take **GSK2256098**.
2. You will take your dose of **GSK2256098** twice daily.
3. Record the date, the number of capsules you took, and when you took them. Record doses as soon as you take them; do not batch entries together at a later time.
4. If a dose is missed, do not make up that dose; resume dosing with the next scheduled dose.
5. Capsules should not be opened or crushed.
6. If you have any comments or notice any side effects, please record them in the Comments column. If you make a mistake while you write, please cross it out with one line, put your initials next to it, and then write the corrected information next to your initials. Example: - SB 9:30 am
7. Please return this form to your physician when you go for your next appointment.

Monitor bilirubin and instruct patients to report any jaundice symptoms to the study team immediately. Patients should be instructed to avoid direct sunlight or UV exposure. Patients should be instructed on the use of protective clothing, sun glasses and sunscreen

Day	Date	Time of AM dose	# of capsules taken	Time of PM dose	# of capsules taken	Comments
1						
2						
3						
4						
5						
6						
7						
8						
9						
10						
11						
12						
13						
14						
15						
16						
17						
18						
19						
20						
21						
22						
23						
24						
25						

26						
27						
28						
Patient's Signature					Date	

	**Physician's Office will complete this section:**<br><br>1. Date patient started protocol treatment ______________________<br><br>2. Date patient was removed from study ______________________<br><br>3. Total number of capsules taken this month (each size) ______________________<br><br>4. Physician/Nurse/Data Manager's Signature<br><br>______________________

Appendix IV-C: Patient medication diary – AZD5363

INSTRUCTIONS TO THE PATIENT:

1. Complete one form for each 4 week-period while you take **AZD5363.**
2. You will take your dose of **AZD5363** twice daily for four days, for the next three days, you will not take any AZD5363. You will repeat 4 days on/3 days off for the 4 week-period. If possible, take the tablets at approximately the same times each day, with water in a fasted state from at least 2 hours prior to the dose to at least 1 hour post-dose.
3. Record the date, the number of tablets you took, and when you took them. Record doses as soon as you take them; do not batch entries together at a later time.
4. If a dose is missed, do not make up that dose; resume dosing with the next scheduled dose. If you vomit after taking the capsule, do not take another capsule until your next scheduled dose.
5. Tablets should not be crushed.
6. If you have any comments or notice any side effects, please record them in the Comments column. If you make a mistake while you write, please cross it out with one line, put your initials next to it, and then write the corrected information next to your initials. Example: SB 9:30 am
7. Please return this form to your physician when you go for your next appointment.

Day	Date	Time of AM dose	# of tablets taken	Time of PM dose	# of tablets taken	Comments
1						
2						
3						
4						
5						
6		Do not take AZD5363 on Days 5, 6 and 7.				
7						
8						
9						
10						
11						
12						
13		Do not take AZD5363 on Days 12, 13 and 14.				
14						
15						
16						
17						
18						
19						
20		Do not take AZD5363 on Days 19, 20 and 21.				
21						
22						

23						
24						
25						
26		Do not take AZD5363 on Days 26. 27 and 28.				
27						
28						
Patient's Signature					Date	

Physician's Office will complete this section:

1. Date patient started protocol treatment ______________________________

2. Date patient was removed from study ______________________________

3. Total number of tablets taken this month (each size) ______________________________

4. Physician/Nurse/Data Manager's Signature

____

Appendix IV-D: Patient medication diary – <u>Abemaciclib</u>

INSTRUCTIONS TO THE PATIENT:

1. Complete one form for each 4 week-period while you take **Abemaciclib**.
2. You will take your dose of **Abemaciclib** twice daily.
3. Record the date, the number of capsules you took, and when you took them. Record doses as soon as you take them; do not batch entries together at a later time.
4. If a dose is missed, do not make up that dose; resume dosing with the next scheduled dose.
5. Capsules should not be opened or crushed.
6. If you have any comments or notice any side effects, please record them in the Comments column. If you make a mistake while you write, please cross it out with one line, put your initials next to it, and then write the corrected information next to your initials. Example: ~~10:30 am~~ SB 9:30 am
7. Please return this form to your physician when you go for your next appointment.

Day	Date	Time of daily dose 1	# of capsules taken	Time of daily dose 2	# of capsules taken	Comments
1						
2						
3						
4						
5						
6						
7						
8						
9						
10						
11						
12						
13						
14						
15						
16						
17						
18						
19						
20						
21						
22						
23						
24						
25						
26						

27						
28						
Patient's Signature						Date

Physician's Office will complete this section:

1. Date patient started protocol treatment ______________________________

2. Date patient was removed from study ______________________________

3. Total number of capsules taken this month (each size) ______________________________

4. Physician/Nurse/Data Manager's Signature ______________________________

APPENDIX V: CONCOMITANT MEDICATIONS PROHIBITED OR FOR USE WITH CAUTION WITH AZD5363

CONCOMITANT MEDICATION

Drugs affecting CYP3A4 metabolism that AstraZeneca strongly recommend are not combined with AZD5363

There are currently no data confirming that there are any pharmacokinetic (PK) interactions between any agents and AZD5363. The potential interactions detailed below are considered on the basis of the preclinical data only. The following lists are not intended to be exhaustive, and a similar restriction will apply to other agents that are known to strongly modulate CYP3A4 activity. Appropriate medical judgment is required. Please contact AstraZeneca with any queries you have on this issue.

Table 1 Strong CYP3A4 inhibitors that may increase exposure to AZD5363 more than 5-fold

Ketoconazole<br>Protease inhibitors (danoprevir, ritonavir, saquinavir, indanavir, tapranavir, telaprevir, elvitegravir, lopinavir, nelfinavir, bocepravir)<br>Cobicistat<br>Conivaptan<br>Nefazodone<br>Mebefradil	Minimum of 2 weeks washout prior to AZD5363 administration and for 2 weeks following discontinuation of AZD5363
Itraconazole<br>Posaconazole<br>Voriconazole<br>Clarithromycin<br>Telithromycin<br>Troleandomycin	Minimum of 1 week washout prior to AZD5363 administration and for 2 weeks following discontinuation of AZD5363

Table 2 Potent Inducers of CYP3A4 that may reduce exposure to AZD5363 by more than 5-fold

Phenobarbital<br>Carbamazepine<br>Phenytoin<br>Rifampicin<br>Rifabutin<br>Mitotane<br>Enzalutamide	Minimum of 2 weeks washout prior to AZD5363 administration and for 2 weeks following discontinuation of AZD5363
St John’s Wort	Minimum of 3 weeks washout prior to AZD5363 administration and for 2 weeks following discontinuation of AZD5363

Drugs affecting CYP3A4 metabolism that AstraZeneca considers may be allowed with caution.

Table 3 Moderate Inhibitors of CYP3A4 that may increase exposure to AZD5363

Diltiazem<br>Verapamil<br>Erythromycin<br>Fluconazole<br>Aprepitant	Drugs are permitted but caution should be exercised and patients monitored closely for possible drug interactions. Please refer to full prescribing information for all drugs prior to co-administration with AZD5363.
Grapefruit juice<br>Seville oranges (and other products containing Seville oranges)	Patients should abstain from eating large amounts of grapefruit and Seville oranges (and other products containing these fruits eg, grapefruit juice or marmalade) during the study (e.g., no more than a small glass of grapefruit juice (120 mL) or half a grapefruit or 1-2 teaspoons (15 g) of Seville orange marmalade daily).

Medicines that are significantly metabolised by CYP3A4 that AstraZeneca strongly recommend are not combined with AZD5363

There are currently no data confirming that there are any pharmacokinetic (PK) interactions between AZD5363 and the following CYP3A4 substrates. The potential interactions detailed below are considered on the basis of the preclinical data only. The following list is not intended to be exhaustive, and a similar restriction will apply to other agents that are known to be sensitive to CYP3A4 inhibitors. Appropriate medical judgment is required. Please contact AstraZeneca with any queries you have on this issue.

Table 4 Exposure, pharmacological action and toxicity that may be increased by inhibition of CYP3A4 by AZD5363

Alfentanil<br>Cyclosporin<br>Diergotamine<br>Ergotamine<br>Fentanyl<br>Sirolimus<br>Tacrolimus<br>Atorvastatin<br>Lovastatin<br>Simvastatin<br>Cerivastatin	Minimum of 1 week washout prior to AZD5363 administration and for 2 weeks following discontinuation of AZD5363
Carbamazepine	Minimum of 2 weeks washout prior to AZD5363 administration and for 2 weeks following discontinuation of AZD5363.

Medicines that are significantly metabolised by CYP3A4 that AstraZeneca considers may be allowed with caution

Table 5 Exposure, pharmacological action and toxicity that may be increased by inhibition of CYP3A4 by AZD5363

Erythromycin<br>Trazodone<br>Tamoxifen<br>Alprazolam<br>Midazolam<br>Triazolam<br>Felodipine<br>Isradipine<br>Nifedipine<br>Methylprednisolone<br>Pimozide<br>Quinidine<br>Domperidone	Drugs are permitted but caution should be exercised and patients monitored closely for possible drug interactions. Please refer to full prescribing information for all drugs prior to co-administration with AZD5363.

Agents that are sensitive to CYP2D6 inhibition that AstraZeneca strongly recommends are not combined with AZD5363

There are currently no data confirming that there are any pharmacokinetic (PK) interactions between AZD5363 and the following CYP2D6 substrates. The potential interactions detailed below are considered on the basis of the preclinical data only. The following list is not intended to be exhaustive, and a similar restriction will apply to other agents that are known to be sensitive to CYP2D6 inhibitors. Appropriate medical judgment is required. Please contact AstraZeneca with any queries you have on this issue.

Table 6 Exposure, pharmacological action and toxicity that may be increased by inhibition of CYP2D6 by AZD5363

Amitryptyline<br>Desipramine<br>Trimipramine<br>Doxepin	Minimum of 2 weeks washout prior to AZD5363 administration and for 2 weeks following discontinuation of AZD5363
Atomoxetine<br>Metoprolol<br>Nefazodone<br>Nebivolol<br>Perphenazine<br>Tropisetron<br>Tolterodine	Minimum of 1 week washout prior to AZD5363 administration and for 2 weeks following discontinuation of AZD5363

Agents that are sensitive to CYP2D6 inhibition that AstraZeneca considers may be allowed with caution

Table 7 Exposure, pharmacological action and toxicity that may be increased by inhibition of CYP2D6 by AZD5363

Venlafaxine<br>Paroxetine<br>Fluoxetine	Drugs are permitted but caution should be exercised and patients monitored closely for possible drug interactions. Please refer to full prescribing information for all drugs prior to co-administration with AZD5363.

Agents that are sensitive to combined CYP3A4 and CYP2D6 inhibition that AstraZeneca strongly recommend are not combined with AZD5363

There are currently no data confirming that there is a pharmacokinetic (PK) interaction between AZD5363 and the following agents; a potential interaction is considered on the basis of the preclinical data only. This list is not intended to be exhaustive, and a similar restriction will apply to other agents with narrow therapeutic windows that are known to depend on combined CYP3A4 and CYP2D6 metabolism. Appropriate medical judgment is required. Please contact AstraZeneca with any queries you have on this issue.

Table 8 Exposure, pharmacological action and toxicity that may be increased by inhibition of CYP3A4 and CYP2D6 by AZD5363

Haloperidol	Minimum of 2 weeks washout prior to AZD5363 administration and for 2 weeks following discontinuation of AZD5363
Tramadol	Minimum of 1 week washout prior to AZD5363 administration and for 2 weeks following discontinuation of AZD5363

Guidance for drugs that are that are significantly metabolised by CYP2B6, CYP2C9 or CYP2C19 and have a narrow therapeutic margin that AstraZeneca considers may be allowed with caution

Weak signals for competitive inhibition of CYP2B6, CYP2C9 and CYP2C19 cytochrome P450 activities have been demonstrated by in vitro laboratory investigations. There are currently no data confirming that there is a pharmacokinetic (PK) interaction between AZD5363 and substrates of these isoforms; a potential interaction is considered on the basis of the preclinical data only. The following list is intended to identify known sensitive substrates of CYP2B6, CYP 2C9 and CYP 2C19 that have a narrow therapeutic margin. The list is not intended to be exhaustive, and a similar restriction should be applied to any other sensitive substrate with narrow therapeutic margin. Appropriate medical judgment is required. Please contact AstraZeneca with any queries you have on this issue.

Table 9 Exposure, pharmacological action and toxicity that may be increased by inhibition of CYP2B6, CYP2C9 and CYP2C19 by AZD5363

<u>CYP2B6</u><br>Bupropion<br><br><u>CYP2C9</u><br>Warfarin<br><br><u>CYP2C19</u><br>Clobazam	Drugs are permitted but caution should be exercised and patients monitored closely for possible drug interactions. Please refer to full prescribing information for all drugs prior to co-administration with AZD5363.

Guidance for statins that are metabolised by CYP3A4 that AstraZeneca considers may be allowed with caution

Time-dependent inhibition of cytochrome P450 (CYP) 3A4/5 was observed during the non clinical in vitro evaluation of the metabolism of AZD5363.

The CYP3A4 isozyme is responsible for the metabolism of atorvastatin (ATV), cerivastatin (CRV), lovastatin (LOV), and simvastatin (SMV), including combinations with ezetimibe (SMV/ezetimibe [SMV/EZE]), and their exposure, pharmacological action and toxicity may increase by inhibition of CYP 3A4 and the potential for CYP-mediated Drug-Drug interactions (DDIs) is high.

However, there is minimal metabolism of fluvastatin (FLV), pravastatin (PRV), or rosuvastatin (RSV) by CYP3A4 thus plasma levels are minimally influenced by CYP3A4 inhibitors, conveying a relatively low potential for clinically significant DDIs via this mechanism.

Emerging in vitro data has revealed that AZD5363 has a potential to inhibit the OATP1B1 transporter. This transporter is implicated in the distribution and clearance of many of the statins. Of the statins that are minimally affected by CYP3A4 inhibition, RSV and PRV (but not FLV) can be affected by OATP1B1 inhibition. Based on an assessment of the potential for AZD5363 to inhibit OATP1B1 based on the in vitro signal the AUC of these drugs may be increased by 1.3-fold for PRV and 1.5-fold for RSV (static assessment based on maximal free liver inlet concentration of AZD5363). As a conservative response to this emerging data it is recommended that doses of RSV be capped to 10 mg once daily and PRV be capped to 40 mg once daily when combined with AZD5363, and for a 2 week period before and after AZD5363 treatment.

In summary, RSV (up to 10 mg once daily), PRV (up to 40 mg once daily) and FLV are appropriate agents to be used in patients included in AZD5363 studies who require statin therapy.

References:

Michalets EL. Update: clinically significant cytochrome P-450 drug interactions. Pharmacother. 1998;18(1):84-112. http://medicine.iupui.edu/clinpharm/ddis/table.asp

Washington School of Pharmacy Drug Interaction Database resources (online)

APPENDIX VI: POSSIBLE PRENATAL EXPOSURE TO TERATOGEN REPORT

Attach to AdEERS 7-Day Report

Possible Prenatal Exposure to Teratogen Report
AdEERS Ticket Number:________________

Study #:
SAE FAX NO: (301) 230-0159
Alternate FAX NO: (301) 897-7404

Initial Report Date: __ - __ - __ (DD MMM YY)	Follow-up Report Date: __ - __ - __ (DD MMM YY)
Principal Investigator:	Reporter:
Reporter Telephone #:	Reporter FAX #:

☐☐☐☐☐ Investigator Number ☐☐☐☐☐ Subject Number

Complete all of the investigator and subject number boxes provided. Use leading zeros, when necessary, to complete all expected boxes.

Example: Investigator #407 would be filled in as: 0 0 4 0 7

☐☐☐ Subject Initials

Record the first letter of the subject's first, middle and last name, in that sequence. If the subject has no middle name, enter a dash.

Example: A - C

Subject's Sex: ☐ Female ☐ Male

Subject's Weight: ______ kg

Subject's Date of Birth: __ - __ - ____ (DD MMM YYYY)

Subject's Ethnicity (check one only): ☐ Hispanic or Latino ☐ Not Hispanic or Latino ☐ Not Available

Subject's Race (check all that apply): ☐ American Indian or Alaska Native ☐ Asian ☐ Black or African American ☐ Native Hawaiian or Other Pacific Islander ☐ White ☐ Not Available

Study Drug: **GDC-0449**

Study Drug Start Date: __ - __ - __ (DD MMM YY)

Study Drug Stop Date: __ - __ - __ (DD MMM YY) **OR** ☐ Study Drug Continuing

Dose:	Route: **ORAL**	Frequency: **QD**	Kit #:

First Day of Last Menstrual Period: __ - __ - __ (DD MMM YY) Estimated Date of Delivery: __ - __ - __ (DD MMM YY)

Method of Contraception (check all that apply):
☐ Oral Contraceptive Pills ☐ Condoms ☐ Periodic Abstinence ☐ Progestin Injection or Implants ☐ Spermacide
☐ Diaphragm ☐ Intrauterine Device (IUD) ☐ Tubal Ligation ☐ Other, specify:________________

Reproductive History: ☐ Gravida ______ ☐ Para ______

Tests performed during pregnancy: ☐ None ☐ Unknown
☐ CVS Results: ☐ Normal ☐ Abnormal
☐ Amniocentesis Results: ☐ Normal ☐ Abnormal
☐ Ultrasound Results: ☐ Normal ☐ Abnormal

Pregnancy Outcome
Was pregnancy interrupted? ☐ Yes ☐ No
If yes, specify: ☐ Elective Termination ☐ Spontaneous Abortion ☐ Ectopic
Date of Termination: __ - __ - __ (DD MMM YY)
If pregnancy was **not** terminated, specify pregnancy outcome (and provide infant outcome information)
☐ Vaginal Birth: ☐ Premature ☐ Term **OR** ☐ C-Section: ☐ Scheduled ☐ Emergency Date of Delivery: __ - __ - __ (DD MMM YY)
Infant outcome information: ☐ Normal ☐ Abnormal

Additional Case Details (if needed):

Note: Report possible teratogen exposure to AdEERS within 24 hours. See Protocol Section 11.3.1 for instructions. Attach this form to the complete 5-day AdEERS report.

APPENDIX VII: CENTRAL LABORATORY GENOTYPE TESTING PROCEDURES

Integral Molecular Testing
Background

The SNAPSHOT NGS assay is a fully validated clinical test designed and developed at the MGH Center for Integrated Diagnostics and is performed in a CLIA-certified laboratory. This assay combines anchored-multiplex PCR with next-generation sequencing for detection of single nucleotide variants (SNVs), insertions/deletions (indels), and copy number variants (CNVs) across a number of known cancer genes using genomic DNA derived from patient specimens. This assay has been clinically validated, and standard operating procedures have been created.. While a total of 106 genes are targeted by this assay, only genetic alterations in SMO, PTCH1, NF2, AKT1, PIK3CA, PTEN, CDKN2A, CDK4, CDK6, CCND1, CCND2, CCND3, and CCNE1 will be evaluated for determining patient eligibility and reported to the site and the Alliance.

Methodology

Nucleic Acid Extraction: As the first step in prospective testing for eligible biomarker evaluation, total nucleic acids consisting of both DNA and RNA will be co-extracted from diagnostic formalin-fixed, paraffin-embedded tumor tissue using a modified Agencourt FormaPure method automated on a Beckman Coulter NXP workstation (Beckman Coulter, Pasadena, Calif). This method has been clinically validated and comprises our nucleic acid extraction platform used in our clinical tumor mutational profiling program.

Gene Variant Testing Using The SNAPSHOT Assay: Nucleic acid samples will subsequently undergo prospective tumor genotyping using the clinical SNAPSHOT genotyping assay for cancer gene variant detection. Double-stranded DNA contained within the total nucleic acid sample will be used to produce adapter-ligated DNA libraries for sequencing in a high-throughput manner. This will include fragmentation of the DNA, repair of the DNA strands to produce blunt ended strands, phosphorylation of the 5' ends of the DNA, attachment of dATPs at the 3' ends, ligation of uniquely indexed adapter sequences to both ends of the DNA followed by two rounds of nested PCR for enrichment amplification of the gene exons of interest. SPRI bead clean-up steps are incorporated within these procedures. Adapter-ligated DNA libraries will be quantified by qPCR using the Kapa Illumina kit and then be sequenced on an Illumina MiSeq or NextSeq genomic sequencer (both the positive and negative strand).

Bioinformatic Variant Analysis: Sequencing data generated through SNAPSHOT testing will be processed through the clinically-validated MGH bioinformatics pipeline. First, the sequencing (FASTQ) data will be demultiplexed, aligned to the human reference genome, and the resultant binary files (BAM) generated. Secondly, somatic mutations in the patient samples will be deciphered and annotated using the Mutect and Oncotator bioinformatics tools developed at the BROAD institute. Quality assessment includes the incorporation of molecular barcoding and a minimum sequencing depth threshold.

Target Mutations

The following genetic alterations will be considered eligible when identified in the primary meningioma tissue:

	SNAPSHOT NGS ASSAY*					
	Eligible Variants				CNV	
Arm	Oncogene		Tumor Suppressor		Amplification	Deletion
**Abemaciclib**	n/a		***CDKN2A***	*Nonsense, indel, frameshift, splice site mutations*	***CDK4***<br>***CDK6***<br>***CCND1***<br>***CCND2***<br>***CCND3***<br>***CCNE1***	***CDKN2A***
**GSK2256098**	n/a		***NF2***	Nonsense mutations<br>Indel mutations<br>Frameshift mutations<br>Splice site mutations	n/a	***n/q***
**Vismodegib**	***SMO***	p.S278I (c.833G>T)<br>p.L412F (c.1234C>T)<br>p.S533N (c.1598G>A)<br>p.T534P (c.1600A>C)<br>p.W535L(c.1604G>T)<br>p.P641A (c.1921C>G)	***PTCH1***	Nonsense mutations<br>Indel mutations<br>Frameshift mutations<br>Splice site mutations	n/a	n/a
**AZD5363**	***AKT1***<br>***PIK3CA***	p.E17K (c.49G>A)<br>p.R88Q (c.263G>A)<br>p.E542K (c.1624G>A)<br>p.E542Q (c.1624G>C)<br>p.E545K (c.1633G>A)<br>p.E545Q (c.1633G>C)<br>p.E545A (c.1634A>C)<br>p.E545G (c.1634A>G)<br>p.E545V (c.1634A>T)<br>p.Q546K (c.1636C>A)<br>p.Q546E (c.1636C>G)<br>p.Q546P (c.1637A>C)<br>p.Q546R (c.1637A>G)<br>p.Q546L (c.1637A>T)<br>p.H1047Y (c.3139C>T)<br>p.H1047R (c.3140A>G)<br>p.H1047L (c.3140A>T)<br>p.G1049R (c.3145G>C)<br>p.G1049S (c.3145G>A)	***PTEN***	Nonsense mutations<br>Indel mutations<br>Frameshift mutations<br>Splice site mutations<br>p.C124R (c.370T>C)<br>p.C124S (c.370T>A)<br>p.C124S (c.371G>C)<br>p.G129E (c.386G>A)<br>p.G129R (c.385G>A)<br>p.G129R (c.385G>C)<br>p.G129V (c.386G>T)<br>p.R130G (c.388C>G)<br>p.R130L (c.389G>T)<br>p.R130P (c.389G>C)<br>p.R130Q (c.389G>A)<br>p.C136R (c.406T>C)<br>p.C136Y (c.407G>A)<br>p.R173C (c.517C>T)	n/a	***n/q***

* Other genetic alterations in these target genes when identified during patient sample testing will be compared to the COSMIC database of known somatic alterations and reported if found. If these additional genetic alternations are determined to be potentially actionable, they will be considered for eligibility.

Assay Protocol

1. The SNAPSHOT assay will be performed in a CLIA-certified laboratory at the MGH Translational Research/Biomarker Laboratory (MGH TRL). While these assays detect genetic alterations across a number of cancer genes, only alterations in SMO, PTCH1, NF2, AKT1, PIK3CA, PTEN, CDKN2A, CDK4, CDK6, CCND1, CCND2, CCND3, and CCNE1 will be reported to the requesting site and to the Alliance.

2. Quality assurance measures will require that each trial sample is submitted with two identifiers and that an email is sent to the MGH TRL centralized mailbox (MGHTRLClinicalTrials@partners.org) indicating intent to submit the sample and the relevant identifiers. The MGH TRL program

coordinator will verify that the sample received matches the information provided by the site, as recorded on the upper portion of the "ALLIANCE A071401 Central Pathology And Biomarker Results Form" that must be included with the specimen. Sample acceptability will be determined based on shipping and received condition and sample identifiers will be confirmed before initiating the testing process. Testing will be delayed in the event of improper sample submission, sample labeling, or completion of the "ALLIANCE A071401 Central Pathology and Biomarker Results Form."

3. Samples received into the MGH TRL for testing will be annotated into a laboratory database and a chain of custody sheet will be initiated to track and record each step of the testing process, the staff member conducting the process, the quantity of sample that was utilized during testing, the testing result(s), and reporting.

4. The H&E slide that corresponds to the diagnostic tissue sections submitted to the MGH TRL will be reviewed by a pathologist and the tumor area to be extracted will be marked.

5. All sections of tumor tissue received for a trial sample will be macroscopically dissected according to the pathologist's marked H&E slide. Nucleic acid will be extracted in accordance with a clinically-validated protocol using Agencourt FormaPure technology on a Biomek NXp robotic workstation. While no sections will be saved, remaining extracted nucleic acid that remains after testing will be stored at -80°C in the laboratory's nucleic acid bank. Double-stranded DNA concentration will be determined using Qubit fluorometric quantitation.

6. Samples undergoing SNAPSHOT testing will be sequenced on an Illumina MiSeq or NextSeq genome sequencer. The assays have been clinically validated and standard operating procedures have been documented.

7. Sequencing data generated will be processed through a validated bioinformatics pipeline developed at MGH where the sequencing (FASTQ) data is demultiplexed, aligned to the human reference genome and the resultant binary files (BAM) generated. Somatic mutations in the patient samples will additionally be deciphered and annotated using the MGH CIDer bioinformatics tool. Quality assessment includes the incorporation of molecular barcoding and a minimum sequencing depth threshold.

8. If NO eligible mutation was identified during testing, the site and the Alliance will be notified of biomarker ineligibility. Central Pathology Review will not be required or performed.

9. If an eligible biomarker has been found, all diagnostic H&E slides received for that case will be forwarded to Dr. Sandro Santagata for Central Pathology Review to confirm diagnosis and tumor grade. The completed and signed central pathology review form will be electronically forwarded to Dr. John Iafrate for inclusion in final reporting. For the purposes of record keeping, electronic signatures will be considered identical to original wet signatures.

10. Expected turnaround time for testing and reporting is 21 days from the date of receiving acceptable FFPE tumor recuts, the full H&E slide series, a de-identified pathology report, and a properly completed "ALLIANCE A071401 Central Pathology And Biomarker Results Form" into the MGH TRL. Inadequate or poor quality sample that requires sample resubmission or that requires repeat testing due to inadequate read depth will delay reporting.

APPENDIX VIII: PATIENT DRUG INFORMATION HANDOUTS AND WALLET CARDS

The following information handouts and wallet cards are included within this appendix:

Appendix VIII-A For patients receiving GSK2256098

Appendix VIII-B For patients receiving AZD5363

Appendix VIII-C: For patients receiving abemaciclib

Appendix VIII-A: For patients receiving GSK2256098

Information for Patients, Their Caregivers and Non-Study Healthcare Team on Possible Interactions with Other Drugs and Herbal Supplements

The patient ______________________________ is enrolled on a clinical trial using the experimental study drug, GSK 2256098. This clinical trial is sponsored by the National Cancer Institute. This form is addressed to the patient, but includes important information for others who care for this patient.

These are the things that you as a healthcare provider need to know:

GSK 2256098 interacts with a certain specific enzyme in your liver.

- The enzyme(s) in question is CYP3A4 and GSK 2256098 is broken down by this enzyme and may be affected by other drugs that inhibit or induce this enzyme.

To the patient: Take this paper with you to your medical appointments and keep the attached information card in your wallet.

GSK 2256098 may interact with other drugs which can cause side effects. For this reason, it is very important to tell your study doctors of any medicines you are taking before you enroll onto this clinical trial. It is also very important to tell your doctors if you stop taking any regular medicines, or if you start taking a new medicine while you take part in this study. When you talk about your current medications with your doctors, include medicine you buy without a prescription (over-the-counter remedy), or any herbal supplements such as St. John's Wort. It is helpful to bring your medication bottles or an updated medication list with you.

Many health care providers can write prescriptions. You must tell all of your health care providers (doctors, physician assistants, nurse practitioners, pharmacists) you are taking part in a clinical trial.

These are the things that you and they need to know:

GSK 2256098 must be used very carefully with other medicines that use certain liver enzymes. Before you enroll onto the clinical trial, your study doctor will work with your regular health care providers to review any medicines and herbal supplements that are considered strong inducers/inhibitors of CYP3A4.

- Please be very careful! Over-the-counter drugs (including herbal supplements) may contain ingredients that could interact with your study drug. Speak to your doctors or pharmacist to determine if there could be any side effects.
- Your regular health care provider should check a frequently updated medical reference or call your study doctor before prescribing any new medicine or discontinuing any medicine. Your study doctor's name is ____ and he or she can be contacted at _____________________.

STUDY DRUG INFORMATION WALLET CARD

You are enrolled on a clinical trial using the experimental study drug GSK2256098. This clinical trial is sponsored by the NCI. GSK2256098 may interact with drugs that are processed by your liver*.]*. Because of this, it is very important to:

- Tell your doctors if you stop taking any medicines or if you start taking any new medicines.
- Tell all of your health care providers (doctors, physician assistants, nurse practitioners, or pharmacists) that you are taking part in a clinical trial.
- Check with your doctor or pharmacist whenever you need to use an over-the-counter medicine or herbal supplement.

GSK2256098 interacts with a specific liver enzyme called CYP3A4**,** and must be used very carefully with other medicines that interact with this enzyme.

- Before you enroll onto the clinical trial, your study doctor will work with your regular health care providers to review any medicines and herbal supplements that are considered strong inducers/inhibitors or substrates of CYP3A4
- Before prescribing new medicines, your regular health care providers should go to a frequently-updated medical reference for a list of drugs to avoid, or contact your study doctor.
- Your study doctor's name is ______________________________

  and can be contacted at _________________________________.

Appendix VIII-B: For patients receiving AZD5363

Information for Patients, Their Caregivers and Non-Study Healthcare Team on Possible Interactions with Other Drugs and Herbal Supplements

The patient ____________________________ is enrolled on a clinical trial using the experimental study drug, AZD5363. This clinical trial is sponsored by the National Cancer Institute. This form is addressed to the patient, but includes important information for others who care for this patient.

These are the things that you as a healthcare provider need to know:

AZD5363 interacts with certain specific enzymes in your liver.

- The enzymes in question are CYP3A4 and CYP2D6. AZD5363 is broken down by these enzymes and may be affected by other drugs that inhibit or induce these enzymes.

To the patient: Take this paper with you to your medical appointments and keep the attached information card in your wallet.

AZD5363 may interact with other drugs which can cause side effects. For this reason, it is very important to tell your study doctors of any medicines you are taking before you enroll onto this clinical trial. It is also very important to tell your doctors if you stop taking any regular medicines, or if you start taking a new medicine while you take part in this study. When you talk about your current medications with your doctors, include medicine you buy without a prescription (over-the-counter remedy), or any herbal supplements such as St. John's Wort. It is helpful to bring your medication bottles or an updated medication list with you.

Many health care providers can write prescriptions. You must tell all of your health care providers (doctors, physician assistants, nurse practitioners, pharmacists) you are taking part in a clinical trial.

These are the things that you and they need to know:

AZD5363 must be used very carefully with other medicines that use certain liver enzymes. Before you enroll onto the clinical trial, your study doctor will work with your regular health care providers to review any medicines and herbal supplements that are considered strong inducers/inhibitors of CYP3A4 or CYP2D6.

- Please be very careful! Over-the-counter drugs (including herbal supplements) may contain ingredients that could interact with your study drug. Speak to your doctors or pharmacist to determine if there could be any side effects.
- Your regular health care provider should check a frequently updated medical reference or call your study doctor before prescribing any new medicine or discontinuing any medicine. Your study doctor's name is _____________________________________ and he or she can be contacted at ____________________.

STUDY DRUG INFORMATION WALLET CARD

You are enrolled on a clinical trial using the experimental study drug AZD5363. This clinical trial is sponsored by the NCI. AZD5363 may interact with drugs that are processed by your liver. Because of this, it is very important to:

- Tell your doctors if you stop taking any medicines or if you start taking any new medicines.
- Tell all of your health care providers (doctors, physician assistants, nurse practitioners, or pharmacists) that you are taking part in a clinical trial.
- Check with your doctor or pharmacist whenever you need to use an over-the-counter medicine or herbal supplement.

AZD5363 interacts with a specific liver enzyme called CYP3A4**,** and must be used very carefully with other medicines that interact with this enzyme.

- Before you enroll onto the clinical trial, your study doctor will work with your regular health care providers to review any medicines and herbal supplements that are considered strong inducers/inhibitors or substrates of CYP3A4
- Before prescribing new medicines, your regular health care providers should go to a frequently-updated medical reference for a list of drugs to avoid, or contact your study doctor.
- Your study doctor's name is ______________________________

  and can be contacted at _________________________________.

Appendix VIII-C: For patients receiving Abemaciclib

Information for Patients, Their Caregivers and Non-Study Healthcare Team on Possible Interactions with Other Drugs and Herbal Supplements

[Note to authors: This appendix consists of an "information sheet" to be handed to the patient at the time of enrollment. Use or modify the text as appropriate for the study agent, so that the patient is aware of the risks and can communicate with their regular prescriber(s) and pharmacist. A convenient wallet-sized information card is also included for the patient to clip out and retain at all times. If you choose to use them, please note that the information sheet and wallet card will require IRB approval before distribution to patients.]

The patient ____________________________ is enrolled on a clinical trial using the experimental study drug, ***Abemaciclib****.* This clinical trial is sponsored by the National Cancer Institute. This form is addressed to the patient, but includes important information for others who care for this patient.

These are the things that you as a healthcare provider need to know:

Abemaciclib interacts with *a certain specific enzyme in your liver*.*

- *The enzyme in question is ***Cytochrome P450 CYP3A.*** Use of medications that block CYP3A with *abemaciclib* may increase the amount of *abemaciclib* in the body. Use of medications that speed up the activity of *abemaciclib* may decrease the amount of *abemaciclib* in the body.

To the patient: Take this paper with you to your medical appointments and keep the attached information card in your wallet.

Abemaciclib may interact with other drugs which can cause side effects. For this reason, it is very important to tell your study doctors of any medicines you are taking before you enroll onto this clinical trial. It is also very important to tell your doctors if you stop taking any regular medicines, or if you start taking a new medicine while you take part in this study. When you talk about your current medications with your doctors, include medicine you buy without a prescription (over-the-counter remedy), or any herbal supplements such as St. John's Wort. It is helpful to bring your medication bottles or an updated medication list with you.

Many health care providers can write prescriptions. You must tell all of your health care providers (doctors, physician assistants, nurse practitioners, pharmacists) you are taking part in a clinical trial.

These are the things that you and they need to know:

Abemaciclib must be used very carefully with other medicines that use certain ***liver enzymes***. Before you enroll onto the clinical trial, your study doctor will work with your regular health care providers to review any medicines and herbal supplements that are considered ***strong inducers/inhibitors*** of ***CYP3A.***

- Please be very careful! Over-the-counter drugs (including herbal supplements) may contain ingredients that could interact with your study drug. Speak to your doctors or pharmacist to determine if there could be any side effects.
- Your regular health care provider should check a frequently updated medical reference or call your study doctor before prescribing any new medicine or discontinuing any medicine. Your study doctor's name is

____ and he or she can be contacted at

____.

STUDY DRUG INFORMATION WALLET CARD

You are enrolled on a clinical trial using the experimental study drug**, Abemaciclib.** This clinical trial is sponsored by the NCI. **Abemaciclib** may interact with drugs that are ***[processed by your liver.*** Because of this, it is very important to:

- Tell your doctors if you stop taking any medicines or if you start taking any new medicines.
- Tell all of your health care providers (doctors, physician assistants, nurse practitioners, or pharmacists) that you are taking part in a clinical trial.
- Check with your doctor or pharmacist whenever you need to use an over-the-counter medicine or herbal supplement.

Abemaciclib interacts with a ***specific liver enzyme called CYP3A*** and must be used very carefully with other medicines that interact with ***this enzyme.***

- Before you enroll onto the clinical trial, your study doctor will work with your regular health care providers to review any medicines and herbal supplements that are considered ***strong inducers/inhibitors of CYP3A.***
- Before prescribing new medicines, your regular health care providers should go to a frequently-updated medical reference for a list of drugs to avoid, or contact your study doctor.
- Your study doctor's name is ______________________________

  and can be contacted at __________________________________.

APPENDIX IX: ALGORITHMS FOR MANAGEMENT OF TOXICITIES WITH AZD5363

Figure 1 Toxicity management algorithm

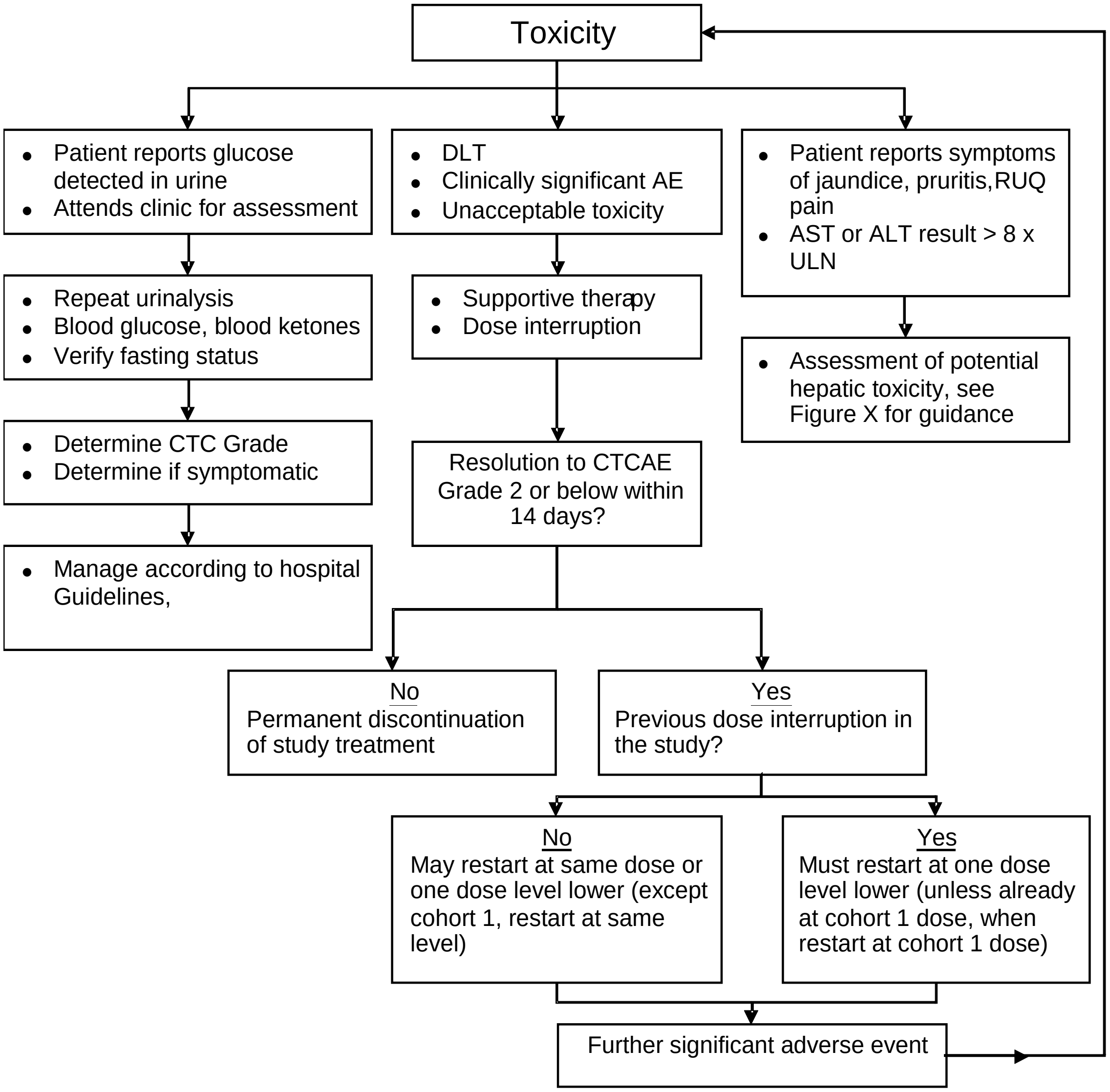

Figure 2 **Glucose intervention plan**

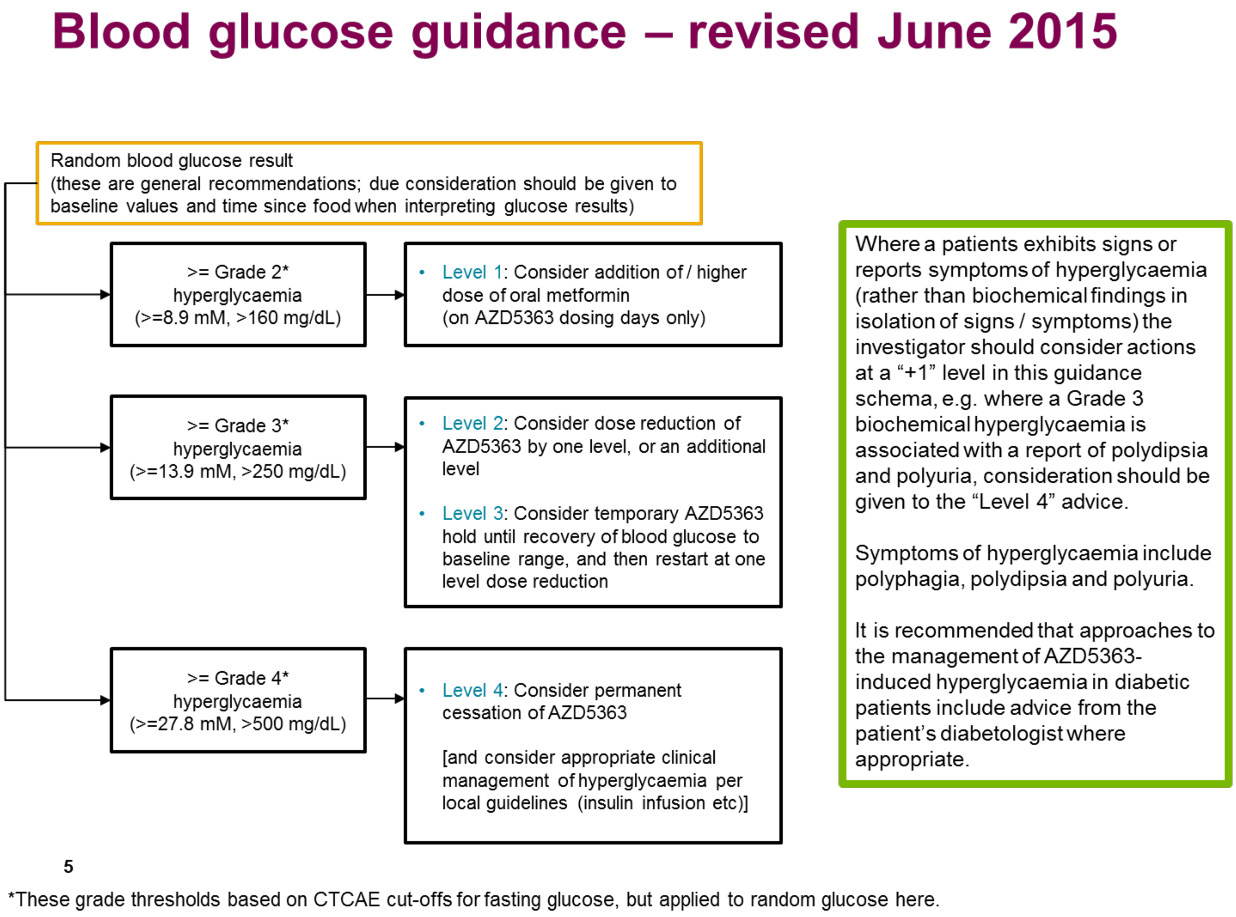

Figure 3 Hepatotoxicity management algorithm

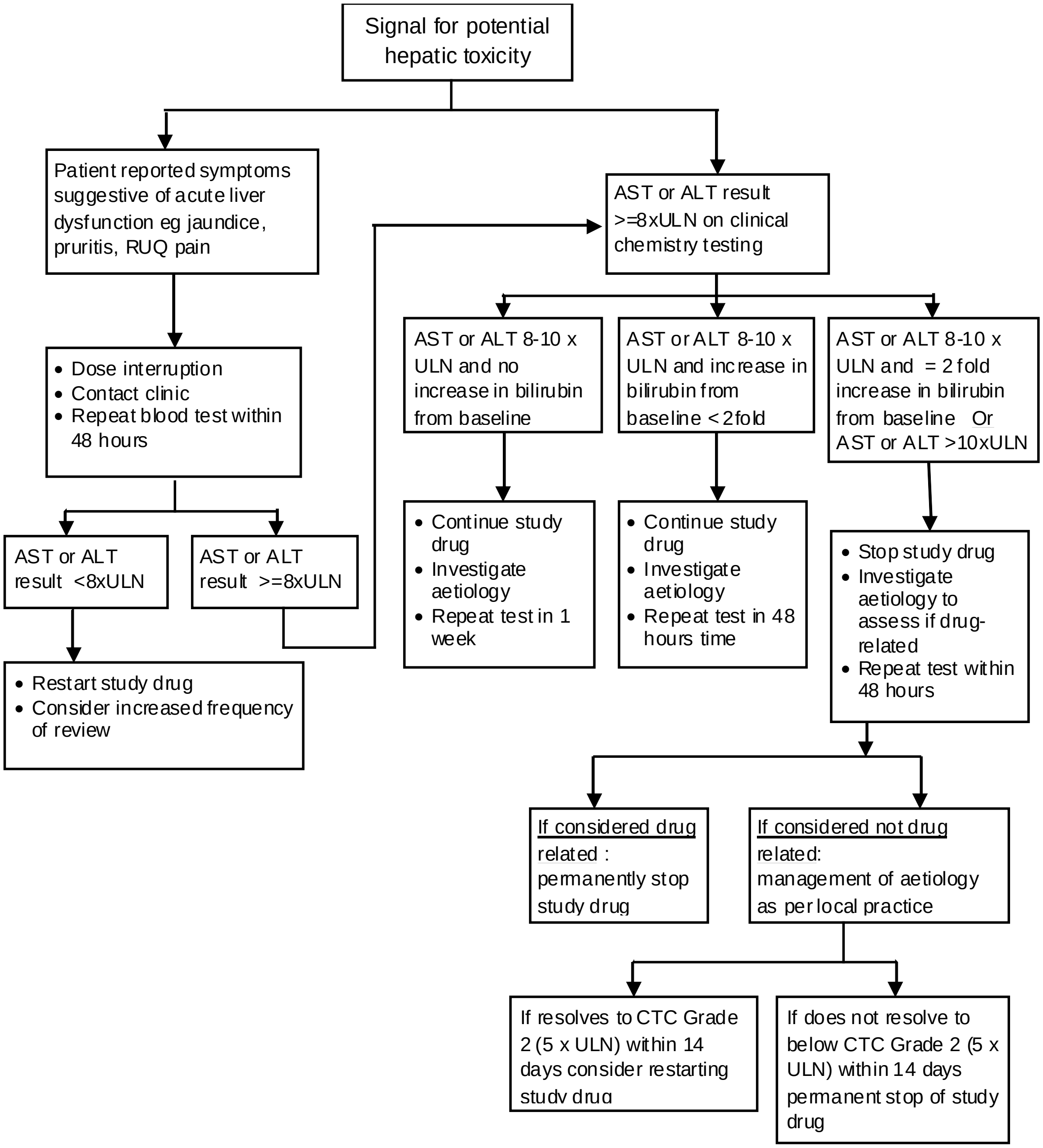

Figure 4 Maculo-Papular Rash management guidance

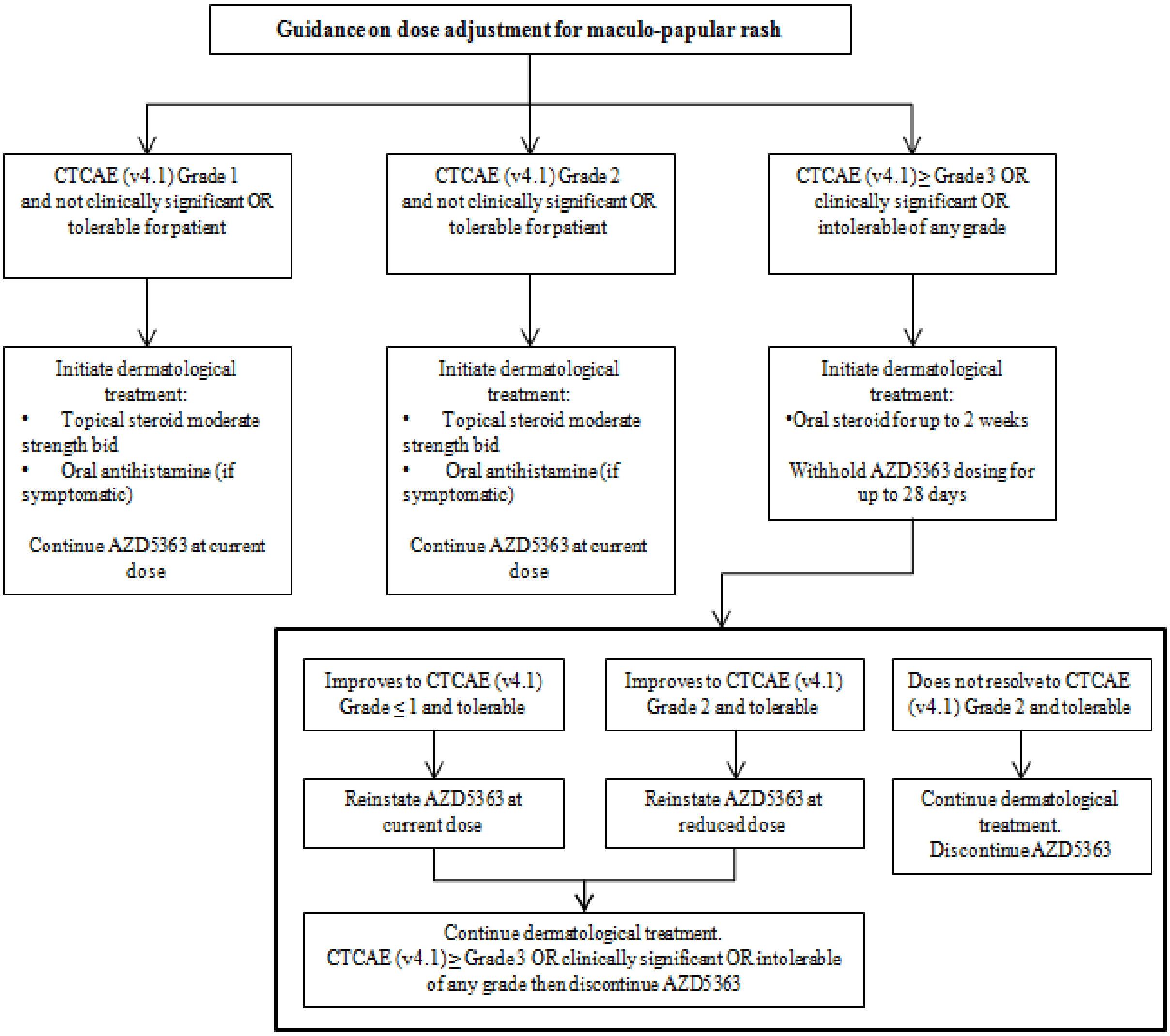

List of Amendments to A071401

Protocol Update #01
XX/XX/2015

ALLIANCE FOR CLINICAL TRIALS IN ONCOLOGY

PROTOCOL UPDATE TO ALLIANCE A071401

PHASE II TRIAL OF SMO/AKT/NF2 INHIBITORS IN PROGRESSIVE MENINGIOMAS WITH SMO/AKT/NF2 MUTATIONS

Industry-supplied agent(s): Vismodegib (IND #126926) and GSK2256098 (IND #126926); IND holder: Alliance

X	**Update:**		**Status Change:**
X	Eligibility changes		Activation
X	Therapy / Dose Modifications / Study Calendar changes		Closure
X	Informed Consent changes		Suspension / temporary closure
	Scientific / Statistical Considerations changes		Reactivation
	Data Submission / Forms changes		
X	Editorial / Administrative changes		
	Other :		

IRB approval (or disapproval) is required within 90 days. Full Board review is recommended. Please follow your local IRB guidelines.

CHANGES TO THE PROTOCOL

Title Page

- The IND numbers for Vismodegib and GSK2256098 have been added beneath the study title. It now reads, **"***Industry-supplied agent(s): Vismodegib (IND #126926), GSK2256098 (IND #126926); IND holder: Alliance"*
- "*Afuresertib (IND # XXXXX)*" has been removed from beneath the study title as per section 1.3, this agent is currently unavailable.
- The "ClinicalTrials.gov Identifier (NCT02523014)" has been added beneath the title.
- The names of two participating groups have been corrected at the bottom of the page. Therefore, "ECOG-ACRIN Medical Research Foundation, Inc." and "NRG Oncology Foundation Inc." have been changed to "ECOG-ACRIN Cancer Research Group" and "NRG Oncology" respectively.

Schema

- "QTcF*** ≤ 500 msec*" has been added to the list of "Required Initial Laboratory Values along with footnote "***".

- A reference to Section 3.3.7 has been added to the following criterion: "No uncontrolled gastric ulcer disease."

Section 3.1 On-Study Guidelines

- The second paragraph has been changed from **"**Although they will not be considered formal eligibility (exclusion) criteria, physicians should recognize that the following may seriously increase the risk to the patient entering this protocol:" to "The following may seriously increase the risk to the patient entering this protocol:"
- A new section header title, which reads "Reproductive considerations, vismodegib" has been added. Also, references to "GDC 0449" have been replaced with "vismodegib" throughout this section. Furthermore, four paragraphs regarding additional reproductive considerations have been added to the send of this section.
- The section header title "Reproductive considerations, GSK2256098" has been added.
- The section header title "Drug interactions" has been added for GSK2256098 above the final two paragraphs within this section.

Section 3.3 Registration Eligibility Criteria

- The word "registration" has been added to the section header title.

Section 3.3.3 Prior Treatment

- The word "medical" has been added to the first bullet. It now reads, "Prior medical therapy is allowed but not required."
- In the fifth bullet, "study drug administration" has been changed to "registration." It now reads, "For patients treated with external beam radiation, interstitial brachytherapy or radiosurgery, an interval > 24 weeks must have elapsed from completion of XRT to registration (See 3.3.1)."

Section 3.3.7 Patient History

- A parenthetical reference that reads, "Grade 3 gastric ulcer disease within 28 days of registration," has been added to the sixth bullet.

Section 3.3.9 Required Initial Laboratory Values

- "QTcF*** ≤ 500 msec*" has been added to the list of "Required Initial Laboratory Values," along with footnote "***" which reads, "QT calculated using Fridericia formula: QTc = QT/(RR^0.33), where RR = 60/HR."

Section 4.3 CTSU Site Registration Procedures

- In the first sentence of the second paragraph, **"**Sites participating on the NCI CIRB initiative and accepting CIRB approval for the study are not required to submit separate IRB approval documentation to the CTSU Regulatory Office for initial, continuing or amendment review" has been changed to "Sites participating on the NCI CIRB initiative that are approved by the CIRB for the study are not required to submit separate IRB approval documentation to the CTSU Regulatory Office for initial, continuing or amendment review."
- The second and third sentences of the second paragraph have been changed from "This information will be provided to the CTSU Regulatory Office from the CIRB at the time the site's Signatory Institution accepts the CIRB approval. The Signatory site may be contacted by the CTSU Regulatory Office or asked to complete information verifying the participating institutions on the study" to "This information will be provided to the CTSU Regulatory Office from the CIRB at the time of the CIRB's approval. The Signatory Institution must inform the CTSU which CIRB-approved institutions aligned with the Signatory Institution are participating in a given study so that the study approval can be applied to those institutions.

Section 5.0 Study Calendar

- "EKG(!)" has been added under the "Tests & Observations" column and "X($)" has been added under the following columns: "Prior to Registration*" and "Day 1 of each cycle (cycle is 28 days)*"
- Within the table, in the "Serum or Urine HCG" row under "Laboratory Studies," "X(1)" has been added under the "Day 1 of each cycle (cycle is 28 days)*" column.
- The following has been added beneath the section entitled "Correlative studies: For patients who consent to participate": "MR Imaging: DCE MRI imaging should be performed at sites with such capability. DCE MRI will be acquired as part of routine clinical imaging and would not be an extra set of images. See "MRI/CT Brain" under "Staging." See Section 14.2 and Appendix II."
- Footnote "$" has been added, which reads: "EKG must be performed at 2 time points: within 28 days of registration, and 1 hour after taking the first dose of GSK2256098."
- Footnote 1 has been changed from "For women of childbearing potential (see Sections 3.1 and 3.3.4). Must be done ≤ 7 days prior to registration and ≤ 14 days prior to initiation of vismodegib for patients with *SMO* mutation" to "For women of childbearing potential (see Sections 3.1 and 3.3.4). Must be done ≤ 7 days prior to registration and ≤ 7 days prior to initiation of vismodegib for patients with *SMO* mutation and ≤ 7 days prior to initiation of GSK 2256098 for patients with NF2 mutation. While on vismodegib, WOCP must continue to receive pregnancy tests on day 1 of every cycle (+/- 3 days).

Section 6.2 Specimen collection and submission

- Within the table, the number of tubes required for the submission of whole blood has been changed from "3" to "1."
- Within the table, "Whole blood for ctDNA plasma[4] (EDTA/lavender top)" has been added under the section title header "For patients registered to A0711401-ST1, submit the following: Optional."
- A reference to Footnote 1 has been added to "ALL diagnostic H&E slides from original diagnosis [1,]***" and to "One paraffin block containing at least 1 cm^2 of viable tumor [1],*,**, ***"
- In footnotes 2 and 3, the reference to Section 14.2 has been changed to Section 14.1.
- In footnote 3, the second and third sentences which previously read "Collect 1 x 10mL during pre-registration which will be used for germline DNA and collect 2 x 10 mL every 16 weeks. See Section 6.4" has been changed to "Collect 1 x10 mL during pre-registration which will be used for germline DNA. See Section 6.4 for instructions."
- Footnote 4 has been added below the table, which reads: "Whole blood to be used for circulating tumor DNA (ctDNA) analyses described in Section 14.1.2. Collect 2 x 10 mL during pre-registration and every 16 weeks while patient is on study. There are specific instructions for plasma extraction for ctDNA. See Section 6.4 for details."
- Two typographical errors have been corrected in footnote "***." "Has" has been changed to "have" and "form" has been changed to "from."

Section 6.2.1 Specimen submission using the Alliance Biospecimen Management System

- Lauren Logan has replaced Sarah Charbonneau as the contact person for tissue samples sent to Dana-Farber. Contact information has been updated.
- The header that previously read, "Recurrent tumor tissue and blood submission for patients who agree to participate," has been changed to "Blood submission for patients who agree to participate:"
- The shipment address for the submission of tissue for the correlative studies and the contact information for questions regarding submission have been added.

Section 6.4 Blood sample submission

- In the first sentence, the reference to Section 14.2 has been changed to Section 14.1.2.
- The section header title "Whole Blood for germline DNA" has been added beneath the first sentence.
- In the first sentence of the first bullet, "Collect three 10 mL of venous blood at pre-registration and then two 10 mL every 16 weeks in lavender top (EDTA anticoagulant) vacutainer tube(s)" has been changed to "Collect one 10 mL of venous blood at pre-registration in lavender top (EDTA anticoagulant) vacutainer tube(s)."
- In the second bullet, "All blood sample…" has been changed to "Whole blood samples…"
- Instructions on the collection procedures of whole blood for circulating tumor DNA have been added.

Section 6.5 CT and MR Imaging Data Submission

- In the third paragraph, the first sentence has been changed from "Complete data sets in digital DICOM format, along with Alliance Adjunctive Data Form (if applicable) and Alliance Image Measurement Form (if applicable), must be submitted to the ALLIANCE Imaging Core Laboratory" to "Complete data sets in digital DICOM format and submit to IROC Ohio."
- In the fourth paragraph, "Data should be transferred electronically (**recommend**) to the IROC Ohio as follows: Electronically" has been changed to "Data should be transferred electronically to the IROC Ohio via TRIAD, Web Transfer or FTP Transfer."
- Additional information about TRIAD has been included.

Section 7.1 CYP3A4 Inhibitors

- The following sentence has been added at the end of this section: "A wallet-size card providing information regarding potential drug interactions has been made available in Appendix VII."

Section 7.2 CYP3A4 Inducers

- The following sentence has been added at the end of this section: "A wallet-size card providing information regarding potential drug interactions has been made available in Appendix VII."

Section 8.1.11 Alliance Policy Concerning the Use of Growth Factors

- The following sentence has been added beneath the third paragraph: "Due to concerns regarding the inherent toxicity of EPO and the investigational agents employed in the protocol, use of EPO is strongly discouraged."

Section 8.1.14 Hepatitis

- This section has been added to provide information on the management of hepatitis for patients on study.

Section 8.1.15 QT Prolongation

- This section has been added to provide guidance on the management of patients that experience QT prolongation while on protocol therapy.

Section 8.3.3 Hepatic Toxicity

- The second bullet has been changed from "For grade 3 ALT, AST delay GSK2256098 until grade ≤ 1 then resume with one dose level reduced" to "For grade 3 or 4 ALT, AST discontinue GSK2256098." In addition the former third bullet, which previously read, "For grade 4 ALT, AST discontinue GSK2256098," has been removed as a result.

- The following bullet has been added: “For combined grade 2 AST/ALT and grade 2 bilirubin, discontinue GSK2256098.

Section 8.3.7 Investigations

- This section has been added for clarity.

Section 8.3.8 Other adverse event

- This section has been added for clarity.

Section 9.1 Routine adverse event reporting

- “Muscle spasms (musculoskeletal disorder others) has been removed from under the “CTCAE v4.0 Term” column and “Musculoskeletal and connective tissue disorders” has been removed from under the “CTCAE vX.0 System Organ Class (SOC)” column.
- “Rash” has been changed to “Rash maculopapular”

Section 10.1 Vismodegib (GDC-0449, Erivedge®, NSC# 747691, IND#126926) IND holder: Alliance

- The IND # (126926) has been added to the section header title.

Section 10.2 GSK2256098 (NSC# 783781, IND #126926)

- Both the NSC# (783781) and the IND# (126926) have been added to the section header title.
- The storage temperature for GSK2256098 has been added under the section entitled “Preparation, Storage and Stability.”
- The following instructions have been added to the “Drug Accountability” section: “Upon completion of the trial, all remaining drug at the site must be destroyed as per local institutional policy, and notification of destruction must be sent to pharmaffairs@alliancenctn.org within 90 days of trial completion.”

Section 13.6.2

- This section has been updated to reflect the monitoring method for CDUS reporting.

Section 13.8 Inclusion of Women and Minorities

- The number “4” has been added to the row that is titled “White” and under the “Hispanic or Latino, Female” column in the Domestic Planned Enrollment Report table.

Section 14.1.2 Circulating tumor DNA (ct-DNA)

- The word “tumor” and the parenthetical reference “(ct-DNA)” have been added to the section header title.

Appendix VII Patient Drug Information Handout and Wallet Card

- This section has been revised in its entirety to include information for patients on possible drug interactions with GSK2256098.

CHANGES TO THE MODEL CONSENT

What is the usual approach to my meningioma?

- The following has been added as the second sentence to the second paragraph: “There are times where surgery or radiation may be curative, depending on the type of meningioma and what other therapies have been tried. In some cases, close follow up with scans may be appropriate.”

Why is this study being done?

- In the second paragraph, the third sentence has been changed from "Vismodegib could shrink your cancer but it could also cause side effects" to "Vismodegib could shrink your meningioma, or your meningioma could stay the same size or grow. It may cause side effects."
- The following sentences have been added after the first sentence of the third paragraph: "GSK2256098 could shrink your meningioma, or your meningioma could stay the same size or grow. It may cause side effects."

What extra tests and procedures will I have if I take part in this study?

- "EKG (for patients with NF2 mutation)" has been added to the list of extra tests to be performed before you begin the study and during the study.
- In addition, "Pregnancy testing monthly if you are a woman who could become pregnant (for patients with SMO mutation only)" has been added to the list of extra tests to be performed during the study.

What possible risks can I expect from taking part in this study?

- Underneath the "If you are a women" header, the first sentence of the first paragraph has been revised to include the following language: "… (for GSK2256098) or 7 (for vismodegib)…" It now reads: "You should not become pregnant while on this study, and for at least 6 (for GSK2256098) or 7 (for vismodegib) months after completing protocol treatment, because the drugs in this study can affect a fetus."
- In addition, the first sentence of the second paragraph has been changed from "You should not father a baby while on this study, and for at least 6 months after completing protocol treatment, because the drugs in this study can affect a fetus" to "You should not father a baby while on this study, and for at least 3 (for vismodegib) or 6 (for GSK2256098) months after completing protocol treatment, because the drugs in this study can affect a fetus."
- The following sentence has been added at the end of the section: "Also, you should not donate blood while on this study and for at least 7 months after completing study drug, if you are receiving vismodegib.

A replacement protocol document and model consent have been issued

---

ATTACH TO THE FRONT OF EVERY COPY OF THIS PROTOCOL

ALLIANCE FOR CLINICAL TRIALS IN ONCOLOGY

PROTOCOL UPDATE TO ALLIANCE A071401

PHASE II TRIAL OF SMO/AKT/NF2 INHIBITORS IN PROGRESSIVE MENINGIOMAS WITH SMO/AKT/NF2 MUTATIONS

Industry-supplied agent(s): Vismodegib (IND #126926) and GSK2256098 (IND #126926); IND holder: Alliance

X	**Update:**		**Status Change:**
	Eligibility changes		Activation
	Therapy / Dose Modifications / Study Calendar changes		Closure
X	Informed Consent changes		Suspension / temporary closure
	Scientific / Statistical Considerations changes		Reactivation
X	Data Submission / Forms changes		
X	Editorial / Administrative changes		
X	Other : Updated CAEPR for Vismodegib		

IRB approval (or disapproval) is required within 90 days. Expedited review is allowed. Please follow your local IRB guidelines. Reconsent not required. Sites should follow your local IRB policy.

Some of the changes included in this update to A071401 have been made in response to an RA from Dr. Naoko Takebe (takeben@mail.nih.gov) dated October 14, 2015. An Action Letter will not be issued.

CHANGES TO THE PROTOCOL

Title Page

- Samantha Sublett has replaced Tamara Robles as the protocol coordinator.

CANCER TRIALS SUPPORT UNIT (CTSU) ADDRESS AND CONTACT INFORMATION

- The table has been updated to comply with the revised CTSU template language.

Section 4.3.1 Downloading Site Registration Documents

- This section has been updated to reflect the steps for accessing the site registration documents. The following changes have been made as a result:
    - The former second sentence “Go to https://www.ctsu.org and log in to the members’ area using your CTEP-IAM username and password” is now a bullet and it has been replaced

with the following sentence: "Permission to view and download this protocol and its supporting documents is restricted and is based on person and site roster assignment housed in the CTSU RSS."
    - A third bullet has been added and the last bullet has been modified to read, "Click on LPO Documents, select the Site Registration documents link, and download and complete the forms provided."

Section 4.3.4 Submitting Regulatory Requirements

- In the first paragraph, "Submit completed forms along with a copy of your IRB Approval (for sites not participating via the NCI CIRB), Model Informed Consent (for sites not participating via the NCI CIRB), and any other required documentation (see above) to the CTSU Regulatory Office, where they will be entered and tracked in the CTSU RSS" has been changed to "Submit completed forms along with a copy of your IRB Approval and Model Informed Consent to the CTSU Regulatory Office, where they will be entered and tracked in the CTSU RSS."

Section 6.2 Specimen collection and submission

- Within the table, under " For patients registered to A071401-ST1, submit the following: Optional", "ALL diagnostic H&E slides from recurrent tumor" has been changed to "One diagnostic H&E slide from recurrent tumor."

Section 9.2 CTCAE Routine Reporting Requirements

- Grade 1 AE's with the Attribution Required for Routine AE Data Submission need to be reported. The second column has been updated to reflect this change.

Section 9.4 Comprehensive Adverse Events and Potential Risks list (CAEPR) for GDC-0449 (Vismodegib, NSC 747691)

An updated CAEPR for vismodegib (Version 2.3, September 15, 2015) has replaced the previous version. In this updated version the following revisions have been made:

- Added New Risks:
    - Also Reported on GDC-0449 Trials But With the Relationship to GDC-0449 Still Undetermined: Ascites; Blood bilirubin increased; Gastrointestinal disorders - Other (small intestinal hemorrhage); Hemorrhoidal hemorrhage; Hypernatremia; Ileus; Lipase increased; Myocardial infarction; Nail ridging; Peripheral motor neuropathy; Stomach pain

- Increase in Risk Attribution:
    - Changed to Likely from Less Likely: Weight loss

- Decrease in Risk Attribution:
    - Changed to Reported But Undetermined from Less Likely: Abdominal pain; Arthralgia; Constipation; Headache; Infection; Vomiting

Section 10.1 Vismodegib (GDC-0449, Erivedge®, NSC# 747691, IND#126926) IND holder: Alliance

- In the section entitled "Formulation", the supply information for vismodegib has been removed as this information can be found on the drug order form.
- The most common all grade adverse events have been removed from the "Adverse Events" section as this information can be found in the CAEPR.

Appendix II 1.5T & 3T ADVANCED MRI PROTOCOL

- The section title has been revised from "Standard MRI Protocols" to "1.5T & 3T Advanced MRI Protocol."
- In addition, the following phrase has been added beneath the section title: For sites acquiring DCE Imaging."
- This table has been updated to reflect the combined standard and advanced imaging protocol.
- Footnote "a" has been changed from "0.1 mmol/kg dose injection with a Gadolinium chelated contrast agent. Use of a power injector is desirable at an injection rate of 3-5cc/sec" to "After 30 seconds of baseline image acquisition, a bolus injection of 0.1 mMol/kg of Gadolinium chelated contrast agent is administered as part of the DCE acquisition. Continue to acquire dynamic data for 8 minutes post contrast injection for a total scan time of 8-9 minutes. Use of a power injector is desirable at an injection rate of 3-5cc/sec."

CHANGES TO THE MODEL CONSENT

What possible risks can I expect from taking part in this study?

- The first sentence of the paragraph above the section entitled "Possible Side Effects of Vismodegib" has been revised to include the following for clarity: "If you are taking GSK2256098…"

The risk list for Vismodegib has been updated to reflect the NCI's new condensed risk list profile.
In this updated version the following revisions have been made:

- Increase in Risk Attribution:
  - Changed to Common from Occasional: Weight loss

- Decrease in Risk Attribution:
  - Changed to Reported But Undetermined from Less Likely (i.e., removed from the Risk Profile): Constipation; Headache; Infection; Pain; Vomiting

Optional Imaging Study

- Recognizing that not all institutions would be able to participate in the advanced imaging sub-study, the section has been revised for the site to communicate their ability to participate to the patients. Patients at institutions that are not able to do advanced images should answer "no" as the imaging sub-study is not applicable.

Samples For The Laboratory Studies and Samples For Future Research Studies

- Questions 3 and 4 were previously incorrectly numbered, and have been renumbered correctly.

A replacement protocol document and model consent form have been issued

---

ATTACH TO THE FRONT OF EVERY COPY OF THIS PROTOCOL

Protocol Update #03
06/15/2016

ALLIANCE FOR CLINICAL TRIALS IN ONCOLOGY

PROTOCOL UPDATE TO ALLIANCE A071401

PHASE II TRIAL OF SMO/AKT/NF2 INHIBITORS IN PROGRESSIVE MENINGIOMAS WITH SMO/AKT/NF2 MUTATIONS

Industry-supplied agent(s): Vismodegib (IND #126926) and GSK2256098 (IND #126926); IND holder: Alliance

X	**Update:**		**Status Change:**
X	Eligibility changes		Activation
	Therapy / Dose Modifications / Study Calendar changes		Closure
X	Informed Consent changes		Suspension / temporary closure
	Scientific / Statistical Considerations changes		Reactivation
	Data Submission / Forms changes		
	Editorial / Administrative changes		
	Other :		

Expedited review is allowed. IRB approval (or disapproval) is required within 90 days. Please follow your IRB of record guidelines.

CHANGES TO THE PROTOCOL

Section 3.3 (Eligibility Criteria)
In Section 3.3.9 (Required Initial Laboratory Values) , the UPC value has been corrected from "UPC $\geq$ 45 mg/mmol" to "UPC $\leq$ 45 mg/mmol."

Section 4.3 (CTSU Site Registration Procedures)

- The last paragraph of the section has been modified as requested by CTSU. Specifically, instructions have been added regarding submission and approval of the Study Specific Worksheet for Local Context (SSW).
- In Section 4.3.4 (Submitting Regulatory Requirements), in the first sentence, the list of materials to submit have been removed and replaced with the text "required forms and documents."

Section 7.0 (Treatment Plan/Intervention)
Under "Arm B (NF2 Mutation)" the following text has been added as the fourth paragraph: "Please note all supply of GSK2256098 for this study expires January 2018. Currently, there are no plans for further supply of GSK2256098 to be manufactured, see Section 10.2."

Section 10.2 (GSK2256098 [NSC# 783781, IND# 126926)

Under "Procurement," the following text has been added as the second paragraph: "GSK2256098 supply has an expiry of January 2018. Currently, there are no plans for further supply to be manufactured, therefore supply of GSK2256098 for this study will expire January 2018."

Section 11.4 (Measurement of Treatment/Intervention Effect)

- In Section 11.4.1 (Measureable lesions), the following underlined text has been added: "Bidimensionally enhancing measurable lesions with clearly defined margins by MRI or CT scan. Necrosis or cystic changes (nonenhancing disease) should not be included in the measurement of tumor area."
- In Section 11.4.3.2 (Evaluation of Measurable Lesions), the following changes have been made:
  - In bullet "a" under "Complete Response," the word enhancing has been added as follows, "Disappearance of all enhancing lesions on consecutive magnetic resonance…."
  - In bullet "a" under "Partial Response," the word enhancing has been added as follows: "> 50% decrease under baseline in the sum of products of perpendicular diameters all enhancing measureable lesions."
  - In bullet "a" under "Progression," the word enhancing has been added as follows, "≥ 25% increase in the sum of products of all enhancing measurable lesions…."
  - Bullet "b" has been added to provide further guidance and considerations for evaluating disease progression.

CHANGES TO THE MODEL CONSENT

What are the costs of taking part in this study?

The first paragraph has been slightly modified and a new paragraph has been added to inform patients that GSK2256098 will only be available until January 2018, and that they should discuss this with their treating physician.

A replacement protocol document and model consent form have been issued

---

ATTACH TO THE FRONT OF EVERY COPY OF THIS PROTOCOL

Protocol Update #04
XX/XX/2016

ALLIANCE FOR CLINICAL TRIALS IN ONCOLOGY

PROTOCOL UPDATE TO ALLIANCE A071401

PHASE II TRIAL OF SMO/AKT/NF2 INHIBITORS IN PROGRESSIVE MENINGIOMAS WITH SMO/AKT/NF2 MUTATIONS

Industry-supplied agent(s): Vismodegib (IND #126926) and GSK2256098 (IND #126926); IND holder: Alliance

X	**Update:**		**Status Change:**
X	Eligibility changes		Activation
	Therapy / Dose Modifications / Study Calendar changes		Closure
X	Informed Consent changes		Suspension / temporary closure
	Scientific / Statistical Considerations changes		Reactivation
	Data Submission / Forms changes		
	Editorial / Administrative changes		
X	Other : Vismodegib CAEPR Update		

The changes included in this update to Alliance A071401 have been made in response to the NCI Action Letter from Dr. Naoko Takebe dated June 13, 2016. This Action Letter is posted on the A071401 Study Page on the Alliance web site. A revised CAEPR with new risks has been added to the protocol. Therefore, the model consent form has been revised to incorporate these new risks consistent with the new NCI Model Consent Template instructions. There are no changes to the risk/benefit ratio.

Expedited review is allowed. IRB approval (or disapproval) is required within 90 days. Please follow your IRB of record guidelines. Please follow the policy of your IRB of record regarding notifying patients of new information contained in this update.

UPDATES TO THE PROTOCOL

Section 3.3 (Registration Eligibility Criteria)
In Section 3.3.5, the eligible age for enrollment has been changed due to a safety report for vismodegib. For patients with SMO mutation who will be assigned to receive vismodegib, only patients age 30 and over are eligible to register. Patients with NF2 mutation, patients age 18 and over are eligible.

Section 9.4 (Comprehensive Adverse Events and Potential Risks list [CAEPR] for GDC-0449 [Vismodegib, NSC 747691])

This section has been modified to include the updated Vismodegib CAEPR (Version 2.4, April 1, 2016) provided by CTEP. Changes from Version 2.3 to Version 2.4 have been outlined below:

- Added New Risk:
  - Reported but With Insufficient Evidence for Attribution: Esophageal pain; Febrile neutropenia; Hip fracture; Hypoxia; Vasculitis

- Increase in Risk Attribution:
  - Changed to Likely from Less Likely: Anorexia
  - Changed to Likely from Reported but With Insufficient Evidence for Attribution: Irregular menstruation
  - Changed to Less Likely from Reported but With Insufficient Evidence for Attribution: Arthralgia; Constipation; Dyspepsia; Vomiting

- Provided Further Clarification
  - Gastrointestinal disorders - Other (small intestinal hemorrhage), Hemorrhoidal hemorrhage, Retroperitoneal hemorrhage, and Upper gastrointestinal hemorrhage (all previously under Reported but With Insufficient Evidence for Attribution) are now reported as Gastrointestinal hemorrhage (under Reported but With Insufficient Evidence for Attribution).
  - Eye disorders - Other (central retinal vein occlusion) (previously under Reported but With Insufficient Evidence for Attribution) is now reported as Retinal vascular disorder (under Reported but With Insufficient Evidence for Attribution).
  - Footnote #2, "Irregular menstruation was observed in 30% (3 of 10) women of child bearing age and/or in 28% (18 of 64) women who had menses at baseline who were enrolled in studies of advanced BCC" has been added.
  - Footnote #3, "Gastrointestinal hemorrhage includes Anal hemorrhage, Cecal hemorrhage, Colonic hemorrhage, Duodenal hemorrhage, Esophageal hemorrhage, Esophageal varices hemorrhage, Gastric hemorrhage, Hemorrhoidal hemorrhage, Ileal hemorrhage, Intra-abdominal hemorrhage, Jejunal hemorrhage, Lower gastrointestinal hemorrhage, Oral hemorrhage, Pancreatic hemorrhage, Rectal hemorrhage, Retroperitoneal hemorrhage, and Upper gastrointestinal hemorrhage under the GASTROINTESTINAL DISORDERS SOC" has been added.

- Modified Specific Protocol Exceptions to Expedited Reporting (SPEER) reporting requirements:
  - Added: Irregular menstruation

- Deleted Risk:
  - Reported but With Insufficient Evidence for Attribution: Acute coronary syndrome; Blurred vision; Bone pain; Chills; Dry skin; Ear and labyrinth disorders - Other (autophony); Edema face; Electrocardiogram QT corrected interval prolonged; Eye disorders - Other (blepharitis); Eye disorders - Other (central retinal vein occlusion); Gastrointestinal disorders - Other (altered saliva); Gastrointestinal disorders - Other (discolored stool); Gastrointestinal disorders - Other (eructation); Gait disturbance; Hepatobiliary disorders - Other (cholestasis); Hepatobiliary disorders - Other (hepatitis); Hepatobiliary disorders - Other (hepatotoxicity); Hirsutism; Hot flashes; Joint range of motion decreased; Metabolism and nutrition disorders - Other (cachexia); Metabolism and nutrition disorders - Other (increased appetite); Metabolism and nutrition disorders - Other (salt craving); Multi-organ failure; Nail loss; Pain of skin; Purpura; Skin and subcutaneous tissue disorders - Other (abnormal hair growth); Skin and subcutaneous tissue disorders - Other (hair texture abnormal); Skin and subcutaneous tissue disorders - Other

(ingrown hair); Skin and subcutaneous tissue disorders - Other (milia); Skin and subcutaneous tissue disorders - Other (skin fragility); Tinnitus; Urinary frequency; Vertigo; Watering eyes; Wound complication

UPDATES TO THE MODEL CONSENT

What possible risks can I expect from taking part in this study?

Under the bolded text, "Possible side effects of Vismodegib," the NCI has made the following changes to the condensed risk profile based on the revisions to the Vismodegib CAEPR described above:

- Increase in Risk Attribution
  - Changed to Common from Occasional: Loss of appetite
  - Changed to Common from Reported but With Insufficient Evidence for Attribution: Abnormal menstrual period
  - Changed to Occasional from Reported but With Insufficient Evidence for Attribution: Pain in joints; Constipation; Heartburn; Vomiting

A replacement protocol document and model consent form have been issued

---

ATTACH TO THE FRONT OF EVERY COPY OF THIS PROTOCOL

Protocol Update #05
XX/XX/2016

ALLIANCE FOR CLINICAL TRIALS IN ONCOLOGY

PROTOCOL UPDATE TO ALLIANCE A071401

PHASE II TRIAL OF SMO/AKT/NF2 INHIBITORS IN PROGRESSIVE MENINGIOMAS WITH SMO/AKT/NF2 MUTATIONS

Industry-supplied agent(s): Vismodegib (IND #126926) and GSK2256098 (IND #126926); IND holder: Alliance

X	**Update:**		**Status Change:**
	Eligibility changes		Activation
	Therapy / Dose Modifications / Study Calendar changes		Closure
X	Informed Consent changes		Suspension / temporary closure
X	Scientific / Statistical Considerations changes		Reactivation
	Data Submission / Forms changes		
X	Editorial / Administrative changes		
X	Other: Added requirement for all scans to be submitted for retrospective central radiology review		

Expedited review is allowed. IRB approval (or disapproval) is required within 90 days. Please follow your IRB of record guidelines.

UPDATES TO THE PROTOCOL

Cover Page
Meagan Wilts has replaced Carla Hilton as the data manager. Contact information has been updated accordingly.

Section 1.2 (Genetic Analysis of Meningioma)
In the first paragraph, a new second from last sentence has been added for clarity ("Additionally, 7% of NF2-wildtype meningiomas harbor oncogenic alterations in PIK3CA").

Section 1.11 (Central Radiology Review)
With this update, the protocol will be modified to require all scans to be submitted for retrospective central radiology review. This section has been added to provide background regarding this change.

Section 2.2 (Secondary Objectives)
Objective 2.2.3 has been added to include the new objective to determine activity of SMO and FAK inhibitor as measured by response rate by central radiology review.

Section 3.3 (Registration Eligibility Criteria)
In Section 3.3.7 (Patient History), the word "No" has been adding to the beginning of bullets 4-9 for clarity.

Section 5.0 (Study Calendar)
- Footnote 3 has been revised to state that all MRIs must be submitted to the Imaging Core Laboratory, not just DCE MRI.
- A new final sentence has been added to Footnote A: "All MRIs should follow the consensus MRI protocol outlined in Appendix II even if the site is not acquiring the DCE sequence."

Section 6.5 (CT and MRI Imaging Data Submission)
- A new first paragraph has been added (starting with "Acquisition of MR imaging…") in order to state the defined MR imaging parameters.
- The second paragraph of Section 6.5 has been modified to state that all MR images should be submitted to IROC. A sentence has also been added to state that any MRI performed prior to approval of Update #05 should be transmitted to IROC.
- In the third paragraph, a new final sentence has been added for clarity: "The DCE MRI acquisition protocol is outlined in Appendix III."
- In the fourth paragraph, the phrase, "For all patients," has been added to the first sentence.

Section 9.3 (Expedited Adverse Event Reporting [CTEP-AERS])
At the bottom of the table in Section 9.3.1, the following statement has been removed "NOTE: Deaths clearly due to progressive disease should NOT be reported via CTEP-AERS but rather should be reported via routine reporting methods (e.g., CDUS and/or CTMS)." Below the table, this statement also appeared as the seventh bullet under the heading "Additional Instructions or Exclusions to CTEP-AERS Expedited Reporting Requirements for Phase 1 and Early Phase 2 Trials Utilizing an Agent Under a non-CTEP IND:" Therefore, the seventh bullet has been removed as this exclusion is not correct. All deaths, even those clearly due to progressive disease are required to be submitted as an adverse event via CTEP-AERS.

Section 11.0 (Measurement of Effect)
The following sentence has been added to the end of the section: "Primary endpoint will be based on local radiology review. Central radiology review will be carried out for measurement of secondary endpoint."

Section 12.1 (Duration of Treatment)
In Section 12.1.1 (CR, PR or SD), the following underlined text has been added for clarity: ": Patients who are in CR, PR or SD , as assed by local radiology review, will continue on therapy…."

Section 13.4 (Supplementary Analysis Plans)
- The following underlined text has been added to the first sentence: "Overall survival and progression free survival will be summarized…."
- A third paragraph has been added to outline how response rate will be determined by central review.

Appendix II (Required Consensus MRI Acquisition Parameters)
- Appendix II has been added in order to provide a clear distinction between the required consensus MRI acquisition parameters (formerly referred to as "Standard MRI Protocols") and the advanced

DCE imaging protocols that are to be used for patients who elect to participate in the advanced imaging study.

- Above the tables, a second paragraph describing the MRI acquisition parameter requirements has been added. A third paragraph has also been added that states: “For any patients enrolled prior to Update #05 (or with images acquired prior to Update #05), MRI parameters should remain consistent with baseline or prior image acquisition protocols.”
- In the 1.5T table, the following changes have been made:
  - Footnote j has been added below the table, which reads: “FOV and matrix size should be chosen to keep resolution *less than* 1.5mm isotropic voxel size. Note that all voxel measurements should be equal in x, y, and z dimensions.”
  - In the “FOV” row, the following has been added in the “3D T1 Pre” and “3D T1 Post” columns: “(for ≤ 1.5mm isotropic)[j]”
  - In the “Slice Thickness” row, references to footnote j have been added in the “3D T1 Pre” and “3D T1 Post” columns.
  - In the “Parallel Imaging” row, the text has been changed from “Yes-If available” to “Up to 2x.
- In the 3T Protocol table, the following changes have been made:
  - Footnote i has been added below the table, which reads: “FOV and matrix size should be chosen to keep resolution at 1mm isotropic voxel size. Note that all voxel measurements should be equal in x, y, and z dimensions.”
  - In the “FOV” row, references to footnote i have been added in the “3D T1 Pre” and “3D T1 Post” columns.
  - In the “Slice Thickness” row, references to footnote i have been added in the “3D T1 Pre” and “3D T1 Post” columns.

Appendix III 1.5T & 3T ADVANCED MRI PROTOCOL

- The 1.5T and 3T advanced MRI protocols have been separated out into two separate tables.
- The parameters for 1.5T and 3T MRI have been completely revised for clarity and to allow for whole brain coverage given that meningiomas can be larger and occasionally there can be multiple.

CHANGES TO THE MODEL CONSENT

What extra tests and procedures will I have if I take part in this study?

At the end of the section, a paragraph has been added to describe that image submission to a central image library is now required as part of the patient’s study participation. This was also added to clarify that only the advanced imaging is optional, not image submission.

A replacement protocol document and model consent form have been issued

---

ATTACH TO THE FRONT OF EVERY COPY OF THIS PROTOCOL

ALLIANCE FOR CLINICAL TRIALS IN ONCOLOGY

PROTOCOL UPDATE TO ALLIANCE A071401

PHASE II TRIAL OF SMO/AKT/NF2 INHIBITORS IN PROGRESSIVE MENINGIOMAS WITH SMO/AKT/NF2 MUTATIONS

Industry-supplied agent(s): Vismodegib (IND #126926) and GSK2256098 (IND #126926); IND holder: Alliance

X	**Update:**		**Status Change:**
X	Eligibility changes		Activation
X	Therapy / Dose Modifications / Study Calendar changes		Closure
X	Informed Consent changes		Suspension / temporary closure
X	Scientific / Statistical Considerations changes		Reactivation
	Data Submission / Forms changes		
X	Editorial / Administrative changes		
	Other:		

Full Board review recommended. IRB approval (or disapproval) is required within 90 days. Please follow your IRB of record guidelines. No new patients may be consented onto this protocol until IRB approval for this amendment has been obtained.

UPDATES TO THE PROTOCOL

Cover Page

- The radiation oncology co-chair, Paul Brown has moved from MD Anderson to Mayo Clinic. His contact information has been updated accordingly.
- Erin Twohy has replaced Keith Anderson as the secondary statistician.
- Meagan Wilts name has been changed to Meagan Odegaard. Her e-mail address has been updated accordingly.

Cover Page (2)

- Under “Protocol Contacts:,” the fax number has been removed for Roxann Neumann (who manages non-paraffin specimens at the Alliance Biorepository at Mayo Clinic).

- Under “Protocol-related questions may be directed as follows”, the following changes have been made:
    - In the row “Questions regarding CTEP-AERS reporting:,” the phone number for the Regulatory Affairs Manager has been removed. Please direct all questions to regulatory@allianceNCTN.org.
    - In the last row of the table, the text in the first column has been changed from “Questions regarding initial specimens/specimen submission” to “Questions regarding status of central pathology review and biomarker testing.”

Cover Page (3)

The CTSU Address and Contact Information table has been updated with the most recent version provided by CTSU.

Schema

- Under “Pre-Registration Eligibility Criteria,” the first sentence has been changed from “…for central path review and SMO and NF2 testing” to “….for central path review and integral biomarker testing.”
- Under “Registration Eligibility Criteria,” the text that follows has been bulleted for readability. Additionally, the following changes have been made:
    - In the first bullet, PTCH1 has been added as an eligible mutation.
    - In the sixth bullet, reference to “XRT” has been changed to “radiation treatment (XRT, brachytherapy, radiosurgery)” for clarity.
    - A ninth bullet has been added to align with eligibility that states “No craniotomy within 28 days of registration.”
    - In the eleventh bullet for age ≥ 18 years, the text “(for patients with NF2 mutation)” has been added.
    - A twelfth bullet has been added that states, “Age ≥ 30 years (for patients with SMO/PTCH1 mutation).”
    - In the eighteenth bullet, the word “No” has been added to the beginning of the sentence to clarify that patients with current Child Pugh Class B or C liver disease are NOT eligible.
    - A new twenty first bullet has been added that states, “No uncontrolled hypertension defined as BP >140/90.”
    - In the last bullet, the following underlined text has been added for clarity, “No CYP3A4 inhibitors <u>or inducers</u> for 14 days prior to registration <u>or during study treatment (for NF2 patients).</u>”
    - The “Required Initial Laboratory Values” have also been moved down to appear in line with the “Registration Eligibility Criteria” as these lab values are not needed for Pre-Registration.
    - Under “Required Initial Laboratory Values,” the value for UPC has been corrected from “≥ 45 mg/mmol*” to “≤ 45mg/mmol.”
- In the schema diagram, the following changes have been made:
    - The text “Central SMO and NF2 Testing” has been changed to “Central Biomarker Testing” for readability. Throughout the protocol, references to “SMO and NF2 testing” have all been changed to “biomarker testing.”
    - PTCH1 has been added as an eligible mutation. Patients with a PTCH1 mutation identified by central biomarker testing will go on to receive vismodegib. Therefore, this has been indicated in the schema.
    - The text “If negative SMO or NF2” has been changed to “If negative for mutation or biomarker cohort is closed.” Additionally, instead of these patients going “Off Study” the text has been changed to “Patient does not continue to registration.”

Section 1.3 (Available Agents that act on SMO, PTCH1, AKT and NF-2 mutated tumors)

- “PTCH1” has been added to the section title.
- The first paragraph has been completely revised to clarify that the tumor samples will be tested as described in Appendix VII, and that results will only be reported for mutations with an available agent.
- The second paragraph has been modified to include reference to PTCH1 mutations, as this mutation will lead to aberrant activation of the Hedgehog (Hh) pathway.

Section 2.1 (Primary objectives)

Reference to PTCH1 has been added to objective 2.1.1.

Section 2.3 (Correlative Science Objectives)

Objective 2.3.3 has been added that reads: “To evaluate volumetric response by central radiology review.”

Section 3.0 (Patient Selection)

The second paragraph has been removed that instructed sites to go to the A071401 study page to check cohort status and accrual, and contact the Alliance Registration Office for spot confirmation. A new process has been added to Section 4.4 with this update.

Section 3.3 (Registration Eligibility Criteria)

- In Section 3.3.1 (Documentation of Disease), the text under “Molecular Documentation” has been completely revised. Specifically, PTCH1 has been added as an eligible mutation. Additionally, the specific mutations (i.e. SMO W535L, SMOL412F;; etc.) have been removed as this information can be found in Appendix VII.
- In Section 3.3.3 (Prior Treatment), in the fifth bullet, reference to “XRT” has been changed to “radiation treatment” for clarity.
- In Section 3.3.5, PTCH1 mutation has been added as follows as these patients will be receiving vismodegib, “For patients with SMO/PTCH1 mutation: Age ≥ 30 years.”
- In Section 3.3.8 (Concomitant Medications), the second bullet has been modified to require drug discontinuation 14 days prior to registration (as opposed to 14 days from start of study treatment) to align with the bullet for CYP3A4 inhibitors that precedes it.

Section 4.3 (CTSU Site Registration Procedures)

This section (including subsections 4.3.1, 4.3.2, 4.3.3 and 4.3.4) has been updated with the most recent site registration language provided by CTSU.

Section 4.4 (Patient Pre-Registration Requirements)

- In the second bullet for Cohort Status and Accrual, all but the first sentence has been removed. This language directed sites to contact the Alliance Registration Office for spot confirmation, and this process is no longer in place. New text has been added to instruct sites to take into account any local diagnosis and biomarker results as they consider patients for pre-registration, and contact the study chair and protocol coordinator should they have any questions.
- In the third bullet “Central pathology review...,” the second sentence has been revised as follows: “ALL diagnostic H&E~~’s and one tissue block must be submitted~~ slides must be submitted together with 15-25 unstained slides cut from the FFPE block that contains representative tumor tissue (See Section 6.~~0~~2.”

Section 4.5 (Patient Registration Procedures)

- The text in this section has been divided into multiple bullets with headings for readability.
- In the first bullet, the following changes have been made:
  - In the first sentence, of the first bullet, the following underlined text has been added and strikethrough text removed: "Sites will be notified via e-mail whether or not the patient is eligible ~~based on the central pathology and central molecular review~~ within ~~10 business days of delivery~~ 21 days of receipt of a suitable patient tumor specimen ~~to~~ by the MGH Translational Research Laboratory."
  - As of this update, biomarker testing will be performed prior to central pathology review. Therefore, in the second sentence, the following strikethrough text has been removed and underlined text added: "If a sample ~~has been deemed unsuitable after central pathology review based on differential diagnosis,~~ has insufficient tissue amount and/or tumor cellularity, ~~the sample will NOT undergo SMO and NF2 testing and~~ a request for an alternative sample will be made.
  - New third and fourth sentences have been added that read as follows: "Specimens will be routed as outlined in Section 6.2 and testing will be performed as outlined in Appendix VII. Patient eligibility will be based on central biomarker testing, central pathology review (only if eligible mutation positive), and cohort status (i.e. if no slots are available in the identified cohort, the patient will be deemed ineligible)."
- In the third bullet, the following text has been added: "Please note: Once accrual to the patient cohort has been completed, no additional patients may be able to register (even if the patient is deemed eligible from central testing because a slot was available at that time). The Alliance Registration Office will communicate this back to the site."
- In the fourth bullet, the word "molecular" has been removed as follows, as patient eligibility is also based on cohort accrual status: "Registration must occur within 14 days of receiving notification of patient ~~molecular~~ eligibility from the central testing laboratory."

Section 4.8 (Treatment Assignments and Patient Cohorts)

- In the first paragraph, the second sentence has been revised to add reference to the *PTCH1* mutation.
- In the second paragraph (that provides instructions for registration and crossover/re-registration for patients with more than mutation), a new second sentence has been added to instruct institutions to contact the protocol coordinator and study chair prior to registration to confirm which arm should be selected for patient registration. Additionally, the fourth and fifth sentences have been removed that provided enrollment instructions for patients who wish to crossover upon discontinuation of study agent. Instead, language has been added to contact the protocol coordinator and study chair prior to crossover/re-registration to confirm that study drug is available and to coordinate re-registration through the Alliance Registration Office.
- In the listing for specific treatment groups, PTCH1 mutation has been added as a mutation for Group 3 and 4.

Section 5.0 (Study Calendar)

- Gridlines have been added to the table for readability.
- Within the table, under Tests & Observations, reference to footnote ! has been removed after EKG. Therefore, below the table, in footnote $, the following sentence has been added: "Required only for patients with NF2 mutation enrolling/enrolled on GSK2256098."
- Under the table, in footnote A, the last sentence has been removed that stated that all MRIs should follow the consensus MRI protocol outlined in Appendix II, as the footnote placement and wording was confusing. As a result, under the table in footnote 3, the following sentence has been added for clarity: "All MRIs should follow the consensus MRI protocol outlined in Appendix II, unless the patient has consented to the optional DCE substudy, in which case all MRIs should follow the MRI protocol outlined in Appendix III."

Section 6.2 (Specimen Collection and Submission)

- Sections 6.2 (Specimen Collection and Submission), Section 6.3 (Tissue Collection and Processing for Histopathology Review) and Section 6.4 (Blood sample submission) have been consolidated and renumbered to fall within Section 6.2. Subsequent subsections have been renumbered accordingly.
- In Section 6.2 (Specimen Collection and Submission), the following changes have been made:
  - Under "For all patients registered to Alliance A071401," the following changes have been made:
    - In the first paragraph, the first sentence has been completely revised to clarify that H&E slides will be used for central pathology review and FFPE tumor biopsy or surgical tissue will be used for biomarker testing. The second sentence has been completely revised to specify what type of diagnostic slides should be submitted for central pathology review. In the second sentence, reference to footnote ** has been removed. The third sentence has been revised to clarify that the specimens should be submitted to together.
    - The second paragraph has been completely revised to change the order of central pathology review and biomarker testing. Previously, central pathology review was performed first and then biomarker testing. To expedite result reporting back to institutions, Dr. Borger will first perform biomarker testing and if an eligible mutation is found, the residual tissue will be forwarded to Dr. Santagata for central pathology review.
    - In the third paragraph, reference to the PTCH1 mutation has been added. A final sentence has also been added to direct sites to Appendix VII for testing procedures.
    - The fourth paragraph has been removed that read: "Typical turnaround time for central path review is within 3 working days and for SMO and NF2 mutation testing within 10 days of receipt of the slides and tissue." The following text has been added in its place: "Results will be returned to the site within 21 days of specimen receipt at Massachusetts General Hospital."
  - The specimen submission table and footnotes have been completely revised and reformatted for clarity. Instructions for specimen collection, preparation and submission previously contained within footnotes can now be found in the respective subsections of Section 6.2. Modifications to submission instructions include the following:
    - A new column has been added titled "Every 4 cycles during treatment*." This timepoint was previously included under footnote 4 as "every 16 weeks while patient is on study." Footnote * has been added that reads: "Prior to cycles 5, 9, 13, 17, etc."
    - In the first column of the second row under "Mandatory for all patients registered to A071401," the requirement to submit "One paraffin block containing at least 1 cm^2 of viable tumor" has been changed to "15-25 unstained slides of tumor tissue cut from a single paraffin block containing at least 1 cm^2 of representative and viable tumor tissue."
    - In the "Submit to:" column for the specimens included under "Mandatory for all patients registered to A071401:," the institution has been changed from "Brigham/Dana-Farber Cancer Institute" to "Massachusetts General Hospital."
- In Section 6.2.1 (Specimen submission using the Alliance Biospecimen Management System), the following changes have been made:
  - Under "ALL tumor tissue for Central Pathology Review…," the shipping address has been changed. Specimens for central pathology review and biomarker testing should be sent to Dr. Darrel Borger's lab at Massachusetts General Hospital. Additionally, contacts have been added for whom to direct questions to related to receipt of specimens or status of central review and biomarker testing.

- Under “Blood submission for patients who agree to participate:” the fax number for Roxann Neumann has been removed.
- Under “Tissue submission for the correlative studies for patients who agree to participate,” the name and address for the Alliance Biorepository at Mayo Clinic has been changed from “Alliance Operations Office” to “Alliance Biorepository at Mayo Clinic FFPE Tissue.” This change has been made in order to ensure that specimens are delivered to this location, so please use this new address for recurrent tissue specimen submission on A071401-ST1.The Mayo Biorepository for tissue specimen submission has been referred to throughout the protocol as “Mayo FFPE.”

• Section 6.3 has been renumbered as Section 6.2.2 (Tissue collection and processing for histopathology review [mandatory for all patients]).Within Section 6.2.2 the following changes have been made:
  - In the first paragraph, the first sentence has been revised as follows: “Submission of ALL diagnostic H&E slides and ~~at least 1 paraffin block~~ 15-25 unstained slides of FFPE tumor tissue from the original ~~diagnosis~~ diagnostic tissue is required for all patients enrolled on the study.” The third sentence about recurrent tissue submission has been removed as this now appears in Section 6.2.3 (Recurrent tissue submission to Alliance Biorepository at Mayo FFPE [A071401-ST1]).
  - Former subsections 6.3.1 and 6.3.2 now appear as the second and third paragraphs under this section.
  - The sentence “Central pathology review is required prior to registration” has been removed as this is repeated throughout the protocol. The sentence previously appeared as the first sentence of Section 6.3.1.
  - A fourth paragraph has been added to provide clear instructions for unstained slide preparation and submission.
  - A fifth paragraph has been added to direct sites to contact the MGH lab if they are unable to cut slides for central submission, and that submitting an alternative may delay turnaround time.
  - A sixth paragraph has been added to remind sites to accompany tissue submission with a de-identified surgical pathology report.
  - A seventh paragraph has been added to remind sites to accompany tissue submission with a completed “Central Pathology and Biomarker Results Form.”
  - The eighth paragraph (formerly Section 6.3.3) has been revised to state that residual material from central pathology and biomarker review will be batch shipped every three to five months to the Mayo FFPE. Previously, it stated that upon enrollment material would be sent for storage.

• Section 6.3.4 (Collection of paraffin blocks of archived meningioma tumors) now appears as Section 6.2.3 (Recurrent tissue submission to Alliance Biorepository at Mayo FFPE [A071401-ST1]). The following changes have been made within this subsection:
  - References to the “Alliance Biorepository at Mayo Clinic” have been specified to “Alliance Biorepository at Mayo Clinic FFPE” throughout this section.
  - In the second to last paragraph, instructions for slice thickness and labeling have been added.

• Section 6.4 (Blood sample submission) now appears as Section 6.2.4 (Blood sample submission [A071401-ST1]). The following changes have been made within this subsection:
  - References to the “Alliance Biorepository at Mayo Clinic” have been specified to “Alliance Biorepository at Mayo Clinic BAP” throughout this section.
  - In the first bullet under “Whole blood for circulating tumor DNA (ctDNA) plasma,” the timepoints for collection (i.e. pre-registration and every four cycles) have been added to the first paragraph. Additionally, the fifth bullet has been modified to reference Mayo BAP and direct sites to shipping instructions in Section 6.2.1.

- At the end of the section, in footnote *, a sentence has been added to direct questions regarding acceptable cryovial choices to the Mayo BAP contact.

Section 7.0 (Treatment Plan/Intervention)

- Reference to *PTCH1* mutations have been added. Patients with *PTCH1*-mutation will receive vismodegib.
- Under the table for Arm A, two sentences have been added to provide instructions for what to do if a dose of vismodegib is missed. These sentences previously appeared as part of a paragraph under the table for Arm B, therefore, the paragraph has been removed.
- Under the table for Arm B, the following sentence has been removed as it was repetitive: “In patients harboring NF2-mutated meningioma, GSK2256098 will be administered at 750 mg po twice daily.”

Section 11.4 (Measurement of Treatment/Intervention Effect)

In Section 11.4.3.2 (Evaluation of Measureable Lesions), in progression subbulett “b,” the phrase “in the first 16 weeks of therapy” has been removed as no new lesions/sites in the scans can appear in any scans (not just those in the first 16 weeks of therapy).

Section 13.1 (Study Design)

- The entire section has been revised to provide more specific definitions and analysis plans of the two co-primary endpoints of response rate (RR) and progression-free survival at 6 months (PFS6).
- A new paragraph has been added to state that the grade II/III NF2 mutation cohort will be expanded with this update.

Section 13.2 (Statistical Design and Analysis for the Primary Endpoint)

- The following sentence has been removed from the section: “The statistical design and primary endpoint is the same for each treatment arm within the trial.”
- Section 13.2.1 (Primary Endpoints) has been revised in its entirety to clarify that 1) PFS6 is defined as the number of patients not having progressive disease or death within six months of the first day of treatment divided by the total number of evaluable patients; 2) RR is defined as the number of responses divided by the total number of evaluable patients; and 3) Patients who do not experience progressive disease but who withdraw from protocol treatment and re-register onto the second tumor mutation arm will not be evaluable for PFS6 endpoint for the efficacy analysis for the first tumor mutation arm.
- In Section 13.2.2 (Statistical Design), the following changes have been made:
  - The first paragraph has been removed that stated that a total of 12 patients per cohort will be enrolled for a total of 48 patients. With Update #06, the NF2 Grade II/III Cohort will be expanded to include 24 patients.
  - The entire section has been revised to separate the statistical design into sections for the SMO/PTCH1 mutation arm and the NF2 mutation arm.
  - For the SMO/PTCH1 mutation arm, the statistical design has been subdivided into each mutation defined cohort (i.e. Grade II/III and Grade I) with updated power calculations.
  - Three paragraphs have been added to describe the statistical design for the NF2 arm, which has been expanded with this update to 36 total evaluable patients as follows: NF2 Grade II/III cohort will accrue a total of 24 evaluable patients, and NF2 Grade I will accrue 12 evaluable patients. Power calculations have been updated accordingly for the NF2 arm and each cohort.
  - Additionally, the overall power and family-wise alpha considerations have been updated.
- In Section 13.2.3 (Analysis Plan), the following changes have been made:
  - The first paragraph has been modified to state that the final analysis will be done after the last evaluable patient has been followed for each 6 months in each analysis cohort (defined by mutation status, tumor grade and specific primary endpoint).

- The RR and PFS6 benchmarks for considering the treatment "active" for both arms, now only applies to SMO/PTCH1 mutation arm (as the accrual for the NF2 arm has been increased).
- A paragraph and three subbullets have been added to outline the RR and PFS6 benchmarks that must be observed for the treatment to be considered active.

Section 13.3 (Sample size, accrual time and study duration)

- In Section 13.3.1 (Sample size), the entire paragraph has been replaced with updated accrual figures as the total study accrual has been increased from 48 evaluable patients to 60 evaluable patients. Additionally, the maximum target accrual has been increased from 56 patients to 69 patients.
- In Section 13.3.2 (Accrual rate and accrual duration), the accrual figures have been updated as follows: 24 SMO/PTCH1 mutation and 36 NF2 mutation evaluable patients. The anticipated maximum accrual duration has also been increased from 3 to 4 years.

Section 13.4 (Supplementary Analysis plans)

In the third paragraph, text has been added to the first sentence to clarify that central review will be performed using bi-dimensional measurements for secondary endpoints and volumetric analysis for exploratory aims.

Section 13.8 (Inclusion and Women and Minorities)

The table has been updated to reflect the increase in maximum target accrual from 56 to 69 patients.

Section 14.1 (Correlative Studies using Biospecimens [Alliance A071401-ST1])

In Section 14.1.1.1 (Background), reference to PTCH1 has been added to the first sentence.

Appendix VII (Central Laboratory Genotype Testing Procedures)

- Under the appendix title, the following strikethrough text has been removed, "Integral Molecular Testing ~~for SMO or NF2 Mutation~~."
- Under "Background," PTCH1 has been added as a gene that will be evaluated. Additionally, the last two sentences have been removed and replaced with the following: "This assay has been clinically-validated, and standard operating procedures have been created, and will be locked down during the duration of the trial.While a total of 91 genes are targeted by this assay, only mutations in SMO, PTCH1 and NF2 will be evaluated for determining patient eligibility and reported to the site and the Alliance."
- The entire paragraph under "Target Mutations" has been revised to 1) specify additional SMO gene mutations and state that if additional mutations in SMO are potentially activating mutations, they will be reported as potentially eligible.
- Under "Target Mutations," information about PTCH1 gene evaluation has been added. References to PTCH1 mutation have also been added throughout the appendix.
- In bullet #1, the following statement has been added for clarity: "Since the SNaPshot detects mutations across a number of cancer genes, ineligible mutations (i.e. those other than SMO, PTCH1, NF2) may also be identified, however, these will not undergo a complete evaluation."
- Bullet #2 has been completely revised to better describe the specimens that will be used for biomarker testing.
- In bullet #3, the second sentence has been revised. It now appears as the following two sentences: "The MGH TRL program coordinator will verify that the sample received matches the information provided by the site, as recorded on the "ALLIANCE A071401 Central Pathology And Biomarker Results Form" that must be included with the specimen. Sample acceptability will be determined based on shipping and received condition and sample identifiers will be confirmed before initiating the testing process." A final sentence has also been added to speak to delays in turnaround time caused by improper sample submission, labeling or missing "Central Pathology and Biomarker Results Form."

- Bullet #5 has been added; it states: “The H&E slide that corresponds to the tissue sections submitted to the MGH TRL will be reviewed by a pathologist and the tumor area to be extracted will be marked.” Subsequent bullets have been renumbered.
- In bullet #7, the statement has been removed that referred sites to Section 14.2 for the list of specific genetic alterations being interrogated as these can be found towards the beginning of the appendix.
- In bullet #8, in the second sentence “Mutect and Oncotator bioinformatics tools developed at the Broad institute” has been changed to “MGH CIDer bioinformatics tool.”
- Bullet #10 has been added; it reads: “If NO eligible mutation was identified during testing, the site and the Alliance will be notified of biomarker ineligibility. Central Pathology Review will not be required or performed.” Subsequent bullets have been renumbered.
- Bullet #11 has been added; it reads: “If an eligible biomarker for SMO, PTCH1, or NF2 was found, all diagnostic H&E slides received for that case will be forwarded to Dr. Sandro Santagata for Central Pathology Review to confirm diagnosis and tumor grade. The completed and signed central pathology review form will be electronically forwarded to Dr. Darrell Borger for inclusion in final reporting.” Subsequent bullets have been renumbered.
- In bullet #12, the expected turnaround time has been extended from two weeks to 21 days. The last sentence has also been removed; which read: “Central laboratory confirmation testing may be performed in batch with a longer turnaround time, if required.”

UPDATES TO THE MODEL CONSENT

Why is this study being done?

- In the first paragraph, reference to PTCH1 has been added as a meningioma mutation of interest in the fifth sentence. Reference to PTCH1 has also been added to the sixth sentence. Additionally, the following text has been added as the second to last sentence: “SMO and PTCH1 are part of the same pathway, called the Hedgehog pathway.” Additionally, the last sentence has been changed from “…that target these 2 genes” to “…that target these genes.”
- In the second paragraph, the first sentence has been changed from “…vismodegib, blocks the SMO receptor” to “…vismodegib, blocks the Hedgehog pathway.”
- In the third paragraph, the last sentence has been revised to state that 41 people will be taking part in the GSK2256098 group (previously, it was 28).
- In the fourth paragraph, the first sentence has been changed to say that 69 people will be taking part in the study (previously, it was 56). The following text has been added as the second sentence: “There may be as many as 843 people who will have their tissue tested for mutations in order to find 69 people to take part in the study.”

What are the study groups?

- In the first paragraph, the first sentence has been revised and split into two sentences. The sentence previously read: “…researchers will look at the tissue of your tumor to see if you have the SMO, or NF2 mutation.” The sentences now read, “…researchers will look at the tissue of your tumor to see what types of mutations it has across different types of genes. If you have the SMO, PTCH1, or NF2 mutation, then you may be eligible to participate on the study.”
- In the second paragraph, PTCH1 has been added as patients with this mutation will go on to receive vismodegib.
- The following text has been added as the fifth paragraph: “There are only a certain number of spots available on the trial for each type of mutation (for example, SMO/PTCH1 or NF2). If you are found to have one of the mutations listed above, but there are no more spots left for that mutation, then you will not be eligible to participate on the study (so you will not receive any of the medicines listed above).”
- The text in the second step in the schema has been changed from “Your tumor is tested for 2 genes, SMO and NF2” to “Your tumor is tested for mutated genes (including SMO, PTCH1 and NF2).”

- Under “Group 1” in the schema, reference to PTCH1 has been added as patients with PTCH1 mutations will be assigned to receive vismodegib.

What extra tests and procedures will I have if I take part in this study?

- In the third paragraph, the fourth sentence has been changed from “The tissue sample will be used to test for the SMO and NF2 mutation” to “The tissue sample will be used to test for SMO, PTCH1, NF2, and other mutations.”
- Under “During the study,” the last bullet has been modified to say that patients with PTCH1 mutation will need to take monthly pregnancy tests.

What are the costs of taking part in this study?

- In the first paragraph, the following text has been removed: “The cost of getting the medications ready and giving it to you is also provided at no charge.” This statement was included previously in error and has been removed, as the cost of drug preparation and administration does not apply to this study with oral agent.
- The following text has been added: “The two required EKGs for patients receiving GSK2256098 (patients with NF2 mutation) will be paid for by the study. The monthly pregnancy testing for women who could become pregnant receiving vismodegib (those patients with SMO/PTCH1 mutations) will be paid for by the study. Also, the testing to identify SMO, PTCH1, and NF2 mutations will be paid for by the study.” The sentences that previously followed now appear as the second paragraph.

Optional Sample Collections for Laboratory Studies and/or Biobanking for Possible Future Studies

- In the second paragraph, the following underlined text has been added to the first sentence, “would like to collect blood and use a sample from your prior surgeries or biopsies for research on meningiomas.”
- In the second paragraph, in the second sentence, “ genetic biomarkers” has been changed to “genetic and other tumor-related biomarkers.”

What is involved?

Bullet 1 has been revised for patient readability as follows:

- In the first sentence, the following underlined text has been added and strikethrough text removed: “If you agree to provide additional blood and tissue samples, about 2 tablespoons of blood will be collected from a vein in your arm ~~approximately~~ before you start treatment and every 4 ~~months~~ cycles (or about every 16 weeks), while you are receiving the study drug.”
- A new second sentence has been added that reads: “Leftover tissue from the central testing (which is performed before you start the study) will be saved.”
- A new fourth sentence has been added which read: “If your cancer gets worse or comes back and you are having a new surgery, then a tissue sample from that surgery will also be collected.”
- Text in the fifth sentence has been changed from, “Your sample and some related health information…” to “These samples and some related health information….” Subsequent text now appears as the second paragraph.
- In the first sentence of the second paragraph, The following underlined text has been added and strikethrough text removed, “If you agree to have leftover samples sent to the Biobank, any remaining samples from central testing or the additional research ~~may~~ will be stored in the Biobank….”

How will information about me be kept private?

- In bullet 1, the first sentence has been revised. The following underlined text has been added and

strikethrough text removed, "When your sample~~(s)~~ ~~is~~ <u>are</u> sent to the researchers to test for ~~SMO~~ mutations <u>and other tumor biomarkers</u>, and to look at…."

- In bullet 4, the first sentence has been revised from "The laboratory running the SMO/NF2 testing…" to "The laboratory running the mutation testing…."

<u>Samples for the Laboratory Studies</u>

Model consent question #2 has been changed from "I have to have my specimen collected and I agree…" to "I agree to have my blood and tumor tissue collected and I agree…."

A replacement protocol document and model consent form have been issued

---

ATTACH TO THE FRONT OF EVERY COPY OF THIS PROTOCOL

ALLIANCE FOR CLINICAL TRIALS IN ONCOLOGY

PROTOCOL UPDATE TO ALLIANCE A071401

PHASE II TRIAL OF SMO/AKT/NF2 INHIBITORS IN PROGRESSIVE MENINGIOMAS WITH SMO/AKT/NF2 MUTATIONS

Industry-supplied agent(s): Vismodegib (IND #126926) and GSK2256098 (IND #126926); IND holder: Alliance

X	**Update:**		**Status Change:**
	Eligibility changes		Activation
	Therapy / Dose Modifications / Study Calendar changes		Closure
X	Informed Consent changes		Suspension / temporary closure
	Scientific / Statistical Considerations changes		Reactivation
	Data Submission / Forms changes		
	Editorial / Administrative changes		
X	Other: Vismodegib CAEPR update		

The changes included in this update to Alliance A071401 have been made in response to the NCI Action Letter from Dr. Naoko Takebe dated Month XX, 2017. This Action Letter is posted on the A071401 Study Page on the Alliance website. A revised CAEPR for vismodegib with new risks has been added to the protocol. Therefore, the model consent form has been revised to incorporate these new risks consistent with the NCI Model Consent Template instructions. There are no changes to the risk/benefit ratio.

Expedited review is allowed. IRB approval (or disapproval) is required within 90 days. Please follow your IRB of record guidelines.

UPDATES TO THE PROTOCOL

Section 3.1 On-Study Guidelines

Under the underlined heading "Serious or Life-threatening Birth Defect Effects of Vismodegib," the following changes have been made:

- At the end of the first paragraph, the following strikethrough text has been removed and underlined text added: , "…women of childbearing potential and men must agree to use two

methods of contraception (i.e., barrier contraception and another method of contraception) prior to study entry, for the duration of study participation, and for ~~12~~ 24 months following treatment (for women) and 2 months (for men)."

- In the first sentence of the fourth paragraph, beginning "Women of child-bearing potential…" the last phrase has been changed to require that two forms of contraception be used for at least 24 months post-treatment (previously, it was only required for at least 7 months post-treatment).
- In the same paragraph, a final sentence has been added which states: "Women should not breastfeed children for 24 months after the last dose of vismodegib."
- In the fifth paragraph beginning, "Vismodegib is present in semen," in the third and last sentences, text has been changed to state that male patients must use condoms and should not donate semen for 2 months after the final dose of vismodegib (previously, this was 3 months after the final dose).
- The seventh paragraph has been revised to state that patients should not donate blood or blood products during treatment and for 24 months after discontinuation of vismodegib (previously, this was during treatment and for 7 months after discontinuation).

Section 9.3 Expedited Adverse Event Reporting (CTEP-AERS)
In Section 9.3.1, throughout the bullets under "Expedited Adverse Event Reporting for Suspected Exposure to Agent that May Cause Serious or Life-threatening Birth Defects for Patients Receiving Vismodegib," language has been revised to require that adverse events related to pre-natal exposure, pregnancy, or abortions must be reported via CTEP-AERS if the event(s) occur within 24 months after the last dose of vismodegib (previously, this was 12 months after the last dose of vismodegib).

Section 9.4 Comprehensive Adverse Events and Potential Risks list (CAEPR) for GDC-0449 (Vismodegib, NSC 747691)
This section has been modified to include the updated Vismodegib CAEPR (Version 2.5, December 22, 2016) provided by CTEP. Changes from Version 2.4 to Version 2.5 have been outlined below:

- Added New Risk:
  - Rare but Serious: Musculoskeletal and connective tissue disorder - Other (premature epiphyseal closure)
- Increase in Risk Attribution:
  - Changed to Less Likely from Also Reported on GDC-0449 Trials But With Insufficient Evidence for Attribution: Abdominal pain; CPK increased; Dehydration

UPDATES TO THE MODEL CONSENT

What possible risks can I expect from taking part in this study?

- Under the bolded text, "Possible side effects of Vismodegib," the following changes have been made to the condensed risk profile based on the revisions to the Vismodegib CAEPR described above:
  - Added New Risk:
    - Rare: Bone growth may stop early in teenagers leading to short stature
  - Increase in Risk Attribution:
    - Changed to Common from Also Reported on GDC-0449 Trials But With Insufficient Evidence for Attribution (i.e., added to the Risk Profile): Dehydration
  - Provided Further Clarification:
    - Pain in Joints (previously under Occasional) is now reported as Pain (under Occasional).
- In the paragraph, under "If you are woman," the first and second sentence have been revised to state that women should not become pregnant or breastfeed for at least 24 months after completing

protocol treatment with vismodegib (previously, this was 7 and 6 months, respectively, after completing protocol treatment with vismodegib).

- In the paragraph, under "If you are a man," the first sentence has been revised to state that men should not father a baby for at least 2 months after completing protocol treatment with vismodegib (previously, this was 3 months after competing protocol treatment with vismodegib).
- The last paragraph has been revised to state that patients on vismodegib should not donate blood or blood products for 24 months after completing protocol treatment (previously, this was 7 months after completing protocol treatment with).

A replacement protocol document and model consent form have been issued

---

ATTACH TO THE FRONT OF EVERY COPY OF THIS PROTOCOL

ALLIANCE FOR CLINICAL TRIALS IN ONCOLOGY

PROTOCOL UPDATE TO ALLIANCE A071401

PHASE II TRIAL OF SMO/AKT/NF2 INHIBITORS IN PROGRESSIVE MENINGIOMAS WITH SMO/AKT/NF2 MUTATIONS

X	**Update**:	X	**Status Change**:
X	Eligibility changes		Activation
X	Therapy / Dose Modifications / Study Calendar changes		Closure
X	Informed Consent changes		Suspension / temporary closure
X	Scientific / Statistical Considerations changes	X	Reactivation
X	Data Submission / Forms changes		
X	Editorial / Administrative changes		
X	Other: Added new arm: AZD5363		

Expedited review is allowed. IRB approval (or disapproval) is required within 90 days. Please follow your IRB of record guidelines.

UPDATES TO THE PROTOCOL

- A new arm/cohort has been added for AKT1/PIK3CA/PTEN-mutated patients. These patients will go on to receive AZD5363. Therefore, updates have been made throughout the protocol to reference these mutations and include treatment information.

Cover Page

- In the agent information section under the study title, the agents "*AZD5363 (NSC #782347*)" has been added to reflect the addition of the new treatment arm. Additionally, the IND # has been removed after each of the agents as it is now listed just once at the end of the agent information.
- The title for Dr. Evanthia Galanis has been updated to "Study Co-Chair & Neuro-Oncology Committee Chair" (previously, "Study Co-Chair").
- The titles for Dr. Sandro Santagata, MD, PhD have been combined to "Neuropathology Co-Chair & Correlative Co-Chair."
- Darrel Borger, PhD has been removed as a Correlative Co-chair.
- Karla Ballman has replaced Qian Shi as the Primary Statistician.
- Sakuni Taniya Silva has replaced Samantha Sublett as the Protocol Coordinator.

Study Resources

- The document history table has been removed. This table will now appear as a separate document on the protocol landing page on the member side of the Alliance website.
- The contact information for "Questions regarding CTEP-AERS reporting" has been changed to the Alliance Pharmacovigilance Inbox from the Regulatory Affairs Manager.
- The contact information for "Questions regarding status of central pathology review and biomarker testing" has been changed to Sandro Santagata MD, PhD & John Iafrate, MD, PhD (previously Dr. Santagata and Darrel Borger, PhD).

Eligibility Summary and Schema

- The criteria listed in the eligibility summary have been revised to align with the changes made in Section 3.0. to include the new treatment arm.
- The ninth bullet under Registration Eligibility criteria has been updated for clarify that patients cannot have a craniotomy 28 days before or after registration. Previously, the statement read "no craniotomy within 28 days of registration."
- The "Required Initial Laboratory Values" have been removed from the schema as there are specific criteria based on the mutation and enrolling agent. A final bullet has been added under "Required Initial Laboratory Values" that reads: "Required initial laboratory values as indicated in Section 3.3.11."
- The schema has been completely revised to now include a cohort for patients with AKT1/PIK3CA/PTEN mutations who will receive AZD5363. The text for the previous NF2 cohort that went on to receive GSK2256098 has been greyed out. The phrase "prior to August 2017" has been added under "NF2" and the phrase "(closed to accrual July 2017)" has been added below "GSK2256098."

Section 1.2 (Genetic analysis of meningioma)

- In the sixth sentence of the first paragraph, the text "Notably, many of these mutations occur…" has been changed to "Notably, AKT1, SMO and PIK3CA pathway mutations occur…." for clarification.

Section 1.3 (Available Agents that Act on SMO, PTCH1, AKT1, and NF-2 Mutated Tumors)

- The first paragraph that begins, "Note: Afuresertib, the agent identified for patients with AKT1 mutation…" has been removed as it is no longer applicable to the agents used in this trial.
- In the now second paragraph that begins, "AKT is a serine/threonine…" sentences three through six of the paragraph that referenced afuresertib has been removed and replaced with language regarding AZD5363.

Section 1.6 (AZD5363)

- The section title has been changed from "Afuresertib" to "AZD5363," and all text in this section has been replaced with information regarding AZD5363.

Section 1.7 (Clinical Experience with AZD5363 as Monotherapy)

- In the section title, "AZD5363" has replaced "Afuresertib" for accuracy.
- The entire section has been replaced with information regarding clinical experience with AZD5363.

Section 1.12 (Impact of the Trial)

References to AZD5363 and AKT1/PIK3CA/PTEN mutations have been added. Additionally, reference to PTCH1 mutation has been added as it had been erroneously omitted.

Section 2.1 (Primary objectives)

- Objective 2.1.3 regarding the AKT1 inhibitor has been added.

Section 2.2 (Secondary objectives)
The entire section has been revised to include mention of AKT and CDK inhibitors.

Section 2.3 (Correlative science objectives)
- Objective 2.3.1 has been revised to indicate the purpose of evaluating the biomarkers.
- Objective 2.3.2 has been revised to mention the AKT and CDK inhibitors.

Section 3.1 (On-study guidelines)
- New second and third bullets have been added to provide guidance on medical conditions that may increase risk to the patients entering the protocol.
- A new subsection header "**Reproductive considerations, AZD5363**" and subsequent paragraph have been added prior to the "**Drug interactions**:" header.
- Under "**Drug Interactions**," the following changes have been made:
  - In the first paragraph, the following has been added as the final sentence, "The risk that AZD5363 will cause drug-drug interactions with substrates of P-gp is low."
  - A final sentence has been added to refer sites to Section 8.1 for other ancillary care and potential interactions of the study drugs.

Section 3.2 (Pre-registration eligibility criteria)
The title of Section 3.2.1 has been changed from "Central Pathology Review Submission" to "Tissue available for central pathology review and biomarker testing." Additionally, in the first sentence of the second paragraph, it has been clarified that the central pathology review and biomarker testing will be performed by MGH/DFCI.

Section 3.3 (Registration eligibility criteria)
- In Section 3.3.1 Documentation of disease, the following changes have been made:
  - In the paragraph for "Molecular documentation" references to AKT1, PIK3CA, PTEN mutations, CDKN2A, CDKN2A copy number loss, CDK4, CDK6, CCND1, CCND2, CCND3, or CCNE1 copy number gain have been added. Clarification has been added regarding central testing, regardless of whether local testing has been performed.
  - In the first bullet for "residual measurable disease," the interval of separation between the two scans showing progression has been extended from 12 months to 14 months. Additionally, the following sentence has been added: "For patients with SMO/PTCH1 mutations enrolling to receive vismodegib, the change can occur between scans separated by up to 25 months."
  - In the third bullet for "post radiation patients," a final sentence has been added that states: "If the progressive lesion is outside of the radiation field, then an interval of at least 2 weeks must have elapsed from completion of radiation to registration."
- In Section 3.3.3 Prior treatment,
  - In the third bullet, clarification has been added that no chemotherapy or investigation agents may have been administered within 28 days prior to registration, rather than start of treatment.
  - In the fourth bullet, clarification has been added that no case of nitrosourea or mitomycin C within 6 weeks prior to registration.
  - In the fifth bullet, the same clarification regarding the interval of time that must elapse (2 weeks) from completion of radiation for a progressive lesion outside of the radiation field has been added.

  - The eighth bullet has been updated to clarify that patients cannot have a craniotomy 28 days before or after registration. Previously, the statement read "no craniotomy within 28 days of registration."
- In Section 3.3.4, reference to Section 3.1 for agent-specific reproductive considerations has been added.
- In Section 3.3.5 it has been added that patients with AKT1/PIK3CA/PTEN mutation, CDKN2A copy number loss, or CDK4/CDK6/CCND1/CCND2/CCND3/CCNE1 copy number gain must be age ≥ 18 years to register.
- In Section 3.3.7 Patient history,
  - The second bullet has been modified to clarify that metastatic meningiomas are allowed if not outside of CNS and that spinal meningiomas are allowed.
  - The former seventh bullet has been removed regarding uncontrolled diabetes.
  - A new ninth bullet has been added that no major surgery may have been performed within 28 days prior to registration for patients receive AZD5363.
  - Two final sections have been added for consideration of patients with AZD5363 cardiac criteria, as well as miscellaneous criteria.
  - The final bullet beginning with "Non-leukocyte depleted whole blood transfusions…" has been removed because there is no optional pharmacogenetic evaluations in this study.
- The following underlined text has been added to the Section 3.3.8 titled, "Concomitant medications (Only regarding NF2/CDKN2A/CDK4/CDK6/CCND1/CCND2/CCND3/CCNE1/AKT1/PIK3CA/PTEN genetic alterations)."
  - Additionally, in the bullets that follow, references to patients with AKT1/PIK3CA/PTEN mutations enrolled to AZD5363 have been added.
  - In the first bullet, references to patients with NF2/CDKN2A/CDK4/CDK6/CCND1/CCND2/CCND3/CCNE1 genetic alterations has been added.
  - In the second bullet, CYP2D6 substrates has been added to prohibited concomitant treatments
- Section 3.3.9 Diabetic Status has been added. Subsequent subsections have been renumbered.
- In Section 3.3.10 Required Initial Laboratory Values,
  - The fourth lab value, Cal. Creatinine Clearance, has been changed from "> 45 mL/min" to "> 50 mL/min."
  - 'Sodium, Potassium, Magnesium, Total Calcium,' with a value of 'Within normal limits per institutional guidelines' has been added.
  - Fasting triglyceride and its value has been removed.
  - The value for QTcF has changed from ≤ 500 msec to < 450 msec.
  - Fasting cholesterol and its value have been removed.
  - 'Mean resting heart rate (determined from EKG),' with a value of 50-100 BPM has been added.
  - The footnotes have been revised to include ***, which clarifies the EKG requirement.

Section 4.0 (Patient Registration)

All text has been updated to align with current CTSU boilerplate language.

Section 4.3 (Patient Pre-Registration Requirements)

- The phrase "based on AKT1, PIK3CA, PTEN, NF2, SMO, PTCH1, CDKN2A, CDK4, CDK6, CCND1, CCND2, CCND3, CCNE1 gene status" has been added to the third sentence in the last paragraph in this section.

- In the third bullet, the first sentence now states that "Patients must have tissue available for analysis in order to be pre-registered for this study."

Section 4.4 (Patient registration procedures)

- In the first bullet, the sentence "If a sample fails testing due to poor tissue (nucleic acid) quality, a request for a specimen from an alternative surgery will be made" has been added as the third sentence.
- In the fourth bullet, the phrase "28 days" has been replaced "14 days" in the first sentence to note the time allowed between receiving notification of patient eligibility and registration. Additionally, a new second sentence has been added that reads: "Please keep in mind that spots cannot be held or saved on cohorts, patient registration is on a first-come basis."

Section 4.5 (Patient Registration/Randomization Procedure)

The section title has been updated from **"Patient Enrollment through OPEN"** to "**Patient Registration/Randomization Procedures**."

Section 4.6.1 (Registration to Substudies described in Section 14.0)

- The first bullet has been updated to read "Identification of molecular and histological biomarkers of response, Alliance A071401-ST1."
- A final paragraph has been added regarding enrollment and imaging submission for the imaging substudy.

Section 4.7 (Treatment assignments and patient cohorts)

- In the first paragraph, references to AKT1, PIK3CA, PTEN, NF2, SMO, CDKN2A, CDK4, CDK6, CCND1, CCND2, CCND3, CCNE1 genetic alterations, and AZD5363 have been added.
- In the first sentence of the second paragraph, "SMO is least common" has been added in the parenthesis.
- The groups have been bulleted for readability.
- In Group 1 and Group 2 for NF2 mutations, the parenthetical text "Closed to new patient enrollment on 7/19/2017" has been added.
- The corresponding study drugs have been added to the group names.
- Group 5 (AKT1/PIK3CA/PTEN mutation, Grade I – AZD5363) and Group 6 (AKT/PIK3CA/PTEN mutation, Grade II/III – AZD5363) have been added.

Section 5.0 (Study Calendar)

The study calendar footnotes have been revised and re-characterized for readability and to reflect the Alliance standard for study calendars. Specifically, the following changes have been made:

- In the row for "Adverse Event Assessment," footnote Ω has been removed from the first column and changed to footnote 1, which has been referenced in each column/timepoint for which the assessment is required. Subsequent footnotes has been renumbered accordingly.
- In the row for "Patient Medication Diary," footnote Φ has been removed from the first column and changed to footnote 2, which has been referenced in each column/timepoint for which the diary is required. Subsequent footnotes has been renumbered accordingly.
- In the row for "Registration Fatigue/Uniscale Assessment," footnote # has been removed from the first column and changed to footnote 3, which has been referenced in the prior to registration column. Subsequent footnotes has been renumbered accordingly.
- A new row for "ECHO/MUGA" has been added, and letter "A" added in the "Prior to Registration" and "Day 1 of each cycle…" columns. Below the table, footnote A has been added, it reads: "Required only for patients with NF2/CDKN2A/CDK4/CDK6/CCND1/CCND2/CCND3/CCNE1 genetic alterations

enrolling/enrolled on AZD5363. For patients on AZD5363, required prior to registration, on cycle 4, and at discontinuation, withdrawal or removal." Subsequent footnotes have been re-lettered accordingly.

- In the row for "EKG," the text in the second and third columns has been changed from "X($)" to "B." Therefore, footnote $ now appears as footnote B. Footnote B has also been clarified to state that the EKG must be performed within 28 days prior to registration. Subsequent footnotes have been re-lettered accordingly.
- In the row for "Chemistry (Creatinine, AST, ALT, Alk. Phos., Bili, glucose)," a reference to footnote 4 has been added in the "Prior to registration" column. Below the table, footnote 4 has been added.
- In the row for "Urine Protein," footnote ! has been changed to footnote 5. Subsequent footnotes have been renumbered accordingly.
- In the row for "Fasting cholesterol, triglycerides," the text in the second and third columns has been changed from "X(%)" to "C." Therefore, footnote % now appears as footnote C. Subsequent footnotes have been re-lettered accordingly. Additionally, the following text has been added as the second sentence: "Required every 12 weeks for patients with AKT1/PIK3CA/PTEN mutations enrolled receiving AZD5363."
- The row, "Electrolytes (Na, K, Mg, Ca [corrected for serum albumin], P)," has been added.
- In the row for "MRI/CT Brain," reference to footnote "(3)" in the first column has been removed. Additionally, former footnotes 3 and 4 have been combined and now appear as footnote 8. A reference to footnote 8 has also been added to the last column in the row.
- In the row for "Tissue and Blood samples," the second and third sentences have been modified to require blood and tissue samples at recurrence/progression.
- Footnote 6 (formerly footnote 1) has been revised to clarify that serum or urine HCG must be performed with 7 days prior to registration for all women of childbearing potential, along with additional requirements for Cycle 1, Day 1 for patients receiving AZD5363, and at treatment discontinuation.
- Footnote B has been revised to state that EKG is "required", formally "as clinically indicated."
- Footnote D (formerly footnote A) has been revised to remove the brain MRI/CT at the 'every 12 weeks until evidence of progression' time point.

Section 6.1 (Data Collection and Submission)

The first paragraph has been updated with new CTSU template language text.

Section 6.2 (Specimen collection and submission)

- Under "For all patients registered to Alliance A071401," the following changes have been made:
    - In the first paragraph the diagnostic specimen submission requirement has changed from FFPE tumor biopsy or surgical tissue sample to tissue from a single representative tumor tissue block, along with a pathology report for the specimen submitted.
    - In the second paragraph, references to genetic alterations in SMO, PTCH1, NF2, AKT1, PIK3CA, PTEN, CDKN2A, CDK4, CDK6, CCND1, CCND2, CCND3, CCNE1 have been added.
    - In the second paragraph, Dr. John Iafrate's laboratory has replaced Dr. Darrel Borger's laboratory as the location where integral molecular testing will take place.
    - The word "complete" has been added to third paragraph to clarify that results will be returned within 21 days of complete specimen receipt.
- The following changes have been made to the table:
    - The column header "After initial biopsy/surgery and at recurrence" has been changed to "≤ 90 days after registration."

- Certain text has been abbreviated for readability (i.e., Massachusetts General Hospital now appears as MGH and treatment as tx).
- A new column has been added for "Recurrence/Progression."
- Under "For patients registered to A071401-ST1…," in the first column, "recurrent tumor" has been changed to "recurrent/progressive tumor." An X has also been added in the "Recurrence/Progression" column to indicate that these specimens should be submitted.
- In the same row, a reference to footnote 3 has been added. Footnote 3 has been added below the table, it reads: "New biopsy is not required, only submit if surgery is performed."
- In the row for "Whole blood for ctDNA plasma," 2 x 10 mL of blood are now to be submitted prior to registration, every 4 cycles during treatment, and at recurrence/progression for patients who consent to A071401-ST1. Additionally, the shipping conditions have been changed from "Dry Ice/ship overnight" to "Dry Ice/ship overnight or freeze." Footnote *** has been added to the every 4 cycles during treatment time point for ctDNA submission, specifying that for patients receiving AZD5363, whole blood for ctDNA plasma is to be submitted pre-dose on Cycle 1 Day 1, and pre-dose Cycle 2 Day 1.
- In the row for "Whole Blood" and "Whole blood for ctDNA plasma," references to footnote 4 have been added. Below the table, footnote 4 has been added, it reads: "Whole blood and whole blood for ctDNA plasma must be shipped SEPARATELY due to shipping conditions (cool pack vs. frozen/dry ice). See Section 6.2.4 for instructions."

– In Section 6.2.1 Specimen submission using the Alliance Biospecimen Management System, under "ALL tumor tissue for Central Pathology Review and Integral Biomarker Testing," the contact for shipment location and Central Pathology review has been updated.

– In Section 6.2.2 Mandatory diagnostic tumor tissue sample and H&E slide submission for central laboratory tumor genotyping and central pathology review
  - A new first paragraph has been added to elaborate on the integral molecular testing that will determine eligibility for this trial.
  - Submission instructions have been numbered for clarity.
  - A bolded statement has been added under bullet 7 regarding the start of the 21 day turnaround time for testing.

– In the title of Section 6.2.3 Recurrent/progression tissue submission to Alliance Biorepository at Mayo FFPE (A071401-ST1), the word "Progression" has been added. Additionally, the first paragraph has been revised and text added to clarify that upon enrollment, if patient has recurrent disease then tissue samples should be submitted. Additionally, language regarding submission of progression samples has been added, and it has been clarified that the samples are only required if surgery is performed. New biopsies/surgery is not required.

– In Section 6.2.4 Blood sample submission (A071401-ST1), the following changes have been made:
  - A new second paragraph has been added to instruct sites that whole blood for germline DNA and whole blood for ctDNA must be shipped separately due to different required shipping conditions.
  - In the first bullet under "Whole blood for germline DNA," a new final sentence has been added, it reads: "Batch shipping is not allowed, please indicate on the packing slip whether the frozen samples will be sent right away, or shipped at a later date."
  - In the first bullet under "Whole blood for circulating tumor DNA (ctDNA) plasma," the first sentence has been modified to require submission of the specimen at recurrence/progression.
  - In the third bullet under "Whole blood for circulating tumor DNA (ctDNA) plasma," text "(or lab standard)" has been added after every mention of centrifuging at 1500g.
  - In the bullet beginning "Freeze sample at -80 °C…," the second sentence has been clarified to state that the blood should be shipped on dry ice within 30 days of the blood

draw. Additionally, the last sentence has been turned into a bullet. And a new bullet has been added to provide instructions for batch shipping.

- In the last bullet beginning "Plasma samples…," in bullet #5, the text "(be sure to label sample with 'PPP')" has been added.

Section 6.3 (CT and MRI Imaging Data Submission)

- The "TRIAD Access Requirements" has been updated using the CTSU language template.

Section 7.0 (Treatment Plan/Intervention)

- The header "Treatment will be administered as follows," has been added before the third paragraph. The third paragraph has been bulleted and completely revised to include description of treatment with AZD5363.
- In the fourth paragraph, "For all arms" has been added to the beginning of the first sentence. Additionally, third and fourth sentences have been added to refer sites to Sections 5, 8, 11, and 12.
- The header "Arm A (SMO/PTCH1 mutation)" has been changed to "Arm A (SMO/PTCH1 mutation – Vismodegib)" and now appears as Section 7.1.
- The header "Arm B (NF2 mutation)" has been changed to "Arm B (NF2 mutation - GSK2256098)" and now appears as Section 7.2.

Section 7.2 (Arm B (NF2 mutation – GSK2256098))

- The second paragraph has been revised to state that all supply of GSK2256098 for the study expires by September 2019 (previously, this was January 2018).
- The paragraph that previously followed, which began "Contrast enhanced cranial magnetic resonance imaging (MRI) will be performed…" has been removed as an overview of where to find this information can be found in Section 7.0.

Section 7.3 (Arm C (AKT1/PIK3CA/PTEN mutation – AZD5363))

This section has been added to provide treatment instructions.

Section 7.4 (Important interaction information for patients receiving GSK2256098 or AZD5363)

- This section header has been added. Section 7.1 now appears as Section 7.4.1 CYP3A4 inhibitors, and Section 7.2 now appears as Section 7.4.2 CYP3A4 inducers. These sections have both been clarified to state that strong inhibitors and inducers of CYP3A4 are not allowed for patients receiving GSK2256098 or AZD5363.
- Section 7.5.3 CYP2D6 Inhibitors has been added.

Section 8.1 (Ancillary therapy, concomitant medications, and supportive care)

- In Section 8.1.7 Rash, specific instructions for AZD5363 have been added.
- Section 8.1.9 Hyperglycemia, has been added, and subsequent subsections have been renumbered.
- In Section 8.1.14 Reproductive considerations, two sentences have been added to provide pregnancy prevention instructions for AZD5363.
- In Section 8.1.16 QT Prolongation, a header has been added that states "For patients receiving GSK2256098."
- Section 8.1.17 Use of metformin has been added to provide guidance for management of patients receiving both metformin and AZD5363.

Section 8.3 (GSK2256098 Dose modifications)

The last sentence has been bulleted, and a new first sentence has been added to the bullet to clarify that descriptors in the subsections below use CTCAE version 4.0.

Section 8.4 (AZD5363 dose modifications)
This section has been added to provide dose modifications for AZD5363.

Section 9.0 (Adverse Events)
The sentence "However, The descriptions and grading scales found in the NCI Common Terminology Criteria for Adverse Events (CTCAE) version 5.0 will be utilized for expedited AE reporting beginning April 1, 2018" has been added as the third sentence.

Section 9.1 (Routine adverse event reporting)
- Nausea, Vomiting, Anaphylaxis, Dry skin, Pruritus, Stomal ulcer, and Neutrophil count decreased have been added to the routine AE reporting table, along with each CTCAE SOC category.

Section 9.3 (Expedited Adverse Event Reporting)
- CTCAE has been updated to version 5.
- New language regarding pregnancy loss, neonatal death, and deaths on study has been added under "Additional instructions…"
- The following language has been removed from Section 9.3.1:
  - "**NOTE:** Protocol specific exceptions to expedited reporting of serious adverse events are found in the Specific Protocol Exceptions to Expedited Reporting (SPEER) portion of the CAEPR"
  - In the sixth bullet under "**Additional Instructions or Exclusion to CTEP-AERS Expedited Reporting Requirements for Phase 1 or Early Phase 2 Trials Utilizing an Agent Under a non-CTEP IND**," the second sentence about reporting new primary malignancies on the Notice of New Primary Form has been removed.
  - The former ninth bullet has been replaced with the new heading "Pregnancy loss."

Section 9.4 (Comprehensive Adverse Events and Potential Risks list [CAEPR] for GDC-0449 [Vismodegib, NSC 747691])
The SPEER column has been removed from the CAEPR for vismodegib. SPEER related language in the first paragraph (sentences 2 and 3) and the entire second paragraph have been removed.

Section 9.5 (Comprehensive Adverse Events and Potential Risks list [CAEPR] for AZD5363 [NSC 782347])
The CAEPR for AZD5363 has been added with a SPEER column omitted.

Section 10.0 Drug Information
In Section 10.1 through Section 10.3, under 'procurement,' the distributor has been updated from Biologics Inc. to McKesson Specialty Pharmacy.

Section 10.2 (GSK2256098 (NSC# 783781, IND #126926))
Under "Procurement," the second paragraph has been modified to state that supplies of GSK2256098 for this trial may expire as late as September 2019 (previously, supplies had an expiry of January 2018 at the latest).

Section 10.3 (AZD5363 (NSC # 782347, IND #126926))
This section has been added to provide drug information regarding AZD5363.

Section 11.4.3.3 (Evaluation of Non-Measurable Lesions)

Under Progression (PD), the NOTE has been corrected to "Equivocal progression of a nonmeasurable lesion should not normally trump change in a measurable lesion. It must be representative of overall disease status change." Previously, this NOTE began with "Unequivocal progression…"

Section 13.1 (Study Design)

- The first paragraph has been modified to reflect the addition of the AKT1/PIK3CA/PTEN cohorts and the new CDK inhibitor cohort for NF2-mutated patients.
- The last paragraph has been modified to list the agents associated with each of the mutations.

Section 13.2 (Statistical design and analysis for primary endpoint)

- In Section 13.2.1 Primary endpoints, a new final sentence has been added to the end of the second paragraph, "In the event of re-registration of patients from one mutation arm to another, additional sensitivity analyses may be performed."
- In Section 13.2.2 Statistical design, the following changes have been made:
    - Headers for each of the mutations and arms have been updated to include the corresponding study agent (i.e. "SMO/PTCH1 mutation defined arm" has been changed to "SMO/PTCH1 mutation – Vismodegib arm"). This change has been made throughout the protocol document.
    - Under the header, "NF2 mutation – GSK2256098 arm," the former final paragraph has been removed (formerly starting with "Overall power and family wise alpha consideration…Thus the trial has 8% to 94% power…").
    - A new underlined header titled "AKT1/PIK3CA/PTEN mutation – AZD5363 arm" has been added along with four paragraphs.
    - A new under lined header titled "Overall power and family-wise alpha consideration" has been added along with one paragraph.
- In Section 13.2.3 Analysis Plan,
    - In the first paragraph, reference to a 6 month PFS endpoint and RR endpoint.
    - Under the description of the SMO/PTCH1 mutation analysis, in the first bullet, the number of responses to be observed before considering the agent active has changed from 3 to 4 or more responses.

Section 13.3 (Sample size, accrual time and study duration)

Section 13.3.1 Sample size and Section 13.3.2 Accrual rate and accrual duration have been revised to reflect the addition of the AKT1/PIK3CA/PTEN mutation – AZD5363 cohort. Specifically, the accrual has been changed from 60 to 84 patients as 24 evaluable patients are required for AKT1/PIK3CA/PTEN mutation – AZD5363 cohort. Additionally, the maximum target accrual has been changed from 69 to 124 patients.

Section 13.4 (Supplementary Analysis Plan)

- In the first paragraph, the following underlined text has been added for clarification: "Overall survival and progression free-survival will be summarized for each cohort and mutation, separately, within each treatment group with Kaplan-Meier curves and estimates. No formal comparison will be made among the cohorts or mutations of the treatment groups."
- In the second paragraph, histology grade subgroups has been added regarding AE summaries.
- In the final paragraph, the two-sided confidence interval has changed from ninety to ninety-five percent.

Section 13.8 (Inclusion of women and minorities)
The table has been updated to include the patients for the AKT1/PIK3CA/PTEN mutation – AZD5363 cohort.

Section 14.1 (Correlative studies using Biospecimens (Alliance A071401-ST1))
- In Section 14.1.1.1 Background, reference to CDK inhibitor and AKT/PIK3CA/PTEN-mutated meningiomas has been added.
- In Section 14.1.1.2 Objectives, references to PTCH1, PIK3CA and PTEN mutations, as well as AKT and CDK inhibition have been added. Additionally, mentions of using tumor samples from progression have been added. In the final paragraph, a sentence regarding analysis for immunohistochemical markers to evaluate pathway activation has been added.

Appendix II (Required Consensus MRI Acquisition Parameters)
The first paragraph, beginning with "For sites that do NOT have…" has been removed.

Appendix IV (Patient Medication Diaries)
- For document organization purposes, "Appendix IV Patient Medication Diary – vismodegib" and "Appendix V Patient Medication Diary – GSK2256098" have been renamed to "Appendix IV-A Patient medication diary - vismodegib" and "Appendix IV-B Patient Medication Diary – GSK2256098," respectively. Therefore, Appendix IV has been retitled "Patient Medication Diaries." Subsequent appendices have been numbered appropriately.
- Appendix IV-C Patient medication diary – AZD5363 has been added.

Appendix V (Concomitant Medications Prohibited or for use with Caution with AZ5363)
This appendix has been added.

Appendix VII (Central Laboratory Genotype Testing Procedures)
- Throughout the appendix, references to AKT1, PIK3CA, PTEN, CDKN2A, CDK4, CDK6, CCND1, CCND2, CCND3, and CCNE1 have been added.
- In the last sentence of the first paragraph, the number of genes targeted by the SNAPSHOT NGS assay has been updated from 91 to 106.
- Under Methodology, the paragraphs "Nucleic Acid Extraction," "Gene Variant Testing," and "Bioinformatic Variant Analysis" have been added.
- The entire paragraph under "Target Mutations" has been completely revised and now includes a table with references to NF2, SMO, PTH1, AKT1, PIK3CA, PTEN, CDKN2A, CDK4, CDK6, CCND1, CCND2, CCND3, and CCNE1.
- Under "Assay Protocol,"
    - The first bullet has been added regarding SNAPSHOT assay being performed on genetic alterations in SMO, PTCH1, NF2, AKT1, PIK3CA, PTEN, CDKN2A, CDK4, CDK6, CCND1, CCND2, CCND3, and CCNE1.
    - A new bullet #6 has been added to indicate that samples will be sequenced on Illumina MiSeq or NextSeq genome sequencer.
    - A new bullet #7 has been added to explain how sequencing data will be processed.
    - In bullet #9, the phrase "for SMO, PTCH1, PTCH1, NF2, AKT1, PIK3CA, or PTEN was" has been removed.

Appendix VIII (Patient Drug Information Handouts and Wallet Cards)
- The Appendix title has been pluralized.
- The patient drug information handout and wallet card for patients receiving GSK2256098 is now titled Appendix VIII-A.

- Appendix VIII-B has been added for patients receiving AZD5363.

UPDATES TO THE MODEL CONSENT

- The document title "Research Study Informed Consent Document" has been added.
- The study number has been added at the beginning of the official study title.
- Under the study title, a new paragraph has been added to explain that the study sponsor is the Alliance for Clinical Trials in Oncology and National Cancer Institute.
- Throughout the consent, references to AKT, PIK3CA and PTEN mutations have been added.

Why is this study being done?

- The first paragraph has been revised to reflect the addition of the new arm for AZD5363.
- A new third sentence has been added to the second paragraph, which reads: "Vismodegib has not been FDA approved for meningioma."
- A header has been added above the third full paragraph that reads: "The group described below closed to new patient participation in July of 2017:" The paragraph has been highlighted in grey to visibly show that it is closed.
- A fourth paragraph has been added to explain that patients with AKT1, PIK3CA or PTEN mutations will receive AZD5363.
- A fourth paragraph has been added to explain that patients with AKT1, PIK3CA or PTEN mutations will receive AZD5363.
- The fifth paragraph has been revised to state that 84 people will take part in the study (Vismodegib: 24 people, GSK2256098: 36 people, and AZD5363: 24 people).

What are the study groups?

- The second sentence of the first paragraph has been changed from, "If you have the SMO, PTCH1 or NF2 mutation…" to "If you have a certain type of mutation…."
- A header has been added above the third full paragraph that reads: "The group described below closed to new patient participation in July of 2017:" The paragraph has been highlighted in grey to visibly show that it is closed.
- A new fourth paragraph has been added to provide an overview of the treatment of AZD5363 for patients with AKT1, PIK3CA or PTEN mutations.
- The first sentence of the seventh paragraph has been revised to list out the name of the study groups as opposed to mutations (i.e. changed from "SMO/PTCH1 mutation" to "SMO/PTCH1 – Vismodegib").
- In the diagram, the following changes have been made:
  - Reference to AKT, PIK3CA, and PTEN mutations has been made as follows: "Your tumor is tested for mutated genes (including SMO, PTCH1, NF2, AKT, PIK3CA, and PTEN)."
  - A note has been added to Group 2 that states, "Closed as of 7/2017." The Group has also been highlighted in grey to visibly show that the group is closed.
  - Groups 3 has been added to the schema.
  - The following underlined text has been added, "Your tumor does not have any of these genes or the group you are eligible for is no longer open to new patients."

What extra tests and procedures will I have if I take part in this study?

- The risks of the biopsy have been added to Page 8.
- Under "You will need to have the following extra tests to find out if you can be the study," the following changes have been made:
  - In the first bullet, a second sentence has been added to state that fasting glucose will be required for patients going on to receive AZD5363.

- In the second to last bullet, it has been clarified that EKG will be required for patients with NF2 mutation going on to receive GSK2256098.

- Under "During the study," the following changes have been made:
    - In the second bullet, it has been clarified that EKG will be required for patients with NF2 mutation going on to receive GSK2256098.

<u>What possible risks can I expect from taking part in this study?</u>

- The sixth paragraph has been modified to state that patients may (previously, "will") receive a handout and wallet card. Additionally, references to AZD5363 have been added as handouts and wallet cards have been made available in the protocol appendix for these patients.
- The following text has been added to the header for Possible Side Effects of Vismodegib, "(for patients with SMO or PTCH1 mutations)."
    - "Weight loss" has been replaced with "Anorexia (weight loss)" in the Common, Some May Be Serious table.
    - Joint pain has been added to the Occasional, Some May Be Serious table.
- The following text has been added to the header for Possible Side Effects of GSK2256098, "(for patients with NF2 mutations enrolled before July of 2017)."
- A new header "Possible Side Effects of AZD5363 (for patients with AKT1, PIK3CA or PTEN mutations)" and a corresponding condensed risk list have been added.
- The paragraphs regarding reproductive risks have been revised as follows:
    - In the first paragraph that begins "Reproductive risks," a third sentence has been added to stipulate that patients will need to use two methods of birth control.
    - Three final sentences have been added to the end of the first paragraph which state: "Some methods might not be approved for use in this study. You should not breastfeed a baby while on this study, and for at least 6 months after completing protocol treatment. For more information about risks and side effects, ask your study doctor."
    - The headers and corresponding paragraphs for "If you are woman" and "If you are man" have been removed and replaced with completely new paragraphs regarding reproductive risk information organized by agent and then by gender.

<u>What are the costs of taking part in this study?</u>

- References to AZD5363 has been added to the first and second paragraphs.
- The third paragraph has been revised to state that GSK2256098 will be available until September 2019 (previously, agent was only available through January 2018).

<u>Who will see my medical information?</u>

- This entire section has been updated with current privacy language.
- In the first paragraph, the first sentence has been changed from There are organizations that may inspect your records" to "There are some organizations that may inspect, share, and disclose your records."
- Under the second paragraph, the first bullet has been changed from "The study sponsor and any drug company…" to "The Alliance and any drug company supporting the study and their authorized agents".
- A second bullet has been added to list the labs at Dana-Farber Cancer Institute and Massachusetts General Hospital that are performing central pathology review and biomarker testing.

<u>Optional Sample Collections for Laboratory Studies and/or Biobanking for Possible Future Studies</u>

Under "<u>What is involved?</u>" in bullet #1, text has been added to inform patients that if they consent, then a blood sample will also be collected and submitted if their meningioma gets worse or comes back.

A replacement protocol document and model consent form have been issued

---

ATTACH TO THE FRONT OF EVERY COPY OF THIS PROTOCOL

Update #09

ALLIANCE FOR CLINICAL TRIALS IN ONCOLOGY

PROTOCOL UPDATE TO ALLIANCE A071401

PHASE II TRIAL OF SMO/AKT/NF2 INHIBITORS IN PROGRESSIVE MENINGIOMAS WITH SMO/AKT/NF2/CDKx/CCNx MUTATIONS

[X] **Update**:	[X] **Status Change**:
[X] Eligibility changes	[ ] Activation
[X] Therapy / Dose Modifications / Study Calendar changes	[ ] Closure
[X] Informed Consent changes	[ ] Suspension / temporary closure
[X] Scientific / Statistical Considerations changes	[X] Reactivation
[ ] Data Submission / Forms changes	
[X] Editorial / Administrative changes	
[ ] Other:	

If your site utilizes the CIRB as your IRB of record
No recommended IRB level of review is provided by the Alliance since the CIRB is the IRB of record for this trial. The site has 30 days after the posting of this amendment to implement it at their site. Please refer to the amendment application and CIRB guidelines for further instructions.

If your site utilizes a local IRB as your IRB of record
Expedited IRB approval is allowed. The proposed changes in this amendment are minor and do not affect the overall risk/benefit ratio. IRB approval (or disapproval) is required within 90 days. Please follow your local IRB guidelines.

UPDATES TO THE PROTOCOL

Cover Page

- The title of the study has been updated to include CDKx and CCNx mutations.
- The phrase *"Abemaciclib (NSC #783671)"* has been added to the list of *"Industry-supplied agents."*

Cancer Trials Support Unit (CTSU) Address and Contact Information

All text included in the table has been revised with updated CTSU language.

Schema

- The vismodegib box has been grayed out to indicate that the arm is closed. Additionally, the date accrual to the vismodegib arm was closed has been added below the drug name.
- Two new boxes have been added to include the new mutational group and treatment regimen added with this update. They read:"NF2, CDKN2A, CDK4, CDK6, CCND1, CCND2, CCND3, or CCNE1 (As of Update #09)" and "Abemaciclib 200 mg PO BID."

Table of Contents

The table of contents has been updated.

Section 1.2 (Genetic analysis of meningioma)

New seventh and eighth sentences have been added to the first paragraph, which read: "Furthermore, high grade and progressive meningiomas have loss of CDKN2A, which is part of the CDK pathway and many of these CDKN2A alterations co-occur in NF2 mutated meningiomas. NF2 loss and CDKN2A loss have been demonstrated to promote meningioma progression in preclinical models." Four new references (#18-21) have been added as a result of this new language, and all subsequent references have been renumbered throughout the text and in the references list in Section 16.0.

Section 1.3 (Available Agents that Act on SMO, PTCH1, AKT1, and NF-2 Mutated Tumors)

A new fifth paragraph has been added to provide information regarding abemaciclib. The new paragraph begins "Abemaciclib is a selective and potent small molecule CDK4 and CDK6 dual…"

Section 1.10 (Abemaciclib)

A new section has been added to provide background information on abemaciclib. Subsequent sections have been renumbered accordingly.

Section 1.11 (Clinical Experience with Abemaciclib)

A new section has been added to describe the clinical experience with abemaciclib. Subsequent sections have been renumbered accordingly.

Section 1.14 (Impact of the Trial)

The underlined language has been added to the first sentence: "Based on the biomarker work, we have designed a phase 2 study of vismodegib (SMO inhibitor), AZD5363 (AKT inhibitor), GSK2256098 (a FAK inhibitor), or abemaciclib (CDK inhibitor) in patients with recurrent or progressive meningiomas harboring genetic alterations in SMO/PTCH1, AKT1/PIK3CA/PTEN, NF2, or the CDK pathway (CDK4/CDK6/CCND1/CCND2/CCND3/CCNE1/CDKN2A), respectively."

Section 2.1 (Primary Objectives)

A new primary objective for the CDK inhibitor arm has been added as Section 2.1.4.

Section 3.1 (On-study guidelines)

The reproductive considerations for abemaciclib have been added as a new fourth subsection under the "In addition:" heading.

Section 3.3.1 (Documentation of Disease)

In the third bullet of the third criterion titled "**Post radiation patients**," the underlined language has been added to the second sentence "If the progressive meningioma lesion has been radiated, at least 24…"

Section 3.3.3 (Prior Treatment)

The fifth bullet has been updated to state that "For patients treated with external beam radiation, interstitial brachytherapy or radiosurgery, an interval > 4 weeks must have elapsed from completion of radiation treatment to registration." Previously, this was an interval greater than 2 weeks.

Section 3.3.8 (Concomitant medications [Only regarding NF2/CDKN2A/CDK4/CDK6/CCND1/CCND2/CCND3/CCNE1/AKT1/PIK3CA/PTEN genetic alterations])
A new third bullet has been added to address concomitant use of CYP3A inducers and inhibitors for patients enrolled to the abemaciclib arm.

Section 3.3.10 (Required Initial Laboratory Values)
The third footnote (***) has been revised to indicate that triplicate EKG is required if the patient is assigned to the AZD5363 arm. Patients on all other arms require a single EKG. Previously, a single EKG was required for all arms.

Section 3.3.11 (Comorbid Conditions)
A new section has been added to state that patients cannot enroll if they have uncontrolled medical comorbidities.

Section 3.4 (Additional Registration Eligibility Criteria for Abemaciclib Arm)
A new section has been added to define additional eligibility criteria for patients who are assigned to the abemaciclib arm.

Section 4.0 (Patient Registration)
This entire section has been revised with updated CTSU language.

Section 4.7 (Treatment Assignments and Patient Cohort)

- The word "abemaciclib" has been added to the list of agents in the second sentence of the first paragraph.
- A new seventh bullet has been added under the third paragraph that reads: "Group 7: NF2 mutation/CDKN2A copy number loss/CDK4, CDK6, CCND1, CCND2, CCND3, or CCNE1 copy number gain – abemaciclib."

Section 5.0 (Study Calendar)
The entire study calendar has been updated to include the abemaciclib arm. Specifically, the following changes have been made:

- In Footnote 6, the phrase "and ≤ 7 days prior to initiation of abemaciclib for patients with *NF2* mutation" has been added to the second sentence to indicate that patients need to take a pregnancy test within 7 days prior to beginning treatment on abemaciclib.
- Footnote 9 has been added to provide direction for patients on the abemaciclib arm. Footnote 9 has been added under "Day 1 of each cycle (cycle is 28 days)" for "Complete blood count, differential, platelets" and under "Day 1 of each cycle (cycle is 28 days)" for "Chemistry (Creatinine, AST, ALT, Alk. Phos., Bili, glucose) HbA1c (if required)."
- Footnote B has been revised to provide direction for patients on the AZD5363 and abemaciclib arm. Footnote B has been added under "Post Treatment Follow Up" for "EKG."
- Footnote E has been added to indicate that these items on the study calendar are only required of patients enrolled in the abemaciclib arm. Footnote E has been added under "Post treatment follow up" for "Complete Blood Count, Differential, Platelets" and "Chemistry (Creatinine, AST, ALT, Alk. Phos., Bili, glucose) HbA1c (if required)."

Section 6.1 (Data Collection and Submission)

- This entire section has been revised with updated CTSU language.
- A new Section 6.1.2, regarding the Data Quality Portal, has been added.

Section 6.3 (CT and MR Imaging Data Submission)

The language under **"1) TRIAD based data transfer"** has been revised with updated CTSU language.

Section 7.0 (Treatment Plan/Intervention)

A new fourth bullet has been added below the third paragraph entitled "Treatment will be administered as follows." The new bullet reads: "In patients harboring alterations in the CDK pathway or in NF2 (after Update #9), a CDK inhibitor (abemaciclib) will be administered."

Section 7.3 (Arc C [AKT1/PIK3CA/PTEN mutation – AZD6363])

The frequency of the dosing in the table has been corrected from "…repeated every cycle" to "…repeated weekly."

Section 7.4 (Arm D [CDK4, CDK6, CDKN2A, CCND1, CCND2, CCND3, CCNE1 alterations-Abemaciclib])

This section has been added to provide instructions for patients enrolled to the abemaciclib arm. Subsequent sections have been renumbered.

Section 7.5 (Important interaction information for patients receiving GSK2256098, AZD5363, and abemaciclib)

- The title of this section has been revised to add abemaciclib.
- Section 7.5.1
  - Abemaciclib has been added to the list of agents in the first and second sentence.
  - A new third paragraph has been added to provide direction if co-administration of abemaciclib with a strong CYP3A inhibitor is unavoidable.
- Section 7.5.2
  - Abemaciclib has been added to the list of agents in the first and second sentence.

Section 8.1.5 (Diarrhea)

Information regarding diarrhea management for patients enrolled to the abemaciclib arm has been added as the third paragraph.

Section 8.1.14 (Reproductive Considerations)

Directions on pregnancy prevention for patients enrolled to the abemaciclib arm has been added as the fourth sentence of the paragraph.

Section 8.1.15 (Liver Toxicity)

- The section has been retitled to Liver Toxicity, previously Hepatitis.
- A final sentence has been added that reads: "For abemaciclib specific hepatic toxicity, please see Section 8.5.5."
- Two new third and fourth paragraphs and a table regarding hepatic monitoring tests has been added to direct sites on how to monitor patients with hepatic abnormalities.

Section 8.4.1 (Dose Levels)

The dose levels have been modified so that the first dose reduction will be 320mg and the second dose reduction will be 200mg.

Section 8.5 (Abemaciclib Dose Modifications)
This entire section has been added to provide information about abemaciclib dose modifications.

Section 9.3.1 (Phase I and Early Phase 2 Studies)

In the fifth bullet under the section titled "**Expedited Adverse Event Reporting for Suspected Exposure to Agent that May Cause Serious or Life-threatening Birth Defects for Patients Receiving Vismodegib,"** AZD5363 has been replaced with GDC-0449.

Section 9.5 (Comprehensive Adverse Events and Potential Risks list [CAEPR] for AZD5363 [NSC 782347])

The CAEPR for AZD5363 has been updated.

Section 10.3 (AZD5363 [NSC # 782347, IND # 126926])

- Under *Formulation*, the AZD5363 supplied dose has been updated to 160 (previously 80) or 200mg tablets. Both doses will be available in 60 (previously 30) tablet or 76 (previously 60) tablet bottles.
- Directions for obtaining the investigator brochure for AZD5363 has been added to the end of the section.

Section 10.4 (Abemaciclib [LY2835219, VERENZIO™, NSC#783671])
This entire section has been added to provide drug information regarding abemaciclib.

Section 13.1 (Study Design)

- In the first sentence of the first paragraph, "Grade I-III" has been added after "…PTEN-mutated meningioma."
- The following text was deleted from the first sentence of the first paragraph: "in regard to two co-primary endpoints."
- The former second ("The co-primary endpoints are response rate (RR) and progression-free survival at 6 months [PFS6] after starting treatment."), fourth ("Patients with recurrent or progressive Grade I-III meningiomas will be eligible for this trial."), and fifth ("Samples will undergo central pathology review. Patient's tumor samples will undergo mutational testing.") sentence have been removed.
- In the new third sentence, "confirmed by central molecular review" has been added after "…PTEN mutations."
- In the fourth sentence, for clarity, the words "up to" have been added after "there will be."
- A few fifth sentence has been added, which reads: "Below is a table to summarize the analysis and primary or co-primary endpoints per arm."
- The former second paragraph regarding how the study is powered for the RR endpoint and PFS6 endpoint has been removed.
- A table has been added to summarize the analysis cohorts and primary or co-primary endpoints per arm.
- The new second paragraph (former third paragraph) below the table now reads: "There are no planned interim analyses. The sample size calculations were done with EAST v6.3 and PASS 15.01."

Section 13.2 (Statistical Design and Analysis for the Primary Endpoint)

- The first sentence of Section 13.2.1 now reads: "The primary end points used in this study are progression-free survival at 6 months (PFS6) and response rate (RR)."

- The statistical design for the abemaciclib arm has been added to Section 13.2.2 as paragraphs 10-12.
- In Section 13.2.2, under Overall power and family-wise alpha consideration, the over-all type I error bound of is 19%, formerly 15%.
- The analysis plan for abemaciclib has been added to Section 13.2.3 as the fifth paragraph.

Section 13.3.1 (Sample Size)

- The first sentence of the second paragraph has been updated to reflect that there will be a total of 108 evaluable patients (previously 84) and 24 patients in the NF2-abemaciclib arm.
- The underlined phrase has been added to the second sentence of the third paragraph: "We anticipate accruing an additional 3 patients in NF2 mutation – GSK2256098 grade II/III cohort and NF2, CDKx, CCNx mutations– abemaciclib grade II/III cohort to account for ineligibilities or cancellations."

Section 16.0 (References)

References 18-21 have been added, found in the text under Section 1.2.

Appendix IV (Patient Medication Diaries)

- A medication diary for abemaciclib has been added as Appendix IV-D. The cover page of this appendix has been updated accordingly.

Appendix VII (Central Laboratory Genotype Testing Procedures)

Information regarding the abemaciclib arm has been added as the first row in the table under **Target Mutations.**

Appendix VIII (Patient Drug Information Handouts and Wallet Cards)

A drug information handout and wallet card for abemaciclib has been added as Appendix VIII-C. The cover page of this appendix has been updated accordingly.

UPDATES TO THE MODEL CONSENT

Why is this study being done?

- Language explaining abemaciclib has been added as the fifth paragraph
- In the last paragraph, the total number of people taking place in the study has been updated to 108, previously 84.

What are the study groups?

- A sentence has been added to indicate that the vismodegib arm has been closed to new patient participation
- A new fifth paragraph regarding patients with a genetic change in the CDK pathway receiving abemaciclib has been added.
- "CDK – abemaciclib" has been added to the list of medications in the first sentence of the seventh paragraph.
- Abemaciclib has been added to the schema table.

What extra tests and procedures will I have if I take part in this study?

Under "Before you begin the study:" clarification has been added to the third bullet point, which states that patients who are unable to undergo a brain MRI can have a brain CT done.

What possible risks can I expect from taking part in this study?

The risk list for abemaciclib has been added.

<u>What are the costs of taking part in this study?</u>
Abemaciclib has been added to the list of drugs in the first sentence of the first paragraph.

A replacement protocol document and model consent form have been issued

---

ATTACH TO THE FRONT OF EVERY COPY OF THIS PROTOCOL

ALLIANCE FOR CLINICAL TRIALS IN ONCOLOGY

PROTOCOL UPDATE TO ALLIANCE A071401

PHASE II TRIAL OF SMO/AKT/NF2/CDK INHIBITORS IN PROGRESSIVE MENINGIOMAS WITH SMO/AKT/NF2/CDK PATHWAY MUTATIONS

X	**Update:**	X	**Status Change:**
X	Eligibility changes		Activation
X	Therapy / Dose Modifications / Study Calendar changes		Closure
X	Informed Consent changes		Suspension / temporary closure
X	Scientific / Statistical Considerations changes	X	Reactivation
X	Data Submission / Forms changes		
X	Editorial / Administrative changes		
	Other:		

***If your site utilizes the CIRB as your IRB of record:* No recommended IRB level of review is provided by the Alliance since the CIRB is the IRB of record for this trial. The site has 30 days after the posting of this amendment to implement it at their site. Please refer to the amendment application and CIRB guidelines for further instructions.**

***If your site utilizes a local IRB as your IRB of record:* IRB approval (or disapproval) is required within 90 days. Please follow your local IRB guidelines. Expedited IRB Approval is allowed. The proposed changes in this amendment are minor and do not affect the overall risk/benefit ratio.**

UPDATES TO THE PROTOCOL

Cover Page (Page 1)

- The title of the study has been changed to "Phase II Trial of SMO/AKT/NF2/CDK Inhibitors in Progressive Meningiomas with SMO/AKT/NF2/CDK Pathway Mutations."
- Dr. Ian Dunn's contact information has been updated.
- Dr. David E. Piccioni has been added as the SWOG Study Champion.
- Dr. Susan Geyer has replaced Dr. Karla Ballman as the Primary Statistician and the contact information has been updated accordingly.
- Diane Feldman has replaced Sakuni Taniya Silva as the Protocol Coordinator. Her contact information has been added.

CTSU Address and Contact Information (Page 3)
The CTSU Contact Information table has been updated with current CTSU boilerplate language.

Schema (Page 4)

- The title of the study has been updated to reflect the change on the cover page.
- The **Required Initial Laboratory Values** has been updated from "Require initial laboratory values as indicated in Section 3.3.11" to "Require initial laboratory values as indicated in Section 3.3.10."

Section 1.11 (Clinical Experience with Abemaciclib)
The first sentence in the sixth paragraph has been updated from "The results suggest that abemaciclib has clinical activity in patients with NSCLC and metastatic breast cancer" to "The results suggest that abemaciclib has clinical activity in patients with NSCLC and metastatic breast cancer and has been FDA approved for her2 negative advanced breast cancer."

Section 3.3.9 (Diabetic status)
The first sentence in the eighth bullet point has been corrected from "Patients without a pre-existing diagnosis of Type 2 diabetes mellitus must have fasting glucose ≥ 7.0 mmol/L (126 mg/dL)" to "Patients without a pre-existing diagnosis of Type 2 diabetes mellitus must have fasting glucose ≤ 7.0 mmol/L (126 mg/dL)."

Section 3.3.10 (Required Initial Laboratory Values)
Magnesium has been removed as one of the required initial laboratory values.

Section 4.1 (Investigator and Research Associate Registration with CTEP [formerly CTEP Registration Procedures])
The section has been renamed "Investigator and Research Associate Registration with CTEP" and all the text has been completely updated with current CTSU boilerplate language.

Section 4.2 (CTSU Site Registration Procedures)

- All the text in Section 4.2 (CTSU Site Registration Procedures) has been completely revised to include updated CTSU boilerplate language.
- A new Section 4.2.1 (Additional site registration requirements) and Section 4.2.2 (Protocol specific requirements for A071401 site registration) has been added to include updated CTSU boilerplate language. Subsequent sections have been renumbered.
- The former Section 4.2.2 (Requirement for A071401 Site Registration) has been completely removed.
- All the text in Section 4.2.3 (formerly Section 4.2.1) (Submitting Regulatory Requirement) and Section 4.2.5 (formerly Section 4.2.4) (Checking Site's Registration Status [formerly Checking Your Site's Registration Status]) has been completely revised to include updated CTSU boilerplate language.

Section 4.5 (Patient Registration/Randomization Procedure)
All the text has been completely revised to include updated CTSU boilerplate language.

Section 4.7 (Treatment Assignments and Patient Cohorts)

- The third sentence in the second paragraph has been completely removed.
- The following information has been added within parenthesis below the "Group 1" bullet, under Specific Treatment Groups: "(Closed to new patient enrollment on 7/19/2017)."

- The following information has been added within parenthesis below the “Group 3” bullet, under Specific Treatment Groups: “(Closed to new patient enrollment on 02/07/2018).”
- The following information has been added within parenthesis below the “Group 4” bullet, under Specific Treatment Groups: “(Closed to new patient enrollment on 2/07/2018).”

Section 5.0 (Study Calendar)
The entire study calendar and footnotes has been updated to reflect the updated budget and national coverage analysis, as follows:

- Under laboratory studies, the HbA1C has been separated and listed as a separate line, with an “X” and “PRN” listed under the first three columns.
- The language within parenthesis in the “Serologic Hepatitis B…” line has been removed, and a “PRN” has been added in the “Prior to registration” column of that row, to indicate that it is required as clinically indicated.
- The Fasting cholesterol, triglycerides test has removed “C” under the “Prior to Registration” column.
- The Magnesium test within the “Electrolytes” row has been separated below as a separate line, with an “X (10)” in the second column and a “PRN” in the third and fourth columns. Corresponding footnote 10 reads: “Magnesium performed only Day 1 of Cycle 1.”
- Footnotes 4, A, B and C have been updated to be more clear.

Section 6.1 (Data Collection and Submission)
All the text has been completely revised to include updated CTSU boilerplate language.

Section 6.1.2 (Data Quality Portal)
The fifth paragraph has been completely revised to include updated CTSU boilerplate language.

Section 6.3 (CT and MR Imaging Data Submission)
All the text in subsection, “TRIAD based data transfer,” has been completely revised to include updated CTSU boilerplate language.

Section 7.1 (Arm A [SMO/PTCH1 mutation – Vismodegib])
The following language has been added beneath the table, “**Note: Closed to accrual February 2018**.”

Section 7.2 (Arm B [NF2 mutation – GSK2256098])
The following language has been added beneath the table, “**Note: Closed to accrual July 2017**.”

Section 7.5 (Important interaction information for patients receiving GSK2256098, AZD5363, and abemaciclib)

- Section 7.5.3 has been renamed from “CYP2D6 Inhibitors” to “CYP2D6 Substrates.”
- Section 7.5.4 (CYP3A4 Substrates), Section 7.5.5 (CYP2C9 Substrates) and Section 7.5.6 (Concurrent HMG CoA Reductase Inhibitors) has been added to ensure it is consistent with the AZD5363 investigator brochure.

Section 8.1.7 (Rash)
The second sentence in the second paragraph has been updated from “The need for an interruption or dose reduction of AZD5363 should be considered with reference to the Rash Management Algorithm in Appendix X” to “The need for an interruption or dose reduction of AZD5363 should be considered with reference to the Rash Management Algorithm in Appendix IX.”

Section 8.1.9 (Hyperglycemia)
The second sentence has been updated from "A suggested algorithm for the management of hyperglycemia is provided in Appendix X" to "A suggested algorithm for the management of hyperglycemia is provided in Appendix IX."

Section 10.3 (AZD5363 [NSC # 782347, IND #126926])
All the text in subsection "*Drug Interactions*" has been completely updated to ensure it is consistent with the AZD5363 investigator brochure.

Section 13.2.1 (Primary endpoints)
The third sentence in the second paragraph has been completely removed.

UPDATES TO THE MODEL CONSENT

Title Page (Page 1)

- The Official Study Title for Internet Search on http://www.ClinicalTrials.gov has been updated from "A071401, Phase II Trial of SMO/AKT/NF2 Inhibitors in Progressive Meningiomas with SMO/AKT/NF2 Mutations" to "A071401, Phase II Trial of SMO/AKT/NF2/CDK Inhibitors in Progressive Meningiomas with SMO/AKT/NF2/CDK Pathway Mutations."
- The bulleted paragraphs "**Notes for local investigators**" at the beginning of the consent form has been removed.

Why is this study being done?

- After the sixth sentence in the second paragraph, the following language has been added, "New patient participating in this group February of 2018."
- The heading for the third paragraph has been removed.
- After the fifth sentence in the third paragraph, the following language has been added, "New patient participation in this group closed July of 2017."
- The first sentence in the fifth paragraph has been revised from "The purpose of this study is to test the good and bad effects of the drugs called abemaciclib" to "The medication, abemaciclib, blocks CDK pathway (CDKN2A, CDK4, CDK6, CCND1, CCND2, CCND3, or CCNE1 are part of the same pathway)."
- After the third sentence in the fifth paragraph, the following language has been added, "There will be about 24 people taking part in this group."
- The first sentence in the sixth paragraph has been revised from "There will be about 108 people taking part in this study" to "In all, there will be about 108 people taking part in this study."

What are the study groups?
After the first sentence in the sixth paragraph the following language has been added, "You will be offered the drug from the more rare gene alteration."

What possible risks can I expect from taking part in this study?

- The heading for the possible side effects tables of vismodegib has been updated from "**Possible Side Effects of Vismodegib (for patients with SMO or PTCH1 mutations)**" to "**Possible Side Effects of Vismodegib (for patients with SMO or PTCH1 mutations enrolled before February of 2018)**."

- In the **Reproductive risks** subsection, new language regarding the reproductive risks if you are receiving abemaciclib has been added.

What are my rights in this study?
The second sentence in the second paragraph has been completely removed.

What are the costs of taking part in this study?
After the third paragraph, the following language has been added as a new paragraph:

- If you are found to have an altered (mutated) AKT1, PIK3CA or PTEN gene, you will undergo additional blood tests for blood sugar (HbA1c), cholesterol, and triglycerides. In addition, you will undergo testing for your heart including an EKG and ECHO/MUGA scan. These additional tests will be paid for by the study.

A replacement protocol document and model consent form have been issued

---

ATTACH TO THE FRONT OF EVERY COPY OF THIS PROTOCOL

ALLIANCE FOR CLINICAL TRIALS IN ONCOLOGY

PROTOCOL UPDATE TO ALLIANCE A071401

PHASE II TRIAL OF SMO/AKT/NF2/CDK INHIBITORS IN PROGRESSIVE MENINGIOMAS WITH SMO/AKT/NF2/CDK PATHWAY MUTATIONS

X	**Update:**	X	**Status Change:**
	Eligibility changes		Activation
X	Therapy / Dose Modifications / Study Calendar changes		Closure
X	Informed Consent changes		Suspension / temporary closure
	Scientific / Statistical Considerations changes	X	Reactivation
	Data Submission / Forms changes		
X	Editorial / Administrative changes		
	Other:		

***If your site utilizes the CIRB as your IRB of record:* No recommended IRB level of review is provided by the Alliance since the CIRB is the IRB of record for this trial. The site has 30 days after the posting of this amendment to implement it at their site. Please refer to the amendment application and CIRB guidelines for further instructions.**

***If your site utilizes a local IRB as your IRB of record:* IRB approval (or disapproval) is required within 90 days. Please follow your local IRB guidelines. Expedited IRB Approval is allowed. The proposed changes in this amendment are minor and do not affect the overall risk/benefit ratio.**

UPDATES TO THE PROTOCOL

The naming convention for study agent AZD5363 has been updated in all sections of the protocol document to "Capivasertib."

Study Resources (Page 2)

- Lori Cappello has replaced Wanda L. Dekrey as the A041701 Nursing Contact. All contact information has been updated accordingly.
- The Alliance Imaging contact information has been updated.

Section 5.0 (Study Calendar)

- Footnote A has been added to the "Prior to Registration" time point on the study for the "HbA1c" study to reflect the CTSU National Coverage Analysis.
- Footnote 11 has been added as a new footnote to the "Prior to Registration" time point for the "Electrolytes (Na, K, P, Ca [corrected for serum albumin])" study on the Study Calendar to reflect the National Coverage Analysis.
- Footnote A has been updated from "For patients on AZD5363 only, required prior to registration and as clinically indicated thereafter" to "For patients on capivasertib only. Required prior to registration and as clinically indicated thereafter" for clarity.
- The first sentence in footnote C has been updated from "Required at baseline and every 6 cycles thereafter for patients with NF2 mutation enrolling/enrolled on GSK2256098" to "Required at Day 1 of Cycle 1 and every 6 cycles thereafter for patients with NF2 mutation enrolling/enrolled on GSK2256098" to reflect the National Coverage Analysis.
- The second sentence in footnote C has been updated from "For patients with AKT1/PIK3CA/PTEN mutations enrolled receiving capivasertib required at baseline and as clinically indicated thereafter" to "For patients with AKT1/PIK3CA/PTEN mutations enrolled receiving capivasertib required at Day 1 of Cycle 1 and as clinically indicated thereafter" to reflect the National Coverage Analysis.

Section 6.2.1 (Specimen submission using the Alliance Biospecimen Management System)
William Pisano has replaced Fiona Watkinson as the personnel contact for Central Pathology Review. Contact information has been updated accordingly.

Section 6.3 (CT and MR Imaging Data Submission)
In the **FTP Transfer** subsection, the address for where to submit the data has been updated.

Section 7.1 (SMO/PTCH1 mutation – Vismodegib Arm)
The heading has been renamed from "Arm A (SMO/PTCH1 mutation - Vismodegib)" to "SMO/PTCH1 mutation – Vismodegib Arm" for clarity.

Section 7.2 (NF2 mutation – GSK2256098 Arm)
The heading has been renamed from "Arm B (NF2 mutation – GSK2256098)" to "NF2 mutation – GSK2256098 Arm" for clarity.

Section 7.3 (AKT1/PIK3CA/PTEN mutation – Capivasertib Arm)
The heading has been renamed from "Arm C (AKT1/PIK3CA/PTEN mutation – AZD6363)" to "AKT1/PIK3CA/PTEN mutation – Capivasertib Arm" for clarity.

Section 7.4 (CDK4, CDK6, CDKN2A, CCND1, CCND2, CCND3, CCNE1 alterations- Abemaciclib Arm)
The heading has been renamed from "Arm D (CDK4, CDK6, CDKN2A, CCND1, CCND2, CCND3, CCNE1 alterations- Abemaciclib" to "CDK4, CDK6, CDKN2A, CCND1, CCND2, CCND3, CCNE1 alterations- Abemaciclib Arm" for clarity.

Section 10.1 (Vismodegib [GDC-0449, Erivedge®, NSC# 747691, IND#126926] IND holder: Alliance)
After the *Procurement* subsection, a new subsection entitled, "*Investigator Brochure Availability*," has been added.

Section 10.2 (GSK2256098 [NSC# 783781, IND #126926])
After the *Procurement* subsection, a new subsection entitled, "*Investigator Brochure Availability*," has been added.

Section 10.3 (Capivasertib [NSC # 782347, IND #126926])

- After the *Procurement* subsection, a new subsection entitled, "*Investigator Brochure Availability*," has been added.
- The third and fourth sentence in the *Formulation* subsection has been updated to "Capivasertib film-coated tablets contain capivasertib, microcrystalline cellulose, dibasic calcium phosphate, croscarmellose sodium and magnesium stearate. The tablet film coat contains hypromellose, titanium dioxide, polyethylene glycol, polydextrose, copovidone plasdone, medium chain triglycerides, yellow iron oxide, red iron oxide and black iron oxide."

Section 10.4 (Abemaciclib [LY2835219, VERENZIO™, NSC#783671])

After the *Procurement* subsection, a new subsection entitled, "*Investigator Brochure Availability*," has been added.

UPDATES TO THE MODEL CONSENT

The naming convention for study agent AZD5363 has been updated in all sections of the model consent document to "Capivasertib."

What are the costs of taking part in this study?

The fourth paragraph has been updated to "If you are found to have an altered (mutated) AKT1, PIK3CA or PTEN gene, before you start treatment with capivasertib, you will have additional blood tests for blood sugar (HbA1c), cholesterol, and triglycerides. In addition, before you start capivasertib, you will undergo testing for your heart that includes an EKG and ECHO/MUGA scan. These additional pre-treatment tests will be paid for by the study."

A replacement protocol document and model consent form have been issued

---

ATTACH TO THE FRONT OF EVERY COPY OF THIS PROTOCOL

ALLIANCE FOR CLINICAL TRIALS IN ONCOLOGY

PROTOCOL UPDATE TO ALLIANCE A071401

PHASE II TRIAL OF SMO/AKT/NF2/CDK INHIBITORS IN PROGRESSIVE MENINGIOMAS WITH SMO/AKT/NF2/CDK PATHWAY MUTATIONS

X	**Update:**	X	**Status Change:**
X	Eligibility changes		Activation
X	Therapy / Dose Modifications / Study Calendar changes		Closure
X	Informed Consent changes		Suspension / temporary closure
	Scientific / Statistical Considerations changes	X	Reactivation
	Data Submission / Forms changes		
X	Editorial / Administrative changes		
X	Other: Updated CAEPR for capivasertib (AZD5363)		

The changes included in this update to A071401 have been made in response to the NCI Action Letter from Dr. John Sandlund (john.sandlund@nih.gov). This Action Letter is posted on the A071401 study page on the Alliance and CTSU websites. A revised CAEPR for capivasertib (AZD5363) with new risks has been added to the protocol. Therefore, the model consent form has been revised to incorporate these new risks, consistent with the NCI Model Consent Template instructions. There are no changes to the risk/benefit ratio.

***If your site utilizes the CIRB as your IRB of record:* No recommended IRB level of review is provided by the Alliance since the CIRB is the IRB of record for this trial. The site has 30 days after the posting of this amendment to implement it at their site. Please refer to the amendment application and CIRB guidelines for further instructions.**

***If your site utilizes a local IRB as your IRB of record:* IRB approval (or disapproval) is required within 90 days. Please follow your local IRB guidelines. Expedited IRB Approval is allowed. The proposed changes in this amendment are minor and do not affect the overall risk/benefit ratio.**

UPDATES TO THE PROTOCOL

Schema (Page 4)

- The first bullet point in the **Registration Eligibility Criteria** has been updated to the following for consistency: "Presence of specific SMO/PTCH1, NF2 or AKT1/PIK3CA./PTEN/CDK pathway mutations (see Section 3.3.1)."
- The third bullet point in the **Registration Eligibility Criteria** has been updated to the following for clarity: "Measurable disease as defined by a bi-dimensionally measurable (See Section 3.3.2)."
- The fourth bullet point in the **Registration Eligibility Criteria** has been updated to the following for consistency: "~~main~~ Target lesion on MRI brain or CT head images (MRI preferred) (See Section 3.3.2)."
- The eighth bullet point in the **Registration Eligibility Criteria** has been updated to the following for clarity: "Steroid dosing stable for at least 4 days prior to registration."

Section 1.7 (Clinical Experience with Capivasertib as Monotherapy)

The section has been completely updated to reflect the updated the new version of capivasertib IB.

Section 3.3.1 (Documentation of Disease)

- The third sentence of the first bullet point has been updated to the following in order to allow for a wider window: "The change must occur between scans separated by no more than ~~14~~ 25 months."
- The second sentence of the second bullet point has been updated to the following in order to allow for a wider window: The change must occur between scans separated by no more than ~~12~~ 25 months.

Section 3.3.2 (Measurable disease)

The first sentence of the first paragraph has been updated to the following for clarity: "Measurable disease is defined by a bidimensionally measurable main lesion on MRI or CT images (MRI preferred) with clearly defined margins and a minimum diameter of 10 mm in both dimensions."

Section 3.3.7 (Patient history)

The fifteenth bullet point has been updated to the following for accuracy: "Cardiac ejection ~~injection~~ fraction outside institutional range of normal or < 50% (whichever is higher) as measured by echocardiogram…"

Section 4.2 (CTSU Site Registration Procedures)

Section 4.2.2 (Protocol specific requirements for A071401 site registration) has been completely removed as IROC credentialing is not required. Subsequent sections have been renumbered.

Section 5.0 (Study Calendar)

The second paragraph of Footnote B has been updated to the following for clarity: "For patients with NF2 mutation enrolling/enrolled on abemaciclib: ECG should be performed if clinically indicated prior to registration and ~~for the following:~~ every cycle from the start of treatment, and at post-treatment follow up."

Section 7.5 (Important interaction information for patients receiving GSK2256098, capivasertib, and abemaciclib)

References to "Appendix IX" has been updated to "Appendix VIII" in Sections 7.5.1 (CYP3A4 Inhibitors), 7.5.2 (CYP3A4 Inducers), 7.5.3 (CYP2D6 Substrates), 7.5.4 (CYP3A4 Substrates, and 7.5.5 (CYP2C9 Substrates) to address error.

Section 8.4.3 (Diarrhea)
This section has been completely updated according to capivasertib PSSR edition 3.

Section 8.4.5 (Hepatic toxicity)
The following text has been updated to the following to address error: "Please see Appendix IX ~~X~~ Figure 3 for hepatic toxicity management."

Section 8.4.6 (Dermatologic toxicity)

- This section and subsequent sections have been renumbered to address error.
- This section has been completely updated according to capivasertib PSSR edition 3.

Section 8.4.7 (Metabolism)
This section has been completely updated according to capivasertib PSSR edition 3.

Section 8.4.8 (Other toxicities)
This section has been completely updated according to capivasertib PSSR edition 3.

Section 9.3.1 (Phase 1 and Early Phase 2 Studies)
In the "Additional Instructions or Exclusion to CTEP-AERS Expedited Reporting Requirements for Phase 1 and Early Phase 2 Trials Utilizing an Agent Under a non-CTEP IND" subsection, new requirements for patients receiving capivasertib (AZD5363) have been added after the fifth bullet point.

Section 9.5 (Comprehensive Adverse Events and Potential Risks list [CAEPR] for Capivasertib [AZD5363] [NSC 782347])
This section has been revised to include the updated capivasertib (AZD5363) CAEPR (Version 2.4, March 29, 2021) provided by CTEP. Changes from Version 2.2 to Version 2.4 include the following:

- The footnotes have been reordered.
- Decrease in Risk Attribution:
  - Changed to Also Reported on AZD5363 Trials But With Insufficient Evidence for Attribution from Less Likely: Abdominal pain; Anemia; Back pain; Creatinine increased; Constipation; Cough; Dizziness; Edema limbs; Headache; Proteinuria
  - Changed to Also Reported on AZD5363 Trials But With Insufficient Evidence for Attribution from Likely: Fatigue; Fever
- Deleted Risk:
  - Also Reported on AZD5363 Trials But With Insufficient Evidence for Attribution: Acute kidney injury; Alanine aminotransferase increased; Alkaline phosphatase increased; Aspartate aminotransferase increased; Blood bilirubin increased; Confusion; Dehydration; Esophageal pain; Gastrointestinal disorders - Other (intestinal obstruction); Glucose intolerance; Heart failure; Hypercalcemia; Hypokalemia; Hypomagnesemia; Hyponatremia; Hypophosphatemia; Lung infection; Malaise; Neuralgia; Neutrophil count decreased; Oropharyngeal pain; Pleural effusion; Productive cough; Sepsis; Skin infection; Small intestinal obstruction; Thromboembolic event; Tumor pain; Urinary tract infection; White blood cell decreased
- Provided Further Clarification:
  - Footnote #2 has been added: Mucositis oral may include aphthous ulcer, aphthous stomatitis, mouth ulceration.
  - Footnote #3 has been updated from "Rash may include dry skin, skin fissures, xeroderma, dermatitis exfoliative, exfoliative rash, eyelid exfoliation, skin exfoliation, xerosis, rashes

and acnes, rash, rash generalized, rash macular, rash maculo-papular, rash maculovesicular, rash morbilliform, rash vesicular, rash follicular, acne pustular, rash pustular, folliculitis, eyelid folliculitis, acne, dermatitis acneiform, drug eruption pruritus, eyelids pruritus, pruritus generalized, erythema, and erythematous rash" to "Rash may include rash erythematous, rash maculo-papular, and rash popular".

Section 10.3 (Capivasertib (AZD5363) (NSC # 782347, IND #126926)

- The first sentence in the first paragraph in the *Drug Interactions* subsection has been completely replaced with the following sentence in accordance with the updated capivasertib IB, "Capivasertib is metabolized primarily by CYP3A4 and UGT2B7 enzymes."
- A new paragraph has been added after the second paragraph in the *Drug Interactions* subsection to provide information in accordance with the updated capivasertib IB.
- The following sentence has been added after the first sentence in the **Distribution** paragraph in the *Pharmacokinetics* subsection, "The mean volume of distribution ranged from 517 to 1215 L, indicating that capivasertib is likely to be well distributed in the body."
- The Metabolism paragraph in the *Pharmacokinetics* subsection has been completely updated in accordance with the updated capivasertib IB.

UPDATES TO THE MODEL CONSENT

Why is this study being done?

- The fourth sentence of the fourth paragraph has been updated to the following: "Researchers hope to learn if the study drug will shrink your tumor ~~the cancer by at least one-half compared to its present size~~."
- The fourth sentence of the fifth paragraph has been updated to the following: "Researchers hope to learn if the study drug will shrink ~~the cancer by at least one-half compared to its present size~~ your tumor.

What are the study groups?

The second sentence of the sixth paragraph has been updated to the following: "If you fall into this category, you ~~You~~ will be offered the drug from the more rare gene alteration."

What possible risks can I expect from taking part in this study?

Based on the updated CAEPR described above, the following changes have been made to the NCI condensed risk profile for capivasertib (AZD5363) (found under "**Possible Side Effects of capivasertib [AZD5363] [for patients with AKT1, PIK3CA or PTEN mutations]**"):

- Decrease in Risk Attribution:
  - Changed to Also Reported on AZD5363 Trials But With Insufficient Evidence for Attribution from Common (removed from the Risk Profile): Tiredness; Fever
  - Changed to Also Reported on AZD5363 Trials But With Insufficient Evidence for Attribution from Occasional (removed from the Risk Profile): Anemia which may require blood transfusion; Pain; Constipation; Swelling of arms or legs; Dizziness; Headache; Cough
- Deleted Risk:
  - Occasional: Weight loss; Shortness of breath

A replacement protocol document and model consent form have been issued

---

ATTACH TO THE FRONT OF EVERY COPY OF THIS PROTOCOL

ALLIANCE FOR CLINICAL TRIALS IN ONCOLOGY

PROTOCOL UPDATE TO ALLIANCE A071401

PHASE II TRIAL OF SMO/AKT/NF2/CDK INHIBITORS IN PROGRESSIVE MENINGIOMAS WITH SMO/AKT/NF2/CDK PATHWAY MUTATIONS

X	**Update:**	X	**Status Change:**
X	Eligibility changes		Activation
X	Therapy / Dose Modifications / Study Calendar changes		Closure
X	Informed Consent changes		Suspension / temporary closure
	Scientific / Statistical Considerations changes		Reactivation
	Data Submission / Forms changes		
X	Editorial / Administrative changes		
	Other:		

***If your site utilizes the CIRB as your IRB of record:* No recommended IRB level of review is provided by the Alliance since the CIRB is the IRB of record for this trial. The site has 30 days after the posting of this amendment to implement it at their site. Please refer to the amendment application and CIRB guidelines for further instructions.**

***If your site utilizes a local IRB as your IRB of record:* IRB approval (or disapproval) is required within 90 days. Please follow your local IRB guidelines. Expedited IRB Approval is allowed. The proposed changes in this amendment are minor and do not affect the overall risk/benefit ratio.**

UPDATES TO THE PROTOCOL

Title Page
Lilli Johnson has replaced Diane Feldman as the protocol coordinator. All contact information has been updated accordingly.

Study Resources (Page 2)
The email address for Lori Cappello, the A071401 Nursing Contact, has been updated.

Section 3.3.7 (Patient history)
The fourth sub bullet under the "For patients going on to receive capivasertib…." bullet has been updated to the following to address error: "Uncontrolled hypotension ~~hypertension~~ (SBP < 90 mmHg and/or DBP < 50 mmHg)."

Section 5.0 (Study Calendar)
The second paragraph of footnote B has been updated from "For patients with NF2 mutation enrolling/enrolled on abemaciclib: ECG should be performed if clinically indicated prior to registration and every cycle from the start of treatment, and at post-treatment follow up" to "For patients with NF2 mutation enrolling/enrolled on abemaciclib: ECG should be performed when clinically indicated at the following time points: Prior to registration, every cycle from the start of treatment, and at post-treatment follow up" for clarity.

Section 10.1 (Vismodegib [GDC-0449, Erivedge®, NSC# 747691, IND#126926] IND holder: Alliance)
Under the "Investigator Brochure Availability" heading, the email address for the Alliance Central Protocol Operations Program office has been updated to "protocols@alliancenctn.org".

Section 10.2 (GSK2256098 [NSC# 783781, IND #126926])
Under the "Investigator Brochure Availability" heading, the email address for the Alliance Central Protocol Operations Program office has been updated to "protocols@alliancenctn.org".

Section 10.3 (Capivasertib [AZD5363] [NSC # 782347, IND #126926])
Under the "Investigator Brochure Availability" heading, the email address for the Alliance Central Protocol Operations Program office has been updated to "protocols@alliancenctn.org".

Section 10.4 (Abemaciclib [LY2835219, VERENZIO™, NSC#783671])
Under the "Investigator Brochure Availability" heading, the email address for the Alliance Central Protocol Operations Program office has been updated to "protocols@alliancenctn.org".

UPDATES TO THE MODEL CONSENT

What are the study groups?

- The groups listed in the schema table have been renumbered in order to be consistent with the protocol.
- Group 7 in the schema table has been updated to the following for consistency: "Tumor has CDK ~~gene~~ pathway."

A replacement protocol document and model consent form have been issued

---

ATTACH TO THE FRONT OF EVERY COPY OF THIS PROTOCOL

Protocol Update #14

ALLIANCE FOR CLINICAL TRIALS IN ONCOLOGY

PROTOCOL UPDATE TO ALLIANCE A071401

PHASE II TRIAL OF SMO/AKT/NF2/CDK INHIBITORS IN PROGRESSIVE MENINGIOMAS WITH SMO/AKT/NF2/CDK PATHWAY MUTATIONS

X	**Update:**		**Status Change:**
	Eligibility changes		Activation
	Therapy / Dose Modifications / Study Calendar changes		Closure
	Informed Consent changes		Suspension / temporary closure
	Scientific / Statistical Considerations changes		Reactivation
	Data Submission / Forms changes		
	Editorial / Administrative changes		
X	Other: Capivasertib CAEPR update		

The changes included in this update to A071401 have been made in response to the NCI Action Letter from Dr. Rabih Said (rabih.said@nih.gov), Dr. Jeffery Moscow, and Dr. Meg Mooney . This Action Letter is posted on the A071401 study page on the Alliance and CTSU websites. A revised CAEPR for capivasertib (AZD5363) with a new risk has been added to the protocol. There are no changes to the risk/benefit ratio.

<u>If your site utilizes the CIRB as your IRB of record:</u> No recommended IRB level of review is provided by the Alliance since the CIRB is the IRB of record for this trial. The site has 30 days after the posting of this amendment to implement it at their site. Please refer to the amendment application and CIRB guidelines for further instructions.

<u>If your site utilizes a local IRB as your IRB of record:</u> IRB approval (or disapproval) is required within 90 days. Please follow your local IRB guidelines. Expedited IRB Approval is allowed. The proposed changes in this amendment are minor and do not affect the overall risk/benefit ratio.

UPDATES TO THE PROTOCOL

Section 9.5 (Comprehensive Adverse Events and Potential Risks list (CAEPR) for AZD5363 (capivasertib, NSC 782347)
This section has been revised to include the updated capivasertib CAEPR (Version 2.5, October 18, 2021) provided by CTEP. Changes from Version 2.4 to Version 2.5 include the following:

- Added new risk:
  - Rare but serious: Erythema multiforme

UPDATES TO THE MODEL CONSENT

Possible Side Effects of capivasertib (AZD5363) (for patients with AKT1, PIK3CA or PTEN mutations)
The CAEPR version date has been removed from capivasertib risk list.

A replacement protocol document and model consent form have been issued

---

ATTACH TO THE FRONT OF EVERY COPY OF THIS PROTOCOL

ALLIANCE FOR CLINICAL TRIALS IN ONCOLOGY

PROTOCOL UPDATE TO ALLIANCE A071401

PHASE II TRIAL OF SMO/AKT/NF2/CDK INHIBITORS IN PROGRESSIVE MENINGIOMAS WITH SMO/AKT/NF2/CDK PATHWAY MUTATIONS

X	**Update:**		**Status Change:**
X	Eligibility changes		Activation
X	Therapy / Dose Modifications / Study Calendar changes		Closure
X	Informed Consent changes		Suspension / temporary closure
	Scientific / Statistical Considerations changes		Reactivation
	Data Submission / Forms changes		
X	Editorial / Administrative changes		
X	Other: Updated CTSU template language		

If your site utilizes the CIRB as your IRB of record
No recommended IRB level of review is provided by the Alliance since the CIRB is the IRB of record for this trial. The site has 30 days after the posting of this amendment to implement it at their site. Please refer to the amendment application and CIRB guidelines for further instructions.

If your site utilizes a local IRB as your IRB of record
IRB approval (or disapproval) is required within 90 days. Please follow your local IRB guidelines. Expedited IRB approval is allowed. The proposed changes in this amendment are minor and do not affect the overall risk/benefit ratio.

UPDATES TO THE PROTOCOL

Cover Page (Page 1)

- Alexandra LeVasseur has replaced Lilli Johnson as the Protocol Coordinator; all contact information has been updated.
- Kayla Pride has replaced Meagan Odegaard as the Data Manager; all contact information has been updated.

Protocol Contacts (Page 2)

- Amanda Sand has replaced Helen Tollefson as the contact for paraffin-embedded tissue at the Alliance Biorepository at Mayo Clinic; all contact information has been updated.
- Katie Halverson has replaced Roxann Neumann as the contact for non-paraffin biospecimens at the Alliance Biorepository at Mayo Clinic; all contact information has been updated.

CTSU Address and Contact Information (Page 3)

All text within the table has been updated to reflect current CTSU boilerplate language.

Eligibility Summary (Page 4)

- In the 5th criterion, the phrase "cancer-directed hormonal therapy, or" has been added for consistency with Section 3.3.3.
- A new 16th criterion has been added for consistency with Section 3.4.1 which reads: "For abemaciclib arm: grade 2/3 disease as determined by central pathology review."

Schema (Page 5)

In the box for the Abemaciclib arm, the phrase "(closed to accrual October 2022)" has been added, and all text within the box has been greyed out to indicate this arm is no longer open to enrollment.

Section 3.3 (Registration Eligibility Criteria)

- In Section 3.3.3, in the 3rd bullet, the phrase "cancer-directed hormonal therapy" has been added to update the types of prior treatment that are not permitted.
- In Section 3.3.10, the following changes have been made:
  - The 5th laboratory value has been removed (previously, "UPC ≤ 45mg/mmol") as this is no longer required for eligibility.
  - In the now 7th laboratory value, the word "Phosphorus" has been removed as this is no longer required for eligibility.
  - In the now 9th laboratory value, the word "Mean" has been removed as only a single EKG is required for eligibility.
- A new criterion has been added as Section 3.4.1 which reads: "**Grade 2/3 disease as determined by central pathology review**." All subsequent subsections have been renumbered accordingly.

Section 4.0 (Patient Registration)

Text throughout this section has been updated to reflect current CTSU boilerplate language.

Section 4.7 (Treatment Assignments and Patient Cohorts)

In the 7th bullet below the 3rd paragraph, the phrase "(Closed to new patient enrollment on 10/03/2022)" has been added to reflect the closure to enrollment for this treatment arm.

Section 5.0 (Study Calendar)

- In Footnote *, a new 4th sentence has been added that reads: "See Section 15.0 for information regarding permitted activities provided by the Local Healthcare Provider; however, imaging should continue to be performed at the registering institution."
- In Footnote ***, the phrase "(+/-1 month)" has been added to the 1st sentence for clarity.
- In Footnote B, the following changes have been made to clarify EKG requirements:
  - A new 1st sentence has been added for clarity which reads: "EKG required on all patients for resting heart rate at eligibility (i.e. prior to registration); triplicate EKG at eligibility only required for patients assigned to capivasertib arm, all other treatment arms should have single EKG at eligibility (see Section 3.3.10)."

- In the now 2nd sentence, the phrase "After registration, patients with NF2 mutation enrolled to receive GSK2256098, single EKG must be performed 1 hour after taking…" has replaced "For patients with NF2 mutation enrolling/enrolled to receive GSK2256098, EKG must be performed at 2 time points: within 28 days prior to registration, and 1 hour after taking…" for clarity.
- In the now 3rd sentence, the word "enrolling" has been removed" and "EG" has been corrected to "EKG."
- In the now 4th sentence, the phrase "; after registration, EKG should be performed as clinically indicated" has been added for clarity.
- A new 5th sentence has been added that reads: "For triplicate EKGs, it is recommended that three individual EKGs should be obtained in succession, no more than 2 minutes apart, and the full set of triplicates should be completed within 5 minutes."

Section 6.1 (Data Collection and Submission)
Text throughout this section has been updated to reflect current CTSU boilerplate language.

Section 6.1.3 (ICAREdata)
A new section has been added to include language regarding ICARE.

Section 6.2.1 (Specimen submission using the Alliance Biospecimen Management System)
Contact information for questions related to blood and tissue submission has been updated.

Section 6.3 (CT and MR Imaging Data Submission)
Text throughout this section has been updated to reflect current CTSU boilerplate language.

Section 8.1.12 (Alliance Policy Concerning the Use of Growth Factors)
In the 2nd list item under the 5th paragraph, a new subpart d has been added that reads: "Biosimilars are allowed per institutional standards."

Section 8.4 (Capivasertib Dose Modifications)
All text throughout Section 8.4.3, Section 8.4.6, and Section 8.4.7 has been revised to reflect updated dose modification guidance for capivasertib.

Section 13.8 (Inclusion of Women and Minorities)
All text below the enrollment table has been removed for consistency with the Alliance model protocol.

Section 15.0 (General Regulatory Considerations and Credentialing)
All text within the section has been replaced with four new paragraphs which provide information regarding permitted activities provided by the Local Healthcare Provider.

Section 15.1 (IRB Terminations)
A new section has been added to note institutions must not close a trial with the IRB of record until a formal notice from the Alliance regarding termination to patient follow-up has been received.

Appendix VII (Central Laboratory Genotype Testing Procedures)
In the **Target Mutations** table, a new 2nd row has been added under "Abemaciclib" to include NF2 mutations.

UPDATES TO THE MODEL CONSENT

<u>Why is this study being done?</u>

- In the 8th sentence of the 2nd paragraph, the word “closed” has been added as it was erroneously omitted.
- The 5th paragraph has been highlighted in grey to reflect the closure of this treatment arm to new patient enrollment.
- A new 6th sentence has been added to the 5th paragraph which reads: “New patient participation in this group closed October of 2022.”

<u>What are the study groups?</u>

- In the 5th paragraph, a new heading has been added which reads: “<u>The group described below closed to new patient participation in October of 2022</u>.”
- The 5th paragraph has been highlighted in grey to reflect the closure of this treatment arm to new patient enrollment.
- In the box for Group 7, the phrase “Closed as of 10/03/2022” has been added, and all text has been highlighted in grey to reflect the closure of this treatment arm to new patient enrollment.

<u>What extra tests and procedures will I have if I take part in this study?</u>

In the bulleted list below the 2nd paragraph, the following revisions have been made to reflect the laboratory requirements for eligibility:

- The 2nd bullet, “Urine analysis,” has been removed as this is no longer required for eligibility.
- In the now 4th bullet, the phrase “(for patients with NF2 mutation going on to receive GSK2256098)” has been removed as an EKG is required for all patients.
- A new 5th bullet has been added as it had been erroneously omitted. It reads: “ECHO (for patients with AKT/PIK3CA/PTEN mutation going on to receive capivasertib).”

<u>What possible risks can I expect from taking part in this study?</u>

In the **Possible Side Effects of Abemaciclib** table, the risks have been updated to reflect information provided in the updated investigator’s brochure. Specifically, the following changes have been made:

- “Dry mouth” and “Infections” have been added to the Common, Some May Be Serious table.
- “Dizziness,” “Rash/itchiness,” and “Muscle weakness” have been added to the Occasional, Some May Be Serious table.
- A new table for Rare, and Serious has been added to include the risk of “Swelling of the lungs with symptoms that may include shortness of breath, cough, difficulty breathing.”

<u>Who will see my medical information?</u>

New 7th – 11th paragraphs have been added to include language on ICARE.

A replacement protocol document and model consent form have been issued

---

ATTACH TO THE FRONT OF EVERY COPY OF THIS PROTOCOL

ALLIANCE FOR CLINICAL TRIALS IN ONCOLOGY

PROTOCOL UPDATE TO ALLIANCE A071401

PHASE II TRIAL OF SMO/AKT/NF2/CDK INHIBITORS IN PROGRESSIVE MENINGIOMAS WITH SMO/AKT/NF2/CDK PATHWAY MUTATIONS

X	**Update:**		**Status Change:**
X	Editorial/Administrative changes		Activation
X	Eligibility changes		Closure
X	Therapy/Dose Modifications/Study Calendar changes		Suspension
X	Scientific/Statistical Considerations changes		Reactivation
X	Correlative Science/BioMS changes		
X	Informed Consent changes		
X	Other: Updated CTSU template language		

Reconsent is required for all patients. Please follow the policy of your IRB of record regarding notifying patients of new information contained in this update.

No recommended IRB level of review is provided by the Alliance since the CIRB is the IRB of record for this trial. The site has 30 days after the posting of this amendment to implement it at their site. Please refer to the amendment application and CIRB guidelines for further instructions.

UPDATES TO THE PROTOCOL

Cover Page

- The title for Dr. Priscilla Brastianos has been updated to include "& Neuro-Oncology Committee Co-Chair."
- The title for Dr. Evanthia Galanis has been updated to remove "& Neuro-Oncology Committee Chair."
- The title for Dr. Jann Sarkaria has been updated to include "& Neuro-Oncology Committee Co-Chair."
- The telephone number for the Primary Statistician, Dr. Susan Geyer, has been removed.
- Katharine Dooley has replaced Erin Twohy as the Secondary Statistician; all contact information has been updated.
- The email address for the Protocol Coordinator, Alexandra LeVasseur, has been updated.

Protocol Contacts

- The telephone number and institution have been removed for Lori Cappello in alignment with the Alliance Protocol Template as all inquiries should be sent via email.
- Dr. David Chan has replaced Dr. Heidi Finnes as the A071401 Pharmacy Contact; all contact information has been updated accordingly.
- All contact information for the Alliance Biorepository at Mayo Clinic has been removed and replaced with a single email inbox and shipping address for all specimen types.
- The contact information for IROC Ohio has been updated to reflect its change of address.
- For questions regarding status of central pathology review and biomarker testing, Nancy Higgins has replaced Dr. Sandro Santagata and Dr. John Iafrate as the contact.

Section 1.10 (Abemaciclib)

In the 4th sentence of the 3rd paragraph, the word "sex" has replaced the word "gender" to align with current federal mandates per the January 20, 2025 Executive Order.

Section 4.0 (Patient Registration)

All text has been updated to reflect the current CTSU boilerplate language.

Section 4.3 (Patient Pre-Registration Requirements)

A new 4th paragraph has been added within the 3rd bullet point to clarify that if biomarker testing has already been performed by the site, then the sequencing report can be sent for central review for eligibility so that eligible patients may be enrolled before receiving central lab sequencing analysis results.

Section 4.4 (Patient Registration Procedures)

In the last sentence of the 1st bullet, the phrase "(or central review of local sequencing report)" has been added for clarity.

Section 5.0 (Study Calendar)

In the 1st sentence of Footnote D, the phrase "for 1 year" has been corrected to "while on protocol treatment until disease progression" for alignment and consistency with the imaging requirements detailed throughout the protocol. Additionally, a new 3rd sentence has been added for clarity. It reads: "For patients who discontinue treatment for reasons other than progressive disease, scans should be performed every 16 weeks (+/- 4 weeks) until documented progression or until 2 years post-registration."

Section 6.1 (Data Collection and Submission)

All text has been updated to reflect the current CTSU boilerplate language.

Section 6.2 (Specimen collection and submission)

- In the 1st subsection heading, the phrase "pre-registered" has replaced "registered" for accuracy.
- All text under the "For all patients pre-registered to Alliance A071401" heading has been replaced with updated language to more accurately describe the tissue requirements and process overview.
- In Footnote *** below the table, in the 2nd sentence, the phrase "whole blood for ctDNA plasma" has replaced "ctDNA" for consistency with the table.

Section 6.2.1 (Specimen submission using the Alliance Biospecimen Management System)

- In the 2nd paragraph, the email address for BioMS has been updated in two instances.
- In the 4th paragraph, the phrase "whole blood, PPP" has replaced "serum, whole blood" for accuracy.
- The 5th paragraph has been removed for accuracy as a printed packing slip is no longer required.

- The following changes have been made to the language under the "**All tumor tissue for Central Pathology Review and Integral Biomarker Testing**" heading for accuracy:
  - In the 1st sentence of the 1st paragraph, the word "priority" has been added.
  - The 2nd sentence of the 1st paragraph has been removed (previously, "Do not ship specimens on Fridays or Saturdays or for receipt on national holidays").
  - The address below the 1st paragraph has been updated.
  - The contact information below the 2nd paragraph has been updated to include a single contact person for all questions about receipt of specimens or status of central review.
- The following changes have been made to the language below the "**Blood submission for patients…**" heading to utilize a single shipping address and email inbox for all specimen types submitted to the Alliance Biorepository at Mayo Clinic and for accuracy and clarity:
  - In the 1st sentence, the word "priority" has been added.
  - In the 2nd sentence, the phrase "or the day before a national holiday" has been added.
  - In the 1st line of the shipping address, the phrase "Biorepository at Mayo Clinic" has been added, and "BAP Freezer" has been removed.
  - All contact information has been replaced with a single telephone number and email address for ABMAYO, regardless of specimen type.
- The following changes have been made to the language below the "**Tissue submission for…**" heading to utilize a single shipping address and email inbox for all specimen types submitted to the Alliance Biorepository at Mayo Clinic and for accuracy and clarity:
  - The shipping address has been completely replaced with updated information.
  - All contact information has been replaced with a single telephone number and email address for ABMAYO, regardless of specimen type.

Section 6.2.2 (Mandatory tumor tissue sample…)
- In the section heading, the word "**diagnostic**" has been removed for accuracy.
- All text in this section has been replaced with updated language to more accurately describe the tissue requirements and process overview.

Section 6.2.3 (Recurrent/progression tissue submission…)
- In the section heading, the acronym "**FFPE**" has been removed for accuracy.
- In the 1st sentence of the 1st paragraph, the acronym "FFPE" has been removed for accuracy.
- In the 4th paragraph, the phrase "FFPE contact" has been removed for accuracy.

Section 6.2.4 (Blood sample submission [A071401-ST1])
- In the 2nd paragraph, the word "plasma" has been added for consistency.
- The following changes have been made to the language under the "**Whole blood germline DNA**" heading for accuracy:
  - In the 4th sentence of the 1st bullet, the phrase "Biorepository at Mayo Clinic" has replaced "BAP Freezer" for accuracy.
  - In the 2nd bullet, the word "Clinic" has replaced "BAP" for accuracy.
- The following changes have been made to the language under the "**Whole blood for circulating tumor DNA…**" heading for accuracy:
  - In the 2nd sentence of the 4th bullet, the phrase "Biorepository at Mayo Clinic" has replaced "BAP Freezer."
  - In the 3rd sentence of the 1st sub-bullet below the 4th bullet, the word "frozen" has replaced "refrigerated."
  - In the 1st sentence of the 5th bullet, the word "Clinic" has replaced "BAP."

Section 9.3 (Expedited Adverse Event Reporting [CTEP-AERS])
The expedited reporting requirements table has been replaced with an updated table to reflect current NCI reporting requirements.

Section 13.8 (Inclusion of Women and Minorities)

- The word "sex" has replaced the word "gender" in four instances throughout the section to align with current federal mandates per the January 20, 2025 Executive Order.
- The 4th sentence of the 2nd paragraph has been moved to create a new paragraph above the Domestic Planned Enrollment Report table for clarity.
- A new 3rd paragraph and subsequent table entitled Domestic Planned Screening Enrollment Report have been added to ensure the number of patients expected to pre-register are provided.

Appendix VII (Central Laboratory Genotype Testing Procedures)

- In the 1st sentence of the 1st paragraph under the **Methodology** heading, the phrase "Quick-DNA/RNA FFPE kit from Zymo (Zymo Research Corp., Irvine, CA) for a manual rapid nucleic extraction" has replaced "a modified Agencourt FormaPure method automated on a Beckman Coulter NXP workstation (Beckman Coulter, Pasadena, Calif)" to more accurately describe the methods used.
- In the 2nd list item under the **Assay Protocol** heading, the 1st sentence has been removed to more accurately describe the protocol used (previously, "Quality assurance measures will require that each trial sample is submitted with…").
- In the 2nd sentence of the 5th list item under the **Assay Protocol** heading, the phrase "Quick-DNA/RNA FFPE kit from Zymo for a manual rapid nucleic extraction" has replaced "Agencourt FormaPure technology on a Biomek NXp robotic workstation" for accuracy.
- In the 1st sentence of the 9th list item under the **Assay Protocol** heading, the name "Dr. Maria Martinez-Lage Alvarez" has been added.

UPDATES TO THE MODEL CONSENT

Why is this study being done?
The 2nd and 3rd sentences of the 4th paragraph have been replaced with the following language: "Capivasertib has been approved by the FDA to treat other cancers" (previously, "Capivasertib has been tested in other cancers. Capivasertib is not FDA approved.").

How long will I be in this study?

- In the 2nd sentence, the phrase "If you stop taking the medication before your tumor grows" has replaced "After you finish taking the medication" and the phrase "every 4 months for 2 years after you first started the study or until your tumor grows" has replaced "for 2 years" for clarity and consistency with the clinical follow-up requirements outlined in the protocol.
- A new 3rd sentence has been added to clarify that the follow-up period for this study is 5 years from enrollment as this information had been erroneously omitted. The new sentence reads as follows: "After you finish taking the medication, your study doctor will follow your condition every 6 months for a maximum of 5 years from the time you enrolled on the study either in clinic, by phone, or from your medical records."

<u>What possible risks can I expect from taking part in this study?</u>

- In the **Possible Side Effects of Capivasertib (AZD5363)** table, the risks have been updated to reflect information provided in the updated investigator's brochure. Specifically, the following changes have been made:
    - In the Common, Some May be Serious table, "Tiredness" has been added.
    - In the Occasional, Some May be Serious table, "Low red blood cell count in the blood, which can cause fatigue and shortness of breath (anemia). May require a blood transfusion," "Indigestion," "Change in sense of taste," and "Infection which may cause painful and frequent urination" have been added.
    - In the Rare, and Serious table, "Blisters on the skin" has been added.
- In the **Possible Side Effects of Abemaciclib** table, the risks have been updated to reflect information provided in the updated investigator's brochure. Specifically, the following changes have been made:
    - In the Common, Some May be Serious table, "headache" and "abdominal pain" have been added.
    - In the Occasional, Some May be Serious table, "taste changes" has been added.

<u>Who will see my medical information?</u>

In between the 6th and 7th paragraphs, a new subsection heading has been added for clarity that reads: "<u>Alliance for Clinical Trials in Oncology and Alliance Data Innovation Lab</u>." All text after this new subheading has been revised to align with the ICAREdata template language.

A replacement protocol document and model consent form have been issued

---

ATTACH TO THE FRONT OF EVERY COPY OF THIS PROTOCOL
