# Supplementary material for: Abemaciclib in meningiomas with somatic NF2 or CDK pathway alterations: the phase 2 Alliance A071401 trial
Source: Nat Med. 2026 Jan 16;32(2):717–24. doi: 10.1038/s41591-025-04141-4 (PMC12920099; doi:10.1038/s41591-025-04141-4)
Supplement: Supplementary file 2 — Reporting Summary [file 41591_2025_4141_MOESM2_ESM.pdf]

nature portfolio

Corresponding author(s): Priscilla K. Brastianos

Last updated by author(s): 11/2/2025

# Reporting Summary

Nature Portfolio wishes to improve the reproducibility of the work that we publish. This form provides structure for consistency and transparency in reporting. For further information on Nature Portfolio policies, see our Editorial Policies and the Editorial Policy Checklist.

## Statistics

For all statistical analyses, confirm that the following items are present in the figure legend, table legend, main text, or Methods section.

| n/a | Confirmed | |
|---|---|---|
| ☐ | ☒ | The exact sample size (*n*) for each experimental group/condition, given as a discrete number and unit of measurement |
| ☐ | ☒ | A statement on whether measurements were taken from distinct samples or whether the same sample was measured repeatedly |
| ☐ | ☒ | The statistical test(s) used AND whether they are one- or two-sided<br>*Only common tests should be described solely by name; describe more complex techniques in the Methods section.* |
| ☐ | ☒ | A description of all covariates tested |
| ☐ | ☒ | A description of any assumptions or corrections, such as tests of normality and adjustment for multiple comparisons |
| ☐ | ☒ | A full description of the statistical parameters including central tendency (e.g. means) or other basic estimates (e.g. regression coefficient) AND variation (e.g. standard deviation) or associated estimates of uncertainty (e.g. confidence intervals) |
| ☐ | ☒ | For null hypothesis testing, the test statistic (e.g. *F*, *t*, *r*) with confidence intervals, effect sizes, degrees of freedom and *P* value noted<br>*Give P values as exact values whenever suitable.* |
| ☒ | ☐ | For Bayesian analysis, information on the choice of priors and Markov chain Monte Carlo settings |
| ☐ | ☒ | For hierarchical and complex designs, identification of the appropriate level for tests and full reporting of outcomes |
| ☐ | ☒ | Estimates of effect sizes (e.g. Cohen's *d*, Pearson's *r*), indicating how they were calculated |

*Our web collection on statistics for biologists contains articles on many of the points above.*

## Software and code

Policy information about availability of computer code

| | |
|---|---|
| Data collection | Medidata Rave Electronic Data Capture was used to collect the clinical trial data. |
| Data analysis | SAS 9.4 was used for data analysis. |

For manuscripts utilizing custom algorithms or software that are central to the research but not yet described in published literature, software must be made available to editors and reviewers. We strongly encourage code deposition in a community repository (e.g. GitHub). See the Nature Portfolio guidelines for submitting code & software for further information.

## Data

Policy information about availability of data

All manuscripts must include a data availability statement. This statement should provide the following information, where applicable:
- Accession codes, unique identifiers, or web links for publicly available datasets
- A description of any restrictions on data availability
- For clinical datasets or third party data, please ensure that the statement adheres to our policy

De-identified patient data may be requested from Alliance for Clinical Trials in Oncology via concepts@alliancenctn.org if data are not publicly available. Requests are acknowledged within 24 hours of receipt and then sent for internal review. A formal review process includes verifying the availability of data, conducting a review of any existing agreements that may have implications for the project, and ensuring that any transfer is in compliance with the IRB. The investigator will be required to sign a data release form prior to transfer.

# Field-specific reporting

Please select the one below that is the best fit for your research. If you are not sure, read the appropriate sections before making your selection.

☒ Life sciences ☐ Behavioural & social sciences ☐ Ecological, evolutionary & environmental sciences

For a reference copy of the document with all sections, see nature.com/documents/nr-reporting-summary-flat.pdf

# Life sciences study design

All studies must disclose on these points even when the disclosure is negative.

| | |
|---|---|
| Sample size | A total of 24 evaluable patients provided least 85% power to detect a true PFS6 rate of at least 41.5%, with a significance level of .02 against the null hypothesis of 15% PFS6 rate. If at least 8 patients (at least 31.1%) demonstrated PFS6 among the 24 evaluable patients, the agent would be considered worthy of further testing in this mutation-defined grade 2/3 cohort. The PFS6 hypothesis was derived from historical benchmark data from a comprehensive review of prior trials of medical therapies in patients with meningiomas, where PFS6 for grade 2/3 meningiomas ranged from 0 to 29%1. A total of 24 evaluable patients provided at least 89% power to detect a true RR of at least 20%, with a significance level of .021 against the null hypothesis of 2.5% RR. If at least 3 responses (at least 12.5%) were observed among the 24 evaluable patients, the agent would be considered worthy of further testing in this mutation-defined treatment arm. Of note, |
| Data exclusions | Thirty-six patients started treatment. One patient with a grade 1 tumor was registered in error and was considered unevaluable for primary endpoint analyses. The first 24 eligible patients that began treatment were considered evaluable for the analysis of primary endpoint, as per the pre-specified study design |
| Replication | The first 24 eligible patients that began treatment were considered evaluable for the analysis of primary endpoint, as per the pre-specified study design. As the trial did over accrue, demographics, PFS6, RR, as well as secondary endpoints are also reported in all 35 evaluable patients. |
| Randomization | Alliance A071401 is a prospective, multi-arm phase II study evaluating the efficacy of targeted therapies in patients with specific genetic mutations. Each mutation group was designed to be evaluated separately in a phase II study design. A tumor sample from each patient undergoes central pathology review and genetic testing for arm determination. Here we report the results of the abemaciclib treatment arm. Patients with recurrent or progressive grade 2/3 meningiomas harboring CDK pathway or NF2 mutations who met eligibility criteria were enrolled in the abemaciclib arm. Of note, this is a small phase 2 study, as such, we were not powered to do any specific subset analyses based on sex. |
| Blinding | Investigators were not blinded to allocation because each arm of this genomically driven trial was designed as a single arm Phase II study. |

# Reporting for specific materials, systems and methods

We require information from authors about some types of materials, experimental systems and methods used in many studies. Here, indicate whether each material, system or method listed is relevant to your study. If you are not sure if a list item applies to your research, read the appropriate section before selecting a response.

## Materials & experimental systems

| n/a | Involved in the study | |
|---|---|---|
| ☒ | ☐ | Antibodies |
| ☒ | ☐ | Eukaryotic cell lines |
| ☒ | ☐ | Palaeontology and archaeology |
| ☒ | ☐ | Animals and other organisms |
| ☐ | ☒ | Human research participants |
| ☐ | ☒ | Clinical data |
| ☒ | ☐ | Dual use research of concern |

## Methods

| n/a | Involved in the study | |
|---|---|---|
| ☒ | ☐ | ChIP-seq |
| ☒ | ☐ | Flow cytometry |
| ☐ | ☒ | MRI-based neuroimaging |

## Human research participants

Policy information about studies involving human research participants

| | |
|---|---|
| Population characteristics | Across the first 24 patients enrolled to the study, the median age at enrollment was 64 years (range, 39-84), and 14 were female (58%). Most patients had an Eastern Cooperative Oncology Group performance (ECOG) status of 0 or 1 at study entry (21/24 = 88%). Fifteen patients had grade 2 (63%) and 9 (38%) had grade 3 meningioma. All patients had undergone surgery for their meningioma (100%), 23 had received radiation (96%) and 9 had received prior medical therapy (38%). Twenty-three patients (96%) had previously received at least 2 treatment modalities (Table 1). Sex was self-reported and provided by the local investigators as part of registration to the trial. Patients were not excluded based on sex, and analyses were conducted across both sexes. |
| Recruitment | The Alliance for Clinical Trials in Oncology comprises nearly 10,000 cancer specialists at hospitals, medical centers, and |

Recruitment | community clinics across the United States and Canada. Several hundred hospitals have activated this specific trial throughout the US and includes both academic centers and community sites.

Ethics oversight | This Alliance study was designed by the principal investigators and conducted in accordance with the provision of the Declaration of Helsinki and Good Clinical Practice guidelines. The National Cancer Institute Central Institutional Review Board approved the protocol. All patients provided signed informed consent.

Note that full information on the approval of the study protocol must also be provided in the manuscript.

# Clinical data

Policy information about clinical studies

All manuscripts should comply with the ICMJE guidelines for publication of clinical research and a completed CONSORT checklist must be included with all submissions.

Clinical trial registration | Clinicaltrials.gov identifier: NCT02523014

Study protocol | Submitted with manuscript submission

Data collection | During the period that the abemaciclib arm was open from September 15, 2021 to October 3, 2022, 96 patients were screened to the study. The median follow-up since start of treatment was 21 months.

Outcomes | Patients underwent contrast-enhanced brain magnetic response imaging (MRI) every 8 weeks using a consensus MRI protocol. Response was determined by local investigator review using standard Macdonald response criteria28. Progression-free survival at 6 months rate (PFS6) was defined as the number of patients not having progressive disease or death within six months after the first day of treatment divided by the total number of evaluable patients. Response rate (RR) was defined as the number of responses (partial or complete response) divided by the total number of evaluable patients. A patient was deemed to have a response if they had a confirmed partial or complete response.

# Magnetic resonance imaging

## Experimental design

Design type | NA

Design specifications | NA

Behavioral performance measures | NA

## Acquisition

Imaging type(s) | Structural, diffusion

Field strength | 1.5T or 3T

Sequence & imaging parameters | Per at least Minimum Standard from Brain Tumor Imaging Protocol (B. Ellingson, et al., Neuro Oncol 2015 17:1188-98. This includes a minimum of: sagittal or axial 3D T1w pre- and post-gadolinium MPRAGE or equivalent IR-GRE, slice thickness 1.5 mm or less, FOV 256, phase and frequency both 172 or greater, TR 2100, TE minimum, TI 1100, flip angle 10-15; axial 2D FLAIR (3D also allowed), TSE, slice thickness 4 mm or less, FOV 240, phase and frequency 256 or greater, TR >6000, TE 100-140, TI 2000-2500, flip angle 90/160 or more; axial T2-weighted, TSE, slice thickness 4 mm or less, FOV 240, phase and frequency both 256 or greater, TR >2500, TE 80-120, flip angle 90/160 or more.

Area of acquisition | Whole brain

Diffusion MRI ☒ Used ☐ Not used

Parameters | 3 directions or more, b = 0, 500, 1000 s/mm2, no cardiac gating, single shot EPI, slice thickness 4 mm or less, FOV 240, phase and frequency 128 or more, TR >5000, TE minimum, flip angle 90/180

## Preprocessing

Preprocessing software | NA

Normalization | NA

Normalization template | NA

Noise and artifact removal | NA

Volume censoring | NA

## Statistical modeling & inference

| | |
|---|---|
| Model type and settings | NA |
| Effect(s) tested | NA |

Specify type of analysis: ☐ Whole brain ☐ ROI-based ☐ Both

| | |
|---|---|
| Statistic type for inference (See Eklund et al. 2016) | NA |
| Correction | NA |

## Models & analysis

| n/a | Involved in the study |
|---|---|
| ☒ | ☐ Functional and/or effective connectivity |
| ☒ | ☐ Graph analysis |
| ☒ | ☐ Multivariate modeling or predictive analysis |
